# Hydrogen-Bond-Driven Peptide Nanotube Formation: A DFT Study

**DOI:** 10.3390/molecules28176217

**Published:** 2023-08-24

**Authors:** Rubén D. Parra

**Affiliations:** Department of Chemistry and Biochemistry, DePaul University, Chicago, IL 60614, USA; rparra1@depaul.edu

**Keywords:** peptide nanotube, hydrogen bonds, bolaamphiphile tubules, self-assembly, supramolecular chemistry, DFT calculations

## Abstract

DFT calculations were carried out to examine geometries and binding energies of H-bond-driven peptide nanotubes. A bolaamphiphile molecule, consisting of two N-α amido glycylglycine head groups linked by either one CH_2_ group or seven CH_2_ groups, is used as a building block for nanotube self-assembly. In addition to hydrogen bonds between adjacent carboxy or amide groups, nanotube formation is also driven by weak C-H· · ·O hydrogen bonds between a methylene group and the carboxy OH group, and between a methylene group and an amide O=C group. The intratubular O-H· · ·O=C hydrogen bonds account for approximately a third of the binding energies. Binding energies calculated with the wB97XD/DGDZVP method show that the hydrocarbon chains play a stabilizing role in nanotube self-assembly. The shortest nanotube has the length of a single monomer and a diameter than increases with the number of monomers. Lengthening of the tubular structure occurs through intertubular O-H· · ·O=C hydrogen bonds. The average intertubular O-H· · ·O=C hydrogen bond binding energy is estimated to change with the size of the nanotubes, decreasing slightly towards some plateau value near 15 kcal/mol according to the wB97XD/DGDZVP method.

## 1. Introduction

Since its inception about a century ago, the hydrogen bond (H-bond for short) has proven valuable as a conceptual tool for understanding and explaining a great many phenomena occurring in chemistry, biochemistry, and materials science, among other fields [1,2,3,4,5]. Indeed, the ubiquitous presence of hydrogen bonds in biological systems is a testament to the importance of these interactions [6,7]. Defined as an attractive interaction between a hydrogen atom from a molecule or a molecular fragment D-H, in which D is more electronegative than H, and an atom or a group of atoms, A, in the same or a different molecule in which there is evidence of bond formation [8], the hydrogen bond, D-H· · ·A, is a dominant member of the toolbox used by researchers in their quest to design materials tailored to specific functions [9,10,11,12,13,14]. Given its fundamental and practical importance, the hydrogen bond has been the subject of numerous theoretical and experimental studies [15,16,17,18,19,20,21,22]. The results of these studies led to the identification of several characteristics of H-bonds [8]. Two salient characteristics demonstrated in the hydrogen bond interaction are its directionality and its tunability. The latter feature allows for the strength of this interaction to be modulated from weak to very strong interactions. The nature of the hydrogen bond has been shown to change from closed shell to partly covalent depending on the strength of the interaction. In particular, the strength of D-H· · ·A can be modulated by changing either the electron-withdrawing ability of the donor D-H, or the basicity of the H-bond acceptor A. The directionality of the interaction refers to the tendency for linearity in the case of moderate to strong hydrogen bonds. It is important to mention, however, that large deviations from linearity are sometimes observed, especially in the case of weak hydrogen bonds [3]. Because of its long range and flexible directionality, the hydrogen bond allows for bifurcation, wherein the D-H group engages with two equal or different acceptors, A, a case called a bifurcated donor. The case of two D-H groups and one acceptor also occurs (bifurcated acceptor) [2,3,4]. Cooperativity is one more salient feature of the hydrogen bond that makes this interaction noteworthy [5,23,24,25]. Cooperativity makes the whole more than the sum of its parts. Specifically, each one of the individual H-bonds making up a chain of inter-linked H-bonds is more strongly bound than it would be in the absence of the others.

Supramolecular chemistry is one area wherein the attributes of noncovalent interactions, including H-bonds, have been consistently put to good use [26,27,28,29,30,31,32,33]. DNA is a prime example of an H-bond-driven supramolecular assembly that nature itself provides [34]. Water is yet another natural substance imbued with remarkable properties stemming from its ability to engage in a wide array of H-bonds. For example, the self-assembly of water clusters into supramolecular assemblies has been the subject of much research [35,36]. In general, nature provides a plethora of examples of supramolecular architectures built from molecular motifs of varying complexity [37]. Inspired by nature, many researchers have devoted part of their scientific endeavors to refine their understanding of intermolecular interactions and to use that understanding for the design and the development of novel materials with desired properties and functionalities. Some recent examples using H-bonds as building motifs for supramolecular assemblies include the design, synthesis, and studies of H-bonded supramolecular elastomers [38], the use of dynamic supramolecular H-bonding for tunable luminescence [39], and the development of stiff and tough hydrogels [40]. Other examples are seen in the design of organic frameworks of permanent porosity [41], the development and study of supramolecular H-bonded liquid crystals [42], and the self-assembly of H-bonds into supramolecular polymers [43]. Likewise, the design and development of H-bonded supramolecular networks for organic electronic devices [44] and for proton conduction [45,46,47] have been given considerable attention. Some recent research efforts in the field of materials science include, for example, an examination of the many roles played by hydrogen bonds at the catalytic interface of water splitting [48,49,50,51], in composite phase materials [52], and in the performance of both organic and perovskite solar cells [53,54,55].

Studies pertaining to the self-assembly of peptides through hydrogen bonds are particularly relevant to the work presented here. The rich conformational and chemical functionality of peptides make them convenient molecular building blocks for natural supramolecular structures. Inspired by nature, many researchers design, develop, and study unnatural novel materials based on peptide self-assembly. Some applications are found in the fields of medicine, nanotechnology, catalysis, biomaterials, food industry, and others [56,57,58,59,60,61,62,63,64,65,66,67,68,69,70,71,72]. For example, peptide self-assembled nanotube structures have been considered as nanocarriers for the food and pharmaceutical fields [64], and for the treatment of diseases including cancer [66,67,68,69]. The design and use of peptide nanostructures as biosensors [70], catalysts [71], and semiconductors [72] have been reported. Because of their large number of atoms, self-assembled peptides are generally difficult to investigate using theoretical calculations, especially with high level ab initio methods. Many computational studies, nonetheless, have been reported to examine properties of peptide nanostructures [73,74,75,76,77,78,79,80,81,82,83,84,85,86,87,88,89,90,91,92,93,94,95,96]. For example, mathematical conformation analysis along with ab initio Hartree−Fock calculations were used to study possible molecular conformations in peptide nanorings and nanotubes [74]. A combined molecular dynamics and quantum mechanics approach was followed for the theoretical design of cyclic lipopeptide nanotube as a molecular channel across lipid membranes [76]. The effects of the amino acid sequence and solvent polarity on the self-assembling of cyclic peptide nanotubes have also been examined using a combined molecular dynamics and quantum mechanics approach [82]. Using DFT calculations, the ability of alanine-based cyclic peptides to separate phenylalanine enantiomers was investigated in the gas phase and in water [85]. The structure and stability of short β-peptide nanotubes was examined using both the HF/3-21G and the B3LYP/6-31G(d) methods [87]. DFT calculations were used to investigate cyclic peptide nanotubes as novel drug-delivery vehicles [89]. DFT calculations with dispersive corrections were carried out to study the rigidity of diphenylalanine-based nanotubes [91]. Methyl-blocked α,γ-peptide nanotube segments were studied at the B3LYP/6-31G(d) for geometry optimization, and at the B3LYP/6-31+Gd(d) and M05-2X/6-31+G(d) levels for interaction energies [95].

To contribute to the field of peptide nanotubes, this work presents the results of DFT calculations on the nanotube self-assembly of the bolaamphiphilic peptide monomer bis(N-α-amido-glycylglycine)-1,7-heptane dicarboxylate. It is worth noting that a bolaamphiphile contains two hydrophilic head groups at the ends of a hydrophobic chain. The bolaamphiphile chosen for this study, with seven carbons linking two N-α amido glycylglycine head groups, has been reported by Matsui’s research group to organize into peptide nanotubes in acidic solutions [97,98]. For comparative purposes, similar DFT calculations were carried out on the equivalent but simpler bolaamphiphile with just one carbon linking the two glycylglycine head groups. Systematic geometry optimizations on tubular structures of increasing size were performed. Binding energies on the optimized geometries were conducted to assess the relative importance of the various intermolecular interactions, namely amide–amide, and carboxylic acid–carboxylic acid hydrogen bond interactions. The contribution from the noncovalent interactions between hydrocarbon chains was also examined.

## 2. Results and Discussion

### 2.1. (P-CH_2_-P)_n_ Bolaamphiphile Nanotubes

A bolaamphiphile molecule contains two functional head groups connected by a hydrophobic chain. This study focuses on two peptide-based amphiphilic carboxy groups symmetrically placed at the two ends of the molecule. The smaller bolaamphiphile molecule consists of two N-α amido glycylglycine head groups linked by a single CH_2_ group, HOOC-[CH_2_-NHCO]_2_-CH_2_-[CONH-CH_2_]_2_-COOH, or P-CH_2_-P for short. The larger one has seven CH_2_ groups separating the two head groups, P-(CH_2_)_7_-P. For convenience, the former is discussed in this subsection, while the latter is discussed in the next subsection.

**Figure 1:**
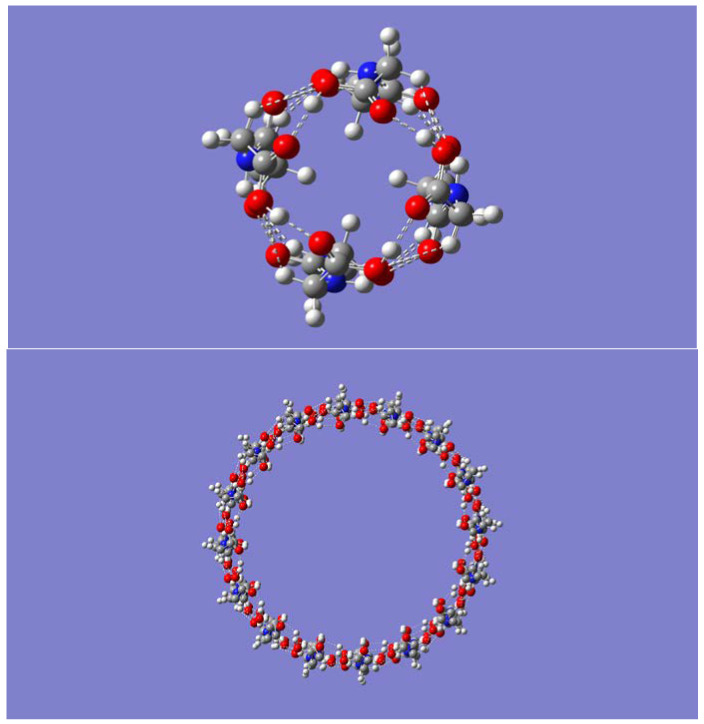
Top view of the B3LYP/6-31G(d) optimized geometries of the smallest (*n* = 4, (**upper panel**)), and largest (*n* = 16, (**lower panel**)) (P-CH_2_-P)_n_ nanotubes considered in this work.

Geometry optimizations showed that formation of tube-like self-assembly (P-CH_2_-P)_n_ structures requires a minimum of four monomers (*n* = 4), or a tetramer. Tubular geometries containing more than four building molecules were also optimized, namely, hexamer, octamer, and decamer. The optimized geometries had C_nh_ symmetry, with *n* being the number of self-assembled molecules. Frequency calculations confirmed that all geometries are indeed minimum energy structures with no imaginary frequencies. Geometry optimizations of even larger sizes, *n* = 12 and *n* = 16 were also carried out. Top views for both the smallest and the largest optimized (P-CH_2_-P)_n_ nanotubes are shown in Figure 1. Inspection of Figure 1 reveals the inner cavity of the tubes, with some hydrogen atoms from the CH_2_ groups pointing inward while others point outward.

**Figure 2:**
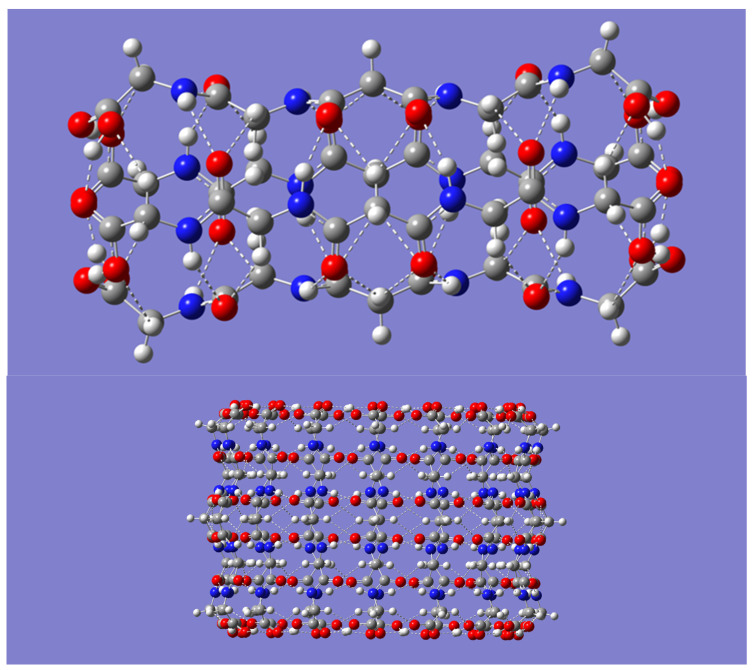
Side view of the B3LYP/6-31G(d) optimized geometry of the smallest (*n* = 4, (**upper panel**)), and largest (*n* = 16, (**lower panel**)) (P-CH_2_-P)_n_ nanotubes considered in this work.

Corresponding side views of the nanotubes are shown in Figure 2. Both Figure 1 and Figure 2 readily reveal that intratubular H-bonds drive the tubular formation, specifically, H-bonds between adjacent molecules involving the amide motifs, N-H· · ·O=C, and those involving the carboxy groups, O-H· · ·O=C. Weaker C-H· · ·O=C bonds with the C-H group as H-bond donor appear to be present as well.

Binding energies and average binding energies for the different (P-CH_2_-P)_n_ nanotubes are listed in Table 1, in kcal/mol. Binding energies, ΔE, are the negative values of the interaction energies. The interaction energies are calculated as the difference between the total energy of the nanotube and the sum of the energies of the individual monomers with the same geometry as they have in the optimized nanotubes. Dividing the binding energy by the number of molecules in the nanotube structure yields the corresponding average binding energy, ΔE/*n*. Table 1 shows that both DFT methods predict large binding energies that increase linearly with the size of the molecules making up the nanotube. The B3LYP method tends to underestimate the calculated binding energies relative to those obtained with the wB97XD method. The gap in the binding energies between the two methods increases with the size of the systems. For example, for *n* = 4, the gap is 59.20 kcal/mol, while, for *n* = 16, the gap is more than three times that amount, 186.27 kcal/mol.

Table 1 also shows that the average binding energies significantly increase as the system size grows from *n* = 4 to *n* = 6, regardless of the DFT method used. A small additional increase is seen at *n* = 8, which is followed by a steady but small decline in the following larger systems. The difference in average binding energies between the two DFT methods becomes somewhat smaller with *n*. Accordingly, the difference decreases from 14.80 to 11.64 kcal/mol as the systems grows from *n* = 4 to *n* = 16.

It is important to notice that the binding energies in Table 1 do not account for the energy cost or deformation energy associated with the change in the geometry from the isolated optimized monomer to the geometry adopted upon nanotube formation. To gauge the magnitude of this deformation energy, the geometry of the free monomer was first optimized at the B3LYP/6-31G(d) level. Corresponding frequency calculations resulted in no imaginary frequencies, confirming the minimum energy nature of the optimized monomer. Single-point energy calculations on the optimized monomer were performed with both the B3LYP/6-31G(d) and the wB97XD/DGDZVP methods. Comparing the energies of the monomer in each nanotube with that of the optimized monomer resulted in a relatively large average deformation energy of 17.1 ± 0.6 kcal/mol with the B3LYP/method. Likewise, the average deformation energy with the wB97XD method is 19.4 ± 0.7 kcal/mol. Thus, the binding energies based on the optimized monomer as a reference point can be obtained by subtracting the average deformation energies from the average binding energies listed in Table 1. Using this approach, for example, the average binding energy for the largest nanotube, *n* = 16, is 23.6 kcal/mol (B3LYP) or 32.9 kcal/mol (wB97XD).

Part of the stability of the nanosystems presented in Table 1 can be attributed to the H-bonding interactions, O-H· · ·O=C, between the carboxy groups of adjacent molecules. The contribution from these interactions may be estimated by calculating the binding energies after removal of the carboxy groups, replacing them with hydrogen atoms and keeping the geometry of each remaining structure as that in the original optimized system. The results shown in Table 2 demonstrate that removal of the carboxy groups greatly reduces the wB97XD binding energies of the nanotubes to about 62% of its original value for the smallest system, and to about 66–70% for all the other system sizes. That is, the O-H· · ·O=C hydrogen bonds account for approximately a third or more of the wB97XD binding energies of the (P-CH_2_-P)_n_ nanotubes. This is also true for the related average binding energies. The B3LYP binding energies of the nanotubes are similarly reduced upon removal of the carboxy groups, namely, to 50% for the tetramer and to about 60–68% for the others.

Geometrical parameters (distances and angles) for the various H-bonds driving the formation of the (P-CH_2_-P)_n_ nanotubes are displayed in Table 3. Inspection of Figure 2, from one end to the center of the nanotube, makes it apparent that any monomer in a nanotube participates in the following six interactions as an H-bond donor or acceptor with adjacent molecules: O-H· · ·O=C, between the carboxy groups; C-H· · ·O-H, between a methylene group and the carboxylic acid OH group; two N-H· · ·O=C, between amide groups; C-H· · ·O=C, between a methylene group and an amide O=C group; and, lastly, a bifurcated C-H· · ·O=C, between the central methylene and amide O=C groups. Note that these interactions repeat, passing from the center all the way to the other end of the nanotube, due to the symmetry of the molecule. Not listed in Table 3 are the dimensions of the nanotubes. Although the length remains constant at about 18 Å, the diameter of the nanotubes increases from about 5 Å in the tetramer to about 22 Å in the hexadecamer.

As seen in Table 3, the lengthening of the interaction distances concomitant with the narrowing of the respective angles for the O-H· · ·O=C hydrogen bonds indicates a weakening of these interactions with the increasing size of the nanotubes. For example, from the smallest to the largest system, this H-bond distance is increased by 0.216 Å, while the angle is reduced by 18.0 degrees. Although its angles change little, the changes in H-bond distances suggest that the bifurcated C-H· · ·O=C interaction weakens with *n*. In contrast, all other H-bonds are strengthened as the size of the nanotube grows, although the rate of change becomes smaller with *n*.

The observations made previously on geometrical grounds can be further supported by examining the calculated electron density at the critical point of each of the H-bonds, ρ_cp_, driving the formation of the (P-CH_2_-P)_n_ nanotubes. The H-bond critical electron densities calculated with the wB97XD/DGDZVP method are shown in Table 4. Inspection of Table 4 reveals, for example, that the weakening of the O-H· · ·O=C interaction is accompanied by a sizeable reduction in the corresponding ρ_cp_ with increasing size of the nanotubes. Moreover, the critical bond density for the bifurcated C-H· · ·O=C interaction exhibits an important decrease as the system grows from *n* = 4 to *n* = 6. This decrease is followed by a steady yet small increase in ρ_cp_ as the system size increases gradually from *n* = 6 to *n* = 16. The results for the bifurcated H-bond are consistent with the geometrical changes mentioned before, namely, a large elongation of the H-bond followed by a comparatively small but consistent reduction in the H-bond distance. The bond critical densities for the other H-bonds tend to increase with the size of the nanotube, with some already nearing plateau. Lastly, the H-bond between a methylene group and the carboxylic acid OH group appears so weak when *n* = 4 that it actually lacks the corresponding H-bond critical point. Nonetheless, this C-H· · ·O-H interaction does gain considerable strength with increasing size of the nanotube, as demonstrated by the presence of the H-bond critical point for *n* > 4. Geometrically, the very weak C-H· · ·O-H interaction when *n* = 4 is consistent with a relatively large H-bond distance and narrow angle (Table 3). For *n* > 4, this H-bond distance consistently shortens, while the H-bond angle widens greatly, indicating that the H-bond is indeed becoming stronger. 

### 2.2. (P-(CH_2_)_7_-P)_n_ Bolaamphiphile Nanotubes

The self-assembly into nanotubes of the larger bolaamphiphile molecule is discussed in this section. The building molecules consist of two N-α amido glycylglycine head groups linked by seven methylene groups, HOOC-[CH_2_-NHCO]_2_-(CH_2_)_7_-[CONH-CH_2_]_2_-COOH, or P-(CH_2_)_7_-P for short. Matsui and Gologan reported that, in two weeks and at pH 4, this heptane bolaamphiphile undergoes self-assembly into a crystalline tubule [97]. These authors suggested an assembly of the building motifs into a planar multilayer of intermolecular H-bonds between adjacent amide groups, accompanied by H-bonds between carboxy groups that bring two monomers together, extending the system in the direction perpendicular to that of the amide–amide H-bonds. At low pH, the planar multilayer curls tightly into tubular shapes.

**Figure 3:**
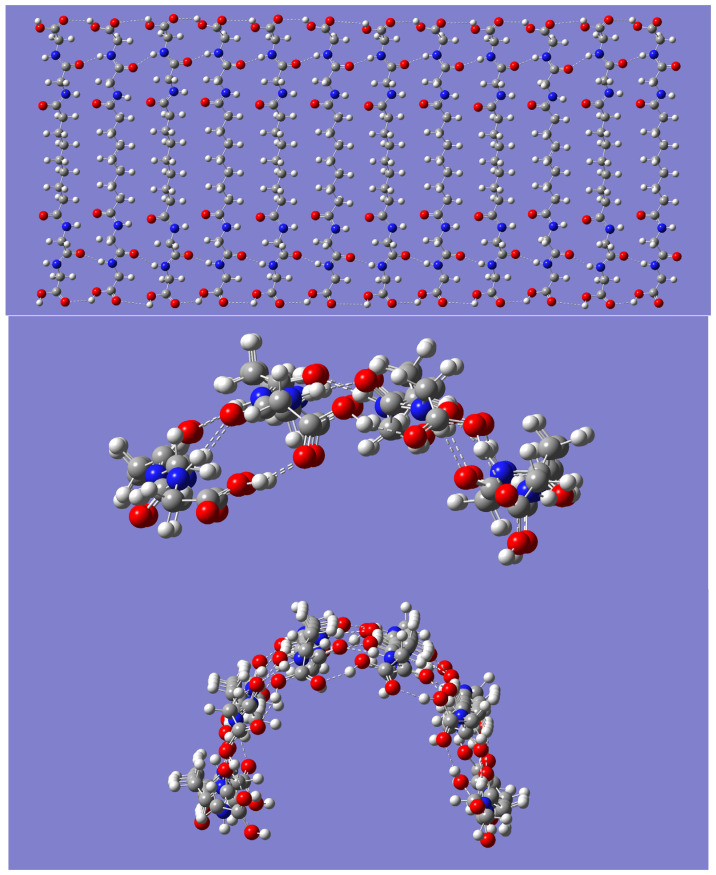
Initial guess geometry for B3LYP/6-31G(d) optimization of the (P-(CH_2_)_7_-P)_n_ systems ((**top panel**) shows *n* = 12); resulting optimized geometry for *n* = 4 (**middle panel**) and for *n* = 6 (**bottom panel**).

A sheet-like arrangement of up to 12 P-(CH_2_)_7_-P monomers, positioned relative to one another as shown in Figure 3 (top panel), was built as the initial guess for geometry optimization. It is important to note that, in this approach, the H-bonds between carboxy groups run parallel with the H-bonds between amide groups. Consequently, the idea is first to examine the possible folding of the initial sheet-like geometry into nanotubes with the length of the given monomer or building block, along with a diameter that increases with the number of monomers in the system. The tubular lengthening via carboxylic acid H-bonds is discussed in the next subsection. Geometry optimizations for the smaller (P-(CH_2_)_7_-P)_n_ systems of size *n* = 4 and *n* = 6 resulted in minimum energy structures with partial folding, as shown in the middle and bottom panels, respectively, in Figure 3.

**Figure 4:**
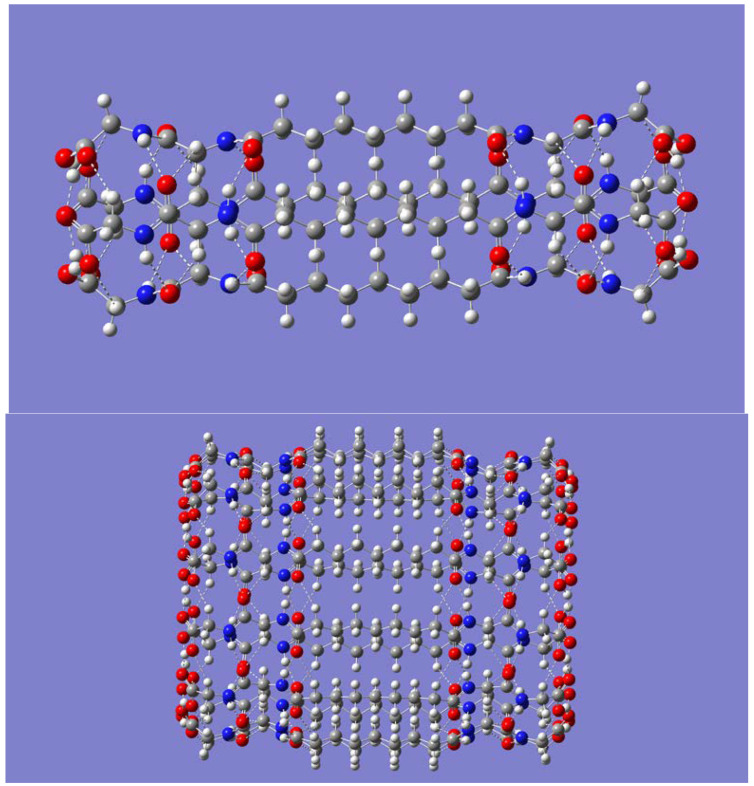
Side view of the B3LYP/6-31G(d) optimized geometry of the (P-(CH_2_)_7_-P) tetramer (**upper panel**) and dodecamer (**lower panel**).

For the larger-size monomers, geometry optimizations resulted in complete tubular folding. It was, nonetheless, possible to obtain the tubular shapes for the *n* = 4 and *n* = 6 systems by using a guess geometry built by bringing the corresponding end monomers in the optimized structures shown in Figure 3 closer. The open structure for *n* = 4 is just 2.18 kcal/mol lower in energy than the completely folded one. In sharp contrast, the open structure for *n* = 6 is much higher in energy (by 39.48 kcal/mol) than the completely folded one. In general, the optimized self-assembled (P-(CH_2_)_7_-P)_n_ nanotubes for *n* = 4, 6, 8, and 10 resulted in structures of symmetry C_nh_, with *n* being the number of self-assembled molecules. Frequency calculations confirm that all these optimized geometries, including the partially folded ones, are indeed minimum energy structures with no imaginary frequencies. Likewise, geometry optimization for the larger dodecamer was also successfully carried out. Side views of the optimized geometries are shown in Figure 4 for the nanotubes of sizes *n* = 4 and *n* = 12. Figure 4 shows the H-bonds driving the tubular formation. Not surprisingly, these are the same type of interactions seen in the smaller nanotubes (P-CH_2_-P)_n_ discussed earlier. One difference that is worthy of notice is the potential for the dispersion interactions in adjacent molecules emerging from the longer hydrocarbon chain in each P-(CH_2_)_7_-P monomer.

Binding energies and average binding energies for the different (P-(CH_2_)_7_-P)_n_ nanotubes of size *n* are listed in Table 5. The results parallel those of the smaller (P-CH_2_-P)_n_ nanotubes (Table 1). For example, Table 5 shows that both DFT methods predict large binding energies that increase linearly with the size of the molecules making up the nanotube, and that the B3LYP method underestimates the calculated binding energies relative to those obtained with the wB97XD method. Moreover, the increase in the average binding energies seems to level off rather quickly, and the difference in average binding energies between the two DFT methods becomes smaller with *n*. To obtain the average binding energies using the geometry of the optimized monomer as a reference point, the deformation energies were calculated for the monomer with the geometry in each of the nanotubes. For the smallest nanotube, the deformation energy is 10.53 kcal/mol (B3LYP) or 11.22 kcal/mol (wB97XD). The corresponding values for the other nanotubes, *n* > 4, are somewhat larger, but much closer to one another, with an average deformation energy of 13.0 ± 0.1 kcal/mol (B3LYP) or 13.6 ± 0.1 kcal/mol (wB97XD). Despite the relatively large deformation energies, the average binding energies remain significant. For example, the average binding energy for the largest nanotube, *n* = 12, is 26.6 kcal/mol (B3LYP) or 40.6 kcal/mol (wB97XD) after accounting for the deformation energy.

Table 6 displays the difference between the binding energies (and average binding energies) of the (P-(CH_2_)_7_-P)_n_ nanotubes and those of the (P-CH_2_-P)_n_ nanotubes. The results are obtained by subtracting the values listed in Table 1 from the related ones in Table 5. These changes in binding energies are important because they help provide insight into the role played by the hydrocarbon chain in stabilizing the nanotubes. The negative changes in binding energies seen in Table 6 for the B3LYP method suggest that the hydrocarbon chain plays a destabilizing role; the opposite, however, is true for the wB97XD method, which shows corresponding positive changes in the binding energies. The inability of the B3LYP hybrid functional to account for dispersion interactions helps explain the negative changes in binding energies. On the other hand, the wB97XD method is better equipped to capture the important dispersion interactions taking place among the hydrocarbon chains of adjacent monomers. Interestingly, the B3LYP change in the average binding energy remains basically constant for the larger nanotubes. In contrast, the corresponding changes with the wB97XD method show a steady decrease with increasing system size, although this change seems to stabilize at about 0.6 kcal/mol for the largest *n*.

Given that the various types of H-bond interactions driving the formation of both (P-CH_2_-P)_n_ and (P-(CH_2_)_7_-P)_n_ nanotubes are basically the same, it was of interest to examine how the presence of the longer hydrocarbon chain modifies the geometrical parameters (distances and angles) for these interactions. Table 7 shows changes in the geometrical parameters listed in Table 3 upon addition of the longer hydrocarbon chain for nanotubes of size up to *n* = 12. Examination of Table 7 reveals that changes are minimal, within the second and third decimal place, for most H-bond distances. Larger changes, within the first decimal place, occur for the C-H· · ·O=C hydrogen bonds which were originally involved in bifurcated H-bonds. Changes in corresponding H-bond angles are also small, within the tenths, for half of the H-bonds, and within the ones for the other half. Again, larger angle changes are seen in the originally bifurcated C-H· · ·O=C hydrogen bond and the N-H· · ·O=C closer to it. Lastly, the lengths of the (P-(CH_2_)_7_-P)_n_ nanotubes lie between 26 and 25 Å, that is, about 8 Å longer than in their smaller (P-CH_2_-P)_n_ counterparts.

The H-bond critical electron densities calculated with the wB97XD/DGDZVP method for the (P-(CH_2_)_7_-P)_n_ nanotubes are shown in Table 8. Cross-examination of Table 8 and Table 4 shows that the calculated H-bond critical bond densities are quite similar (within the third or fourth decimal place) for both the larger and smaller bolaamphiphiles considered here, in agreement with the similar geometrical characteristics discussed above.

### 2.3. Intertubular Elongation through O-H· · ·O=C Hydrogen Bonds

**Figure 5:**
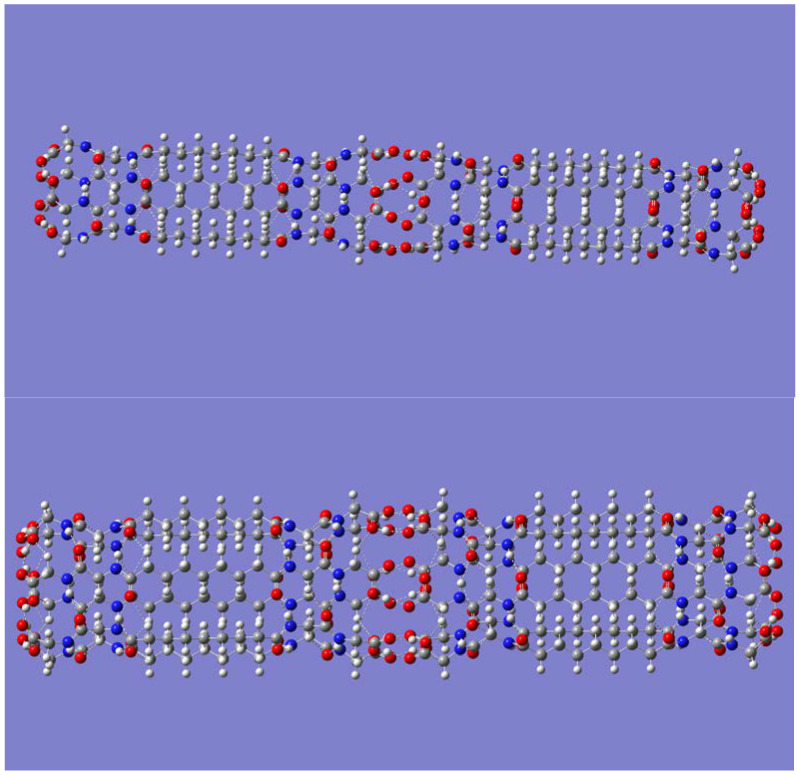
Side view of the B3LYP/6-31G(d) optimized geometry of the (P-(CH_2_)_7_-P)_4_ dimer (**upper panel**) and the (P-(CH_2_)_7_-P)_6_ dimer (**lower panel**).

Each nanotubular assembly of the (P-(CH_2_)_7_-P)_n_ bolaamphiphile peptide could in principle be extended via intertubular O-H· · ·O=C hydrogen bond interactions, as proposed by Matsui and Gologan [97]. That is, the H-bonds between adjacent carboxy groups in each parent nanotube are disrupted and replaced with the same type of H-bonds but now between adjacent parent nanotubes, which would result in a tubular elongation. To test this hypothesis, a geometry optimization of two (P-(CH_2_)_7_-P)_n_ nanotubes of size *n* = 4 was first carried out. The two nanotubes were positioned relative to each other, so as to favor O-H· · ·O=C interactions between the tubes. Figure 5 shows that the geometry optimization successfully culminated in an elongated nanotube about 54 Å long (slightly more than twice the length of the parent nanotube, which is about 26 Å). The corresponding geometry optimization of two nanotubes of size *n* = 6 also resulted in tubular elongation through the O-H· · ·O=C hydrogen bonds (Figure 5). BSSE-corrected dimer binding energies were calculated at the B3LYP/6-31G(d) and wB97XD/DGDZVP levels for each of the nanotube dimers. The B3LYP results were 67.71 and 84.34 kcal/mol for the smaller and larger nanotube dimer systems, respectively. Dividing these dimer binding energies by *n* gives an average dimer binding energy of 16.93 and 14.06 kcal/mol for *n* = 4 and *n* = 6, respectively. The wB97XD/DGDZVP method resulted in binding energies of 72.83 and 93.86 kcal/mol and related average binding energies of 18.21 and 15.64 kcal/mol for *n* = 4 and *n* = 6, respectively. Although B3LYP somewhat underestimates the H-bond binding energies when compared with wB97XD, both methods agree that the intertubular O-H· · ·O=C hydrogen bonds provide a significant contribution to the stability of the elongated nanotubes. Intertubular growth is then expected to continue, which would progressively extend the length of the nanotubular structures. Because of the large computational cost, it becomes impractical to examine whether the decrease in the average dimer binding energies seen as *n* increases from 4 to 6 will continue for larger values of *n*.

**Figure 6:**
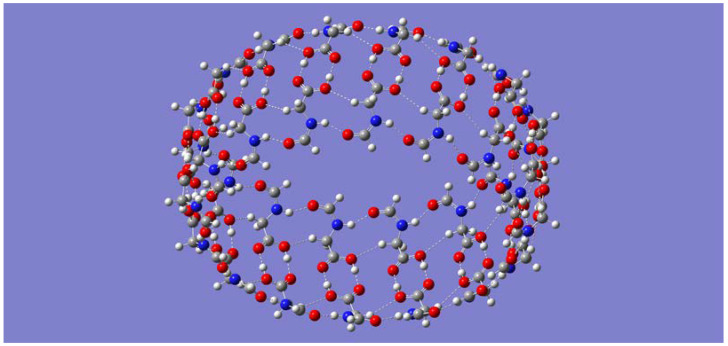
B3LYP/6-31G(d) optimized geometry of the smaller (modified) nanotube of dimer of size *n* = 16.

To estimate the extent to which the O-H· · ·O=C hydrogen bond binding energies change with the size of the nanotubes upon dimer elongation, geometry optimization for smaller systems was performed. Specifically, only the region containing the intertubular H-bonds and the closest intratubular N-H· · ·O=C hydrogen bonds was considered. This approach significantly reduces the size of each system. Accordingly, the geometries of systems of size *n* = 4, 6, 10, and 16 were optimized. In particular, the B3LYP/6-31G(d) optimized geometry of the *n* = 16 system is displayed in Figure 6.

Frequency calculations consistently show no imaginary frequencies, which confirms the minimum energy structure nature of the optimized dimer geometries for all *n* values considered. BSSE-corrected dimer binding (and average binding) energies calculated at the B3LYP/6-31G(d) and wB97XD/DGDZVP levels for each of the modified nanotube dimers are listed in Table 9. It is worth noting that the results listed in Table 9 for the smaller *n* = 4 and *n* = 6 size dimer systems are close to those obtained for the larger (P-(CH_2_)_7_-P)_n_ dimer counterparts discussed previously. The results listed in Table 9 indicate that, as *n* increases, the average dimer binding energy does indeed decrease. However, this decrease in dimer binding energy tends towards some plateau value near 13.35 or 14.91 kcal/mol, depending on the DFT method used.

## 3. Computational Methods

All calculations were performed using the Gaussian 16 program [99]. Geometry optimization and frequency calculations were conducted with the B3LYP/6-31G(d) method. Binding energies, corrected for basis set superposition error [100], were performed with both the B3LYP/6-31G(d) and the wB97XD/DGDZVP methods on the B3LYP/6-31G(d) geometries. Optimized geometries for the (P-CH_2_-P)_n_ and (P-(CH_2_)_7_-P)_n_ are provided in Supplementary Material Files S1 and S2, respectively. Optimized geometries for the nanotube dimers are provided as Supplementary Material File S3.

Historically, B3LYP has been one of the most widely used DFT methods for geometry optimization of molecules and the study of H-bonded complexes, including peptide self-assemblies [85,87,89,91,95,101,102]. Known limitations of the B3LYP hybrid functional include the underestimation of reaction barriers, and the unreliable description of isomerization energies. Moreover, B3LYP tends to give bond dissociation enthalpies that are too low when compared with experiments. Lastly, a perhaps more pertinent limitation of the B3LYP method is its inability to calculate binding energies accurately, particularly in cases where dispersion interactions are important. Efforts to improve upon the known limitations of B3LYP have been reported [103], and, especially, DFT methods that include dispersion corrections are found to be generally desired [104,105,106] when calculating intermolecular or non-bonded interaction energies that contain an important van der Waals or dispersion energy component. Accordingly, the wB97XD functional, which includes the empirical Grimme’s D2 dispersion method as well as long-range corrections [107,108,109,110,111], is also used here for binding energy calculations. 

## 4. Conclusions

Nanotube self-assembly of the bolaamphiphile HOOC-[CH_2_-NHCO]_2_-(CH_2_)_7_-[CONH-CH_2_]_2_-COOH (P-(CH_2_)_7_-P) is shown to occur through a network of intratubular H-bond interactions. Indeed, an initial guess geometry, consisting of a sheet-like arrangement of several P-(CH_2_)_7_-P building monomers, folds into a nanotube upon full geometry optimization [97,98]. The length of the resulting nanotube is that of the building monomer (~18 Å), and the diameter increases linearly with the number of monomers. Any given monomer in a nanotube participates in the following interactions as an H-bond donor or acceptor with adjacent molecules: O-H· · ·O=C, between the carboxy groups; C-H· · ·O-H, between a methylene group and the carboxylic acid OH group; N-H· · ·O=C, between amide groups; and C-H· · ·O=C, between a methylene group and an amide O=C group. The two DFT methods used for calculating binding energies yield similar qualitative results. Compared with the wB97XD method, however, the B3LYP method underestimates the calculated binding energies. This result is not surprising, given the known limitation of B3LYP regarding the calculation of non-bonded interaction energies. The underestimation of binding energies occurs for both the (P-(CH_2_)_7_-P)_n_ and the P-(CH_2_)-P)_n_ nanotubes investigated here, though it becomes a bit more severe in the (P-(CH_2_)_7_-P) nanotubes due to the interactions among the hydrocarbon chains. Furthermore, the contribution to the binding energies from the interactions among adjacent hydrocarbon chains was estimated by comparing the binding energies of the (P-(CH_2_)_7_-P)_n_ and the P-(CH_2_)-P)_n_ nanotubes. The B3LYP method predicts a destabilization, while the wB97XD method predicts a stabilizing contribution from the inter-hydrocarbon chain interaction. The result highlights the limitation of the B3LYP method to account for the attractive van der Waals or dispersion interactions and, hence, the need for using DFT methods that incorporate dispersion interactions. Lastly, the gap in the binding energies between the two methods increases with the size of the systems, a result consistent with another known limitations of B3LYP, i.e., its tendency to degrade the quality of the results with the size of the system [95].

The length of the self-assembled nanotubes increases via intertubular H-bonding of carboxy groups between adjacent parent nanotubes in a manner akin to that suggested by Matsui and Gologan [97]. The intertubular O-H· · ·O=C hydrogen bond binding energies are estimated to change with the size of the nanotubes upon dimer elongation. Specifically, as *n* increases, the average dimer binding energy decreases towards some plateau value near 13.35 or 14.91 kcal/mol, depending on the DFT method used. Although only small nanotube dimers were considered, intertubular H-bonds between carboxy groups are expected to extend progressively the length of the nanotubular structures.

In short, the results of this work provide support for the notion that bolaamphiphile molecules can be used as building blocks to design and develop self-assembled nanotube structures of controllable length and diameter. Although the bolaamphiphiles chosen for this study contain two N-α amido glycylglycine head groups separated by one or seven methylene groups, it is conceivable to have different or modified head groups bridged by potentially any number of methylene groups. Current research in the author’s lab includes the formation of self-assembled nanotubes and other supramolecular structures based on modified glycylglycine headgroup motifs. For example, azelaic acid, which has two carboxy groups separated by seven methylene groups, is currently being investigated in the author’s lab. Azelaic acid can be thought of as resulting from the removal of both [CH_2_-NHCO]_2_ groups in HOOC-[CH_2_-NHCO]_2_-(CH_2_)_7_-[CONH-CH_2_]_2_-COOH. Given the B3LYP limitations for the accurate calculation of binding energies, DFT methods that explicitly account for dispersion interactions are recommended, especially for those systems where dispersion interactions contribute to the stability of the supramolecular structures. 

## Figures and Tables

**Table 1:** Binding energies, ΔE, of the various (P-CH_2_-P)_n_ nanotubes calculated at both the B3LYP/6-31G(d) and the wB97XD/DGDZVP levels on the B3LYP/6-31G(d) optimized geometries. Also listed are the average binding energies, ΔE/*n*, where *n* is the size of the nanotube. Values are listed in kcal/mol.

	B3LYP		wB97XD	
Size, *n*	ΔE	ΔE/*n*	ΔE	ΔE/*n*
4	120.62	30.16	179.82	44.96
6	244.42	40.74	323.49	53.92
8	341.29	42.66	437.95	54.74
10	426.44	42.64	543.57	54.36
12	505.22	42.10	644.38	53.70
16	651.23	40.70	837.50	52.34

**Table 2:** Binding energies, ΔΕ, of the (P-CH_2_-P)_n_ nanotubes upon removal of the carboxy groups of the B3LYP/6-31G(d) optimized geometries. Also listed are the average binding energies, ΔE/*n*, where *n* is the size of the nanotube. Binding energy values, in kcal/mol, calculated at both the B3LYP/6-31G(d) and the wB97XD/DGDZVP levels.

	B3LYP		wB97XD	
Size, *n*	ΔE	ΔE/*n*	ΔE	ΔE/*n*
4	60.01	15.00	111.39	27.85
6	146.70	24.45	212.57	35.43
8	211.98	26.50	290.75	36.34
10	271.89	27.19	365.77	36.58
12	329.65	27.47	439.67	36.64
16	442.36	27.65	586.39	36.65

**Table 3:** B3LYP/6-31G(d) optimized geometrical parameters of the different H-bonds driving the formation of the (P-CH_2_-P)_n_ nanotubes. Distances in Å, angles in degrees.

			Distances			
Size, *n*	O-H· · ·O=C	C-H· · ·O-H	N-H· · ·O=C	N-H· · ·O=C	C-H· · ·O=C	C-H· · ·O=C
4	1.723	2.824	2.651	2.056	2.503	2.546
6	1.724	2.548	2.023	1.916	2.310	2.671
8	1.761	2.432	1.933	1.900	2.320	2.650
10	1.808	2.373	1.898	1.898	2.323	2.634
12	1.853	2.338	1.881	1.898	2.320	2.624
16	1.939	2.307	1.865	1.896	2.311	2.608
			**Angles**			
**Size, *n***	**O-H** **· · ·O=C**	**C-H** **· · ·O-H**	**N-H** **· · ·O=C**	**N-H** **· · ·O=C**	**C-H** **· · ·O=C**	**C-H** **· · ·O=C**
4	155.7	123.1	143.2	150.0	123.0	141.3
6	155.1	128.6	161.4	165.6	144.0	140.6
8	151.2	131.4	165.6	170.1	146.0	141.4
10	147.1	133.0	166.9	172.2	146.2	141.8
12	143.5	134.3	167.1	173.3	146.2	142.0
16	137.7	136.1	166.9	173.9	146.0	142.1

**Table 4:** wB97XD/DGDZVP electron density values, ρ_CP_ (a.u.), calculated at H-bond critical points on the B3LYP/6-31G(d) optimized geometries of (P-CH_2_-P)_n_ nanotubes.

			ρ_CP_			
Size, *n*	O-H· · ·O=C	C-H· · ·O-H	N-H· · ·O=C	N-H· · ·O=C	C-H· · ·O=C	C-H· · ·O=C
4	0.0410	--	0.0051	0.0193	0.0094	0.0079
6	0.0413	0.0071	0.0193	0.0257	0.0135	0.0061
8	0.0382	0.0091	0.0236	0.0262	0.0132	0.0063
10	0.0343	0.0104	0.0256	0.0261	0.0130	0.0065
12	0.0308	0.0112	0.0266	0.0259	0.0130	0.0067
16	0.0254	0.0121	0.0276	0.0258	0.0131	0.0070

**Table 5:** Binding energies, ΔE, of the various (P-(CH_2_)_7_-P)_n_ nanotubes calculated at both the B3LYP/6-31G(d) and the wB97XD/DGDZVP levels on the B3LYP/6-31G(d) optimized geometries. Also listed are the average binding energies, ΔE/*n*, where *n* is the size of the nanotube. Values are listed in kcal/mol.

	B3LYP		wB97XD	
Size, *n*	ΔE	ΔE/*n*	ΔE	ΔE/*n*
4	107.05	26.76	186.73	46.68
6	228.75	38.13	330.23	55.04
8	320.42	40.05	443.85	55.48
10	400.48	40.05	549.64	54.96
12	475.09	39.59	650.80	54.23

**Table 6:** Difference in binding energies, Δ(ΔE), of the various (P-(CH_2_)_7_-P)_n_ nanotubes and those of the (P-(CH_2_)-P)_n_ nanotubes, calculated at both the B3LYP/6-31G(d) and the wB97XD/DGDZVP levels on the B3LYP/6-31G(d) optimized geometries. Also listed are the corresponding difference in average binding energies, Δ(ΔE)/*n*, where *n* is the size of the nanotube. Values are listed in kcal/mol.

	B3LYP		wB97XD	
Size, *n*	Δ(ΔE)	Δ(ΔE)/*n*	Δ(ΔE)	Δ(ΔE)/*n*
4	−13.57	−3.39	6.91	1.73
6	−15.67	−2.61	6.74	1.12
8	−20.87	−2.61	5.90	0.74
10	−25.96	−2.60	6.07	0.61
12	−30.13	−2.51	6.42	0.64

**Table 7:** Changes in the B3LYP/6-31G(d) optimized geometrical parameters of the different H-bonds in (P-(CH_2_)_7_-P)_n_ nanotubes relative to (P-CH_2_-P)_n_ nanotubes. Changes in distances are in Å, while changes in angles are in degrees.

			Distances			
Size, *n*	O-H· · ·O=C	C-H· · ·O-H	N-H· · ·O=C	N-H· · ·O=C	C-H· · ·O=C	C-H· · ·O=C
4	−0.003	−0.034	−0.061	0.176	0.050	−0.069
6	−0.004	−0.015	0.001	0.075	0.023	−0.155
8	−0.006	−0.013	0.006	0.074	0.016	−0.155
10	−0.010	−0.013	0.008	0.077	0.010	−0.151
12	−0.013	−0.015	0.009	0.079	0.010	−0.151
			**Angles**			
**Size, *n***	**O-H** **· · ·O=C**	**C-H** **· · ·O-H**	**N-H** **· · ·O=C**	**N-H** **· · ·O=C**	**C-H** **· · ·O=C**	**C-H** **· · ·O=C**
4	−0.3	0.4	−0.2	−5.7	−3.9	4.4
6	0.1	0.2	−0.3	−5.7	−2.9	4.0
8	0.2	0.1	−0.2	−7.1	−2.1	3.8
10	0.3	0.1	−0.3	−8.0	−1.6	3.6
12	0.5	0.0	−0.2	−8.4	−1.3	3.7

**Table 8:** wB97XD/DGDZVP electron density values, ρ_CP_ (a.u.), calculated at H-bond critical points on the B3LYP/6-31G(d) optimized geometries of (P-(CH_2_)_7_-P)_n_ nanotubes.

			ρ_CP_			
Size, *n*	O-H· · ·O=C	C-H· · ·O-H	N-H· · ·O=C	N-H· · ·O=C	C-H· · ·O=C	C-H· · ·O=C
4	0.0413	--	0.0058	0.0135	0.0088	0.0087
6	0.0422	0.0073	0.0193	0.0221	0.0130	0.0080
8	0.0388	0.0094	0.0233	0.0225	0.0128	0.0083
10	0.0350	0.0107	0.0251	0.0222	0.0127	0.0085
12	0.0318	0.0116	0.0261	0.0219	0.0127	0.0087

**Table 9:** Dimer binding energies, ΔE, of the various modified nanotube dimers calculated at both the B3LYP/6-31G(d) and the wB97XD/DGDZVP levels on the B3LYP/6-31G(d) optimized geometries. Also listed are the corresponding average binding energies, ΔE/*n*, where *n* is the size of the individual nanotube. Values are listed in kcal/mol.

	B3LYP		wB97XD	
Size, *n*	ΔE	ΔE/*n*	ΔE	ΔE/*n*
4	61.50	15.38	67.20	16.80
6	86.63	14.44	95.06	15.84
8	112.54	14.07	123.93	15.49
10	138.53	13.85	153.00	15.30
16	213.65	13.35	238.54	14.91

## Data Availability

Not applicable.

## Supplementary Materials

The following supporting information can be downloaded at: https://www.mdpi.com/article/10.3390/molecules28176217/s1, File S1: Optimized (P-CH_2_-P)_n_ geometries; File S2: Optimized (P-(CH_2_)_7_-P)_n_ geometries; File S3: Optimized dimer geometries.
