# Supplementary material for: Hydrogen-Bond-Driven Peptide Nanotube Formation: A DFT Study
Source: Molecules. 2023 Aug 24;28(17):6217. doi: 10.3390/molecules28176217 (PMC10488343; doi:10.3390/molecules28176217)
Supplement: Supplementary file 1 [file molecules-28-06217-s001.zip › S1.pdf]

Optimized [P-CH_2-P]$_n$ Nanotubes, (*n* = 4, 6, 8, 10, 12, 16)

[P-CH_2-P]$_4$

C	0.00000000	0.00000000	0.00000000
O	0.00000000	0.00000000	1.23066777
C	3.69086554	0.00000000	2.52950863
O	4.92059765	0.04797557	2.53016739
C	3.84446768	-9.68335878	2.30538414
O	5.07510274	-9.69230681	2.30472538
C	0.15360215	-9.68335878	-0.22412449
O	0.15450510	-9.74028237	1.00522577
C	2.52851344	0.12550118	-3.68941344
O	1.29787838	0.13444921	-3.68875468
C	2.68211559	-9.55785759	-3.91353792
O	1.45238348	-9.60583316	-3.91419668
C	6.21937899	0.12550118	-1.15990481
O	6.21847604	0.18242478	-2.38925506
C	6.37298113	-9.55785759	-1.38402930
O	6.37298113	-9.55785759	-2.61469707
H	-0.11502764	-10.62096806	-2.03228759
H	-0.29954359	1.01125367	-1.76305603
H	1.88964965	0.93954572	2.83545322
H	2.07416560	-10.69267601	2.56622166
H	4.29881552	1.13481842	-3.95025096
H	4.48333147	-10.49740332	-4.21948252
H	6.48800877	1.06311047	0.64825829
H	6.67252472	-10.56911127	0.37902673
C	0.65262201	-8.46957546	-1.00014582
H	1.74424048	-8.59151432	-1.08335472
H	0.27811210	-8.46031265	-2.02367004
C	0.53667651	-1.16016033	-0.83096703
H	0.16189758	-1.13393820	-1.85409872
H	1.62381956	-0.99996024	-0.90764573
C	2.90589548	-1.18773117	1.98396620
H	1.88242513	-1.20414551	2.35837816
H	2.82330281	-1.02272515	0.89797580
C	3.02184098	-8.49714630	1.81478741

H	2.94372373	-8.61427924	0.72226680
H	1.99863964	-8.53051996	2.18880684
C	3.35114014	-1.06071129	-3.19881671
H	4.37434148	-1.02733763	-3.57283614
H	3.42925739	-0.94357835	-2.10629610
C	3.46708564	-8.37012642	-3.36799549
H	3.54967832	-8.53513244	-2.28200509
H	4.49055600	-8.35371209	-3.74240745
C	5.72035912	-1.08828213	-0.38388348
H	6.09486904	-1.09754494	0.63964075
H	4.62874065	-0.96634327	-0.30067457
C	5.83630462	-8.39769726	-0.55306227
H	4.74916157	-8.55789735	-0.47638357
H	6.21108355	-8.42391939	0.47006942
C	0.13759887	-3.52117534	-1.12919041
O	0.26239436	-3.38968352	-2.34815788
C	2.74092872	-6.15137890	2.30576157
O	1.52100419	-6.26903902	2.17658746
C	2.69984557	-3.56142228	2.36570696
O	1.47670987	-3.47664407	2.24121835
C	0.17868203	-6.11113197	-1.18913579
O	0.30668870	-6.18207847	-2.41278876
C	3.67313556	-5.99643532	-3.74973625
O	4.89627126	-6.08121353	-3.62524764
C	3.63205240	-3.40647869	-3.68979087
O	4.85197693	-3.28881858	-3.56061676
C	6.23538225	-6.03668225	-0.25483889
O	6.11058676	-6.16817407	0.96412857
C	6.19429910	-3.44672562	-0.19489350
O	6.06629244	-3.37577912	1.02875946
C	-0.23840414	-4.83886484	-0.44968954
H	0.12238239	-4.85707844	0.58449657
H	-1.33661871	-4.85750524	-0.39697970
C	3.43010703	-4.85432555	2.73248295
H	4.46430766	-4.82958001	2.37212717
H	3.48322740	-4.87889831	3.83056103

C	2.94287409	-4.70353205	-4.11651225
H	1.90867346	-4.72827760	-3.75615646
H	2.88975372	-4.67895930	-5.21459032
C	6.61138526	-4.71899277	-0.93433976
H	6.25059873	-4.70077916	-1.96852588
H	7.70959984	-4.70035237	-0.98704960
N	0.23714757	-2.46098519	-0.27557536
N	3.58677128	-7.21306050	2.16450186
N	3.51150384	-2.46806407	2.27432634
N	0.31241501	-7.20598162	-0.38539985
N	2.86147729	-7.08979353	-3.65835563
N	2.78620984	-2.34479710	-3.54853115
N	6.13583356	-7.09687240	-1.10845394
N	6.06056612	-2.35187597	-0.99862945
H	4.51307120	-2.58786930	2.12201941
H	4.58392544	-7.05464923	2.01863433
H	0.45968359	-7.08763084	0.61709352
H	0.38882935	-2.62085090	0.72047859
H	1.85990992	-6.96998830	-3.50604870
H	1.78905568	-2.50320836	-3.40266363
H	5.98415178	-6.93700669	-2.10450789
H	5.91329754	-2.47022676	-2.00112281
N	-0.21982013	-10.71911980	-1.02812617
N	-0.40665804	1.05948233	-0.75550659
N	2.89452589	1.02623365	2.94345482
N	3.08136380	-10.75236848	2.67083523
N	3.29161733	1.19451089	-4.05486453
N	3.47845524	-10.58409124	-4.32748411
N	6.59280126	1.16126220	-0.35590313
N	6.77963917	-10.61733992	-0.62852271
C	3.65935250	-12.04932300	2.97658933
H	3.43462543	-12.34184472	4.01177613
H	4.74070280	-11.96919481	2.86712851
C	3.43133482	2.32532736	3.30929530
H	3.19820733	2.56238320	4.35673935
H	4.51460112	2.28466709	3.19703879

C	-0.53487067	-12.03536449	-0.50097288
H	-1.57221784	-12.31136812	-0.73637845
H	-0.42455705	-11.99825299	0.58262036
C	-0.76288836	2.33928586	-0.16826692
H	-1.80863595	2.59285980	-0.39141524
H	-0.65065874	2.25560891	0.91253063
C	2.94164631	-11.88318495	-4.69332459
H	3.17477379	-12.12024079	-5.74076864
H	1.85838001	-11.84252468	-4.58106808
C	2.71362862	2.49146541	-4.36061863
H	2.93835569	2.78398713	-5.39580543
H	1.63227832	2.41133722	-4.25115781
C	7.13586949	-11.89714345	-1.21576238
H	7.02363987	-11.81346650	-2.29655994
H	8.18161707	-12.15071740	-0.99261406
C	6.90785181	2.47750690	-0.88305642
H	6.79753819	2.44039539	-1.96664966
H	7.94519897	2.75351053	-0.64765084
C	0.38289904	-13.10559593	-1.07955657
O	0.94470667	-13.96840693	-0.41999751
C	3.12316373	-13.13458055	2.05075279
O	3.81600033	-13.96690750	1.48303975
C	3.51264743	-12.99256162	-3.81828357
O	2.84696053	-13.87180103	-3.29018530
C	6.25291213	-13.02154624	-0.68797422
O	5.71825420	-13.87030159	-1.38714803
C	5.99008209	3.54773834	-0.30447273
O	5.42827446	4.41054934	-0.96403178
C	2.86033369	3.43470402	2.43425428
O	3.52602059	4.31394343	1.90615600
C	0.12006900	3.46368864	-0.69605508
O	0.65472693	4.31244400	0.00311873
C	3.24981739	3.57672296	-3.43478208
O	2.55698079	4.40904990	-2.86706905
O	0.49179808	-12.99919474	-2.39768889
H	1.24585844	-13.56151838	-2.73538612

O	1.80191277	-13.07885484	1.94192469
H	1.48658474	-13.64840642	1.18361503
O	4.83154736	-12.90007367	-3.70602502
H	5.16532800	-13.49380824	-2.97464000
O	6.14166205	-12.97973376	0.63358855
H	5.40605431	-13.58069628	0.94436115
O	5.88118305	3.44133715	1.01365959
H	5.12712269	4.00366078	1.35135683
O	1.54143376	3.34221607	2.32199573
H	1.20765313	3.93595065	1.59061071
O	0.23131908	3.42187617	-2.01761785
H	0.96692682	4.02283869	-2.32839045
O	4.57106835	3.52099725	-3.32595399
H	4.88639638	4.09054883	-2.56764433

[P-CH$_2$-P]$_6$

C	0.21842896	4.45383188	4.74193300
O	1.42925016	4.67305747	4.85315500
C	3.96634604	2.03775091	4.74193300
O	4.76161156	1.09876178	4.85315500
C	-3.74791707	2.41608097	4.74193300
O	-3.33236140	3.57429569	4.85315500
C	-0.21842896	-4.45383188	4.74193300
O	-1.42925016	-4.67305747	4.85315500
C	-3.96634604	-2.03775091	4.74193300
O	-4.76161156	-1.09876178	4.85315500
C	3.74791707	-2.41608097	4.74193300
O	3.33236140	-3.57429569	4.85315500
H	-4.74136674	0.80529217	5.53553700
H	-1.67327990	4.50879013	5.53553700
H	3.06808685	3.70349796	5.53553700
H	1.67327990	-4.50879013	5.53553700
H	-3.06808685	-3.70349796	5.53553700
H	4.74136674	-0.80529217	5.53553700
C	-3.44445287	1.55837003	3.51209500
H	-2.38514018	1.26517888	3.59223400
H	-4.04483686	0.65029103	3.52454700

C	-0.37263840	3.76216871	3.51209500
H	-0.09689304	2.69818143	3.59223400
H	-1.45924988	3.82807699	3.52454700
C	3.07181447	2.20379867	3.51209500
H	2.28824714	1.43300254	3.59223400
H	2.58558698	3.17778596	3.52454700
C	0.37263840	-3.76216871	3.51209500
H	0.09689304	-2.69818143	3.59223400
H	1.45924988	-3.82807699	3.52454700
C	-3.07181447	-2.20379867	3.51209500
H	-2.28824714	-1.43300254	3.59223400
H	-2.58558698	-3.17778596	3.52454700
C	3.44445287	-1.55837003	3.51209500
H	2.38514018	-1.26517888	3.59223400
H	4.04483686	-0.65029103	3.52454700
C	-0.80813858	4.61497790	1.27854400
O	-2.02598538	4.48011183	1.42142300
C	3.59261880	3.00735749	1.27854400
O	2.86689797	3.99461073	1.42142300
C	-4.40075739	1.60762040	1.27854400
O	-4.89288335	0.48550111	1.42142300
C	0.80813858	-4.61497790	1.27854400
O	2.02598538	-4.48011183	1.42142300
C	-3.59261880	-3.00735749	1.27854400
O	-2.86689797	-3.99461073	1.42142300
C	4.40075739	-1.60762040	1.27854400
O	4.89288335	-0.48550111	1.42142300
C	-4.59636104	2.42675816	0.00000000
H	-3.96815487	3.32203171	0.00000000
H	-5.64554026	2.75102228	0.00000000
C	-0.19654630	5.19394451	0.00000000
H	0.89288642	5.09753878	0.00000000
H	-0.44031494	6.26469242	0.00000000
C	4.39981474	2.76718635	0.00000000
H	4.86104129	1.77550707	0.00000000
H	5.20522531	3.51367014	0.00000000

C	0.19654630	-5.19394451	0.00000000
H	-0.89288642	-5.09753878	0.00000000
H	0.44031494	-6.26469242	0.00000000
C	-4.39981474	-2.76718635	0.00000000
H	-4.86104129	-1.77550707	0.00000000
H	-5.20522531	-3.51367014	0.00000000
C	4.59636104	-2.42675816	0.00000000
H	3.96815487	-3.32203171	0.00000000
H	5.64554026	-2.75102228	0.00000000
N	0.08849829	4.31177190	2.25429000
N	3.77835315	2.07924419	2.25429000
N	-3.68985486	2.23252772	2.25429000
N	-0.08849829	-4.31177190	2.25429000
N	-3.77835315	-2.07924419	2.25429000
N	3.68985486	-2.23252772	2.25429000
H	1.09180454	4.33482937	2.05832700
H	4.29997462	1.22188422	2.05832700
H	-3.20817009	3.11294515	2.05832700
H	-1.09180454	-4.33482937	2.05832700
H	-4.29997462	-1.22188422	2.05832700
H	3.20817009	-3.11294515	2.05832700
N	-3.75980186	-2.97214025	5.70144200
N	-4.45384988	1.77001380	5.70144200
N	-0.69404803	4.74215404	5.70144200
N	3.75980186	2.97214025	5.70144200
N	0.69404803	-4.74215404	5.70144200
N	4.45384988	-1.77001380	5.70144200
C	-0.31666905	5.25162097	7.01211500
H	-0.79638495	6.22096066	7.19647300
H	0.76485838	5.37495027	7.03070600
C	-4.70637170	2.35156704	7.01211500
H	-5.78570244	2.42079073	7.19647300
H	-4.27241429	3.34986192	7.03070600
C	-4.38970264	-2.90005393	7.01211500
H	-5.03727266	-2.02508835	7.03070600
H	-4.98931749	-3.80016993	7.19647300

C	0.31666905	-5.25162097	7.01211500
H	0.79638495	-6.22096066	7.19647300
H	-0.76485838	-5.37495027	7.03070600
C	4.70637170	-2.35156704	7.01211500
H	4.27241429	-3.34986192	7.03070600
H	5.78570244	-2.42079073	7.19647300
C	4.38970264	2.90005393	7.01211500
H	4.98931749	3.80016993	7.19647300
H	5.03727266	2.02508835	7.03070600
C	-4.08092487	1.47697885	8.09656700
O	-3.20508451	1.83184736	8.87392200
C	-0.76136123	4.27267404	8.09656700
O	-0.01611591	3.69160829	8.87392200
C	-3.31956364	-2.79569519	8.09656700
O	-3.18896860	-1.85976093	8.87392200
C	0.76136123	-4.27267404	8.09656700
O	0.01611591	-3.69160829	8.87392200
C	4.08092487	-1.47697885	8.09656700
O	3.20508451	-1.83184736	8.87392200
C	3.31956364	2.79569519	8.09656700
O	3.18896860	1.85976093	8.87392200
O	-2.52094843	-3.85266072	8.07523900
H	-1.68711569	-3.71312982	8.61011600
O	-4.59697627	0.25687502	8.07523900
H	-4.05922259	-0.39547987	8.61011600
O	-2.07602784	4.10953574	8.07523900
H	-2.37210691	3.31764995	8.61011600
O	2.07602784	-4.10953574	8.07523900
H	2.37210691	-3.31764995	8.61011600
O	4.59697627	-0.25687502	8.07523900
H	4.05922259	0.39547987	8.61011600
O	2.52094843	3.85266072	8.07523900
H	1.68711569	3.71312982	8.61011600
C	0.21842896	4.45383188	-4.74193300
O	1.42925016	4.67305747	-4.85315500
C	3.96634604	2.03775091	-4.74193300

O	4.76161156	1.09876178	-4.85315500
C	3.74791707	-2.41608097	-4.74193300
O	3.33236140	-3.57429569	-4.85315500
C	-3.96634604	-2.03775091	-4.74193300
O	-4.76161156	-1.09876178	-4.85315500
C	-3.74791707	2.41608097	-4.74193300
O	-3.33236140	3.57429569	-4.85315500
C	-0.21842896	-4.45383188	-4.74193300
O	-1.42925016	-4.67305747	-4.85315500
H	-1.67327990	4.50879013	-5.53553700
H	3.06808685	3.70349796	-5.53553700
H	4.74136674	-0.80529217	-5.53553700
H	-3.06808685	-3.70349796	-5.53553700
H	-4.74136674	0.80529217	-5.53553700
H	1.67327990	-4.50879013	-5.53553700
C	-0.37263840	3.76216871	-3.51209500
H	-1.45924988	3.82807699	-3.52454700
H	-0.09689304	2.69818143	-3.59223400
C	3.07181447	2.20379867	-3.51209500
H	2.58558698	3.17778596	-3.52454700
H	2.28824714	1.43300254	-3.59223400
C	3.44445287	-1.55837003	-3.51209500
H	4.04483686	-0.65029103	-3.52454700
H	2.38514018	-1.26517888	-3.59223400
C	-3.07181447	-2.20379867	-3.51209500
H	-2.58558698	-3.17778596	-3.52454700
H	-2.28824714	-1.43300254	-3.59223400
C	-3.44445287	1.55837003	-3.51209500
H	-4.04483686	0.65029103	-3.52454700
H	-2.38514018	1.26517888	-3.59223400
C	0.37263840	-3.76216871	-3.51209500
H	1.45924988	-3.82807699	-3.52454700
H	0.09689304	-2.69818143	-3.59223400
C	-0.80813858	4.61497790	-1.27854400
O	-2.02598538	4.48011183	-1.42142300
C	3.59261880	3.00735749	-1.27854400

O	2.86689797	3.99461073	-1.42142300
C	4.40075739	-1.60762040	-1.27854400
O	4.89288335	-0.48550111	-1.42142300
C	-3.59261880	-3.00735749	-1.27854400
O	-2.86689797	-3.99461073	-1.42142300
C	-4.40075739	1.60762040	-1.27854400
O	-4.89288335	0.48550111	-1.42142300
C	0.80813858	-4.61497790	-1.27854400
O	2.02598538	-4.48011183	-1.42142300
N	0.08849829	4.31177190	-2.25429000
N	3.77835315	2.07924419	-2.25429000
N	3.68985486	-2.23252772	-2.25429000
N	-3.77835315	-2.07924419	-2.25429000
N	-3.68985486	2.23252772	-2.25429000
N	-0.08849829	-4.31177190	-2.25429000
H	4.29997462	1.22188422	-2.05832700
H	3.20817009	-3.11294515	-2.05832700
H	1.09180454	4.33482937	-2.05832700
H	-4.29997462	-1.22188422	-2.05832700
H	-3.20817009	3.11294515	-2.05832700
H	-1.09180454	-4.33482937	-2.05832700
N	-0.69404803	4.74215404	-5.70144200
N	3.75980186	2.97214025	-5.70144200
N	4.45384988	-1.77001380	-5.70144200
N	-3.75980186	-2.97214025	-5.70144200
N	-4.45384988	1.77001380	-5.70144200
N	0.69404803	-4.74215404	-5.70144200
C	4.38970264	2.90005393	-7.01211500
H	4.98931749	3.80016993	-7.19647300
H	5.03727266	2.02508835	-7.03070600
C	-0.31666905	5.25162097	-7.01211500
H	-0.79638495	6.22096066	-7.19647300
H	0.76485838	5.37495027	-7.03070600
C	-4.70637170	2.35156704	-7.01211500
H	-4.27241429	3.34986192	-7.03070600
H	-5.78570244	2.42079073	-7.19647300

C	-4.38970264	-2.90005393	-7.01211500
H	-4.98931749	-3.80016993	-7.19647300
H	-5.03727266	-2.02508835	-7.03070600
C	0.31666905	-5.25162097	-7.01211500
H	-0.76485838	-5.37495027	-7.03070600
H	0.79638495	-6.22096066	-7.19647300
C	4.70637170	-2.35156704	-7.01211500
H	5.78570244	-2.42079073	-7.19647300
H	4.27241429	-3.34986192	-7.03070600
C	0.76136123	-4.27267404	-8.09656700
O	0.01611591	-3.69160829	-8.87392200
C	3.31956364	2.79569519	-8.09656700
O	3.18896860	1.85976093	-8.87392200
C	-0.76136123	4.27267404	-8.09656700
O	-0.01611591	3.69160829	-8.87392200
C	-4.08092487	1.47697885	-8.09656700
O	-3.20508451	1.83184736	-8.87392200
C	-3.31956364	-2.79569519	-8.09656700
O	-3.18896860	-1.85976093	-8.87392200
C	4.08092487	-1.47697885	-8.09656700
O	3.20508451	-1.83184736	-8.87392200
O	2.07602784	-4.10953574	-8.07523900
H	2.37210691	-3.31764995	-8.61011600
O	2.52094843	3.85266072	-8.07523900
H	1.68711569	3.71312982	-8.61011600
O	-2.07602784	4.10953574	-8.07523900
H	-2.37210691	3.31764995	-8.61011600
O	-4.59697627	0.25687502	-8.07523900
H	-4.05922259	-0.39547987	-8.61011600
O	-2.52094843	-3.85266072	-8.07523900
H	-1.68711569	-3.71312982	-8.61011600
O	4.59697627	-0.25687502	-8.07523900
H	4.05922259	0.39547987	-8.61011600

[P-CH_2-P]$_8$

C	1.50794300	5.77081700	4.70735400
O	2.73824100	5.72179700	4.81455900

C	5.14686055	3.01430711	4.70735400
O	5.98215024	2.10969268	4.81455900
C	-3.01430711	5.14686055	4.70735400
O	-2.10969268	5.98215024	4.81455900
C	-5.77081700	1.50794300	4.70735400
O	-5.72179700	2.73824100	4.81455900
H	-4.65727500	4.20221800	5.49294300
H	-0.32177389	6.26460758	5.49294300
H	4.20221800	4.65727500	5.49294300
H	-6.26460758	-0.32177389	5.49294300
C	-3.14269900	4.25471500	3.47107600
H	-2.32174200	3.52289600	3.53330600
H	-4.08545600	3.70993400	3.48835900
C	0.78631405	5.23076160	3.47107600
H	0.84934414	4.13278316	3.53330600
H	-0.26553415	5.51217313	3.48835900
C	4.25471500	3.14269900	3.47107600
H	3.52289600	2.32174200	3.53330600
H	3.70993400	4.08545600	3.48835900
C	-5.23076160	0.78631405	3.47107600
H	-4.13278316	0.84934414	3.53330600
H	-5.51217313	-0.26553415	3.48835900
C	0.54948900	6.25079800	1.27387200
O	-0.66328000	6.41460400	1.42953000
C	4.80852905	4.03143426	1.27387200
O	4.06680020	5.00481977	1.42953000
C	-4.03143426	4.80852905	1.27387200
O	-5.00481977	4.06680020	1.42953000
C	-6.25079800	0.54948900	1.27387200
O	-6.41460400	-0.66328000	1.42953000
C	-3.84112200	5.63678900	0.00000000
H	-2.88000400	6.15829400	0.00000000
H	-4.63932900	6.39026200	0.00000000
C	1.26972831	6.70189514	0.00000000
H	2.31810109	6.39104181	0.00000000
H	1.23809660	7.79909859	0.00000000

C	5.63678900	3.84112200	0.00000000
H	6.15829400	2.88000400	0.00000000
H	6.39026200	4.63932900	0.00000000
C	-6.70189514	1.26972831	0.00000000
H	-6.39104181	2.31810109	0.00000000
H	-7.79909859	1.23809660	0.00000000
N	1.35007900	5.69697100	2.22073300
N	4.98301684	3.07371681	2.22073300
N	-3.07371681	4.98301684	2.22073300
N	-5.69697100	1.35007900	2.22073300
H	2.33912600	5.52534000	2.02624500
H	5.56101724	2.25299353	2.02624500
H	-2.25299353	5.56101724	2.02624500
H	-5.52534000	2.33912600	2.02624500
N	-3.92947400	4.89579900	5.67209700
N	0.68329496	6.24041038	5.67209700
N	4.89579900	3.92947400	5.67209700
N	-6.24041038	0.68329496	5.67209700
C	1.15955600	6.62963600	6.99206500
H	0.90968300	7.67845500	7.19330600
H	2.23977300	6.50150600	7.01892400
C	-3.86793066	5.50779048	6.99206500
H	-4.78624458	6.07273062	7.19330600
H	-3.01350030	6.18101766	7.01892400
C	-6.62963600	1.15955600	6.99206500
H	-7.67845500	0.90968300	7.19330600
H	-6.50150600	2.23977300	7.01892400
C	5.50779048	3.86793066	6.99206500
H	6.07273062	4.78624458	7.19330600
H	6.18101766	3.01350030	7.01892400
C	-3.72312700	4.41280000	8.04836300
O	-2.75274700	4.24495300	8.77356100
C	0.48767246	5.75296915	8.04836300
O	1.05514898	4.94812112	8.77356100
C	-5.75296915	0.48767246	8.04836300
O	-4.94812112	1.05514898	8.77356100

C	4.41280000	3.72312700	8.04836300
O	4.24495300	2.75274700	8.77356100
O	-4.79479000	3.63094400	8.05244800
H	-4.67060500	2.78153300	8.56071600
O	-0.82296340	5.95789365	8.05244800
H	-1.33577562	5.26945731	8.56071600
O	-5.95789365	-0.82296340	8.05244800
H	-5.26945731	-1.33577562	8.56071600
O	3.63094400	4.79479000	8.05244800
H	2.78153300	4.67060500	8.56071600
C	-1.50794300	-5.77081700	4.70735400
O	-2.73824100	-5.72179700	4.81455900
C	-5.14686055	-3.01430711	4.70735400
O	-5.98215024	-2.10969268	4.81455900
C	3.01430711	-5.14686055	4.70735400
O	2.10969268	-5.98215024	4.81455900
C	5.77081700	-1.50794300	4.70735400
O	5.72179700	-2.73824100	4.81455900
H	4.65727500	-4.20221800	5.49294300
H	0.32177389	-6.26460758	5.49294300
H	-4.20221800	-4.65727500	5.49294300
H	6.26460758	0.32177389	5.49294300
C	3.14269900	-4.25471500	3.47107600
H	2.32174200	-3.52289600	3.53330600
H	4.08545600	-3.70993400	3.48835900
C	-0.78631405	-5.23076160	3.47107600
H	-0.84934414	-4.13278316	3.53330600
H	0.26553415	-5.51217313	3.48835900
C	-4.25471500	-3.14269900	3.47107600
H	-3.52289600	-2.32174200	3.53330600
H	-3.70993400	-4.08545600	3.48835900
C	5.23076160	-0.78631405	3.47107600
H	4.13278316	-0.84934414	3.53330600
H	5.51217313	0.26553415	3.48835900
C	-0.54948900	-6.25079800	1.27387200
O	0.66328000	-6.41460400	1.42953000

C	-4.80852905	-4.03143426	1.27387200
O	-4.06680020	-5.00481977	1.42953000
C	4.03143426	-4.80852905	1.27387200
O	5.00481977	-4.06680020	1.42953000
C	6.25079800	-0.54948900	1.27387200
O	6.41460400	0.66328000	1.42953000
C	3.84112200	-5.63678900	0.00000000
H	2.88000400	-6.15829400	0.00000000
H	4.63932900	-6.39026200	0.00000000
C	-1.26972831	-6.70189514	0.00000000
H	-2.31810109	-6.39104181	0.00000000
H	-1.23809660	-7.79909859	0.00000000
C	-5.63678900	-3.84112200	0.00000000
H	-6.15829400	-2.88000400	0.00000000
H	-6.39026200	-4.63932900	0.00000000
C	6.70189514	-1.26972831	0.00000000
H	6.39104181	-2.31810109	0.00000000
H	7.79909859	-1.23809660	0.00000000
N	-1.35007900	-5.69697100	2.22073300
N	-4.98301684	-3.07371681	2.22073300
N	3.07371681	-4.98301684	2.22073300
N	5.69697100	-1.35007900	2.22073300
H	-2.33912600	-5.52534000	2.02624500
H	-5.56101724	-2.25299353	2.02624500
H	2.25299353	-5.56101724	2.02624500
H	5.52534000	-2.33912600	2.02624500
N	3.92947400	-4.89579900	5.67209700
N	-0.68329496	-6.24041038	5.67209700
N	-4.89579900	-3.92947400	5.67209700
N	6.24041038	-0.68329496	5.67209700
C	-1.15955600	-6.62963600	6.99206500
H	-0.90968300	-7.67845500	7.19330600
H	-2.23977300	-6.50150600	7.01892400
C	3.86793066	-5.50779048	6.99206500
H	4.78624458	-6.07273062	7.19330600
H	3.01350030	-6.18101766	7.01892400

C	6.62963600	-1.15955600	6.99206500
H	7.67845500	-0.90968300	7.19330600
H	6.50150600	-2.23977300	7.01892400
C	-5.50779048	-3.86793066	6.99206500
H	-6.07273062	-4.78624458	7.19330600
H	-6.18101766	-3.01350030	7.01892400
C	3.72312700	-4.41280000	8.04836300
O	2.75274700	-4.24495300	8.77356100
C	-0.48767246	-5.75296915	8.04836300
O	-1.05514898	-4.94812112	8.77356100
C	5.75296915	-0.48767246	8.04836300
O	4.94812112	-1.05514898	8.77356100
C	-4.41280000	-3.72312700	8.04836300
O	-4.24495300	-2.75274700	8.77356100
O	4.79479000	-3.63094400	8.05244800
H	4.67060500	-2.78153300	8.56071600
O	0.82296340	-5.95789365	8.05244800
H	1.33577562	-5.26945731	8.56071600
O	5.95789365	0.82296340	8.05244800
H	5.26945731	1.33577562	8.56071600
O	-3.63094400	-4.79479000	8.05244800
H	-2.78153300	-4.67060500	8.56071600
C	5.14686055	3.01430711	-4.70735400
O	5.98215024	2.10969268	-4.81455900
C	5.77081700	-1.50794300	-4.70735400
O	5.72179700	-2.73824100	-4.81455900
C	1.50794300	5.77081700	-4.70735400
O	2.73824100	5.72179700	-4.81455900
C	-3.01430711	5.14686055	-4.70735400
O	-2.10969268	5.98215024	-4.81455900
H	-0.32177389	6.26460758	-5.49294300
H	4.20221800	4.65727500	-5.49294300
H	6.26460758	0.32177389	-5.49294300
H	-4.65727500	4.20221800	-5.49294300
C	0.78631405	5.23076160	-3.47107600
H	0.84934414	4.13278316	-3.53330600

H	-0.26553415	5.51217313	-3.48835900
C	4.25471500	3.14269900	-3.47107600
H	3.52289600	2.32174200	-3.53330600
H	3.70993400	4.08545600	-3.48835900
C	5.23076160	-0.78631405	-3.47107600
H	4.13278316	-0.84934414	-3.53330600
H	5.51217313	0.26553415	-3.48835900
C	-3.14269900	4.25471500	-3.47107600
H	-2.32174200	3.52289600	-3.53330600
H	-4.08545600	3.70993400	-3.48835900
C	4.80852905	4.03143426	-1.27387200
O	4.06680020	5.00481977	-1.42953000
C	6.25079800	-0.54948900	-1.27387200
O	6.41460400	0.66328000	-1.42953000
C	0.54948900	6.25079800	-1.27387200
O	-0.66328000	6.41460400	-1.42953000
C	-4.03143426	4.80852905	-1.27387200
O	-5.00481977	4.06680020	-1.42953000
N	4.98301684	3.07371681	-2.22073300
N	5.69697100	-1.35007900	-2.22073300
N	1.35007900	5.69697100	-2.22073300
N	-3.07371681	4.98301684	-2.22073300
H	5.56101724	2.25299353	-2.02624500
H	5.52534000	-2.33912600	-2.02624500
H	2.33912600	5.52534000	-2.02624500
H	-2.25299353	5.56101724	-2.02624500
N	-3.92947400	4.89579900	-5.67209700
N	0.68329496	6.24041038	-5.67209700
N	4.89579900	3.92947400	-5.67209700
N	6.24041038	-0.68329496	-5.67209700
C	5.50779048	3.86793066	-6.99206500
H	6.07273062	4.78624458	-7.19330600
H	6.18101766	3.01350030	-7.01892400
C	1.15955600	6.62963600	-6.99206500
H	0.90968300	7.67845500	-7.19330600
H	2.23977300	6.50150600	-7.01892400

C	-3.86793066	5.50779048	-6.99206500
H	-3.01350030	6.18101766	-7.01892400
H	-4.78624458	6.07273062	-7.19330600
C	6.62963600	-1.15955600	-6.99206500
H	7.67845500	-0.90968300	-7.19330600
H	6.50150600	-2.23977300	-7.01892400
C	0.48767246	5.75296915	-8.04836300
O	1.05514898	4.94812112	-8.77356100
C	4.41280000	3.72312700	-8.04836300
O	4.24495300	2.75274700	-8.77356100
C	-3.72312700	4.41280000	-8.04836300
O	-2.75274700	4.24495300	-8.77356100
C	5.75296915	-0.48767246	-8.04836300
O	4.94812112	-1.05514898	-8.77356100
O	-4.79479000	3.63094400	-8.05244800
H	-4.67060500	2.78153300	-8.56071600
O	-0.82296340	5.95789365	-8.05244800
H	-1.33577562	5.26945731	-8.56071600
O	3.63094400	4.79479000	-8.05244800
H	2.78153300	4.67060500	-8.56071600
O	5.95789365	0.82296340	-8.05244800
H	5.26945731	1.33577562	-8.56071600
C	-5.14686055	-3.01430711	-4.70735400
O	-5.98215024	-2.10969268	-4.81455900
C	-5.77081700	1.50794300	-4.70735400
O	-5.72179700	2.73824100	-4.81455900
C	-1.50794300	-5.77081700	-4.70735400
O	-2.73824100	-5.72179700	-4.81455900
C	3.01430711	-5.14686055	-4.70735400
O	2.10969268	-5.98215024	-4.81455900
H	0.32177389	-6.26460758	-5.49294300
H	-4.20221800	-4.65727500	-5.49294300
H	-6.26460758	-0.32177389	-5.49294300
H	4.65727500	-4.20221800	-5.49294300
C	-0.78631405	-5.23076160	-3.47107600
H	-0.84934414	-4.13278316	-3.53330600

H	0.26553415	-5.51217313	-3.48835900
C	-4.25471500	-3.14269900	-3.47107600
H	-3.52289600	-2.32174200	-3.53330600
H	-3.70993400	-4.08545600	-3.48835900
C	-5.23076160	0.78631405	-3.47107600
H	-4.13278316	0.84934414	-3.53330600
H	-5.51217313	-0.26553415	-3.48835900
C	3.14269900	-4.25471500	-3.47107600
H	2.32174200	-3.52289600	-3.53330600
H	4.08545600	-3.70993400	-3.48835900
C	-4.80852905	-4.03143426	-1.27387200
O	-4.06680020	-5.00481977	-1.42953000
C	-6.25079800	0.54948900	-1.27387200
O	-6.41460400	-0.66328000	-1.42953000
C	-0.54948900	-6.25079800	-1.27387200
O	0.66328000	-6.41460400	-1.42953000
C	4.03143426	-4.80852905	-1.27387200
O	5.00481977	-4.06680020	-1.42953000
N	-4.98301684	-3.07371681	-2.22073300
N	-5.69697100	1.35007900	-2.22073300
N	-1.35007900	-5.69697100	-2.22073300
N	3.07371681	-4.98301684	-2.22073300
H	-5.56101724	-2.25299353	-2.02624500
H	-5.52534000	2.33912600	-2.02624500
H	-2.33912600	-5.52534000	-2.02624500
H	2.25299353	-5.56101724	-2.02624500
N	3.92947400	-4.89579900	-5.67209700
N	-0.68329496	-6.24041038	-5.67209700
N	-4.89579900	-3.92947400	-5.67209700
N	-6.24041038	0.68329496	-5.67209700
C	-5.50779048	-3.86793066	-6.99206500
H	-6.07273062	-4.78624458	-7.19330600
H	-6.18101766	-3.01350030	-7.01892400
C	-1.15955600	-6.62963600	-6.99206500
H	-0.90968300	-7.67845500	-7.19330600
H	-2.23977300	-6.50150600	-7.01892400

C	3.86793066	-5.50779048	-6.99206500
H	3.01350030	-6.18101766	-7.01892400
H	4.78624458	-6.07273062	-7.19330600
C	-6.62963600	1.15955600	-6.99206500
H	-7.67845500	0.90968300	-7.19330600
H	-6.50150600	2.23977300	-7.01892400
C	-0.48767246	-5.75296915	-8.04836300
O	-1.05514898	-4.94812112	-8.77356100
C	-4.41280000	-3.72312700	-8.04836300
O	-4.24495300	-2.75274700	-8.77356100
C	3.72312700	-4.41280000	-8.04836300
O	2.75274700	-4.24495300	-8.77356100
C	-5.75296915	0.48767246	-8.04836300
O	-4.94812112	1.05514898	-8.77356100
O	4.79479000	-3.63094400	-8.05244800
H	4.67060500	-2.78153300	-8.56071600
O	0.82296340	-5.95789365	-8.05244800
H	1.33577562	-5.26945731	-8.56071600
O	-3.63094400	-4.79479000	-8.05244800
H	-2.78153300	-4.67060500	-8.56071600
O	-5.95789365	-0.82296340	-8.05244800
H	-5.26945731	-1.33577562	-8.56071600

$[P\text{-}CH_2\text{-}P]_{10}$

C	-2.89908339	6.89413122	4.68820600
O	-1.88377713	7.59168998	4.78931900
C	1.70686093	7.28150778	4.68820600
O	2.93827570	7.24906263	4.78931900
C	-6.39767639	3.87343086	4.68820600
O	-5.98629112	5.03454980	4.78931900
C	-5.66084238	-4.88759586	4.68820600
O	-6.63800708	-4.13753974	4.78931900
C	-7.45257446	-0.62678844	4.68820600
O	-7.80224537	0.55438271	4.78931900
H	-7.41427359	2.27846014	5.48160400
H	-4.65902807	6.20131365	5.48160400
H	-0.12419218	7.75547611	5.48160400

H	-4.45808090	-6.34731031	5.48160400
H	-7.33751860	-2.51468770	5.48160400
C	-6.11448310	3.02991592	3.44388800
H	-5.04491766	2.77113722	3.48673900
H	-6.69004334	2.10556429	3.46573800
C	-3.16578085	6.04525646	3.44388800
H	-2.45259053	5.20722530	3.48673900
H	-4.17473912	5.63574611	3.46573800
C	0.99214209	6.75151450	3.44388800
H	1.07654282	5.65433031	3.48673900
H	-0.06482645	7.01326446	3.46573800
C	-4.77110046	-4.87892349	3.44388800
H	-4.19447340	-3.94167332	3.48673900
H	-4.06984772	-5.71195417	3.46573800
C	-6.72766063	-1.14274952	3.44388800
H	-5.71025771	-0.72343109	3.48673900
H	-6.64997840	-2.22887152	3.46573800
C	-3.99607024	6.76540782	1.27092300
O	-5.06999610	6.18055055	1.43337700
C	0.74371821	7.82216105	1.27092300
O	-0.46887655	7.98023936	1.43337700
C	-7.20949567	3.12449875	1.27092300
O	-7.73454947	2.02010149	1.43337700
C	-5.19943158	-5.89111463	1.27092300
O	-4.31133791	-6.73174798	1.43337700
C	-7.66913881	-1.70986265	1.27092300
O	-7.44476783	-2.91195768	1.43337700
C	-7.44041755	3.94739919	0.00000000
H	-6.84511584	4.86457366	0.00000000
H	-8.50208339	4.22500115	0.00000000
C	-3.69920121	7.56688074	0.00000000
H	-2.67849039	7.95898090	0.00000000
H	-4.39493658	8.41549697	0.00000000
C	1.45498426	8.29607103	0.00000000
H	2.51122736	8.01332796	0.00000000
H	1.39092662	9.39155897	0.00000000

C	-6.05341519	-5.85644416	0.00000000
H	-6.74174160	-5.00685609	0.00000000
H	-6.64550313	-6.78036466	0.00000000
C	-8.33964727	-1.17985467	0.00000000
H	-8.39713970	-0.08793538	0.00000000
H	-9.36172332	-1.57930150	0.00000000
N	-3.00816801	6.77474432	2.20194200
N	1.54843576	7.24904008	2.20194200
N	-6.41575384	3.71272649	2.20194200
N	-5.51358969	-4.95444891	2.20194200
N	-7.37273977	-0.76742666	2.20194200
H	-2.12015754	7.24342458	2.00987900
H	2.54233466	7.10625092	2.00987900
H	-5.97282163	4.61385625	2.00987900
H	-6.23374144	-4.25473094	2.00987900
H	-7.54407086	0.22195165	2.00987900
N	-7.61392881	-1.54851783	5.66469200
N	-7.06999375	3.22257783	5.66469200
N	-3.82556137	6.76275829	5.66469200
N	0.88010543	7.71979494	5.66469200
N	-5.24960186	-5.72813231	5.66469200
C	-3.65996236	7.34175628	6.99044600
H	-4.48064792	8.03583991	7.20863100
H	-2.71330434	7.87658196	7.01822900
C	-7.27634781	3.78833370	6.99044600
H	-8.34826850	3.86747229	7.20863100
H	-6.82484804	4.77744838	7.01822900
C	-8.11341572	-1.21210359	6.99044600
H	-8.32953175	-0.14650809	7.01822900
H	-9.02713426	-1.77813830	7.20863100
C	-5.85143458	-5.74955851	6.99044600
H	-6.25794156	-6.74456049	7.20863100
H	-6.65261745	-5.01450346	7.01822900
C	1.35440432	8.09087750	6.99044600
H	1.09842788	9.13478982	7.20863100
H	2.43462939	7.96712894	7.01822900

C	-6.62941806	2.87310469	8.03009300
O	-5.66463067	3.14933260	8.72727800
C	-3.67454331	6.22106469	8.03009300
O	-2.73165122	5.87744996	8.72727800
C	-7.05208044	-1.57228365	8.03009300
O	-6.43391374	-0.78172277	8.72727800
C	-4.78108778	-5.41711307	8.03009300
O	-4.74566044	-4.41418662	8.72727800
C	0.68388209	7.19278942	8.03009300
O	1.24472615	6.36058121	8.72727800
O	-6.86231023	-2.88728209	8.04793400
H	-6.05405020	-3.18006566	8.54778000
O	-7.24882743	1.69770448	8.04793400
H	-6.76702520	0.98575426	8.54778000
O	-4.86653892	5.63422563	8.04793400
H	-4.89522657	4.77504956	8.54778000
O	-3.85462377	-6.36942502	8.04793400
H	-3.02863380	-6.13120859	8.54778000
O	-0.62539796	7.41866409	8.04793400
H	-1.15361777	6.74043823	8.54778000
C	2.89908339	-6.89413122	4.68820600
O	1.88377713	-7.59168998	4.78931900
C	-1.70686093	-7.28150778	4.68820600
O	-2.93827570	-7.24906263	4.78931900
C	6.39767639	-3.87343086	4.68820600
O	5.98629112	-5.03454980	4.78931900
C	5.66084238	4.88759586	4.68820600
O	6.63800708	4.13753974	4.78931900
C	7.45257446	0.62678844	4.68820600
O	7.80224537	-0.55438271	4.78931900
H	7.41427359	-2.27846014	5.48160400
H	4.65902807	-6.20131365	5.48160400
H	0.12419218	-7.75547611	5.48160400
H	4.45808090	6.34731031	5.48160400
H	7.33751860	2.51468770	5.48160400
C	6.11448310	-3.02991592	3.44388800

H	5.04491766	-2.77113722	3.48673900
H	6.69004334	-2.10556429	3.46573800
C	3.16578085	-6.04525646	3.44388800
H	2.45259053	-5.20722530	3.48673900
H	4.17473912	-5.63574611	3.46573800
C	-0.99214209	-6.75151450	3.44388800
H	-1.07654282	-5.65433031	3.48673900
H	0.06482645	-7.01326446	3.46573800
C	4.77110046	4.87892349	3.44388800
H	4.19447340	3.94167332	3.48673900
H	4.06984772	5.71195417	3.46573800
C	6.72766063	1.14274952	3.44388800
H	5.71025771	0.72343109	3.48673900
H	6.64997840	2.22887152	3.46573800
C	3.99607024	-6.76540782	1.27092300
O	5.06999610	-6.18055055	1.43337700
C	-0.74371821	-7.82216105	1.27092300
O	0.46887655	-7.98023936	1.43337700
C	7.20949567	-3.12449875	1.27092300
O	7.73454947	-2.02010149	1.43337700
C	5.19943158	5.89111463	1.27092300
O	4.31133791	6.73174798	1.43337700
C	7.66913881	1.70986265	1.27092300
O	7.44476783	2.91195768	1.43337700
C	7.44041755	-3.94739919	0.00000000
H	6.84511584	-4.86457366	0.00000000
H	8.50208339	-4.22500115	0.00000000
C	3.69920121	-7.56688074	0.00000000
H	2.67849039	-7.95898090	0.00000000
H	4.39493658	-8.41549697	0.00000000
C	-1.45498426	-8.29607103	0.00000000
H	-2.51122736	-8.01332796	0.00000000
H	-1.39092662	-9.39155897	0.00000000
C	6.05341519	5.85644416	0.00000000
H	6.74174160	5.00685609	0.00000000
H	6.64550313	6.78036466	0.00000000

C	8.33964727	1.17985467	0.00000000
H	8.39713970	0.08793538	0.00000000
H	9.36172332	1.57930150	0.00000000
N	3.00816801	-6.77474432	2.20194200
N	-1.54843576	-7.24904008	2.20194200
N	6.41575384	-3.71272649	2.20194200
N	5.51358969	4.95444891	2.20194200
N	7.37273977	0.76742666	2.20194200
H	2.12015754	-7.24342458	2.00987900
H	-2.54233466	-7.10625092	2.00987900
H	5.97282163	-4.61385625	2.00987900
H	6.23374144	4.25473094	2.00987900
H	7.54407086	-0.22195165	2.00987900
N	7.61392881	1.54851783	5.66469200
N	7.06999375	-3.22257783	5.66469200
N	3.82556137	-6.76275829	5.66469200
N	-0.88010543	-7.71979494	5.66469200
N	5.24960186	5.72813231	5.66469200
C	3.65996236	-7.34175628	6.99044600
H	4.48064792	-8.03583991	7.20863100
H	2.71330434	-7.87658196	7.01822900
C	7.27634781	-3.78833370	6.99044600
H	8.34826850	-3.86747229	7.20863100
H	6.82484804	-4.77744838	7.01822900
C	8.11341572	1.21210359	6.99044600
H	8.32953175	0.14650809	7.01822900
H	9.02713426	1.77813830	7.20863100
C	5.85143458	5.74955851	6.99044600
H	6.25794156	6.74456049	7.20863100
H	6.65261745	5.01450346	7.01822900
C	-1.35440432	-8.09087750	6.99044600
H	-1.09842788	-9.13478982	7.20863100
H	-2.43462939	-7.96712894	7.01822900
C	6.62941806	-2.87310469	8.03009300
O	5.66463067	-3.14933260	8.72727800
C	3.67454331	-6.22106469	8.03009300

O	2.73165122	-5.87744996	8.72727800
C	7.05208044	1.57228365	8.03009300
O	6.43391374	0.78172277	8.72727800
C	4.78108778	5.41711307	8.03009300
O	4.74566044	4.41418662	8.72727800
C	-0.68388209	-7.19278942	8.03009300
O	-1.24472615	-6.36058121	8.72727800
O	6.86231023	2.88728209	8.04793400
H	6.05405020	3.18006566	8.54778000
O	7.24882743	-1.69770448	8.04793400
H	6.76702520	-0.98575426	8.54778000
O	4.86653892	-5.63422563	8.04793400
H	4.89522657	-4.77504956	8.54778000
O	3.85462377	6.36942502	8.04793400
H	3.02863380	6.13120859	8.54778000
O	0.62539796	-7.41866409	8.04793400
H	1.15361777	-6.74043823	8.54778000
C	1.70686093	7.28150778	-4.68820600
O	2.93827570	7.24906263	-4.78931900
C	5.66084238	4.88759586	-4.68820600
O	6.63800708	4.13753974	-4.78931900
C	-2.89908339	6.89413122	-4.68820600
O	-1.88377713	7.59168998	-4.78931900
C	-7.45257446	-0.62678844	-4.68820600
O	-7.80224537	0.55438271	-4.78931900
C	-6.39767639	3.87343086	-4.68820600
O	-5.98629112	5.03454980	-4.78931900
H	-4.65902807	6.20131365	-5.48160400
H	-0.12419218	7.75547611	-5.48160400
H	4.45808090	6.34731031	-5.48160400
H	-7.33751860	-2.51468770	-5.48160400
H	-7.41427359	2.27846014	-5.48160400
C	-3.16578085	6.04525646	-3.44388800
H	-2.45259053	5.20722530	-3.48673900
H	-4.17473912	5.63574611	-3.46573800
C	0.99214209	6.75151450	-3.44388800

H	1.07654282	5.65433031	-3.48673900
H	-0.06482645	7.01326446	-3.46573800
C	4.77110046	4.87892349	-3.44388800
H	4.19447340	3.94167332	-3.48673900
H	4.06984772	5.71195417	-3.46573800
C	-6.72766063	-1.14274952	-3.44388800
H	-5.71025771	-0.72343109	-3.48673900
H	-6.64997840	-2.22887152	-3.46573800
C	-6.11448310	3.02991592	-3.44388800
H	-5.04491766	2.77113722	-3.48673900
H	-6.69004334	2.10556429	-3.46573800
C	0.74371821	7.82216105	-1.27092300
O	-0.46887655	7.98023936	-1.43337700
C	5.19943158	5.89111463	-1.27092300
O	4.31133791	6.73174798	-1.43337700
C	-3.99607024	6.76540782	-1.27092300
O	-5.06999610	6.18055055	-1.43337700
C	-7.66913881	-1.70986265	-1.27092300
O	-7.44476783	-2.91195768	-1.43337700
C	-7.20949567	3.12449875	-1.27092300
O	-7.73454947	2.02010149	-1.43337700
N	1.54843576	7.24904008	-2.20194200
N	5.51358969	4.95444891	-2.20194200
N	-3.00816801	6.77474432	-2.20194200
N	-7.37273977	-0.76742666	-2.20194200
N	-6.41575384	3.71272649	-2.20194200
H	2.54233466	7.10625092	-2.00987900
H	6.23374144	4.25473094	-2.00987900
H	-2.12015754	7.24342458	-2.00987900
H	-7.54407086	0.22195165	-2.00987900
H	-5.97282163	4.61385625	-2.00987900
N	-7.06999375	3.22257783	-5.66469200
N	-3.82556137	6.76275829	-5.66469200
N	0.88010543	7.71979494	-5.66469200
N	5.24960186	5.72813231	-5.66469200
N	-7.61392881	-1.54851783	-5.66469200

C	1.35440432	8.09087750	-6.99044600
H	1.09842788	9.13478982	-7.20863100
H	2.43462939	7.96712894	-7.01822900
C	-3.65996236	7.34175628	-6.99044600
H	-4.48064792	8.03583991	-7.20863100
H	-2.71330434	7.87658196	-7.01822900
C	-7.27634781	3.78833370	-6.99044600
H	-6.82484804	4.77744838	-7.01822900
H	-8.34826850	3.86747229	-7.20863100
C	-8.11341572	-1.21210359	-6.99044600
H	-9.02713426	-1.77813830	-7.20863100
H	-8.32953175	-0.14650809	-7.01822900
C	5.85143458	5.74955851	-6.99044600
H	6.25794156	6.74456049	-7.20863100
H	6.65261745	5.01450346	-7.01822900
C	-3.67454331	6.22106469	-8.03009300
O	-2.73165122	5.87744996	-8.72727800
C	0.68388209	7.19278942	-8.03009300
O	1.24472615	6.36058121	-8.72727800
C	-6.62941806	2.87310469	-8.03009300
O	-5.66463067	3.14933260	-8.72727800
C	-7.05208044	-1.57228365	-8.03009300
O	-6.43391374	-0.78172277	-8.72727800
C	4.78108778	5.41711307	-8.03009300
O	4.74566044	4.41418662	-8.72727800
O	-7.24882743	1.69770448	-8.04793400
H	-6.76702520	0.98575426	-8.54778000
O	-4.86653892	5.63422563	-8.04793400
H	-4.89522657	4.77504956	-8.54778000
O	-0.62539796	7.41866409	-8.04793400
H	-1.15361777	6.74043823	-8.54778000
O	-6.86231023	-2.88728209	-8.04793400
H	-6.05405020	-3.18006566	-8.54778000
O	3.85462377	6.36942502	-8.04793400
H	3.02863380	6.13120859	-8.54778000
C	-1.70686093	-7.28150778	-4.68820600

O	-2.93827570	-7.24906263	-4.78931900
C	-5.66084238	-4.88759586	-4.68820600
O	-6.63800708	-4.13753974	-4.78931900
C	2.89908339	-6.89413122	-4.68820600
O	1.88377713	-7.59168998	-4.78931900
C	7.45257446	0.62678844	-4.68820600
O	7.80224537	-0.55438271	-4.78931900
C	6.39767639	-3.87343086	-4.68820600
O	5.98629112	-5.03454980	-4.78931900
H	4.65902807	-6.20131365	-5.48160400
H	0.12419218	-7.75547611	-5.48160400
H	-4.45808090	-6.34731031	-5.48160400
H	7.33751860	2.51468770	-5.48160400
H	7.41427359	-2.27846014	-5.48160400
C	3.16578085	-6.04525646	-3.44388800
H	2.45259053	-5.20722530	-3.48673900
H	4.17473912	-5.63574611	-3.46573800
C	-0.99214209	-6.75151450	-3.44388800
H	-1.07654282	-5.65433031	-3.48673900
H	0.06482645	-7.01326446	-3.46573800
C	-4.77110046	-4.87892349	-3.44388800
H	-4.19447340	-3.94167332	-3.48673900
H	-4.06984772	-5.71195417	-3.46573800
C	6.72766063	1.14274952	-3.44388800
H	5.71025771	0.72343109	-3.48673900
H	6.64997840	2.22887152	-3.46573800
C	6.11448310	-3.02991592	-3.44388800
H	5.04491766	-2.77113722	-3.48673900
H	6.69004334	-2.10556429	-3.46573800
C	-0.74371821	-7.82216105	-1.27092300
O	0.46887655	-7.98023936	-1.43337700
C	-5.19943158	-5.89111463	-1.27092300
O	-4.31133791	-6.73174798	-1.43337700
C	3.99607024	-6.76540782	-1.27092300
O	5.06999610	-6.18055055	-1.43337700
C	7.66913881	1.70986265	-1.27092300

O	7.44476783	2.91195768	-1.43337700
C	7.20949567	-3.12449875	-1.27092300
O	7.73454947	-2.02010149	-1.43337700
N	-1.54843576	-7.24904008	-2.20194200
N	-5.51358969	-4.95444891	-2.20194200
N	3.00816801	-6.77474432	-2.20194200
N	7.37273977	0.76742666	-2.20194200
N	6.41575384	-3.71272649	-2.20194200
H	-2.54233466	-7.10625092	-2.00987900
H	-6.23374144	-4.25473094	-2.00987900
H	2.12015754	-7.24342458	-2.00987900
H	7.54407086	-0.22195165	-2.00987900
H	5.97282163	-4.61385625	-2.00987900
N	7.06999375	-3.22257783	-5.66469200
N	3.82556137	-6.76275829	-5.66469200
N	-0.88010543	-7.71979494	-5.66469200
N	-5.24960186	-5.72813231	-5.66469200
N	7.61392881	1.54851783	-5.66469200
C	-1.35440432	-8.09087750	-6.99044600
H	-1.09842788	-9.13478982	-7.20863100
H	-2.43462939	-7.96712894	-7.01822900
C	3.65996236	-7.34175628	-6.99044600
H	4.48064792	-8.03583991	-7.20863100
H	2.71330434	-7.87658196	-7.01822900
C	7.27634781	-3.78833370	-6.99044600
H	6.82484804	-4.77744838	-7.01822900
H	8.34826850	-3.86747229	-7.20863100
C	8.11341572	1.21210359	-6.99044600
H	9.02713426	1.77813830	-7.20863100
H	8.32953175	0.14650809	-7.01822900
C	-5.85143458	-5.74955851	-6.99044600
H	-6.25794156	-6.74456049	-7.20863100
H	-6.65261745	-5.01450346	-7.01822900
C	3.67454331	-6.22106469	-8.03009300
O	2.73165122	-5.87744996	-8.72727800
C	-0.68388209	-7.19278942	-8.03009300

O	-1.24472615	-6.36058121	-8.72727800
C	6.62941806	-2.87310469	-8.03009300
O	5.66463067	-3.14933260	-8.72727800
C	7.05208044	1.57228365	-8.03009300
O	6.43391374	0.78172277	-8.72727800
C	-4.78108778	-5.41711307	-8.03009300
O	-4.74566044	-4.41418662	-8.72727800
O	7.24882743	-1.69770448	-8.04793400
H	6.76702520	-0.98575426	-8.54778000
O	4.86653892	-5.63422563	-8.04793400
H	4.89522657	-4.77504956	-8.54778000
O	0.62539796	-7.41866409	-8.04793400
H	1.15361777	-6.74043823	-8.54778000
O	6.86231023	2.88728209	-8.04793400
H	6.05405020	3.18006566	-8.54778000
O	-3.85462377	-6.36942502	-8.04793400
H	-3.02863380	-6.13120859	-8.54778000

[P-CH$_2$-P]$_{12}$

C	-4.49180000	-7.79280900	4.67280000
O	-5.65710600	-7.39227500	4.76946800
C	0.00639200	-8.99467100	4.67280000
O	-1.20306000	-9.23045100	4.76946800
H	1.89231000	-9.05548500	5.47588700
H	-2.88895400	-8.78843500	5.47588700
C	0.59358700	-8.34177600	3.42051300
H	0.26785200	-7.29022600	3.44374300
H	1.68226300	-8.36281200	3.44699400
C	-3.65682700	-7.52098300	3.42051300
H	-3.41314600	-6.44744700	3.44374300
H	-2.72452300	-8.08353900	3.44699400
C	-3.75260500	-8.65744700	1.26833200
O	-2.64451200	-9.17297300	1.43534100
C	1.07887200	-9.37387100	1.26833200
O	2.29627200	-9.26628400	1.43534100
N	-4.34252900	-7.85156700	2.18735900
N	0.16504300	-8.97092100	2.18735900

H	-5.25398800	-7.43003300	1.99700700
H	-0.83507000	-9.06159100	1.99700700
N	0.90173200	-9.22489700	5.65944200
N	-3.83152600	-8.43986200	5.65944200
C	-4.39287700	-8.63150400	6.98894400
H	-4.46699700	-9.70090900	7.22123200
H	-5.38317500	-8.18346200	7.01523600
C	0.51140900	-9.67154000	6.98894400
H	0.98192100	-10.63473200	7.22123200
H	-0.57023500	-9.77867300	7.01523600
C	0.97168000	-8.63961900	8.01889300
O	0.24720900	-7.93373100	8.70267700
C	-3.47831000	-7.96797000	8.01889300
O	-3.75277600	-6.99441700	8.70267700
O	2.30135200	-8.59198200	8.04300800
H	2.68208200	-7.82533300	8.54361700
O	-2.30296100	-8.59155100	8.04300800
H	-1.58991500	-8.11797800	8.54361700
C	-4.49167500	-7.79387200	-4.67299800
O	-5.65692900	-7.39329900	-4.76993100
C	0.00703100	-8.99552900	-4.67299800
O	-1.20239500	-9.23124900	-4.76993100
H	1.89307400	-9.05634600	-5.47572300
H	-2.88872300	-8.78956300	-5.47572300
C	0.59412200	-8.34252400	-3.42074600
H	0.26893600	-7.29080400	-3.44442100
H	1.68279000	-8.36409400	-3.44701300
C	-3.65673700	-7.52189900	-3.42074600
H	-3.41249700	-6.44848900	-3.44442100
H	-2.72470800	-8.08491300	-3.44701300
C	-3.75278100	-8.65761100	-1.26825100
O	-2.64472000	-9.17329100	-1.43510800
C	1.07880100	-9.37410200	-1.26825100
O	2.29625100	-9.26666300	-1.43510800
C	0.49436200	-10.00345400	0.00004600
H	-0.59857300	-9.97280200	0.00026800

H	0.81243400	-11.05344200	0.00019800
C	-4.57359700	-8.91042700	0.00004600
H	-5.50478100	-8.33741400	0.00026800
H	-4.82313200	-9.97877900	0.00019800
N	-4.34249700	-7.85175400	-2.18746400
N	0.16516400	-8.97106700	-2.18746400
H	-5.25412800	-7.43040600	-1.99752000
H	-0.83500600	-9.06198400	-1.99752000
N	0.90256300	-9.22590300	-5.65943400
N	-3.83130900	-8.44114800	-5.65943400
C	-4.39233600	-8.63268300	-6.98908700
H	-4.46616700	-9.70206200	-7.22156200
H	-5.38272100	-8.18483700	-7.01550400
C	0.51246700	-9.67229100	-6.98908700
H	0.98321800	-10.63531600	-7.22156200
H	-0.56915500	-9.77963700	-7.01550400
C	0.97262100	-8.63998600	-8.01870800
O	0.24809300	-7.93380300	-8.70211600
C	-3.47767900	-7.96875700	-8.01870800
O	-3.75204700	-6.99492200	-8.70211600
O	2.30231500	-8.59235600	-8.04295000
H	2.68289000	-7.82548100	-8.54330000
O	-2.30231500	-8.59235600	-8.04295000
H	-1.58929000	-8.11851000	-8.54330000
C	4.50287100	-7.78641800	4.67280000
O	3.57334500	-8.59533500	4.76946800
C	7.79280900	-4.49180000	4.67280000
O	7.39227500	-5.65710600	4.76946800
H	8.78843500	-2.88895400	5.47588700
H	6.16653100	-6.89612600	5.47588700
C	7.52098300	-3.65682700	3.42051300
H	6.44744700	-3.41314600	3.44374300
H	8.08353900	-2.72452300	3.44699400
C	4.68494900	-6.92739600	3.42051300
H	3.87708000	-6.17959500	3.44374300
H	5.63828900	-6.40127600	3.44699400

C	5.62126600	-7.57857400	1.26833200
O	6.62177200	-6.87670100	1.43534100
C	8.65744700	-3.75260500	1.26833200
O	9.17297300	-2.64451200	1.43534100
N	4.62839200	-7.68652400	2.18735900
N	7.85156700	-4.34252900	2.18735900
H	3.80760300	-8.26510300	1.99700700
H	7.43003300	-5.25398800	1.99700700
N	8.43986200	-3.83152600	5.65944200
N	5.39337200	-7.53812900	5.65944200
C	5.27866300	-8.12009500	6.98894400
H	6.16773500	-8.71898800	7.22123200
H	4.39549800	-8.75369700	7.01523600
C	8.63150400	-4.39287700	6.98894400
H	9.70090900	-4.46699700	7.22123200
H	8.18346200	-5.38317500	7.01523600
C	7.96797000	-3.47831000	8.01889300
O	6.99441700	-3.75277600	8.70267700
C	5.16130900	-6.99629000	8.01889300
O	4.18095400	-6.74720800	8.70267700
O	8.59155100	-2.30296100	8.04300800
H	8.11797800	-1.58991500	8.54361700
O	6.28902100	-6.29019800	8.04300800
H	6.23541800	-5.43589600	8.54361700
C	4.50385300	-7.78684100	-4.67299800
O	3.57432000	-8.59569300	-4.76993100
C	7.79387200	-4.49167500	-4.67299800
O	7.39329900	-5.65692900	-4.76993100
H	8.78956300	-2.88872300	-5.47572300
H	6.16762400	-6.89648900	-5.47572300
C	7.52189900	-3.65673700	-3.42074600
H	6.44848900	-3.41249700	-3.44442100
H	8.08491300	-2.72470800	-3.44701300
C	4.68578700	-6.92777700	-3.42074600
H	3.87830700	-6.17955400	-3.44442100
H	5.63938600	-6.40212300	-3.44701300

C	5.62132000	-7.57881000	-1.26825100
O	6.62194300	-6.87704000	-1.43510800
C	8.65761100	-3.75278100	-1.26825100
O	9.17329100	-2.64472000	-1.43510800
C	8.91042700	-4.57359700	0.00004600
H	8.33741400	-5.50478100	0.00026800
H	9.97877900	-4.82313200	0.00019800
C	5.42985800	-8.41606500	0.00004600
H	4.46802200	-8.93598700	0.00026800
H	6.23031000	-9.16634400	0.00019800
N	4.62856900	-7.68659000	-2.18746400
N	7.85175400	-4.34249700	-2.18746400
H	3.80785600	-8.26541200	-1.99752000
H	7.43040600	-5.25412800	-1.99752000
N	8.44114800	-3.83130900	-5.65943400
N	5.39459400	-7.53858500	-5.65943400
C	5.27995500	-8.12021600	-6.98908700
H	6.16914900	-8.71884500	-7.22156200
H	4.39691600	-8.75399200	-7.01550400
C	8.63268300	-4.39233600	-6.98908700
H	9.70206200	-4.46616700	-7.22156200
H	8.18483700	-5.38272100	-7.01550400
C	7.96875700	-3.47767900	-8.01870800
O	6.99492200	-3.75204700	-8.70211600
C	5.16230700	-6.99613700	-8.01870800
O	4.18175600	-6.74682900	-8.70211600
O	8.59235600	-2.30231500	-8.04295000
H	8.11851000	-1.58929000	-8.54330000
O	6.29004100	-6.29004100	-8.04295000
H	6.23619100	-5.43562000	-8.54330000
C	8.99467100	0.00639200	4.67280000
O	9.23045100	-1.20306000	4.76946800
C	7.78641800	4.50287100	4.67280000
O	8.59533500	3.57334500	4.76946800
H	6.89612600	6.16653100	5.47588700
H	9.05548500	1.89231000	5.47588700

C	6.92739600	4.68494900	3.42051300
H	6.17959500	3.87708000	3.44374300
H	6.40127600	5.63828900	3.44699400
C	8.34177600	0.59358700	3.42051300
H	7.29022600	0.26785200	3.44374300
H	8.36281200	1.68226300	3.44699400
C	9.37387100	1.07887200	1.26833200
O	9.26628400	2.29627200	1.43534100
C	7.57857400	5.62126600	1.26833200
O	6.87670100	6.62177200	1.43534100
N	8.97092100	0.16504300	2.18735900
N	7.68652400	4.62839200	2.18735900
H	9.06159100	-0.83507000	1.99700700
H	8.26510300	3.80760300	1.99700700
N	7.53812900	5.39337200	5.65944200
N	9.22489700	0.90173200	5.65944200
C	9.67154000	0.51140900	6.98894400
H	10.63473200	0.98192100	7.22123200
H	9.77867300	-0.57023500	7.01523600
C	8.12009500	5.27866300	6.98894400
H	8.71898800	6.16773500	7.22123200
H	8.75369700	4.39549800	7.01523600
C	6.99629000	5.16130900	8.01889300
O	6.74720800	4.18095400	8.70267700
C	8.63961900	0.97168000	8.01889300
O	7.93373100	0.24720900	8.70267700
O	6.29019800	6.28902100	8.04300800
H	5.43589600	6.23541800	8.54361700
O	8.59198200	2.30135200	8.04300800
H	7.82533300	2.68208200	8.54361700
C	8.99552900	0.00703100	-4.67299800
O	9.23124900	-1.20239500	-4.76993100
C	7.78684100	4.50385300	-4.67299800
O	8.59569300	3.57432000	-4.76993100
H	6.89648900	6.16762400	-5.47572300
H	9.05634600	1.89307400	-5.47572300

C	6.92777700	4.68578700	-3.42074600
H	6.17955400	3.87830700	-3.44442100
H	6.40212300	5.63938600	-3.44701300
C	8.34252400	0.59412200	-3.42074600
H	7.29080400	0.26893600	-3.44442100
H	8.36409400	1.68279000	-3.44701300
C	9.37410200	1.07880100	-1.26825100
O	9.26666300	2.29625100	-1.43510800
C	7.57881000	5.62132000	-1.26825100
O	6.87704000	6.62194300	-1.43510800
C	8.41606500	5.42985800	0.00004600
H	8.93598700	4.46802200	0.00026800
H	9.16634400	6.23031000	0.00019800
C	10.00345400	0.49436200	0.00004600
H	9.97280200	-0.59857300	0.00026800
H	11.05344200	0.81243400	0.00019800
N	8.97106700	0.16516400	-2.18746400
N	7.68659000	4.62856900	-2.18746400
H	9.06198400	-0.83500600	-1.99752000
H	8.26541200	3.80785600	-1.99752000
N	7.53858500	5.39459400	-5.65943400
N	9.22590300	0.90256300	-5.65943400
C	9.67229100	0.51246700	-6.98908700
H	10.63531600	0.98321800	-7.22156200
H	9.77963700	-0.56915500	-7.01550400
C	8.12021600	5.27995500	-6.98908700
H	8.71884500	6.16914900	-7.22156200
H	8.75399200	4.39691600	-7.01550400
C	6.99613700	5.16230700	-8.01870800
O	6.74682900	4.18175600	-8.70211600
C	8.63998600	0.97262100	-8.01870800
O	7.93380300	0.24809300	-8.70211600
O	6.29004100	6.29004100	-8.04295000
H	5.43562000	6.23619100	-8.54330000
O	8.59235600	2.30231500	-8.04295000
H	7.82548100	2.68289000	-8.54330000

C	4.49180000	7.79280900	4.67280000
O	5.65710600	7.39227500	4.76946800
C	-0.00639200	8.99467100	4.67280000
O	1.20306000	9.23045100	4.76946800
H	-1.89231000	9.05548500	5.47588700
H	2.88895400	8.78843500	5.47588700
C	-0.59358700	8.34177600	3.42051300
H	-0.26785200	7.29022600	3.44374300
H	-1.68226300	8.36281200	3.44699400
C	3.65682700	7.52098300	3.42051300
H	3.41314600	6.44744700	3.44374300
H	2.72452300	8.08353900	3.44699400
C	3.75260500	8.65744700	1.26833200
O	2.64451200	9.17297300	1.43534100
C	-1.07887200	9.37387100	1.26833200
O	-2.29627200	9.26628400	1.43534100
N	4.34252900	7.85156700	2.18735900
N	-0.16504300	8.97092100	2.18735900
H	5.25398800	7.43003300	1.99700700
H	0.83507000	9.06159100	1.99700700
N	-0.90173200	9.22489700	5.65944200
N	3.83152600	8.43986200	5.65944200
C	4.39287700	8.63150400	6.98894400
H	4.46699700	9.70090900	7.22123200
H	5.38317500	8.18346200	7.01523600
C	-0.51140900	9.67154000	6.98894400
H	-0.98192100	10.63473200	7.22123200
H	0.57023500	9.77867300	7.01523600
C	-0.97168000	8.63961900	8.01889300
O	-0.24720900	7.93373100	8.70267700
C	3.47831000	7.96797000	8.01889300
O	3.75277600	6.99441700	8.70267700
O	-2.30135200	8.59198200	8.04300800
H	-2.68208200	7.82533300	8.54361700
O	2.30296100	8.59155100	8.04300800
H	1.58991500	8.11797800	8.54361700

C	4.49167500	7.79387200	-4.67299800
O	5.65692900	7.39329900	-4.76993100
C	-0.00703100	8.99552900	-4.67299800
O	1.20239500	9.23124900	-4.76993100
H	-1.89307400	9.05634600	-5.47572300
H	2.88872300	8.78956300	-5.47572300
C	-0.59412200	8.34252400	-3.42074600
H	-0.26893600	7.29080400	-3.44442100
H	-1.68279000	8.36409400	-3.44701300
C	3.65673700	7.52189900	-3.42074600
H	3.41249700	6.44848900	-3.44442100
H	2.72470800	8.08491300	-3.44701300
C	3.75278100	8.65761100	-1.26825100
O	2.64472000	9.17329100	-1.43510800
C	-1.07880100	9.37410200	-1.26825100
O	-2.29625100	9.26666300	-1.43510800
C	-0.49436200	10.00345400	0.00004600
H	0.59857300	9.97280200	0.00026800
H	-0.81243400	11.05344200	0.00019800
C	4.57359700	8.91042700	0.00004600
H	5.50478100	8.33741400	0.00026800
H	4.82313200	9.97877900	0.00019800
N	4.34249700	7.85175400	-2.18746400
N	-0.16516400	8.97106700	-2.18746400
H	5.25412800	7.43040600	-1.99752000
H	0.83500600	9.06198400	-1.99752000
N	-0.90256300	9.22590300	-5.65943400
N	3.83130900	8.44114800	-5.65943400
C	4.39233600	8.63268300	-6.98908700
H	4.46616700	9.70206200	-7.22156200
H	5.38272100	8.18483700	-7.01550400
C	-0.51246700	9.67229100	-6.98908700
H	-0.98321800	10.63531600	-7.22156200
H	0.56915500	9.77963700	-7.01550400
C	-0.97262100	8.63998600	-8.01870800
O	-0.24809300	7.93380300	-8.70211600

C	3.47767900	7.96875700	-8.01870800
O	3.75204700	6.99492200	-8.70211600
O	-2.30231500	8.59235600	-8.04295000
H	-2.68289000	7.82548100	-8.54330000
O	2.30231500	8.59235600	-8.04295000
H	1.58929000	8.11851000	-8.54330000
C	-4.50287100	7.78641800	4.67280000
O	-3.57334500	8.59533500	4.76946800
C	-7.79280900	4.49180000	4.67280000
O	-7.39227500	5.65710600	4.76946800
H	-8.78843500	2.88895400	5.47588700
H	-6.16653100	6.89612600	5.47588700
C	-7.52098300	3.65682700	3.42051300
H	-6.44744700	3.41314600	3.44374300
H	-8.08353900	2.72452300	3.44699400
C	-4.68494900	6.92739600	3.42051300
H	-3.87708000	6.17959500	3.44374300
H	-5.63828900	6.40127600	3.44699400
C	-5.62126600	7.57857400	1.26833200
O	-6.62177200	6.87670100	1.43534100
C	-8.65744700	3.75260500	1.26833200
O	-9.17297300	2.64451200	1.43534100
N	-4.62839200	7.68652400	2.18735900
N	-7.85156700	4.34252900	2.18735900
H	-3.80760300	8.26510300	1.99700700
H	-7.43003300	5.25398800	1.99700700
N	-8.43986200	3.83152600	5.65944200
N	-5.39337200	7.53812900	5.65944200
C	-5.27866300	8.12009500	6.98894400
H	-6.16773500	8.71898800	7.22123200
H	-4.39549800	8.75369700	7.01523600
C	-8.63150400	4.39287700	6.98894400
H	-9.70090900	4.46699700	7.22123200
H	-8.18346200	5.38317500	7.01523600
C	-7.96797000	3.47831000	8.01889300
O	-6.99441700	3.75277600	8.70267700

C	-5.16130900	6.99629000	8.01889300
O	-4.18095400	6.74720800	8.70267700
O	-8.59155100	2.30296100	8.04300800
H	-8.11797800	1.58991500	8.54361700
O	-6.28902100	6.29019800	8.04300800
H	-6.23541800	5.43589600	8.54361700
C	-4.50385300	7.78684100	-4.67299800
O	-3.57432000	8.59569300	-4.76993100
C	-7.79387200	4.49167500	-4.67299800
O	-7.39329900	5.65692900	-4.76993100
H	-8.78956300	2.88872300	-5.47572300
H	-6.16762400	6.89648900	-5.47572300
C	-7.52189900	3.65673700	-3.42074600
H	-6.44848900	3.41249700	-3.44442100
H	-8.08491300	2.72470800	-3.44701300
C	-4.68578700	6.92777700	-3.42074600
H	-3.87830700	6.17955400	-3.44442100
H	-5.63938600	6.40212300	-3.44701300
C	-5.62132000	7.57881000	-1.26825100
O	-6.62194300	6.87704000	-1.43510800
C	-8.65761100	3.75278100	-1.26825100
O	-9.17329100	2.64472000	-1.43510800
C	-8.91042700	4.57359700	0.00004600
H	-8.33741400	5.50478100	0.00026800
H	-9.97877900	4.82313200	0.00019800
C	-5.42985800	8.41606500	0.00004600
H	-4.46802200	8.93598700	0.00026800
H	-6.23031000	9.16634400	0.00019800
N	-4.62856900	7.68659000	-2.18746400
N	-7.85175400	4.34249700	-2.18746400
H	-3.80785600	8.26541200	-1.99752000
H	-7.43040600	5.25412800	-1.99752000
N	-8.44114800	3.83130900	-5.65943400
N	-5.39459400	7.53858500	-5.65943400
C	-5.27995500	8.12021600	-6.98908700
H	-6.16914900	8.71884500	-7.22156200

H	-4.39691600	8.75399200	-7.01550400
C	-8.63268300	4.39233600	-6.98908700
H	-9.70206200	4.46616700	-7.22156200
H	-8.18483700	5.38272100	-7.01550400
C	-7.96875700	3.47767900	-8.01870800
O	-6.99492200	3.75204700	-8.70211600
C	-5.16230700	6.99613700	-8.01870800
O	-4.18175600	6.74682900	-8.70211600
O	-8.59235600	2.30231500	-8.04295000
H	-8.11851000	1.58929000	-8.54330000
O	-6.29004100	6.29004100	-8.04295000
H	-6.23619100	5.43562000	-8.54330000
C	-8.99467100	-0.00639200	4.67280000
O	-9.23045100	1.20306000	4.76946800
C	-7.78641800	-4.50287100	4.67280000
O	-8.59533500	-3.57334500	4.76946800
H	-6.89612600	-6.16653100	5.47588700
H	-9.05548500	-1.89231000	5.47588700
C	-6.92739600	-4.68494900	3.42051300
H	-6.17959500	-3.87708000	3.44374300
H	-6.40127600	-5.63828900	3.44699400
C	-8.34177600	-0.59358700	3.42051300
H	-7.29022600	-0.26785200	3.44374300
H	-8.36281200	-1.68226300	3.44699400
C	-9.37387100	-1.07887200	1.26833200
O	-9.26628400	-2.29627200	1.43534100
C	-7.57857400	-5.62126600	1.26833200
O	-6.87670100	-6.62177200	1.43534100
N	-8.97092100	-0.16504300	2.18735900
N	-7.68652400	-4.62839200	2.18735900
H	-9.06159100	0.83507000	1.99700700
H	-8.26510300	-3.80760300	1.99700700
N	-7.53812900	-5.39337200	5.65944200
N	-9.22489700	-0.90173200	5.65944200
C	-9.67154000	-0.51140900	6.98894400
H	-10.63473200	-0.98192100	7.22123200

H	-9.77867300	0.57023500	7.01523600
C	-8.12009500	-5.27866300	6.98894400
H	-8.71898800	-6.16773500	7.22123200
H	-8.75369700	-4.39549800	7.01523600
C	-6.99629000	-5.16130900	8.01889300
O	-6.74720800	-4.18095400	8.70267700
C	-8.63961900	-0.97168000	8.01889300
O	-7.93373100	-0.24720900	8.70267700
O	-6.29019800	-6.28902100	8.04300800
H	-5.43589600	-6.23541800	8.54361700
O	-8.59198200	-2.30135200	8.04300800
H	-7.82533300	-2.68208200	8.54361700
C	-8.99552900	-0.00703100	-4.67299800
O	-9.23124900	1.20239500	-4.76993100
C	-7.78684100	-4.50385300	-4.67299800
O	-8.59569300	-3.57432000	-4.76993100
H	-6.89648900	-6.16762400	-5.47572300
H	-9.05634600	-1.89307400	-5.47572300
C	-6.92777700	-4.68578700	-3.42074600
H	-6.17955400	-3.87830700	-3.44442100
H	-6.40212300	-5.63938600	-3.44701300
C	-8.34252400	-0.59412200	-3.42074600
H	-7.29080400	-0.26893600	-3.44442100
H	-8.36409400	-1.68279000	-3.44701300
C	-9.37410200	-1.07880100	-1.26825100
O	-9.26666300	-2.29625100	-1.43510800
C	-7.57881000	-5.62132000	-1.26825100
O	-6.87704000	-6.62194300	-1.43510800
C	-8.41606500	-5.42985800	0.00004600
H	-8.93598700	-4.46802200	0.00026800
H	-9.16634400	-6.23031000	0.00019800
C	-10.00345400	-0.49436200	0.00004600
H	-9.97280200	0.59857300	0.00026800
H	-11.05344200	-0.81243400	0.00019800
N	-8.97106700	-0.16516400	-2.18746400
N	-7.68659000	-4.62856900	-2.18746400

H	-9.06198400	0.83500600	-1.99752000
H	-8.26541200	-3.80785600	-1.99752000
N	-7.53858500	-5.39459400	-5.65943400
N	-9.22590300	-0.90256300	-5.65943400
C	-9.67229100	-0.51246700	-6.98908700
H	-10.63531600	-0.98321800	-7.22156200
H	-9.77963700	0.56915500	-7.01550400
C	-8.12021600	-5.27995500	-6.98908700
H	-8.71884500	-6.16914900	-7.22156200
H	-8.75399200	-4.39691600	-7.01550400
C	-6.99613700	-5.16230700	-8.01870800
O	-6.74682900	-4.18175600	-8.70211600
C	-8.63998600	-0.97262100	-8.01870800
O	-7.93380300	-0.24809300	-8.70211600
O	-6.29004100	-6.29004100	-8.04295000
H	-5.43562000	-6.23619100	-8.54330000
O	-8.59235600	-2.30231500	-8.04295000
H	-7.82548100	-2.68289000	-8.54330000

[P-CH_2-P]$_{12}$

C	5.79492700	10.54102000	-4.64656595
O	4.83322800	11.31199000	-4.73773095
C	9.38768816	7.52101008	-4.64656595
O	8.79423159	8.60131975	-4.73773095
H	10.62899100	6.10908600	-5.46417495
H	7.48206124	9.71159828	-5.46417495
C	9.29016000	6.66632700	-3.38234195
H	8.27161900	6.24849000	-3.37533495
H	9.99883900	5.83982400	-3.41540195
C	6.03189578	9.71407339	-3.38234195
H	5.25078589	8.93826357	-3.37533495
H	7.00291881	9.22168390	-3.41540195
C	6.98247500	10.45001300	-1.26311895
O	8.01028600	9.79061900	-1.43694495
C	10.45001258	6.98247563	-1.26311895
O	11.14724697	5.97994876	-1.43694495
C	10.58145400	7.83855500	0.00077205

H	9.87688200	8.67439700	0.00038805
H	11.60220300	8.23959800	0.00066105
C	6.77630364	11.29122767	0.00077205
H	5.80550111	11.79381695	0.00038805
H	7.56588024	12.05236682	0.00066105
N	5.96860900	10.49648500	-2.16369495
N	9.53110660	7.41339988	-2.16369495
H	5.13693400	11.06029200	-1.97735095
H	8.97849869	8.25255787	-1.97735095
N	10.11067600	6.97019700	-5.64711595
N	6.67366770	10.30881054	-5.64711595
C	6.51674300	10.87414000	-6.97890995
H	7.37330300	11.51385100	-7.22556795
H	5.60436100	11.46427300	-7.00159895
C	10.18203870	7.55254580	-6.97890995
H	11.21820375	7.81577038	-7.22556795
H	9.56494176	8.44691108	-7.00159895
C	9.67903100	6.53741200	-8.00532695
O	8.67375200	6.64039900	-8.68736595
C	6.44049937	9.74378595	-8.00532695
O	5.47233126	9.45422991	-8.68736595
O	10.50083300	5.48657900	-8.02987495
H	10.17409100	4.71273700	-8.54890795
O	7.60188180	9.08743286	-8.02987495
H	7.59614807	8.24745732	-8.54890795
C	5.79209200	10.53928500	4.64683405
O	4.82916000	11.30869700	4.73842505
C	9.38440501	7.52049205	4.64683405
O	8.78921307	8.59983417	4.73842505
H	10.62791600	6.11008900	5.46315005
H	7.48068424	9.71211354	5.46315005
C	9.28665400	6.66601300	3.38243505
H	8.26760900	6.24951300	3.37494205
H	9.99437000	5.83866300	3.41533305
C	6.02877682	9.71244160	3.38243505
H	5.24668965	8.93767414	3.37494205

H	6.99923429	9.21890106	3.41533305
C	6.98195900	10.45058700	1.26504105
O	8.01055600	9.79277500	1.44017305
C	10.44975552	6.98320340	1.26504105
O	11.14832148	5.98183732	1.44017305
N	5.96651000	10.49512300	2.16386205
N	9.52864616	7.41294480	2.16386205
H	5.13575300	11.06022000	1.97732305
H	8.97738003	8.25294330	1.97732305
N	10.10923500	6.97091700	5.64661505
N	6.67206086	10.30892429	5.64661505
C	6.51497300	10.87336200	6.97877905
H	7.37206900	11.51198000	7.22622005
H	5.60306100	11.46426400	7.00144605
C	10.18010570	7.55250437	6.97877905
H	11.21634768	7.81451403	7.22622005
H	9.56373727	8.44740025	7.00144605
C	9.67567500	6.53649700	8.00373805
O	8.66821300	6.63758700	8.68287405
C	6.43774899	9.74165631	8.00373805
O	5.46829000	9.44951228	8.68287405
O	10.49904500	5.48691100	8.03012705
H	10.17120300	4.71116400	8.54573905
O	7.60010285	9.08705535	8.03012705
H	7.59408186	8.24489887	8.54573905
C	11.55125890	3.35599454	-4.64656595
O	11.41639313	4.58117654	-4.73773095
C	11.95625518	-1.31994073	-4.64656595
O	12.30051231	-0.13640927	-4.73773095
H	11.83560775	-3.19605548	-5.46417495
H	12.15775324	1.57652076	-5.46417495
C	11.28294016	-1.85533011	-3.38234195
H	10.26726754	-1.43056824	-3.37533495
H	11.19962601	-2.94086771	-3.41540195
C	11.13408158	2.60369276	-3.38234195
H	10.03317310	2.60744047	-3.37533495

H	11.47252659	1.56890384	-3.41540195
C	12.32663048	2.45191963	-1.26311895
O	12.58714064	1.25888554	-1.43694495
C	12.32663062	-2.45191890	-1.26311895
O	12.11075625	-3.65383160	-1.43694495
C	13.02491327	-1.93952248	0.00077205
H	13.11773518	-0.85028530	0.00038805
H	14.03027204	-2.37772080	0.00066105
C	12.77567391	3.19253339	0.00077205
H	12.44459714	4.23437874	0.00038805
H	13.87219553	3.17242508	0.00066105
N	11.64257962	3.20169182	-2.16369495
N	11.98157543	-1.49744479	-2.16369495
H	11.45316834	4.18844661	-1.97735095
H	12.18419694	-0.51331768	-1.97735095
N	12.07800113	-2.22065400	-5.64711595
N	12.00842553	2.57043415	-5.64711595
C	12.29721130	3.08114497	-6.97890995
H	13.35523467	2.92780957	-7.22556795
H	12.06934685	4.14358351	-7.00159895
C	12.54024496	-1.85933226	-6.97890995
H	13.45905218	-2.40588371	-7.22556795
H	12.73630328	-0.79056708	-7.00159895
C	11.46675681	-2.22146010	-8.00532695
O	10.82874002	-1.43779769	-8.68736595
C	11.44401790	2.33577634	-8.00532695
O	10.55467262	2.81562754	-8.68736595
O	11.30480744	-3.54561301	-8.02987495
H	10.52657703	-3.86176045	-8.54890795
O	11.80112757	1.05044323	-8.02987495
H	11.20312081	0.46054519	-8.54890795
C	11.54802742	3.35677236	4.64683405
O	11.41118812	4.58172455	4.73842505
C	11.95356735	-1.31798549	4.64683405
O	12.29591322	-0.13391110	4.73842505
H	11.83555684	-3.19458611	5.46315005

H	12.15714390	1.57785880	5.46315005
C	11.28023901	-1.85307302	3.38243505
H	10.26515541	-1.42700937	3.37494205
H	11.19564500	-2.93852860	3.41533305
C	11.13072229	2.60474435	3.38243505
H	10.02985982	2.60992016	3.37494205
H	11.46795348	1.56954143	3.41533305
C	12.32667149	2.45269038	1.26504105
O	12.58885608	1.26021914	1.44017305
C	12.32696347	-2.45122251	1.26504105
O	12.11285145	-3.65325598	1.44017305
N	11.64013232	3.20221296	2.16386205
N	11.97951386	-1.49602678	2.16386205
H	11.45228234	4.18923079	1.97732305
H	12.18367847	-0.51225413	1.97732305
N	12.07749130	-2.21912594	5.64661505
N	12.00736975	2.57165079	5.64661505
C	12.29540959	3.08184642	6.97877905
H	13.35303910	2.92735914	7.22622005
H	12.06842124	4.14449639	7.00144605
C	12.53884883	-1.85799472	6.97877905
H	13.45685137	-2.40545964	7.22622005
H	12.73579748	-0.78936948	7.00144605
C	11.46373676	-2.21973405	8.00373805
O	10.82283497	-1.43586941	8.68287405
C	11.44056720	2.33621527	8.00373805
O	10.54847915	2.81514927	8.68287405
O	11.30377789	-3.54411394	8.03012705
H	10.52342263	-3.86083060	8.54573905
O	11.79960272	1.05143419	8.03012705
H	11.19985068	0.46019712	8.54573905
C	10.54102000	-5.79492700	-4.64656595
O	11.31199000	-4.83322800	-4.73773095
C	7.52101008	-9.38768816	-4.64656595
O	8.60131975	-8.79423159	-4.73773095
H	6.10908600	-10.62899100	-5.46417495

H	9.71159828	-7.48206124	-5.46417495
C	6.66632700	-9.29016000	-3.38234195
H	6.24849000	-8.27161900	-3.37533495
H	5.83982400	-9.99883900	-3.41540195
C	9.71407339	-6.03189578	-3.38234195
H	8.93826357	-5.25078589	-3.37533495
H	9.22168390	-7.00291881	-3.41540195
C	10.45001300	-6.98247500	-1.26311895
O	9.79061900	-8.01028600	-1.43694495
C	6.98247563	-10.45001258	-1.26311895
O	5.97994876	-11.14724697	-1.43694495
C	7.83855500	-10.58145400	0.00077205
H	8.67439700	-9.87688200	0.00038805
H	8.23959800	-11.60220300	0.00066105
C	11.29122767	-6.77630364	0.00077205
H	11.79381695	-5.80550111	0.00038805
H	12.05236682	-7.56588024	0.00066105
N	10.49648500	-5.96860900	-2.16369495
N	7.41339988	-9.53110660	-2.16369495
H	11.06029200	-5.13693400	-1.97735095
H	8.25255787	-8.97849869	-1.97735095
N	6.97019700	-10.11067600	-5.64711595
N	10.30881054	-6.67366770	-5.64711595
C	10.87414000	-6.51674300	-6.97890995
H	11.51385100	-7.37330300	-7.22556795
H	11.46427300	-5.60436100	-7.00159895
C	7.55254580	-10.18203870	-6.97890995
H	7.81577038	-11.21820375	-7.22556795
H	8.44691108	-9.56494176	-7.00159895
C	6.53741200	-9.67903100	-8.00532695
O	6.64039900	-8.67375200	-8.68736595
C	9.74378595	-6.44049937	-8.00532695
O	9.45422991	-5.47233126	-8.68736595
O	5.48657900	-10.50083300	-8.02987495
H	4.71273700	-10.17409100	-8.54890795
O	9.08743286	-7.60188180	-8.02987495

H	8.24745732	-7.59614807	-8.54890795
C	10.53928500	-5.79209200	4.64683405
O	11.30869700	-4.82916000	4.73842505
C	7.52049205	-9.38440501	4.64683405
O	8.59983417	-8.78921307	4.73842505
H	6.11008900	-10.62791600	5.46315005
H	9.71211354	-7.48068424	5.46315005
C	6.66601300	-9.28665400	3.38243505
H	6.24951300	-8.26760900	3.37494205
H	5.83866300	-9.99437000	3.41533305
C	9.71244160	-6.02877682	3.38243505
H	8.93767414	-5.24668965	3.37494205
H	9.21890106	-6.99923429	3.41533305
C	10.45058700	-6.98195900	1.26504105
O	9.79277500	-8.01055600	1.44017305
C	6.98320340	-10.44975552	1.26504105
O	5.98183732	-11.14832148	1.44017305
N	10.49512300	-5.96651000	2.16386205
N	7.41294480	-9.52864616	2.16386205
H	11.06022000	-5.13575300	1.97732305
H	8.25294330	-8.97738003	1.97732305
N	6.97091700	-10.10923500	5.64661505
N	10.30892429	-6.67206086	5.64661505
C	10.87336200	-6.51497300	6.97877905
H	11.51198000	-7.37206900	7.22622005
H	11.46426400	-5.60306100	7.00144605
C	7.55250437	-10.18010570	6.97877905
H	7.81451403	-11.21634768	7.22622005
H	8.44740025	-9.56373727	7.00144605
C	6.53649700	-9.67567500	8.00373805
O	6.63758700	-8.66821300	8.68287405
C	9.74165631	-6.43774899	8.00373805
O	9.44951228	-5.46829000	8.68287405
O	5.48691100	-10.49904500	8.03012705
H	4.71116400	-10.17120300	8.54573905
O	9.08705535	-7.60010285	8.03012705

H	8.24489887	-7.59408186	8.54573905
C	3.35599454	-11.55125890	-4.64656595
O	4.58117654	-11.41639313	-4.73773095
C	-1.31994073	-11.95625518	-4.64656595
O	-0.13640927	-12.30051231	-4.73773095
H	-3.19605548	-11.83560775	-5.46417495
H	1.57652076	-12.15775324	-5.46417495
C	-1.85533011	-11.28294016	-3.38234195
H	-1.43056824	-10.26726754	-3.37533495
H	-2.94086771	-11.19962601	-3.41540195
C	2.60369276	-11.13408158	-3.38234195
H	2.60744047	-10.03317310	-3.37533495
H	1.56890384	-11.47252659	-3.41540195
C	2.45191963	-12.32663048	-1.26311895
O	1.25888554	-12.58714064	-1.43694495
C	-2.45191890	-12.32663062	-1.26311895
O	-3.65383160	-12.11075625	-1.43694495
C	-1.93952248	-13.02491327	0.00077205
H	-0.85028530	-13.11773518	0.00038805
H	-2.37772080	-14.03027204	0.00066105
C	3.19253339	-12.77567391	0.00077205
H	4.23437874	-12.44459714	0.00038805
H	3.17242508	-13.87219553	0.00066105
N	3.20169182	-11.64257962	-2.16369495
N	-1.49744479	-11.98157543	-2.16369495
H	4.18844661	-11.45316834	-1.97735095
H	-0.51331768	-12.18419694	-1.97735095
N	-2.22065400	-12.07800113	-5.64711595
N	2.57043415	-12.00842553	-5.64711595
C	3.08114497	-12.29721130	-6.97890995
H	2.92780957	-13.35523467	-7.22556795
H	4.14358351	-12.06934685	-7.00159895
C	-1.85933226	-12.54024496	-6.97890995
H	-2.40588371	-13.45905218	-7.22556795
H	-0.79056708	-12.73630328	-7.00159895
C	-2.22146010	-11.46675681	-8.00532695

O	-1.43779769	-10.82874002	-8.68736595
C	2.33577634	-11.44401790	-8.00532695
O	2.81562754	-10.55467262	-8.68736595
O	-3.54561301	-11.30480744	-8.02987495
H	-3.86176045	-10.52657703	-8.54890795
O	1.05044323	-11.80112757	-8.02987495
H	0.46054519	-11.20312081	-8.54890795
C	3.35677236	-11.54802742	4.64683405
O	4.58172455	-11.41118812	4.73842505
C	-1.31798549	-11.95356735	4.64683405
O	-0.13391110	-12.29591322	4.73842505
H	-3.19458611	-11.83555684	5.46315005
H	1.57785880	-12.15714390	5.46315005
C	-1.85307302	-11.28023901	3.38243505
H	-1.42700937	-10.26515541	3.37494205
H	-2.93852860	-11.19564500	3.41533305
C	2.60474435	-11.13072229	3.38243505
H	2.60992016	-10.02985982	3.37494205
H	1.56954143	-11.46795348	3.41533305
C	2.45269038	-12.32667149	1.26504105
O	1.26021914	-12.58885608	1.44017305
C	-2.45122251	-12.32696347	1.26504105
O	-3.65325598	-12.11285145	1.44017305
N	3.20221296	-11.64013232	2.16386205
N	-1.49602678	-11.97951386	2.16386205
H	4.18923079	-11.45228234	1.97732305
H	-0.51225413	-12.18367847	1.97732305
N	-2.21912594	-12.07749130	5.64661505
N	2.57165079	-12.00736975	5.64661505
C	3.08184642	-12.29540959	6.97877905
H	2.92735914	-13.35303910	7.22622005
H	4.14449639	-12.06842124	7.00144605
C	-1.85799472	-12.53884883	6.97877905
H	-2.40545964	-13.45685137	7.22622005
H	-0.78936948	-12.73579748	7.00144605
C	-2.21973405	-11.46373676	8.00373805

O	-1.43586941	-10.82283497	8.68287405
C	2.33621527	-11.44056720	8.00373805
O	2.81514927	-10.54847915	8.68287405
O	-3.54411394	-11.30377789	8.03012705
H	-3.86083060	-10.52342263	8.54573905
O	1.05143419	-11.79960272	8.03012705
H	0.46019712	-11.19985068	8.54573905
C	-5.79492700	-10.54102000	-4.64656595
O	-4.83322800	-11.31199000	-4.73773095
C	-9.38768816	-7.52101008	-4.64656595
O	-8.79423159	-8.60131975	-4.73773095
H	-10.62899100	-6.10908600	-5.46417495
H	-7.48206124	-9.71159828	-5.46417495
C	-9.29016000	-6.66632700	-3.38234195
H	-8.27161900	-6.24849000	-3.37533495
H	-9.99883900	-5.83982400	-3.41540195
C	-6.03189578	-9.71407339	-3.38234195
H	-5.25078589	-8.93826357	-3.37533495
H	-7.00291881	-9.22168390	-3.41540195
C	-6.98247500	-10.45001300	-1.26311895
O	-8.01028600	-9.79061900	-1.43694495
C	-10.45001258	-6.98247563	-1.26311895
O	-11.14724697	-5.97994876	-1.43694495
C	-10.58145400	-7.83855500	0.00077205
H	-9.87688200	-8.67439700	0.00038805
H	-11.60220300	-8.23959800	0.00066105
C	-6.77630364	-11.29122767	0.00077205
H	-5.80550111	-11.79381695	0.00038805
H	-7.56588024	-12.05236682	0.00066105
N	-5.96860900	-10.49648500	-2.16369495
N	-9.53110660	-7.41339988	-2.16369495
H	-5.13693400	-11.06029200	-1.97735095
H	-8.97849869	-8.25255787	-1.97735095
N	-10.11067600	-6.97019700	-5.64711595
N	-6.67366770	-10.30881054	-5.64711595
C	-6.51674300	-10.87414000	-6.97890995

H	-7.37330300	-11.51385100	-7.22556795
H	-5.60436100	-11.46427300	-7.00159895
C	-10.18203870	-7.55254580	-6.97890995
H	-11.21820375	-7.81577038	-7.22556795
H	-9.56494176	-8.44691108	-7.00159895
C	-9.67903100	-6.53741200	-8.00532695
O	-8.67375200	-6.64039900	-8.68736595
C	-6.44049937	-9.74378595	-8.00532695
O	-5.47233126	-9.45422991	-8.68736595
O	-10.50083300	-5.48657900	-8.02987495
H	-10.17409100	-4.71273700	-8.54890795
O	-7.60188180	-9.08743286	-8.02987495
H	-7.59614807	-8.24745732	-8.54890795
C	-5.79209200	-10.53928500	4.64683405
O	-4.82916000	-11.30869700	4.73842505
C	-9.38440501	-7.52049205	4.64683405
O	-8.78921307	-8.59983417	4.73842505
H	-10.62791600	-6.11008900	5.46315005
H	-7.48068424	-9.71211354	5.46315005
C	-9.28665400	-6.66601300	3.38243505
H	-8.26760900	-6.24951300	3.37494205
H	-9.99437000	-5.83866300	3.41533305
C	-6.02877682	-9.71244160	3.38243505
H	-5.24668965	-8.93767414	3.37494205
H	-6.99923429	-9.21890106	3.41533305
C	-6.98195900	-10.45058700	1.26504105
O	-8.01055600	-9.79277500	1.44017305
C	-10.44975552	-6.98320340	1.26504105
O	-11.14832148	-5.98183732	1.44017305
N	-5.96651000	-10.49512300	2.16386205
N	-9.52864616	-7.41294480	2.16386205
H	-5.13575300	-11.06022000	1.97732305
H	-8.97738003	-8.25294330	1.97732305
N	-10.10923500	-6.97091700	5.64661505
N	-6.67206086	-10.30892429	5.64661505
C	-6.51497300	-10.87336200	6.97877905

H	-7.37206900	-11.51198000	7.22622005
H	-5.60306100	-11.46426400	7.00144605
C	-10.18010570	-7.55250437	6.97877905
H	-11.21634768	-7.81451403	7.22622005
H	-9.56373727	-8.44740025	7.00144605
C	-9.67567500	-6.53649700	8.00373805
O	-8.66821300	-6.63758700	8.68287405
C	-6.43774899	-9.74165631	8.00373805
O	-5.46829000	-9.44951228	8.68287405
O	-10.49904500	-5.48691100	8.03012705
H	-10.17120300	-4.71116400	8.54573905
O	-7.60010285	-9.08705535	8.03012705
H	-7.59408186	-8.24489887	8.54573905
C	-11.55125890	-3.35599454	-4.64656595
O	-11.41639313	-4.58117654	-4.73773095
C	-11.95625518	1.31994073	-4.64656595
O	-12.30051231	0.13640927	-4.73773095
H	-11.83560775	3.19605548	-5.46417495
H	-12.15775324	-1.57652076	-5.46417495
C	-11.28294016	1.85533011	-3.38234195
H	-10.26726754	1.43056824	-3.37533495
H	-11.19962601	2.94086771	-3.41540195
C	-11.13408158	-2.60369276	-3.38234195
H	-10.03317310	-2.60744047	-3.37533495
H	-11.47252659	-1.56890384	-3.41540195
C	-12.32663048	-2.45191963	-1.26311895
O	-12.58714064	-1.25888554	-1.43694495
C	-12.32663062	2.45191890	-1.26311895
O	-12.11075625	3.65383160	-1.43694495
C	-13.02491327	1.93952248	0.00077205
H	-13.11773518	0.85028530	0.00038805
H	-14.03027204	2.37772080	0.00066105
C	-12.77567391	-3.19253339	0.00077205
H	-12.44459714	-4.23437874	0.00038805
H	-13.87219553	-3.17242508	0.00066105
N	-11.64257962	-3.20169182	-2.16369495

N	-11.98157543	1.49744479	-2.16369495
H	-11.45316834	-4.18844661	-1.97735095
H	-12.18419694	0.51331768	-1.97735095
N	-12.07800113	2.22065400	-5.64711595
N	-12.00842553	-2.57043415	-5.64711595
C	-12.29721130	-3.08114497	-6.97890995
H	-13.35523467	-2.92780957	-7.22556795
H	-12.06934685	-4.14358351	-7.00159895
C	-12.54024496	1.85933226	-6.97890995
H	-13.45905218	2.40588371	-7.22556795
H	-12.73630328	0.79056708	-7.00159895
C	-11.46675681	2.22146010	-8.00532695
O	-10.82874002	1.43779769	-8.68736595
C	-11.44401790	-2.33577634	-8.00532695
O	-10.55467262	-2.81562754	-8.68736595
O	-11.30480744	3.54561301	-8.02987495
H	-10.52657703	3.86176045	-8.54890795
O	-11.80112757	-1.05044323	-8.02987495
H	-11.20312081	-0.46054519	-8.54890795
C	-11.54802742	-3.35677236	4.64683405
O	-11.41118812	-4.58172455	4.73842505
C	-11.95356735	1.31798549	4.64683405
O	-12.29591322	0.13391110	4.73842505
H	-11.83555684	3.19458611	5.46315005
H	-12.15714390	-1.57785880	5.46315005
C	-11.28023901	1.85307302	3.38243505
H	-10.26515541	1.42700937	3.37494205
H	-11.19564500	2.93852860	3.41533305
C	-11.13072229	-2.60474435	3.38243505
H	-10.02985982	-2.60992016	3.37494205
H	-11.46795348	-1.56954143	3.41533305
C	-12.32667149	-2.45269038	1.26504105
O	-12.58885608	-1.26021914	1.44017305
C	-12.32696347	2.45122251	1.26504105
O	-12.11285145	3.65325598	1.44017305
N	-11.64013232	-3.20221296	2.16386205

N	-11.97951386	1.49602678	2.16386205
H	-11.45228234	-4.18923079	1.97732305
H	-12.18367847	0.51225413	1.97732305
N	-12.07749130	2.21912594	5.64661505
N	-12.00736975	-2.57165079	5.64661505
C	-12.29540959	-3.08184642	6.97877905
H	-13.35303910	-2.92735914	7.22622005
H	-12.06842124	-4.14449639	7.00144605
C	-12.53884883	1.85799472	6.97877905
H	-13.45685137	2.40545964	7.22622005
H	-12.73579748	0.78936948	7.00144605
C	-11.46373676	2.21973405	8.00373805
O	-10.82283497	1.43586941	8.68287405
C	-11.44056720	-2.33621527	8.00373805
O	-10.54847915	-2.81514927	8.68287405
O	-11.30377789	3.54411394	8.03012705
H	-10.52342263	3.86083060	8.54573905
O	-11.79960272	-1.05143419	8.03012705
H	-11.19985068	-0.46019712	8.54573905
C	-10.54102000	5.79492700	-4.64656595
O	-11.31199000	4.83322800	-4.73773095
C	-7.52101008	9.38768816	-4.64656595
O	-8.60131975	8.79423159	-4.73773095
H	-6.10908600	10.62899100	-5.46417495
H	-9.71159828	7.48206124	-5.46417495
C	-6.66632700	9.29016000	-3.38234195
H	-6.24849000	8.27161900	-3.37533495
H	-5.83982400	9.99883900	-3.41540195
C	-9.71407339	6.03189578	-3.38234195
H	-8.93826357	5.25078589	-3.37533495
H	-9.22168390	7.00291881	-3.41540195
C	-10.45001300	6.98247500	-1.26311895
O	-9.79061900	8.01028600	-1.43694495
C	-6.98247563	10.45001258	-1.26311895
O	-5.97994876	11.14724697	-1.43694495
C	-7.83855500	10.58145400	0.00077205

H	-8.67439700	9.87688200	0.00038805
H	-8.23959800	11.60220300	0.00066105
C	-11.29122767	6.77630364	0.00077205
H	-11.79381695	5.80550111	0.00038805
H	-12.05236682	7.56588024	0.00066105
N	-10.49648500	5.96860900	-2.16369495
N	-7.41339988	9.53110660	-2.16369495
H	-11.06029200	5.13693400	-1.97735095
H	-8.25255787	8.97849869	-1.97735095
N	-6.97019700	10.11067600	-5.64711595
N	-10.30881054	6.67366770	-5.64711595
C	-10.87414000	6.51674300	-6.97890995
H	-11.51385100	7.37330300	-7.22556795
H	-11.46427300	5.60436100	-7.00159895
C	-7.55254580	10.18203870	-6.97890995
H	-7.81577038	11.21820375	-7.22556795
H	-8.44691108	9.56494176	-7.00159895
C	-6.53741200	9.67903100	-8.00532695
O	-6.64039900	8.67375200	-8.68736595
C	-9.74378595	6.44049937	-8.00532695
O	-9.45422991	5.47233126	-8.68736595
O	-5.48657900	10.50083300	-8.02987495
H	-4.71273700	10.17409100	-8.54890795
O	-9.08743286	7.60188180	-8.02987495
H	-8.24745732	7.59614807	-8.54890795
C	-10.53928500	5.79209200	4.64683405
O	-11.30869700	4.82916000	4.73842505
C	-7.52049205	9.38440501	4.64683405
O	-8.59983417	8.78921307	4.73842505
H	-6.11008900	10.62791600	5.46315005
H	-9.71211354	7.48068424	5.46315005
C	-6.66601300	9.28665400	3.38243505
H	-6.24951300	8.26760900	3.37494205
H	-5.83866300	9.99437000	3.41533305
C	-9.71244160	6.02877682	3.38243505
H	-8.93767414	5.24668965	3.37494205

H	-9.21890106	6.99923429	3.41533305
C	-10.45058700	6.98195900	1.26504105
O	-9.79277500	8.01055600	1.44017305
C	-6.98320340	10.44975552	1.26504105
O	-5.98183732	11.14832148	1.44017305
N	-10.49512300	5.96651000	2.16386205
N	-7.41294480	9.52864616	2.16386205
H	-11.06022000	5.13575300	1.97732305
H	-8.25294330	8.97738003	1.97732305
N	-6.97091700	10.10923500	5.64661505
N	-10.30892429	6.67206086	5.64661505
C	-10.87336200	6.51497300	6.97877905
H	-11.51198000	7.37206900	7.22622005
H	-11.46426400	5.60306100	7.00144605
C	-7.55250437	10.18010570	6.97877905
H	-7.81451403	11.21634768	7.22622005
H	-8.44740025	9.56373727	7.00144605
C	-6.53649700	9.67567500	8.00373805
O	-6.63758700	8.66821300	8.68287405
C	-9.74165631	6.43774899	8.00373805
O	-9.44951228	5.46829000	8.68287405
O	-5.48691100	10.49904500	8.03012705
H	-4.71116400	10.17120300	8.54573905
O	-9.08705535	7.60010285	8.03012705
H	-8.24489887	7.59408186	8.54573905
C	-3.35599454	11.55125890	-4.64656595
O	-4.58117654	11.41639313	-4.73773095
C	1.31994073	11.95625518	-4.64656595
O	0.13640927	12.30051231	-4.73773095
H	3.19605548	11.83560775	-5.46417495
H	-1.57652076	12.15775324	-5.46417495
C	1.85533011	11.28294016	-3.38234195
H	1.43056824	10.26726754	-3.37533495
H	2.94086771	11.19962601	-3.41540195
C	-2.60369276	11.13408158	-3.38234195
H	-2.60744047	10.03317310	-3.37533495

H	-1.56890384	11.47252659	-3.41540195
C	-2.45191963	12.32663048	-1.26311895
O	-1.25888554	12.58714064	-1.43694495
C	2.45191890	12.32663062	-1.26311895
O	3.65383160	12.11075625	-1.43694495
C	1.93952248	13.02491327	0.00077205
H	0.85028530	13.11773518	0.00038805
H	2.37772080	14.03027204	0.00066105
C	-3.19253339	12.77567391	0.00077205
H	-4.23437874	12.44459714	0.00038805
H	-3.17242508	13.87219553	0.00066105
N	-3.20169182	11.64257962	-2.16369495
N	1.49744479	11.98157543	-2.16369495
H	-4.18844661	11.45316834	-1.97735095
H	0.51331768	12.18419694	-1.97735095
N	2.22065400	12.07800113	-5.64711595
N	-2.57043415	12.00842553	-5.64711595
C	-3.08114497	12.29721130	-6.97890995
H	-2.92780957	13.35523467	-7.22556795
H	-4.14358351	12.06934685	-7.00159895
C	1.85933226	12.54024496	-6.97890995
H	2.40588371	13.45905218	-7.22556795
H	0.79056708	12.73630328	-7.00159895
C	2.22146010	11.46675681	-8.00532695
O	1.43779769	10.82874002	-8.68736595
C	-2.33577634	11.44401790	-8.00532695
O	-2.81562754	10.55467262	-8.68736595
O	3.54561301	11.30480744	-8.02987495
H	3.86176045	10.52657703	-8.54890795
O	-1.05044323	11.80112757	-8.02987495
H	-0.46054519	11.20312081	-8.54890795
C	-3.35677236	11.54802742	4.64683405
O	-4.58172455	11.41118812	4.73842505
C	1.31798549	11.95356735	4.64683405
O	0.13391110	12.29591322	4.73842505
H	3.19458611	11.83555684	5.46315005

H	-1.57785880	12.15714390	5.46315005
C	1.85307302	11.28023901	3.38243505
H	1.42700937	10.26515541	3.37494205
H	2.93852860	11.19564500	3.41533305
C	-2.60474435	11.13072229	3.38243505
H	-2.60992016	10.02985982	3.37494205
H	-1.56954143	11.46795348	3.41533305
C	-2.45269038	12.32667149	1.26504105
O	-1.26021914	12.58885608	1.44017305
C	2.45122251	12.32696347	1.26504105
O	3.65325598	12.11285145	1.44017305
N	-3.20221296	11.64013232	2.16386205
N	1.49602678	11.97951386	2.16386205
H	-4.18923079	11.45228234	1.97732305
H	0.51225413	12.18367847	1.97732305
N	2.21912594	12.07749130	5.64661505
N	-2.57165079	12.00736975	5.64661505
C	-3.08184642	12.29540959	6.97877905
H	-2.92735914	13.35303910	7.22622005
H	-4.14449639	12.06842124	7.00144605
C	1.85799472	12.53884883	6.97877905
H	2.40545964	13.45685137	7.22622005
H	0.78936948	12.73579748	7.00144605
C	2.21973405	11.46373676	8.00373805
O	1.43586941	10.82283497	8.68287405
C	-2.33621527	11.44056720	8.00373805
O	-2.81514927	10.54847915	8.68287405
O	3.54411394	11.30377789	8.03012705
H	3.86083060	10.52342263	8.54573905
O	-1.05143419	11.79960272	8.03012705
H	-0.46019712	11.19985068	8.54573905
