# Supplementary material for: Hydrogen-Bond-Driven Peptide Nanotube Formation: A DFT Study
Source: Molecules. 2023 Aug 24;28(17):6217. doi: 10.3390/molecules28176217 (PMC10488343; doi:10.3390/molecules28176217)
Supplement: Supplementary file 1 [file molecules-28-06217-s001.zip › S2.pdf]

Optimized $[P\text{-}(CH_2)_7\text{-}P]_n$ Nanotubes, (*n* = 4, 6, 8, 10, 12, 16)

Partially Folded $[P\text{-}(CH_2)_7\text{-}P]_4$

C	8.56579800	-6.47166900	1.80880800
O	8.42718900	-7.24571000	2.75543000
C	8.65098300	-2.93368200	-1.17443300
O	8.83938600	-4.11910500	-0.89397800
H	9.37984900	-1.11707000	-1.79857200
H	9.72255700	-5.26072800	0.64984300
C	7.27692000	-2.26547800	-1.08832800
H	7.17396800	-1.93077200	-0.04170600
H	7.25946400	-1.37278700	-1.71272400
C	7.38140200	-5.99934500	0.97069700
H	7.23144900	-4.92643900	1.14551900
H	7.59787900	-6.09684700	-0.09643400
C	5.03944200	-6.52490700	0.67053700
O	4.95821300	-5.67645700	-0.22998700
C	5.05732500	-2.50589600	-2.03402200
O	5.02026400	-1.29481300	-2.30299200
C	3.87182200	-3.40884600	-2.34830500
H	4.00421400	-4.39432300	-1.89389600
H	3.85934500	-3.54284400	-3.43946500
C	3.84857800	-7.38722500	1.06988900
H	4.04038000	-7.89318300	2.02442400
H	3.75638900	-8.17577300	0.30929300
N	6.19771600	-6.74341000	1.32502400
N	6.14895500	-3.09145300	-1.47280900
H	6.29484300	-7.39907500	2.09261200
H	6.06207900	-4.03954300	-1.10784200
N	9.63712300	-2.05757200	-1.49378100
N	9.74269100	-5.93545600	1.41102000
C	10.96730600	-6.08439000	2.17016500
H	11.57777400	-6.92310400	1.81132700
H	10.71261400	-6.31114300	3.21125400
C	11.05336400	-2.38196800	-1.40147300

H	11.51038600	-2.42088900	-2.39845000
H	11.15393300	-3.35217900	-0.91975100
C	11.76755400	-1.31204200	-0.57435100
O	12.24986900	-1.47624800	0.53368500
C	11.85654500	-4.84547900	2.13235500
O	12.99071200	-4.83556300	2.55303400
O	11.77260100	-0.14554100	-1.21900000
H	12.10085600	0.60410300	-0.65571900
O	11.23601200	-3.79425700	1.56986200
H	11.85120700	-3.02846700	1.45206800
C	2.54509100	-6.57959100	1.12234300
C	1.28928900	-7.44387000	1.28481200
H	2.47335900	-5.99722700	0.19661600
H	2.60159500	-5.84946500	1.94188900
C	0.00060900	-6.61287100	1.23950300
H	1.26247900	-8.19564200	0.48189300
H	1.33650100	-8.00663300	2.22863000
H	0.00064800	-5.89147300	2.06981700
H	0.00064300	-6.01213500	0.31778300
C	2.54757500	-2.76690100	-1.90020000
C	1.29591300	-3.54161700	-2.33525400
H	2.50434900	-1.74741200	-2.30110400
H	2.55043900	-2.67001600	-0.80516000
H	1.29723600	-3.65427500	-3.42961500
H	1.32561500	-4.56096600	-1.92295400
C	-8.56515800	-6.47275500	1.80735700
O	-8.42690300	-7.24753000	2.75343000
C	-8.64976600	-2.93317700	-1.17457000
O	-8.83787600	-4.11871700	-0.89439700
H	-9.37912000	-1.11665500	-1.79846600
H	-9.72168100	-5.26149100	0.64845700
C	-7.27589200	-2.26465000	-1.08796700
H	-7.17327600	-1.93003500	-0.04128500
H	-7.25844200	-1.37189300	-1.71227100
C	-7.38035100	-5.99912000	0.97057000

H	-7.23092500	-4.92632600	1.14657500
H	-7.59603900	-6.09564100	-0.09680200
C	-5.03825200	-6.52428200	0.67075200
O	-4.95692000	-5.67549000	-0.22943700
C	-5.05604800	-2.50488600	-2.03326800
O	-5.01882400	-1.29375100	-2.30198100
C	-3.87069200	-3.40795200	-2.34778300
H	-4.00315900	-4.39345400	-1.89345200
H	-3.85839800	-3.54184600	-3.43895900
C	-3.84744700	-7.38677100	1.06993100
H	-4.03927500	-7.89281600	2.02441600
H	-3.75537800	-8.17525600	0.30925100
N	-6.19663200	-6.74311200	1.32494600
N	-6.14775300	-3.09046600	-1.47224800
H	-6.29394500	-7.39935400	2.09201700
H	-6.06096600	-4.03862000	-1.10743300
N	-9.63611200	-2.05733900	-1.49400100
N	-9.74200200	-5.93674200	1.40916200
C	-10.96716800	-6.08714000	2.16712700
H	-11.57659300	-6.92618800	1.80729200
H	-10.71323400	-6.31424100	3.20832800
C	-11.05222900	-2.38241300	-1.40232000
H	-11.50888200	-2.42107800	-2.39948800
H	-11.15253400	-3.35291600	-0.92114000
C	-11.76743400	-1.31333700	-0.57500200
O	-12.25154400	-1.47877700	0.53206000
C	-11.85744600	-4.84898600	2.12920600
O	-12.99197900	-4.84027000	2.54891500
O	-11.77137600	-0.14609700	-1.21833600
H	-12.10066800	0.60290800	-0.65484200
O	-11.23735300	-3.79691700	1.56780100
H	-11.85310000	-3.03158700	1.44999400
C	-2.54387400	-6.57929200	1.12243700
C	-1.28816500	-7.44372600	1.28480300
H	-2.47209800	-5.99683400	0.19677200

H	-2.60028100	-5.84924300	1.94205700
H	-1.26146400	-8.19545000	0.48183600
H	-1.33540800	-8.00654400	2.22858700
C	-2.54626900	-2.76628200	-1.89982100
C	-1.29484700	-3.54127200	-2.33508500
H	-2.50288400	-1.74678200	-2.30068400
H	-2.54896600	-2.66945100	-0.80477600
H	-1.29635600	-3.65389000	-3.42945000
H	-1.32474300	-4.56062900	-1.92281900
C	0.00065300	-2.84327700	-1.89477000
H	0.00076400	-1.81597800	-2.28971000
H	0.00073900	-2.74379100	-0.79907900
C	8.35900100	1.49973400	-1.39100700
O	8.60886100	0.58543600	-2.18506900
C	7.81444600	5.08599500	1.52275200
O	8.55447300	4.15961100	1.16662100
H	7.56047500	7.08395000	1.82315400
H	8.88881500	3.27831600	-0.52594800
C	6.35667900	4.86493900	1.93608400
H	6.20571800	3.78969000	2.04074000
H	6.18009100	5.34848900	2.90266700
C	7.10579600	1.46688100	-0.50830300
H	7.21791100	0.62375500	0.18398300
H	7.01192500	2.38153300	0.07577900
C	5.05372300	2.31388400	-1.54258500
O	5.23058400	3.45757400	-1.09574900
C	5.04145700	6.69301100	0.97399500
O	5.58385700	7.52477300	1.71477500
C	3.86631000	7.04222500	0.07351000
H	3.82482300	6.35110700	-0.77771600
H	4.02279300	8.05441900	-0.31301000
C	3.84282300	1.97312100	-2.39345800
H	3.86088100	0.91928300	-2.68884000
H	3.90766800	2.57783500	-3.30778600
N	5.90115100	1.28936100	-1.29275300

N	5.39565200	5.38180100	0.97879500
H	5.70214200	0.36836400	-1.69191600
H	5.10780100	4.75217300	0.22866100
N	8.24679500	6.36411400	1.59116900
N	9.14550400	2.58480800	-1.22878300
C	10.42293300	2.73252900	-1.91632200
H	10.35792700	3.49346900	-2.70202800
H	10.68439500	1.77103200	-2.35608900
C	9.60500900	6.68498000	1.20860400
H	9.72763300	7.77233100	1.24819200
H	9.82747200	6.34696000	0.19320500
C	10.66353800	6.06965900	2.12197100
O	11.74375500	5.64962300	1.74490200
C	11.45620500	3.15555900	-0.87889000
O	12.17908400	2.39687900	-0.25319700
O	10.31705800	6.09678700	3.41531100
H	11.05391300	5.69173600	3.91236200
O	11.41420600	4.47464200	-0.69112800
H	11.87480000	4.74784600	0.14053600
C	2.54232300	2.30815000	-1.64880700
C	1.28444500	2.02593700	-2.47361900
H	2.56819400	3.36388400	-1.35186100
H	2.50170300	1.72373000	-0.71849200
C	0.00021200	2.33971800	-1.70065900
H	1.31158100	2.61383000	-3.40294700
H	1.27714500	0.96960400	-2.77982900
H	0.00009500	1.76923800	-0.75999900
H	0.00032900	3.40132700	-1.41060600
C	2.54619100	6.97283200	0.87186000
C	1.29149200	7.23711800	0.02755800
H	2.59874200	7.69117900	1.70001400
H	2.46484800	5.97675800	1.32836800
H	1.34001600	8.24576500	-0.40728300
H	1.27186000	6.53698700	-0.82082100
C	-8.35854100	1.50035600	-1.39061800

O	-8.60792600	0.58600900	-2.18476800
C	-7.81597700	5.08558700	1.52449800
O	-8.55591300	4.15921600	1.16813900
H	-7.56188800	7.08355100	1.82463400
H	-8.88945200	3.27836700	-0.52506600
C	-6.35820000	4.86457200	1.93784800
H	-6.20734200	3.78935000	2.04300500
H	-6.18143200	5.34855300	2.90418000
C	-7.10515500	1.46818800	-0.50813200
H	-7.21694900	0.62538100	0.18460300
H	-7.01138100	2.38312500	0.07550800
C	-5.05327300	2.31505000	-1.54299300
O	-5.23011600	3.45894300	-1.09663200
C	-5.04297300	6.69210300	0.97483200
O	-5.58512700	7.52416600	1.71546400
C	-3.86803000	7.04090600	0.07393700
H	-3.82656900	6.34922200	-0.77682600
H	-4.02471900	8.05283000	-0.31321200
C	-3.84242300	1.97404200	-2.39383200
H	-3.86066800	0.92021500	-2.68923000
H	-3.90706500	2.57883100	-3.30811900
N	-5.90061900	1.29057000	-1.29271000
N	-5.39730500	5.38093200	0.98015700
H	-5.70153000	0.36943900	-1.69152400
H	-5.10958600	4.75107800	0.23013500
N	-8.24830600	6.36371000	1.59292600
N	-9.14569600	2.58491300	-1.22811000
C	-10.42324700	2.73204200	-1.91556100
H	-10.35858000	3.49290100	-2.70137800
H	-10.68439500	1.77039500	-2.35517700
C	-9.60635300	6.68471100	1.20986100
H	-9.72887600	7.77207600	1.24938900
H	-9.82851100	6.34667200	0.19440100
C	-10.66522200	6.06950900	2.12290900
O	-11.74525500	5.64933500	1.74549300

C	-11.45664000	3.15483600	-0.87815700
O	-12.17938200	2.39602200	-0.25248400
O	-10.31924100	6.09689000	3.41638300
H	-11.05627900	5.69191600	3.91322500
O	-11.41498000	4.47394800	-0.69045000
H	-11.87585500	4.74706100	0.14108300
C	-2.54190700	2.30875300	-1.64904400
C	-1.28402200	2.02625600	-2.47374700
H	-2.56756000	3.36448600	-1.35207600
H	-2.50151400	1.72428600	-0.71874800
H	-1.31093100	2.61417400	-3.40306500
H	-1.27694600	0.96992700	-2.77997400
C	-2.54781100	6.97226400	0.87218900
C	-1.29327500	7.23662700	0.02767100
H	-2.60051800	7.69093100	1.70005500
H	-2.46610600	5.97639900	1.32909300
H	-1.34208500	8.24516200	-0.40740000
H	-1.27357600	6.53630200	-0.82054500
C	-0.00082600	7.08103300	0.84325700
H	-0.00092400	7.80855100	1.66779900
H	-0.00062200	6.08801100	1.31679900

Partially Folded [P-$(CH_2)_7$-P]$_6$

C	4.50303800	7.23106700	8.56825300
O	5.70136900	7.50925900	8.53192100
C	-0.00905100	6.09614400	8.58426800
O	0.89515100	6.93214300	8.67829000
H	-1.64660200	5.16640800	9.40581900
H	2.74327500	7.04501200	9.58866800
C	-0.18535100	5.23173000	7.33226800
H	0.58528600	4.44683000	7.39086700
H	-1.15966400	4.74445500	7.33296800
C	3.74180200	6.78771200	7.32152400
H	3.77308700	5.68732100	7.28285500
H	2.69230200	7.07678400	7.37774200
C	3.62552400	7.57836700	5.01723300

O	2.43315000	7.25381400	4.92561400
C	-0.96497100	5.84067100	5.09294600
O	-1.95889300	5.10472800	5.17297800
C	-0.70085000	6.67627700	3.84707300
H	0.27403700	7.16829000	3.91347900
H	-1.46767200	7.46366200	3.82968200
C	4.35109800	8.24573400	3.85567800
H	5.42640800	8.32609200	4.05782700
H	3.96213800	9.27068200	3.78074300
N	4.34479100	7.36013400	6.13873000
N	-0.05878600	5.97569500	6.09555100
H	5.31041900	7.65404500	6.22950700
H	0.79776400	6.49847900	5.91326500
N	-0.87825300	5.81396700	9.58378500
N	3.73897400	7.23056800	9.68312700
C	4.27820700	7.42265100	11.01294200
H	4.35313600	8.48433400	11.28282600
H	5.29840300	7.02478400	11.04978100
C	-0.76109600	6.40478700	10.91041600
H	-1.59987500	7.08411800	11.10855100
H	0.17312200	6.96020900	10.95533800
C	-0.76636500	5.29457300	11.95793200
O	0.17754200	4.96836400	12.65755200
C	3.44014100	6.74867900	12.09535000
O	3.62822900	6.92057500	13.27810100
O	-1.95434900	4.69141600	11.98698900
H	-1.93896300	3.83682500	12.49461600
O	2.47715300	5.96955600	11.57452300
H	1.86499700	5.62436900	12.27019600
C	4.08924300	7.50524200	2.53626900
C	4.59148700	8.24535000	1.29150200
H	3.00844600	7.34294000	2.45379100
H	4.54819600	6.50754000	2.58315300
C	4.21964000	7.50625000	0.00000000
H	4.15607800	9.25543700	1.27023800

H	5.68148600	8.38225800	1.34415700
H	4.69094100	6.51235900	0.00000000
H	3.13458600	7.32440900	0.00000000
C	-0.80315600	5.84429200	2.55914000
C	-0.65114000	6.68708700	1.28653000
H	-1.77009900	5.32737800	2.54911400
H	-0.03142700	5.06157200	2.57238400
H	-1.41393900	7.47996600	1.28234800
H	0.32142500	7.20139300	1.30001000
C	4.50303800	7.23106700	-8.56825300
O	5.70136900	7.50925900	-8.53192100
C	-0.00905100	6.09614400	-8.58426800
O	0.89515100	6.93214300	-8.67829000
H	-1.64660200	5.16640800	-9.40581900
H	2.74327500	7.04501200	-9.58866800
C	-0.18535100	5.23173000	-7.33226800
H	0.58528600	4.44683000	-7.39086700
H	-1.15966400	4.74445500	-7.33296800
C	3.74180200	6.78771200	-7.32152400
H	3.77308700	5.68732100	-7.28285500
H	2.69230200	7.07678400	-7.37774200
C	3.62552400	7.57836700	-5.01723300
O	2.43315000	7.25381400	-4.92561400
C	-0.96497100	5.84067100	-5.09294600
O	-1.95889300	5.10472800	-5.17297800
C	-0.70085000	6.67627700	-3.84707300
H	0.27403700	7.16829000	-3.91347900
H	-1.46767200	7.46366200	-3.82968200
C	4.35109800	8.24573400	-3.85567800
H	5.42640800	8.32609200	-4.05782700
H	3.96213800	9.27068200	-3.78074300
N	4.34479100	7.36013400	-6.13873000
N	-0.05878600	5.97569500	-6.09555100
H	5.31041900	7.65404500	-6.22950700
H	0.79776400	6.49847900	-5.91326500

N	-0.87825300	5.81396700	-9.58378500
N	3.73897400	7.23056800	-9.68312700
C	4.27820700	7.42265100	-11.01294200
H	4.35313600	8.48433400	-11.28282600
H	5.29840300	7.02478400	-11.04978100
C	-0.76109600	6.40478700	-10.91041600
H	-1.59987500	7.08411800	-11.10855100
H	0.17312200	6.96020900	-10.95533800
C	-0.76636500	5.29457300	-11.95793200
O	0.17754200	4.96836400	-12.65755200
C	3.44014100	6.74867900	-12.09535000
O	3.62822900	6.92057500	-13.27810100
O	-1.95434900	4.69141600	-11.98698900
H	-1.93896300	3.83682500	-12.49461600
O	2.47715300	5.96955600	-11.57452300
H	1.86499700	5.62436900	-12.27019600
C	4.08924300	7.50524200	-2.53626900
C	4.59148700	8.24535000	-1.29150200
H	3.00844600	7.34294000	-2.45379100
H	4.54819600	6.50754000	-2.58315300
H	4.15607800	9.25543700	-1.27023800
H	5.68148600	8.38225800	-1.34415700
C	-0.80315600	5.84429200	-2.55914000
C	-0.65114000	6.68708700	-1.28653000
H	-1.77009900	5.32737800	-2.54911400
H	-0.03142700	5.06157200	-2.57238400
H	-1.41393900	7.47996600	-1.28234800
H	0.32142500	7.20139300	-1.30001000
C	-0.77291600	5.85942600	0.00000000
H	-1.74079000	5.33617100	0.00000000
H	-0.00377700	5.07285500	0.00000000
C	-3.20510800	2.68722100	8.56567500
O	-2.87991600	3.87462200	8.66960700
C	-3.67838800	-1.87783200	8.57207400
O	-4.25285900	-0.79043600	8.68927700

H	-3.14150500	-3.69481100	9.35772400
H	-4.07346800	1.00948500	9.36587400
C	-2.91876100	-2.25311300	7.29946600
H	-1.96383000	-1.70355300	7.33459100
H	-2.69122500	-3.31787500	7.28983000
C	-2.87982900	1.87883500	7.30949700
H	-1.80045800	1.66103500	7.35061100
H	-3.41501500	0.93045800	7.30997000
C	-3.88138300	1.96807900	5.08226400
O	-4.26098500	0.78921300	5.14373400
C	-3.73995800	-2.83970500	5.07796300
O	-3.24334000	-3.97452200	5.13619900
C	-4.51829100	-2.37954600	3.85338300
H	-4.76745400	-1.31692700	3.93599100
H	-5.46354800	-2.94044600	3.85041500
C	-4.16111700	2.82393800	3.85410900
H	-3.65586600	3.79120400	3.93896100
H	-5.24310700	3.01708900	3.84478200
N	-3.21304200	2.58386100	6.08989700
N	-3.64608900	-1.93932500	6.08932800
H	-2.83305300	3.52028100	5.93957100
H	-3.96155500	-0.98009200	5.93227800
N	-3.61071000	-2.80939600	9.55225200
N	-3.82220600	1.98286400	9.54288100
C	-4.07997200	2.53457700	10.86474600
H	-5.15448300	2.50514500	11.08520000
H	-3.73618300	3.56662700	10.88128900
C	-4.11692000	-2.57833900	10.89700200
H	-4.89704700	-3.30982300	11.14253600
H	-4.53483600	-1.57490300	10.94007700
C	-2.97725300	-2.72551600	11.90417200
O	-2.54259900	-1.84229000	12.62751200
C	-3.34537200	1.71326300	11.92327100
O	-2.48035500	2.12977100	12.67816400
O	-2.49071000	-3.96303900	11.87174400

H	-1.64014100	-4.06896600	12.37235000
O	-3.76566100	0.45215100	11.90162200
H	-3.19999500	-0.16890400	12.43257400
C	-3.76431100	2.10701100	2.55397000
C	-4.15964100	2.87861700	1.28863700
H	-4.23274500	1.11566600	2.54435600
H	-2.67800900	1.93831600	2.55266300
C	-3.75990200	2.14741400	0.00000000
H	-5.24651100	3.04918700	1.29033900
H	-3.69472000	3.87538000	1.30357000
H	-2.67193500	1.98461400	0.00000000
H	-4.21561700	1.14582300	0.00000000
C	-3.75478700	-2.66617800	2.55136500
C	-4.55931500	-2.33529200	1.28855300
H	-3.46676700	-3.72405500	2.54062500
H	-2.81951000	-2.08850300	2.54798300
H	-5.49700300	-2.91071100	1.29169700
H	-4.85185500	-1.27505800	1.30385500
C	-3.20510800	2.68722100	-8.56567500
O	-2.87991600	3.87462200	-8.66960700
C	-3.67838800	-1.87783200	-8.57207400
O	-4.25285900	-0.79043600	-8.68927700
H	-3.14150500	-3.69481100	-9.35772400
H	-4.07346800	1.00948500	-9.36587400
C	-2.91876100	-2.25311300	-7.29946600
H	-1.96383000	-1.70355300	-7.33459100
H	-2.69122500	-3.31787500	-7.28983000
C	-2.87982900	1.87883500	-7.30949700
H	-1.80045800	1.66103500	-7.35061100
H	-3.41501500	0.93045800	-7.30997000
C	-3.88138300	1.96807900	-5.08226400
O	-4.26098500	0.78921300	-5.14373400
C	-3.73995800	-2.83970500	-5.07796300
O	-3.24334000	-3.97452200	-5.13619900
C	-4.51829100	-2.37954600	-3.85338300

H	-4.76745400	-1.31692700	-3.93599100
H	-5.46354800	-2.94044600	-3.85041500
C	-4.16111700	2.82393800	-3.85410900
H	-3.65586600	3.79120400	-3.93896100
H	-5.24310700	3.01708900	-3.84478200
N	-3.21304200	2.58386100	-6.08989700
N	-3.64608900	-1.93932500	-6.08932800
H	-2.83305300	3.52028100	-5.93957100
H	-3.96155500	-0.98009200	-5.93227800
N	-3.61071000	-2.80939600	-9.55225200
N	-3.82220600	1.98286400	-9.54288100
C	-4.07997200	2.53457700	-10.86474600
H	-5.15448300	2.50514500	-11.08520000
H	-3.73618300	3.56662700	-10.88128900
C	-4.11692000	-2.57833900	-10.89700200
H	-4.89704700	-3.30982300	-11.14253600
H	-4.53483600	-1.57490300	-10.94007700
C	-2.97725300	-2.72551600	-11.90417200
O	-2.54259900	-1.84229000	-12.62751200
C	-3.34537200	1.71326300	-11.92327100
O	-2.48035500	2.12977100	-12.67816400
O	-2.49071000	-3.96303900	-11.87174400
H	-1.64014100	-4.06896600	-12.37235000
O	-3.76566100	0.45215100	-11.90162200
H	-3.19999500	-0.16890400	-12.43257400
C	-3.76431100	2.10701100	-2.55397000
C	-4.15964100	2.87861700	-1.28863700
H	-4.23274500	1.11566600	-2.54435600
H	-2.67800900	1.93831600	-2.55266300
H	-5.24651100	3.04918700	-1.29033900
H	-3.69472000	3.87538000	-1.30357000
C	-3.75478700	-2.66617800	-2.55136500
C	-4.55931500	-2.33529200	-1.28855300
H	-3.46676700	-3.72405500	-2.54062500
H	-2.81951000	-2.08850300	-2.54798300

H	-5.49700300	-2.91071100	-1.29169700
H	-4.85185500	-1.27505800	-1.30385500
C	-3.78163300	-2.63292100	0.00000000
H	-3.47944300	-3.69097300	0.00000000
H	-2.84774300	-2.05155700	0.00000000
C	-0.88101900	-5.47431600	8.52101700
O	-2.05789600	-5.13104100	8.67183600
C	3.77407500	-6.44049000	8.30051400
O	2.57571400	-6.58452900	8.54021000
H	5.70485800	-6.50003400	8.99608700
H	0.78362300	-6.43328100	9.24146900
C	4.27557800	-6.00443600	6.92319500
H	4.03566600	-4.93465100	6.83271600
H	5.35803200	-6.12471000	6.83054800
C	-0.09996700	-5.11422900	7.25527000
H	0.11379400	-4.03589200	7.31544900
H	0.85042200	-5.64529600	7.21255300
C	-0.30028500	-6.17354900	5.05882900
O	0.84012400	-6.65343800	5.11728300
C	4.45379900	-7.56713500	5.05987300
O	5.65224700	-7.74216700	5.27317300
C	3.74158700	-8.19408500	3.86799600
H	2.66410800	-8.00265400	3.91113500
H	3.89681200	-9.27867400	3.92669100
C	-1.19017000	-6.40690400	3.84811400
H	-2.13043100	-5.85555100	3.94632600
H	-1.43183500	-7.47866000	3.83435100
N	-0.83962200	-5.41901200	6.04934700
N	3.67987300	-6.74899100	5.84170600
H	-1.74486600	-4.97095600	5.89316700
H	2.68021200	-6.65794700	5.65974500
N	4.73412100	-6.61268400	9.25291600
N	-0.17209500	-6.15212700	9.45546400
C	-0.71883400	-6.47839800	10.76546000
H	-0.78333600	-7.56536200	10.89587300

H	-1.71301900	-6.04137800	10.83667200
C	4.40555700	-6.93230000	10.62664300
H	5.05419400	-7.73896100	10.99195900
H	3.37261600	-7.27714400	10.65910300
C	4.55165200	-5.75985500	11.59108100
O	3.79622500	-5.51289600	12.51381800
C	0.19291200	-5.90310300	11.84502800
O	-0.05482700	-4.94438800	12.55855800
O	5.65346500	-5.03422100	11.34765100
H	5.68516600	-4.31860000	12.01228300
O	1.34802000	-6.57037800	11.88655400
H	2.02515400	-6.10189100	12.43370700
C	-0.46766100	-6.03297800	2.54537300
C	-1.26380700	-6.38994300	1.28638500
H	0.50380700	-6.54103900	2.52596100
H	-0.25537900	-4.95437800	2.54860000
C	-0.51674700	-6.02124300	0.00000000
H	-1.48339200	-7.46800300	1.28663000
H	-2.23799900	-5.87958500	1.30614100
H	-0.30766400	-4.94126800	0.00000000
H	0.46446900	-6.51918400	0.00000000
C	4.34099200	-7.66186000	2.55166800
C	3.72654700	-8.28578600	1.29159300
H	5.42300000	-7.83985800	2.56880700
H	4.20573600	-6.57130300	2.51482700
H	3.86865900	-9.37633500	1.31416500
H	2.63886400	-8.11911200	1.29233300
C	-0.88101900	-5.47431600	-8.52101700
O	-2.05789600	-5.13104100	-8.67183600
C	3.77407500	-6.44049000	-8.30051400
O	2.57571400	-6.58452900	-8.54021000
H	5.70485800	-6.50003400	-8.99608700
H	0.78362300	-6.43328100	-9.24146900
C	4.27557800	-6.00443600	-6.92319500
H	4.03566600	-4.93465100	-6.83271600

H	5.35803200	-6.12471000	-6.83054800
C	-0.09996700	-5.11422900	-7.25527000
H	0.11379400	-4.03589200	-7.31544900
H	0.85042200	-5.64529600	-7.21255300
C	-0.30028500	-6.17354900	-5.05882900
O	0.84012400	-6.65343800	-5.11728300
C	4.45379900	-7.56713500	-5.05987300
O	5.65224700	-7.74216700	-5.27317300
C	3.74158700	-8.19408500	-3.86799600
H	2.66410800	-8.00265400	-3.91113500
H	3.89681200	-9.27867400	-3.92669100
C	-1.19017000	-6.40690400	-3.84811400
H	-2.13043100	-5.85555100	-3.94632600
H	-1.43183500	-7.47866000	-3.83435100
N	-0.83962200	-5.41901200	-6.04934700
N	3.67987300	-6.74899100	-5.84170600
H	-1.74486600	-4.97095600	-5.89316700
H	2.68021200	-6.65794700	-5.65974500
N	4.73412100	-6.61268400	-9.25291600
N	-0.17209500	-6.15212700	-9.45546400
C	-0.71883400	-6.47839800	-10.76546000
H	-0.78333600	-7.56536200	-10.89587300
H	-1.71301900	-6.04137800	-10.83667200
C	4.40555700	-6.93230000	-10.62664300
H	5.05419400	-7.73896100	-10.99195900
H	3.37261600	-7.27714400	-10.65910300
C	4.55165200	-5.75985500	-11.59108100
O	3.79622500	-5.51289600	-12.51381800
C	0.19291200	-5.90310300	-11.84502800
O	-0.05482700	-4.94438800	-12.55855800
O	5.65346500	-5.03422100	-11.34765100
H	5.68516600	-4.31860000	-12.01228300
O	1.34802000	-6.57037800	-11.88655400
H	2.02515400	-6.10189100	-12.43370700
C	-0.46766100	-6.03297800	-2.54537300

C	-1.26380700	-6.38994300	-1.28638500
H	0.50380700	-6.54103900	-2.52596100
H	-0.25537900	-4.95437800	-2.54860000
H	-1.48339200	-7.46800300	-1.28663000
H	-2.23799900	-5.87958500	-1.30614100
C	4.34099200	-7.66186000	-2.55166800
C	3.72654700	-8.28578600	-1.29159300
H	5.42300000	-7.83985800	-2.56880700
H	4.20573600	-6.57130300	-2.51482700
H	3.86865900	-9.37633500	-1.31416500
H	2.63886400	-8.11911200	-1.29233300
C	4.32918100	-7.71401300	0.00000000
H	5.41477600	-7.89005200	0.00000000
H	4.20107800	-6.62124400	0.00000000

[P-$(CH_2)_7$-P]$_4$

C	1.98669800	2.43072700	8.68945600
O	3.05843200	1.82558600	8.73187000
C	-2.43072700	1.98669800	8.68945600
O	-1.82558600	3.05843200	8.73187000
C	-1.98669800	-2.43072700	8.68945600
O	-3.05843200	-1.82558600	8.73187000
C	2.43072700	-1.98669800	8.68945600
O	1.82558600	-3.05843200	8.73187000
H	-3.53825800	0.54840800	9.64969600
H	0.54840800	3.53825800	9.64969600
H	-0.54840800	-3.53825800	9.64969600
H	3.53825800	-0.54840800	9.64969600
C	-2.39238800	1.06839300	7.47252000
H	-1.49392500	0.44103200	7.58547900
H	-3.23687200	0.37937200	7.45790000
C	1.06839300	2.39238800	7.47252000
H	0.44103200	1.49392500	7.58547900
H	0.37937200	3.23687200	7.45790000
C	-1.06839300	-2.39238800	7.47252000
H	-0.44103200	-1.49392500	7.58547900

H	-0.37937200	-3.23687200	7.45790000
C	2.39238800	-1.06839300	7.47252000
H	1.49392500	-0.44103200	7.58547900
H	3.23687200	-0.37937200	7.45790000
C	1.20574000	2.87709300	5.09949100
O	0.07167100	3.37117700	5.11733000
C	-2.87709300	1.20574000	5.09949100
O	-3.37117700	0.07167100	5.11733000
C	-1.20574000	-2.87709300	5.09949100
O	-0.07167100	-3.37117700	5.11733000
C	2.87709300	-1.20574000	5.09949100
O	3.37117700	-0.07167100	5.11733000
C	-2.84527300	2.07011300	3.84536300
H	-1.99448400	2.75899000	3.89590900
H	-3.75301800	2.69179200	3.86562600
C	2.07011300	2.84527300	3.84536300
H	2.75899000	1.99448400	3.89590900
H	2.69179200	3.75301800	3.86562600
C	-2.07011300	-2.84527300	3.84536300
H	-2.75899000	-1.99448400	3.89590900
H	-2.69179200	-3.75301800	3.86562600
C	2.84527300	-2.07011300	3.84536300
H	1.99448400	-2.75899000	3.89590900
H	3.75301800	-2.69179200	3.86562600
N	1.79340400	2.37660300	6.22353300
N	-2.37660300	1.79340400	6.22353300
N	-1.79340400	-2.37660300	6.22353300
N	2.37660300	-1.79340400	6.22353300
H	2.59857800	1.76137800	6.13019600
H	-1.76137800	2.59857800	6.13019600
H	-2.59857800	-1.76137800	6.13019600
H	1.76137800	-2.59857800	6.13019600
N	-3.14494300	1.48003800	9.73300900
N	1.48003800	3.14494300	9.73300900
N	-1.48003800	-3.14494300	9.73300900

N	3.14494300	-1.48003800	9.73300900
C	2.12679500	3.18144500	11.03297100
H	2.42352900	4.20801200	11.28993800
H	3.02432700	2.56588200	10.97797500
C	-3.18144500	2.12679500	11.03297100
H	-4.20801200	2.42352900	11.28993800
H	-2.56588200	3.02432700	10.97797500
C	-2.12679500	-3.18144500	11.03297100
H	-2.42352900	-4.20801200	11.28993800
H	-3.02432700	-2.56588200	10.97797500
C	3.18144500	-2.12679500	11.03297100
H	2.56588200	-3.02432700	10.97797500
H	4.20801200	-2.42352900	11.28993800
C	-2.66526800	1.21219900	12.13661000
O	-1.86941600	1.55105600	13.00107900
C	1.21219900	2.66526800	12.13661000
O	1.55105600	1.86941600	13.00107900
C	-1.21219900	-2.66526800	12.13661000
O	-1.55105600	-1.86941600	13.00107900
C	2.66526800	-1.21219900	12.13661000
O	1.86941600	-1.55105600	13.00107900
O	-3.19675100	-0.00081000	12.06040500
H	-2.69949900	-0.63899300	12.64776400
O	-0.00081000	3.19675100	12.06040500
H	-0.63899300	2.69949900	12.64776400
O	0.00081000	-3.19675100	12.06040500
H	0.63899300	-2.69949900	12.64776400
O	3.19675100	0.00081000	12.06040500
H	2.69949900	0.63899300	12.64776400
C	-1.24733100	-2.81401800	2.55381000
H	-0.52367300	-3.63716100	2.57466000
H	-0.65638300	-1.88734000	2.52662200
C	-2.81401800	1.24733100	2.55381000
H	-1.88734000	0.65638300	2.52662200
H	-3.63716100	0.52367300	2.57466000

C	1.24733100	2.81401800	2.55381000
H	0.65638300	1.88734000	2.52662200
H	0.52367300	3.63716100	2.57466000
C	2.81401800	-1.24733100	2.55381000
H	3.63716100	-0.52367300	2.57466000
H	1.88734000	-0.65638300	2.52662200
C	-2.10808200	-2.90321100	1.28764000
H	-2.68670300	-3.83890000	1.30318200
H	-2.84619200	-2.08815400	1.28583000
C	-2.90321100	2.10808200	1.28764000
H	-3.83890000	2.68670300	1.30318200
H	-2.08815400	2.84619200	1.28583000
C	2.10808200	2.90321100	1.28764000
H	2.68670300	3.83890000	1.30318200
H	2.84619200	2.08815400	1.28583000
C	2.90321100	-2.10808200	1.28764000
H	3.83890000	-2.68670300	1.30318200
H	2.08815400	-2.84619200	1.28583000
C	-2.83771400	1.27688300	0.00000000
H	-1.90659800	0.69019300	0.00000000
H	-3.65764100	0.54327000	0.00000000
C	1.98669800	2.43072700	-8.68945600
O	3.05843200	1.82558600	-8.73187000
C	-2.43072700	1.98669800	-8.68945600
O	-1.82558600	3.05843200	-8.73187000
C	-1.98669800	-2.43072700	-8.68945600
O	-3.05843200	-1.82558600	-8.73187000
C	2.43072700	-1.98669800	-8.68945600
O	1.82558600	-3.05843200	-8.73187000
H	-3.53825800	0.54840800	-9.64969600
H	0.54840800	3.53825800	-9.64969600
H	-0.54840800	-3.53825800	-9.64969600
H	3.53825800	-0.54840800	-9.64969600
C	-2.39238800	1.06839300	-7.47252000
H	-1.49392500	0.44103200	-7.58547900

H	-3.23687200	0.37937200	-7.45790000
C	1.06839300	2.39238800	-7.47252000
H	0.44103200	1.49392500	-7.58547900
H	0.37937200	3.23687200	-7.45790000
C	-1.06839300	-2.39238800	-7.47252000
H	-0.44103200	-1.49392500	-7.58547900
H	-0.37937200	-3.23687200	-7.45790000
C	2.39238800	-1.06839300	-7.47252000
H	1.49392500	-0.44103200	-7.58547900
H	3.23687200	-0.37937200	-7.45790000
C	1.20574000	2.87709300	-5.09949100
O	0.07167100	3.37117700	-5.11733000
C	-2.87709300	1.20574000	-5.09949100
O	-3.37117700	0.07167100	-5.11733000
C	-1.20574000	-2.87709300	-5.09949100
O	-0.07167100	-3.37117700	-5.11733000
C	2.87709300	-1.20574000	-5.09949100
O	3.37117700	-0.07167100	-5.11733000
C	-2.84527300	2.07011300	-3.84536300
H	-1.99448400	2.75899000	-3.89590900
H	-3.75301800	2.69179200	-3.86562600
C	2.07011300	2.84527300	-3.84536300
H	2.75899000	1.99448400	-3.89590900
H	2.69179200	3.75301800	-3.86562600
C	-2.07011300	-2.84527300	-3.84536300
H	-2.75899000	-1.99448400	-3.89590900
H	-2.69179200	-3.75301800	-3.86562600
C	2.84527300	-2.07011300	-3.84536300
H	1.99448400	-2.75899000	-3.89590900
H	3.75301800	-2.69179200	-3.86562600
N	1.79340400	2.37660300	-6.22353300
N	-2.37660300	1.79340400	-6.22353300
N	-1.79340400	-2.37660300	-6.22353300
N	2.37660300	-1.79340400	-6.22353300
H	2.59857800	1.76137800	-6.13019600

H	-1.76137800	2.59857800	-6.13019600
H	-2.59857800	-1.76137800	-6.13019600
H	1.76137800	-2.59857800	-6.13019600
N	-3.14494300	1.48003800	-9.73300900
N	1.48003800	3.14494300	-9.73300900
N	-1.48003800	-3.14494300	-9.73300900
N	3.14494300	-1.48003800	-9.73300900
C	2.12679500	3.18144500	-11.03297100
H	2.42352900	4.20801200	-11.28993800
H	3.02432700	2.56588200	-10.97797500
C	-3.18144500	2.12679500	-11.03297100
H	-4.20801200	2.42352900	-11.28993800
H	-2.56588200	3.02432700	-10.97797500
C	-2.12679500	-3.18144500	-11.03297100
H	-2.42352900	-4.20801200	-11.28993800
H	-3.02432700	-2.56588200	-10.97797500
C	3.18144500	-2.12679500	-11.03297100
H	2.56588200	-3.02432700	-10.97797500
H	4.20801200	-2.42352900	-11.28993800
C	-2.66526800	1.21219900	-12.13661000
O	-1.86941600	1.55105600	-13.00107900
C	1.21219900	2.66526800	-12.13661000
O	1.55105600	1.86941600	-13.00107900
C	-1.21219900	-2.66526800	-12.13661000
O	-1.55105600	-1.86941600	-13.00107900
C	2.66526800	-1.21219900	-12.13661000
O	1.86941600	-1.55105600	-13.00107900
O	-3.19675100	-0.00081000	-12.06040500
H	-2.69949900	-0.63899300	-12.64776400
O	-0.00081000	3.19675100	-12.06040500
H	-0.63899300	2.69949900	-12.64776400
O	0.00081000	-3.19675100	-12.06040500
H	0.63899300	-2.69949900	-12.64776400
O	3.19675100	0.00081000	-12.06040500
H	2.69949900	0.63899300	-12.64776400

C	-1.24733100	-2.81401800	-2.55381000
H	-0.52367300	-3.63716100	-2.57466000
H	-0.65638300	-1.88734000	-2.52662200
C	-2.81401800	1.24733100	-2.55381000
H	-1.88734000	0.65638300	-2.52662200
H	-3.63716100	0.52367300	-2.57466000
C	1.24733100	2.81401800	-2.55381000
H	0.65638300	1.88734000	-2.52662200
H	0.52367300	3.63716100	-2.57466000
C	2.81401800	-1.24733100	-2.55381000
H	3.63716100	-0.52367300	-2.57466000
H	1.88734000	-0.65638300	-2.52662200
C	-2.10808200	-2.90321100	-1.28764000
H	-2.68670300	-3.83890000	-1.30318200
H	-2.84619200	-2.08815400	-1.28583000
C	-2.90321100	2.10808200	-1.28764000
H	-3.83890000	2.68670300	-1.30318200
H	-2.08815400	2.84619200	-1.28583000
C	2.10808200	2.90321100	-1.28764000
H	2.68670300	3.83890000	-1.30318200
H	2.84619200	2.08815400	-1.28583000
C	2.90321100	-2.10808200	-1.28764000
H	3.83890000	-2.68670300	-1.30318200
H	2.08815400	-2.84619200	-1.28583000
C	2.83771400	-1.27688300	0.00000000
C	-1.27688300	-2.83771400	0.00000000
C	1.27688300	2.83771400	0.00000000
H	0.69019300	1.90659800	0.00000000
H	0.54327000	3.65764100	0.00000000
H	-0.69019300	-1.90659800	0.00000000
H	-0.54327000	-3.65764100	0.00000000
H	3.65764100	-0.54327000	0.00000000
H	1.90659800	-0.69019300	0.00000000

[P-$(CH_2)_7$-P]$_6$

C	2.43234200	3.74223000	8.60348100

O	3.58626000	3.31393800	8.71474200
C	4.45703700	-0.23535500	8.60348100
O	4.66308500	-1.44882300	8.71474200
C	2.02469500	-3.97758500	8.60348100
O	1.07682500	-4.76276100	8.71474200
C	-4.45703700	0.23535500	8.60348100
O	-4.66308500	1.44882300	8.71474200
C	-2.02469500	3.97758500	8.60348100
O	-1.07682500	4.76276100	8.71474200
C	-2.43234200	-3.74223000	8.60348100
O	-3.58626000	-3.31393800	8.71474200
H	0.82584600	4.73614800	9.40414000
H	4.51454800	1.65287000	9.40414000
H	3.68870100	-3.08327800	9.40414000
H	-4.51454800	-1.65287000	9.40414000
H	-3.68870100	3.08327800	9.40414000
H	-0.82584600	-4.73614800	9.40414000
C	1.58473400	3.47040900	7.36006700
H	0.70099100	4.10625300	7.35256600
H	1.24351200	2.42459200	7.43638800
C	3.79782900	0.36278500	7.36006700
H	3.90661500	1.44605100	7.35256600
H	2.72151400	0.13538300	7.43638800
C	2.21309600	-3.10762400	7.36006700
H	3.20562400	-2.66020200	7.35256600
H	1.47800200	-2.28920900	7.43638800
C	-3.79782900	-0.36278500	7.36006700
H	-3.90661500	-1.44605100	7.35256600
H	-2.72151400	-0.13538300	7.43638800
C	-2.21309600	3.10762400	7.36006700
H	-3.20562400	2.66020200	7.35256600
H	-1.47800200	2.28920900	7.43638800
C	-1.58473400	-3.47040900	7.36006700
H	-0.70099100	-4.10625300	7.35256600
H	-1.24351200	-2.42459200	7.43638800

C	1.72617800	4.35562800	5.09058400
O	0.58394900	4.83228600	5.15087100
C	4.63517300	0.68290000	5.09058400
O	4.47685700	1.91042800	5.15087100
C	2.90899500	-3.67272800	5.09058400
O	3.89290700	-2.92185800	5.15087100
C	-4.63517300	-0.68290000	5.09058400
O	-4.47685700	-1.91042800	5.15087100
C	-2.90899500	3.67272800	5.09058400
O	-3.89290700	2.92185800	5.15087100
C	-1.72617800	-4.35562800	5.09058400
O	-0.58394900	-4.83228600	5.15087100
C	2.59338700	4.51379400	3.84880600
H	3.45478500	3.83976600	3.90111400
H	2.98590500	5.54084500	3.86532600
C	5.20575400	0.01095800	3.84880600
H	5.05272700	-1.07204800	3.90111400
H	6.29146400	0.18455300	3.86532600
C	2.61236700	-4.50283600	3.84880600
H	1.59794200	-4.91181400	3.90111400
H	3.30556000	-5.35629100	3.86532600
C	-5.20575400	-0.01095800	3.84880600
H	-5.05272700	1.07204800	3.90111400
H	-6.29146400	-0.18455300	3.86532600
C	-2.61236700	4.50283600	3.84880600
H	-1.59794200	4.91181400	3.90111400
H	-3.30556000	5.35629100	3.86532600
C	-2.59338700	-4.51379400	3.84880600
H	-3.45478500	-3.83976600	3.90111400
H	-2.98590500	-5.54084500	3.86532600
N	2.30364600	3.68809200	6.12421100
N	4.34580500	-0.15097000	6.12421100
N	2.04215800	-3.83906200	6.12421100
N	-4.34580500	0.15097000	6.12421100
N	-2.04215800	3.83906200	6.12421100

N	-2.30364600	-3.68809200	6.12421100
H	4.34488800	-1.15900600	5.96037300
H	1.16871600	-4.34228700	5.96037300
H	3.17617300	3.18328100	5.96037300
H	-4.34488800	1.15900600	5.96037300
H	-1.16871600	4.34228700	5.96037300
H	-3.17617300	-3.18328100	5.96037300
N	1.78848400	4.44135200	9.56942200
N	4.74056600	0.67180300	9.56942200
N	2.95208100	-3.76954900	9.56942200
N	-4.74056600	-0.67180300	9.56942200
N	-2.95208100	3.76954900	9.56942200
N	-1.78848400	-4.44135200	9.56942200
C	5.24105200	0.28673200	10.88113500
H	6.21546700	0.75507900	11.06914000
H	5.35304500	-0.79601300	10.89766000
C	2.37220800	4.68225000	10.88113500
H	2.45381500	5.76029200	11.06914000
H	3.36589000	4.23786600	10.89766000
C	-2.86884300	4.39551800	10.88113500
H	-1.98715500	5.03387900	10.89766000
H	-3.76165100	5.00521300	11.06914000
C	-5.24105200	-0.28673200	10.88113500
H	-6.21546700	-0.75507900	11.06914000
H	-5.35304500	0.79601300	10.89766000
C	-2.37220800	-4.68225000	10.88113500
H	-3.36589000	-4.23786600	10.89766000
H	-2.45381500	-5.76029200	11.06914000
C	2.86884300	-4.39551800	10.88113500
H	3.76165100	-5.00521300	11.06914000
H	1.98715500	-5.03387900	10.89766000
C	-1.49331000	-4.06506800	11.96627700
O	-1.84363800	-3.19137900	12.74833800
C	4.26710700	0.73929000	11.96627700
O	3.68563500	-0.00094800	12.74833800

C	1.49331000	4.06506800	11.96627700
O	1.84363800	3.19137900	12.74833800
C	-2.77379800	3.32577800	11.96627700
O	-1.84199600	3.19232700	12.74833800
C	-4.26710700	-0.73929000	11.96627700
O	-3.68563500	0.00094800	12.74833800
C	2.77379800	-3.32577800	11.96627700
O	1.84199600	-3.19232700	12.74833800
O	-0.27627600	-4.58749000	11.94317600
H	0.37889000	-4.05445000	12.48005300
O	4.11102100	2.05448300	11.94317600
H	3.32181200	2.35535300	12.48005300
O	0.27627600	4.58749000	11.94317600
H	-0.37889000	4.05445000	12.48005300
O	-3.83474500	2.53300700	11.94317600
H	-3.70070200	1.69909700	12.48005300
O	-4.11102100	-2.05448300	11.94317600
H	-3.32181200	-2.35535300	12.48005300
O	3.83474500	-2.53300700	11.94317600
H	3.70070200	-1.69909700	12.48005300
C	1.80037400	4.29324200	2.55344600
C	2.62836200	4.54434600	1.28744500
H	1.41605300	3.26334200	2.53725000
H	0.92330500	4.95088900	2.56235400
C	1.82871800	4.30674000	0.00000000
H	3.51442500	3.89271300	1.29180100
H	3.00866900	5.57661700	1.29761900
C	-4.61824400	-0.58745100	2.55344600
C	-5.24970000	0.00405600	1.28744500
H	-3.53416400	-0.40533300	2.53725000
H	-4.74924800	-1.67583900	2.56235400
C	-4.64410500	-0.56965400	0.00000000
H	-5.12840100	1.09722500	1.29180100
H	-6.33382700	-0.18272500	1.29761900
C	4.61824400	0.58745100	2.55344600

C	5.24970000	-0.00405600	1.28744500
H	3.53416400	0.40533300	2.53725000
H	4.74924800	1.67583900	2.56235400
C	4.64410500	0.56965400	0.00000000
H	5.12840100	-1.09722500	1.29180100
H	6.33382700	0.18272500	1.29761900
C	2.81787000	-3.70579100	2.55344600
C	2.62133800	-4.54840200	1.28744500
H	2.11811100	-2.85800900	2.53725000
H	3.82594300	-3.27505000	2.56235400
C	2.81538700	-3.73708600	0.00000000
H	1.61397600	-4.98993800	1.29180100
H	3.32515800	-5.39389200	1.29761900
C	-2.81787000	3.70579100	2.55344600
C	-2.62133800	4.54840200	1.28744500
H	-2.11811100	2.85800900	2.53725000
H	-3.82594300	3.27505000	2.56235400
C	-2.81538700	3.73708600	0.00000000
H	-1.61397600	4.98993800	1.29180100
H	-3.32515800	5.39389200	1.29761900
C	-1.80037400	-4.29324200	2.55344600
C	-2.62836200	-4.54434600	1.28744500
H	-1.41605300	-3.26334200	2.53725000
H	-0.92330500	-4.95088900	2.56235400
C	-1.82871800	-4.30674000	0.00000000
H	-3.51442500	-3.89271300	1.29180100
H	-3.00866900	-5.57661700	1.29761900
C	2.43234200	3.74223000	-8.60348100
O	3.58626000	3.31393800	-8.71474200
C	4.45703700	-0.23535500	-8.60348100
O	4.66308500	-1.44882300	-8.71474200
C	2.02469500	-3.97758500	-8.60348100
O	1.07682500	-4.76276100	-8.71474200
C	-4.45703700	0.23535500	-8.60348100
O	-4.66308500	1.44882300	-8.71474200

C	-2.02469500	3.97758500	-8.60348100
O	-1.07682500	4.76276100	-8.71474200
C	-2.43234200	-3.74223000	-8.60348100
O	-3.58626000	-3.31393800	-8.71474200
H	0.82584600	4.73614800	-9.40414000
H	4.51454800	1.65287000	-9.40414000
H	3.68870100	-3.08327800	-9.40414000
H	-4.51454800	-1.65287000	-9.40414000
H	-3.68870100	3.08327800	-9.40414000
H	-0.82584600	-4.73614800	-9.40414000
C	1.58473400	3.47040900	-7.36006700
H	0.70099100	4.10625300	-7.35256600
H	1.24351200	2.42459200	-7.43638800
C	3.79782900	0.36278500	-7.36006700
H	3.90661500	1.44605100	-7.35256600
H	2.72151400	0.13538300	-7.43638800
C	2.21309600	-3.10762400	-7.36006700
H	3.20562400	-2.66020200	-7.35256600
H	1.47800200	-2.28920900	-7.43638800
C	-3.79782900	-0.36278500	-7.36006700
H	-3.90661500	-1.44605100	-7.35256600
H	-2.72151400	-0.13538300	-7.43638800
C	-2.21309600	3.10762400	-7.36006700
H	-3.20562400	2.66020200	-7.35256600
H	-1.47800200	2.28920900	-7.43638800
C	-1.58473400	-3.47040900	-7.36006700
H	-0.70099100	-4.10625300	-7.35256600
H	-1.24351200	-2.42459200	-7.43638800
C	1.72617800	4.35562800	-5.09058400
O	0.58394900	4.83228600	-5.15087100
C	4.63517300	0.68290000	-5.09058400
O	4.47685700	1.91042800	-5.15087100
C	2.90899500	-3.67272800	-5.09058400
O	3.89290700	-2.92185800	-5.15087100
C	-4.63517300	-0.68290000	-5.09058400

O	-4.47685700	-1.91042800	-5.15087100
C	-2.90899500	3.67272800	-5.09058400
O	-3.89290700	2.92185800	-5.15087100
C	-1.72617800	-4.35562800	-5.09058400
O	-0.58394900	-4.83228600	-5.15087100
C	2.59338700	4.51379400	-3.84880600
H	3.45478500	3.83976600	-3.90111400
H	2.98590500	5.54084500	-3.86532600
C	5.20575400	0.01095800	-3.84880600
H	5.05272700	-1.07204800	-3.90111400
H	6.29146400	0.18455300	-3.86532600
C	2.61236700	-4.50283600	-3.84880600
H	1.59794200	-4.91181400	-3.90111400
H	3.30556000	-5.35629100	-3.86532600
C	-5.20575400	-0.01095800	-3.84880600
H	-5.05272700	1.07204800	-3.90111400
H	-6.29146400	-0.18455300	-3.86532600
C	-2.61236700	4.50283600	-3.84880600
H	-1.59794200	4.91181400	-3.90111400
H	-3.30556000	5.35629100	-3.86532600
C	-2.59338700	-4.51379400	-3.84880600
H	-3.45478500	-3.83976600	-3.90111400
H	-2.98590500	-5.54084500	-3.86532600
N	2.30364600	3.68809200	-6.12421100
N	4.34580500	-0.15097000	-6.12421100
N	2.04215800	-3.83906200	-6.12421100
N	-4.34580500	0.15097000	-6.12421100
N	-2.04215800	3.83906200	-6.12421100
N	-2.30364600	-3.68809200	-6.12421100
H	4.34488800	-1.15900600	-5.96037300
H	1.16871600	-4.34228700	-5.96037300
H	3.17617300	3.18328100	-5.96037300
H	-4.34488800	1.15900600	-5.96037300
H	-1.16871600	4.34228700	-5.96037300
H	-3.17617300	-3.18328100	-5.96037300

N	1.78848400	4.44135200	-9.56942200
N	4.74056600	0.67180300	-9.56942200
N	2.95208100	-3.76954900	-9.56942200
N	-4.74056600	-0.67180300	-9.56942200
N	-2.95208100	3.76954900	-9.56942200
N	-1.78848400	-4.44135200	-9.56942200
C	5.24105200	0.28673200	-10.88113500
H	6.21546700	0.75507900	-11.06914000
H	5.35304500	-0.79601300	-10.89766000
C	2.37220800	4.68225000	-10.88113500
H	2.45381500	5.76029200	-11.06914000
H	3.36589000	4.23786600	-10.89766000
C	-2.86884300	4.39551800	-10.88113500
H	-1.98715500	5.03387900	-10.89766000
H	-3.76165100	5.00521300	-11.06914000
C	-5.24105200	-0.28673200	-10.88113500
H	-6.21546700	-0.75507900	-11.06914000
H	-5.35304500	0.79601300	-10.89766000
C	-2.37220800	-4.68225000	-10.88113500
H	-3.36589000	-4.23786600	-10.89766000
H	-2.45381500	-5.76029200	-11.06914000
C	2.86884300	-4.39551800	-10.88113500
H	3.76165100	-5.00521300	-11.06914000
H	1.98715500	-5.03387900	-10.89766000
C	-1.49331000	-4.06506800	-11.96627700
O	-1.84363800	-3.19137900	-12.74833800
C	4.26710700	0.73929000	-11.96627700
O	3.68563500	-0.00094800	-12.74833800
C	1.49331000	4.06506800	-11.96627700
O	1.84363800	3.19137900	-12.74833800
C	-2.77379800	3.32577800	-11.96627700
O	-1.84199600	3.19232700	-12.74833800
C	-4.26710700	-0.73929000	-11.96627700
O	-3.68563500	0.00094800	-12.74833800
C	2.77379800	-3.32577800	-11.96627700

O	1.84199600	-3.19232700	-12.74833800
O	-0.27627600	-4.58749000	-11.94317600
H	0.37889000	-4.05445000	-12.48005300
O	4.11102100	2.05448300	-11.94317600
H	3.32181200	2.35535300	-12.48005300
O	0.27627600	4.58749000	-11.94317600
H	-0.37889000	4.05445000	-12.48005300
O	-3.83474500	2.53300700	-11.94317600
H	-3.70070200	1.69909700	-12.48005300
O	-4.11102100	-2.05448300	-11.94317600
H	-3.32181200	-2.35535300	-12.48005300
O	3.83474500	-2.53300700	-11.94317600
H	3.70070200	-1.69909700	-12.48005300
C	1.80037400	4.29324200	-2.55344600
C	2.62836200	4.54434600	-1.28744500
H	1.41605300	3.26334200	-2.53725000
H	0.92330500	4.95088900	-2.56235400
H	3.51442500	3.89271300	-1.29180100
H	3.00866900	5.57661700	-1.29761900
C	-4.61824400	-0.58745100	-2.55344600
C	-5.24970000	0.00405600	-1.28744500
H	-3.53416400	-0.40533300	-2.53725000
H	-4.74924800	-1.67583900	-2.56235400
H	-5.12840100	1.09722500	-1.29180100
H	-6.33382700	-0.18272500	-1.29761900
C	4.61824400	0.58745100	-2.55344600
C	5.24970000	-0.00405600	-1.28744500
H	3.53416400	0.40533300	-2.53725000
H	4.74924800	1.67583900	-2.56235400
H	5.12840100	-1.09722500	-1.29180100
H	6.33382700	0.18272500	-1.29761900
C	2.81787000	-3.70579100	-2.55344600
C	2.62133800	-4.54840200	-1.28744500
H	2.11811100	-2.85800900	-2.53725000
H	3.82594300	-3.27505000	-2.56235400

H	1.61397600	-4.98993800	-1.29180100
H	3.32515800	-5.39389200	-1.29761900
C	-2.81787000	3.70579100	-2.55344600
C	-2.62133800	4.54840200	-1.28744500
H	-2.11811100	2.85800900	-2.53725000
H	-3.82594300	3.27505000	-2.56235400
H	-1.61397600	4.98993800	-1.29180100
H	-3.32515800	5.39389200	-1.29761900
C	-1.80037400	-4.29324200	-2.55344600
C	-2.62836200	-4.54434600	-1.28744500
H	-1.41605300	-3.26334200	-2.53725000
H	-0.92330500	-4.95088900	-2.56235400
H	-3.51442500	-3.89271300	-1.29180100
H	-3.00866900	-5.57661700	-1.29761900
H	4.76641100	1.66313800	0.00000000
H	3.55933100	0.38590000	0.00000000
H	1.44546600	3.27542100	0.00000000
H	0.94288600	4.95940100	0.00000000
H	-2.11386500	2.88952100	0.00000000
H	-3.82352500	3.29626400	0.00000000
H	-3.55933100	-0.38590000	0.00000000
H	-4.76641100	-1.66313800	0.00000000
H	-0.94288600	-4.95940100	0.00000000
H	-1.44546600	-3.27542100	0.00000000
H	3.82352500	-3.29626400	0.00000000
H	2.11386500	-2.88952100	0.00000000

[P-$(CH_2)_7$-P]$_8$

C	-2.47042800	5.43797400	8.57408900
O	-3.67363900	5.17420200	8.68040500
C	-5.59208500	2.09837200	8.57408900
O	-6.25636800	1.06105800	8.68040500
C	2.09837200	5.59208500	8.57408900
O	1.06105800	6.25636800	8.68040500
C	5.43797400	2.47042800	8.57408900
O	5.17420200	3.67363900	8.68040500

H	3.87050900	4.93245300	9.36901300
H	-0.75090800	6.22463400	9.36901300
H	-4.93245300	3.87050900	9.36901300
H	6.22463400	0.75090800	9.36901300
C	2.39423100	4.76295300	7.32384500
H	1.74707100	3.87256600	7.38066600
H	3.42927100	4.42441100	7.32151400
C	-1.67493900	5.06089300	7.32384500
H	-1.50295200	3.97368300	7.38066600
H	-0.70367000	5.55339200	7.32151400
C	-4.76295300	2.39423100	7.32384500
H	-3.87256600	1.74707100	7.38066600
H	-4.42441100	3.42927100	7.32151400
C	5.06089300	1.67493900	7.32384500
H	3.97368300	1.50295200	7.38066600
H	5.55339200	0.70367000	7.32151400
C	-1.70815700	6.03687000	5.08432600
O	-0.51543400	6.36716000	5.14926700
C	-5.47656100	3.06086200	5.08432600
O	-4.86672900	4.13779500	5.14926700
C	3.06086200	5.47656100	5.08432600
O	4.13779500	4.86672900	5.14926700
C	6.03687000	1.70815700	5.08432600
O	6.36716000	0.51543400	5.14926700
C	2.68026400	6.28418900	3.85081000
H	1.64137500	6.62210600	3.92143400
H	3.31649300	7.18065500	3.85156500
C	-2.54836000	6.33882600	3.85081000
H	-3.52190900	5.84316300	3.92143400
H	-2.73237500	7.42260500	3.85156500
C	-6.28418900	2.68026400	3.85081000
H	-6.62210600	1.64137500	3.92143400
H	-7.18065500	3.31649300	3.85156500
C	6.33882600	2.54836000	3.85081000
H	5.84316300	3.52190900	3.92143400

H	7.42260500	2.73237500	3.85156500
N	-2.35872400	5.40798200	6.09679200
N	-5.49189000	2.15615100	6.09679200
N	2.15615100	5.49189000	6.09679200
N	5.40798200	2.35872400	6.09679200
H	-3.30203100	5.05082100	5.93559900
H	-5.90635800	1.23658100	5.93559900
H	1.23658100	5.90635800	5.93559900
H	5.05082100	3.30203100	5.93559900
N	3.03362700	5.49000100	9.54677800
N	-1.73691900	6.02711500	9.54677800
N	-5.49000100	3.03362700	9.54677800
N	6.02711500	1.73691900	9.54677800
C	-2.27417800	6.31939100	10.86825800
H	-2.21307600	7.39504500	11.07457500
H	-3.31512200	6.00368400	10.89292400
C	2.86039800	6.07657100	10.86825800
H	3.66420500	6.79396800	11.07457500
H	1.90110000	6.58939100	10.89292400
C	6.31939100	2.27417800	10.86825800
H	7.39504500	2.21307600	11.07457500
H	6.00368400	3.31512200	10.89292400
C	-6.07657100	2.86039800	10.86825800
H	-6.79396800	3.66420500	11.07457500
H	-6.58939100	1.90110000	10.89292400
C	2.90730800	4.97272500	11.92370600
O	1.98144900	4.64084900	12.65083500
C	-1.46047000	5.57202500	11.92370600
O	-1.88048000	4.68267200	12.65083500
C	5.57202500	1.46047000	11.92370600
O	4.68267200	1.88048000	12.65083500
C	-4.97272500	2.90730800	11.92370600
O	-4.64084900	1.98144900	12.65083500
O	4.09752300	4.38783300	11.92781900
H	4.12116000	3.52946000	12.43656300

O	-0.20528000	6.00005300	11.92781900
H	0.41839500	5.40980500	12.43656300
O	6.00005300	0.20528000	11.92781900
H	5.40980500	-0.41839500	12.43656300
O	-4.38783300	4.09752300	11.92781900
H	-3.52946000	4.12116000	12.43656300
C	2.47042800	-5.43797400	8.57408900
O	3.67363900	-5.17420200	8.68040500
C	5.59208500	-2.09837200	8.57408900
O	6.25636800	-1.06105800	8.68040500
C	-2.09837200	-5.59208500	8.57408900
O	-1.06105800	-6.25636800	8.68040500
C	-5.43797400	-2.47042800	8.57408900
O	-5.17420200	-3.67363900	8.68040500
H	-3.87050900	-4.93245300	9.36901300
H	0.75090800	-6.22463400	9.36901300
H	4.93245300	-3.87050900	9.36901300
H	-6.22463400	-0.75090800	9.36901300
C	-2.39423100	-4.76295300	7.32384500
H	-1.74707100	-3.87256600	7.38066600
H	-3.42927100	-4.42441100	7.32151400
C	1.67493900	-5.06089300	7.32384500
H	1.50295200	-3.97368300	7.38066600
H	0.70367000	-5.55339200	7.32151400
C	4.76295300	-2.39423100	7.32384500
H	3.87256600	-1.74707100	7.38066600
H	4.42441100	-3.42927100	7.32151400
C	-5.06089300	-1.67493900	7.32384500
H	-3.97368300	-1.50295200	7.38066600
H	-5.55339200	-0.70367000	7.32151400
C	1.70815700	-6.03687000	5.08432600
O	0.51543400	-6.36716000	5.14926700
C	5.47656100	-3.06086200	5.08432600
O	4.86672900	-4.13779500	5.14926700
C	-3.06086200	-5.47656100	5.08432600

O	-4.13779500	-4.86672900	5.14926700
C	-6.03687000	-1.70815700	5.08432600
O	-6.36716000	-0.51543400	5.14926700
C	-2.68026400	-6.28418900	3.85081000
H	-1.64137500	-6.62210600	3.92143400
H	-3.31649300	-7.18065500	3.85156500
C	2.54836000	-6.33882600	3.85081000
H	3.52190900	-5.84316300	3.92143400
H	2.73237500	-7.42260500	3.85156500
C	6.28418900	-2.68026400	3.85081000
H	6.62210600	-1.64137500	3.92143400
H	7.18065500	-3.31649300	3.85156500
C	-6.33882600	-2.54836000	3.85081000
H	-5.84316300	-3.52190900	3.92143400
H	-7.42260500	-2.73237500	3.85156500
N	2.35872400	-5.40798200	6.09679200
N	5.49189000	-2.15615100	6.09679200
N	-2.15615100	-5.49189000	6.09679200
N	-5.40798200	-2.35872400	6.09679200
H	3.30203100	-5.05082100	5.93559900
H	5.90635800	-1.23658100	5.93559900
H	-1.23658100	-5.90635800	5.93559900
H	-5.05082100	-3.30203100	5.93559900
N	-3.03362700	-5.49000100	9.54677800
N	1.73691900	-6.02711500	9.54677800
N	5.49000100	-3.03362700	9.54677800
N	-6.02711500	-1.73691900	9.54677800
C	2.27417800	-6.31939100	10.86825800
H	2.21307600	-7.39504500	11.07457500
H	3.31512200	-6.00368400	10.89292400
C	-2.86039800	-6.07657100	10.86825800
H	-3.66420500	-6.79396800	11.07457500
H	-1.90110000	-6.58939100	10.89292400
C	-6.31939100	-2.27417800	10.86825800
H	-7.39504500	-2.21307600	11.07457500

H	-6.00368400	-3.31512200	10.89292400
C	6.07657100	-2.86039800	10.86825800
H	6.79396800	-3.66420500	11.07457500
H	6.58939100	-1.90110000	10.89292400
C	-2.90730800	-4.97272500	11.92370600
O	-1.98144900	-4.64084900	12.65083500
C	1.46047000	-5.57202500	11.92370600
O	1.88048000	-4.68267200	12.65083500
C	-5.57202500	-1.46047000	11.92370600
O	-4.68267200	-1.88048000	12.65083500
C	4.97272500	-2.90730800	11.92370600
O	4.64084900	-1.98144900	12.65083500
O	-4.09752300	-4.38783300	11.92781900
H	-4.12116000	-3.52946000	12.43656300
O	0.20528000	-6.00005300	11.92781900
H	-0.41839500	-5.40980500	12.43656300
O	-6.00005300	-0.20528000	11.92781900
H	-5.40980500	0.41839500	12.43656300
O	4.38783300	-4.09752300	11.92781900
H	3.52946000	-4.12116000	12.43656300
C	-1.82571000	5.94865200	2.55300900
C	-2.59445100	6.34795400	1.28773800
H	-1.65572700	4.86257300	2.54751600
H	-0.83480100	6.41784800	2.54918200
C	-1.86058800	5.95216000	0.00000000
H	-3.59083600	5.88202700	1.29901700
H	-2.76590200	7.43467400	1.29222500
H	-0.86123100	6.41261600	0.00000000
H	-1.69292100	4.86492700	0.00000000
C	-5.49730400	2.91536000	2.55300900
C	-6.32323500	2.65412700	1.28773800
H	-4.60913400	2.26758300	2.54751600
H	-5.12839700	3.94781000	2.54918200
C	-5.52444700	2.89317800	0.00000000
H	-6.69832600	1.62011700	1.29901700

H	-7.21289600	3.30132000	1.29222500
H	-5.14338700	3.92542200	0.00000000
H	-4.63709900	2.24294700	0.00000000
C	-5.94865200	-1.82571000	2.55300900
C	-6.34795400	-2.59445100	1.28773800
H	-4.86257300	-1.65572700	2.54751600
H	-6.41784800	-0.83480100	2.54918200
C	-5.95216000	-1.86058800	0.00000000
H	-5.88202700	-3.59083600	1.29901700
H	-7.43467400	-2.76590200	1.29222500
H	-6.41261600	-0.86123100	0.00000000
H	-4.86492700	-1.69292100	0.00000000
C	2.91536000	5.49730400	2.55300900
C	2.65412700	6.32323500	1.28773800
H	2.26758300	4.60913400	2.54751600
H	3.94781000	5.12839700	2.54918200
C	2.89317800	5.52444700	0.00000000
H	1.62011700	6.69832600	1.29901700
H	3.30132000	7.21289600	1.29222500
H	3.92542200	5.14338700	0.00000000
H	2.24294700	4.63709900	0.00000000
C	5.49730400	-2.91536000	2.55300900
C	6.32323500	-2.65412700	1.28773800
H	4.60913400	-2.26758300	2.54751600
H	5.12839700	-3.94781000	2.54918200
C	5.52444700	-2.89317800	0.00000000
H	6.69832600	-1.62011700	1.29901700
H	7.21289600	-3.30132000	1.29222500
H	5.14338700	-3.92542200	0.00000000
H	4.63709900	-2.24294700	0.00000000
C	5.94865200	1.82571000	2.55300900
C	6.34795400	2.59445100	1.28773800
H	4.86257300	1.65572700	2.54751600
H	6.41784800	0.83480100	2.54918200
C	5.95216000	1.86058800	0.00000000

H	5.88202700	3.59083600	1.29901700
H	7.43467400	2.76590200	1.29222500
H	6.41261600	0.86123100	0.00000000
H	4.86492700	1.69292100	0.00000000
C	1.82571000	-5.94865200	2.55300900
C	2.59445100	-6.34795400	1.28773800
H	1.65572700	-4.86257300	2.54751600
H	0.83480100	-6.41784800	2.54918200
C	1.86058800	-5.95216000	0.00000000
H	3.59083600	-5.88202700	1.29901700
H	2.76590200	-7.43467400	1.29222500
H	0.86123100	-6.41261600	0.00000000
H	1.69292100	-4.86492700	0.00000000
C	-2.91536000	-5.49730400	2.55300900
C	-2.65412700	-6.32323500	1.28773800
H	-2.26758300	-4.60913400	2.54751600
H	-3.94781000	-5.12839700	2.54918200
C	-2.89317800	-5.52444700	0.00000000
H	-1.62011700	-6.69832600	1.29901700
H	-3.30132000	-7.21289600	1.29222500
H	-3.92542200	-5.14338700	0.00000000
H	-2.24294700	-4.63709900	0.00000000
C	-2.47042800	5.43797400	-8.57408900
O	-3.67363900	5.17420200	-8.68040500
C	-5.59208500	2.09837200	-8.57408900
O	-6.25636800	1.06105800	-8.68040500
C	2.09837200	5.59208500	-8.57408900
O	1.06105800	6.25636800	-8.68040500
C	5.43797400	2.47042800	-8.57408900
O	5.17420200	3.67363900	-8.68040500
H	3.87050900	4.93245300	-9.36901300
H	-0.75090800	6.22463400	-9.36901300
H	-4.93245300	3.87050900	-9.36901300
H	6.22463400	0.75090800	-9.36901300
C	2.39423100	4.76295300	-7.32384500

H	1.74707100	3.87256600	-7.38066600
H	3.42927100	4.42441100	-7.32151400
C	-1.67493900	5.06089300	-7.32384500
H	-1.50295200	3.97368300	-7.38066600
H	-0.70367000	5.55339200	-7.32151400
C	-4.76295300	2.39423100	-7.32384500
H	-3.87256600	1.74707100	-7.38066600
H	-4.42441100	3.42927100	-7.32151400
C	5.06089300	1.67493900	-7.32384500
H	3.97368300	1.50295200	-7.38066600
H	5.55339200	0.70367000	-7.32151400
C	-1.70815700	6.03687000	-5.08432600
O	-0.51543400	6.36716000	-5.14926700
C	-5.47656100	3.06086200	-5.08432600
O	-4.86672900	4.13779500	-5.14926700
C	3.06086200	5.47656100	-5.08432600
O	4.13779500	4.86672900	-5.14926700
C	6.03687000	1.70815700	-5.08432600
O	6.36716000	0.51543400	-5.14926700
C	2.68026400	6.28418900	-3.85081000
H	1.64137500	6.62210600	-3.92143400
H	3.31649300	7.18065500	-3.85156500
C	-2.54836000	6.33882600	-3.85081000
H	-3.52190900	5.84316300	-3.92143400
H	-2.73237500	7.42260500	-3.85156500
C	-6.28418900	2.68026400	-3.85081000
H	-6.62210600	1.64137500	-3.92143400
H	-7.18065500	3.31649300	-3.85156500
C	6.33882600	2.54836000	-3.85081000
H	5.84316300	3.52190900	-3.92143400
H	7.42260500	2.73237500	-3.85156500
N	-2.35872400	5.40798200	-6.09679200
N	-5.49189000	2.15615100	-6.09679200
N	2.15615100	5.49189000	-6.09679200
N	5.40798200	2.35872400	-6.09679200

H	-3.30203100	5.05082100	-5.93559900
H	-5.90635800	1.23658100	-5.93559900
H	1.23658100	5.90635800	-5.93559900
H	5.05082100	3.30203100	-5.93559900
N	3.03362700	5.49000100	-9.54677800
N	-1.73691900	6.02711500	-9.54677800
N	-5.49000100	3.03362700	-9.54677800
N	6.02711500	1.73691900	-9.54677800
C	-2.27417800	6.31939100	-10.86825800
H	-2.21307600	7.39504500	-11.07457500
H	-3.31512200	6.00368400	-10.89292400
C	2.86039800	6.07657100	-10.86825800
H	3.66420500	6.79396800	-11.07457500
H	1.90110000	6.58939100	-10.89292400
C	6.31939100	2.27417800	-10.86825800
H	7.39504500	2.21307600	-11.07457500
H	6.00368400	3.31512200	-10.89292400
C	-6.07657100	2.86039800	-10.86825800
H	-6.79396800	3.66420500	-11.07457500
H	-6.58939100	1.90110000	-10.89292400
C	2.90730800	4.97272500	-11.92370600
O	1.98144900	4.64084900	-12.65083500
C	-1.46047000	5.57202500	-11.92370600
O	-1.88048000	4.68267200	-12.65083500
C	5.57202500	1.46047000	-11.92370600
O	4.68267200	1.88048000	-12.65083500
C	-4.97272500	2.90730800	-11.92370600
O	-4.64084900	1.98144900	-12.65083500
O	4.09752300	4.38783300	-11.92781900
H	4.12116000	3.52946000	-12.43656300
O	-0.20528000	6.00005300	-11.92781900
H	0.41839500	5.40980500	-12.43656300
O	6.00005300	0.20528000	-11.92781900
H	5.40980500	-0.41839500	-12.43656300
O	-4.38783300	4.09752300	-11.92781900

H	-3.52946000	4.12116000	-12.43656300
C	2.47042800	-5.43797400	-8.57408900
O	3.67363900	-5.17420200	-8.68040500
C	5.59208500	-2.09837200	-8.57408900
O	6.25636800	-1.06105800	-8.68040500
C	-2.09837200	-5.59208500	-8.57408900
O	-1.06105800	-6.25636800	-8.68040500
C	-5.43797400	-2.47042800	-8.57408900
O	-5.17420200	-3.67363900	-8.68040500
H	-3.87050900	-4.93245300	-9.36901300
H	0.75090800	-6.22463400	-9.36901300
H	4.93245300	-3.87050900	-9.36901300
H	-6.22463400	-0.75090800	-9.36901300
C	-2.39423100	-4.76295300	-7.32384500
H	-1.74707100	-3.87256600	-7.38066600
H	-3.42927100	-4.42441100	-7.32151400
C	1.67493900	-5.06089300	-7.32384500
H	1.50295200	-3.97368300	-7.38066600
H	0.70367000	-5.55339200	-7.32151400
C	4.76295300	-2.39423100	-7.32384500
H	3.87256600	-1.74707100	-7.38066600
H	4.42441100	-3.42927100	-7.32151400
C	-5.06089300	-1.67493900	-7.32384500
H	-3.97368300	-1.50295200	-7.38066600
H	-5.55339200	-0.70367000	-7.32151400
C	1.70815700	-6.03687000	-5.08432600
O	0.51543400	-6.36716000	-5.14926700
C	5.47656100	-3.06086200	-5.08432600
O	4.86672900	-4.13779500	-5.14926700
C	-3.06086200	-5.47656100	-5.08432600
O	-4.13779500	-4.86672900	-5.14926700
C	-6.03687000	-1.70815700	-5.08432600
O	-6.36716000	-0.51543400	-5.14926700
C	-2.68026400	-6.28418900	-3.85081000
H	-1.64137500	-6.62210600	-3.92143400

H	-3.31649300	-7.18065500	-3.85156500
C	2.54836000	-6.33882600	-3.85081000
H	3.52190900	-5.84316300	-3.92143400
H	2.73237500	-7.42260500	-3.85156500
C	6.28418900	-2.68026400	-3.85081000
H	6.62210600	-1.64137500	-3.92143400
H	7.18065500	-3.31649300	-3.85156500
C	-6.33882600	-2.54836000	-3.85081000
H	-5.84316300	-3.52190900	-3.92143400
H	-7.42260500	-2.73237500	-3.85156500
N	2.35872400	-5.40798200	-6.09679200
N	5.49189000	-2.15615100	-6.09679200
N	-2.15615100	-5.49189000	-6.09679200
N	-5.40798200	-2.35872400	-6.09679200
H	3.30203100	-5.05082100	-5.93559900
H	5.90635800	-1.23658100	-5.93559900
H	-1.23658100	-5.90635800	-5.93559900
H	-5.05082100	-3.30203100	-5.93559900
N	-3.03362700	-5.49000100	-9.54677800
N	1.73691900	-6.02711500	-9.54677800
N	5.49000100	-3.03362700	-9.54677800
N	-6.02711500	-1.73691900	-9.54677800
C	2.27417800	-6.31939100	-10.86825800
H	2.21307600	-7.39504500	-11.07457500
H	3.31512200	-6.00368400	-10.89292400
C	-2.86039800	-6.07657100	-10.86825800
H	-3.66420500	-6.79396800	-11.07457500
H	-1.90110000	-6.58939100	-10.89292400
C	-6.31939100	-2.27417800	-10.86825800
H	-7.39504500	-2.21307600	-11.07457500
H	-6.00368400	-3.31512200	-10.89292400
C	6.07657100	-2.86039800	-10.86825800
H	6.79396800	-3.66420500	-11.07457500
H	6.58939100	-1.90110000	-10.89292400
C	-2.90730800	-4.97272500	-11.92370600

O	-1.98144900	-4.64084900	-12.65083500
C	1.46047000	-5.57202500	-11.92370600
O	1.88048000	-4.68267200	-12.65083500
C	-5.57202500	-1.46047000	-11.92370600
O	-4.68267200	-1.88048000	-12.65083500
C	4.97272500	-2.90730800	-11.92370600
O	4.64084900	-1.98144900	-12.65083500
O	-4.09752300	-4.38783300	-11.92781900
H	-4.12116000	-3.52946000	-12.43656300
O	0.20528000	-6.00005300	-11.92781900
H	-0.41839500	-5.40980500	-12.43656300
O	-6.00005300	-0.20528000	-11.92781900
H	-5.40980500	0.41839500	-12.43656300
O	4.38783300	-4.09752300	-11.92781900
H	3.52946000	-4.12116000	-12.43656300
C	-1.82571000	5.94865200	-2.55300900
C	-2.59445100	6.34795400	-1.28773800
H	-1.65572700	4.86257300	-2.54751600
H	-0.83480100	6.41784800	-2.54918200
H	-3.59083600	5.88202700	-1.29901700
H	-2.76590200	7.43467400	-1.29222500
C	-5.49730400	2.91536000	-2.55300900
C	-6.32323500	2.65412700	-1.28773800
H	-4.60913400	2.26758300	-2.54751600
H	-5.12839700	3.94781000	-2.54918200
H	-6.69832600	1.62011700	-1.29901700
H	-7.21289600	3.30132000	-1.29222500
C	-5.94865200	-1.82571000	-2.55300900
C	-6.34795400	-2.59445100	-1.28773800
H	-4.86257300	-1.65572700	-2.54751600
H	-6.41784800	-0.83480100	-2.54918200
H	-5.88202700	-3.59083600	-1.29901700
H	-7.43467400	-2.76590200	-1.29222500
C	2.91536000	5.49730400	-2.55300900
C	2.65412700	6.32323500	-1.28773800

H	2.26758300	4.60913400	-2.54751600
H	3.94781000	5.12839700	-2.54918200
H	1.62011700	6.69832600	-1.29901700
H	3.30132000	7.21289600	-1.29222500
C	5.49730400	-2.91536000	-2.55300900
C	6.32323500	-2.65412700	-1.28773800
H	4.60913400	-2.26758300	-2.54751600
H	5.12839700	-3.94781000	-2.54918200
H	6.69832600	-1.62011700	-1.29901700
H	7.21289600	-3.30132000	-1.29222500
C	5.94865200	1.82571000	-2.55300900
C	6.34795400	2.59445100	-1.28773800
H	4.86257300	1.65572700	-2.54751600
H	6.41784800	0.83480100	-2.54918200
H	5.88202700	3.59083600	-1.29901700
H	7.43467400	2.76590200	-1.29222500
C	1.82571000	-5.94865200	-2.55300900
C	2.59445100	-6.34795400	-1.28773800
H	1.65572700	-4.86257300	-2.54751600
H	0.83480100	-6.41784800	-2.54918200
H	3.59083600	-5.88202700	-1.29901700
H	2.76590200	-7.43467400	-1.29222500
C	-2.91536000	-5.49730400	-2.55300900
C	-2.65412700	-6.32323500	-1.28773800
H	-2.26758300	-4.60913400	-2.54751600
H	-3.94781000	-5.12839700	-2.54918200
H	-1.62011700	-6.69832600	-1.29901700
H	-3.30132000	-7.21289600	-1.29222500

$[P\text{-}(CH_2)_7\text{-}P]_{10}$

C	-4.72175600	5.81473500	8.55660100
O	-3.93678800	6.76486400	8.65662300
C	-0.40216600	7.47959800	8.55660100
O	0.79135900	7.78687600	8.65662300
C	-7.23779600	1.92884100	8.55660100
O	-7.16121500	3.15890400	8.65662300

C	-4.07103800	-6.28750800	8.55660100
O	-5.21723300	-5.83456600	8.65662300
C	-6.98924400	-2.69380500	8.55660100
O	-7.65030200	-1.65365000	8.65662300
H	-7.75361100	0.11416600	9.36046300
H	-6.20569800	4.64982100	9.36046300
H	-2.28741900	7.40940200	9.36046300
H	-2.50457600	-7.33884300	9.36046300
H	-6.33990800	-4.46509600	9.36046300
C	-6.76637900	1.20071200	7.29783200
H	-5.66499800	1.20337300	7.33259600
H	-7.09941200	0.16374300	7.30114900
C	-4.76835500	4.94857400	7.29783200
H	-3.87575500	4.30335200	7.33259600
H	-5.64729900	4.30540000	7.30114900
C	-0.94898100	6.80625000	7.29783200
H	-0.60610500	5.75959600	7.33259600
H	-2.03811000	6.80254100	7.30114900
C	-3.23287100	-6.06416900	7.29783200
H	-2.89505700	-5.01587100	7.33259600
H	-2.34956700	-6.70134300	7.30114900
C	-6.17987700	-3.00578200	7.29783200
H	-5.29040500	-2.35625300	7.33259600
H	-5.83979000	-4.04045900	7.30114900
C	-5.65980600	5.45401800	5.07923200
O	-6.49808200	4.54356200	5.14517400
C	-1.37308800	7.73914400	5.07923200
O	-2.58642000	7.49529600	5.14517400
C	-7.78467100	1.08564300	5.07923200
O	-7.92769800	-0.14365800	5.14517400
C	-3.43810300	-7.06817900	5.07923200
O	-2.31316700	-7.58408100	5.14517400
C	-6.93605600	-3.69741100	5.07923200
O	-6.32920200	-4.77600500	5.14517400
C	-8.23103500	1.86791300	3.85158900

H	-7.93297600	2.91771900	3.93637500
H	-9.32956400	1.83647500	3.83959900
C	-5.56111500	6.34925500	3.85158900
H	-4.70292000	7.02337000	3.93637500
H	-6.46832300	6.96952000	3.83959900
C	-0.76703900	8.40539600	3.85158900
H	0.32349200	8.44633300	3.93637500
H	-1.13640200	9.44044500	3.83959900
C	-4.32002100	-7.25096300	3.85158900
H	-5.22634000	-6.64308300	3.93637500
H	-4.62958600	-8.30544100	3.83959900
C	-7.75697900	-3.32690800	3.85158900
H	-8.13290400	-2.30240200	3.93637500
H	-8.62722900	-3.99804100	3.83959900
N	-4.78642800	5.73140800	6.08061100
N	-0.50346500	7.45019800	6.08061100
N	-7.24113800	1.82341400	6.08061100
N	-3.97180500	-6.32326600	6.08061100
N	-6.92998000	-2.78106100	6.08061100
H	-4.04985300	6.42124000	5.92316100
H	0.49791000	7.57533600	5.92316100
H	-7.05071000	2.81444800	5.92316100
H	-4.85548900	-5.83591100	5.92316100
H	-7.35843500	-1.86736700	5.92316100
N	-6.87360900	-3.61322000	9.54170500
N	-7.68466400	1.11705000	9.54170500
N	-5.56043800	5.42064400	9.54170500
N	-1.31231400	7.65373700	9.54170500
N	-3.43706900	-6.96336200	9.54170500
C	-5.55029600	6.01694800	10.86998300
H	-6.52832700	6.45897600	11.09651300
H	-4.78687800	6.79108800	10.89500300
C	-8.02695700	1.60543100	10.86998300
H	-9.07801800	1.38816700	11.09651300
H	-7.86436700	2.68044900	10.89500300

C	-7.43759400	-3.41930600	10.86998300
H	-7.93793500	-2.45403000	10.89500300
H	-8.16021500	-4.21287400	11.09651300
C	-4.00732200	-7.13798500	10.86998300
H	-4.12548700	-8.20474100	11.09651300
H	-4.97948200	-6.65115300	10.89500300
C	-0.95361000	8.13019600	10.86998300
H	-1.48503700	9.06267500	11.09651300
H	0.11903600	8.30776200	10.89500300
C	-7.14841100	0.90466200	11.90595000
O	-6.29752300	1.43726600	12.60305600
C	-5.25143900	4.93361700	11.90595000
O	-4.25000000	4.86436400	12.60305600
C	-6.31493200	-3.46984400	11.90595000
O	-5.93960700	-2.53881900	12.60305600
C	-3.06936500	-6.51898700	11.90595000
O	-3.31296300	-5.54516100	12.60305600
C	-1.34859600	7.07809800	11.90595000
O	-0.57912100	6.43344000	12.60305600
O	-5.76787800	-4.68001700	11.92386000
H	-4.90816800	-4.73623100	12.42162400
O	-7.41715600	-0.39594000	11.92386000
H	-6.75467900	-0.94674300	12.42162400
O	-6.23333300	4.03937300	11.92386000
H	-6.02113100	3.20437000	12.42162400
O	-1.91546600	-7.17648700	11.92386000
H	-1.18690500	-6.71664100	12.42162400
O	-2.66858900	6.93178300	11.92386000
H	-2.98771600	6.13152200	12.42162400
C	4.72175600	-5.81473500	8.55660100
O	3.93678800	-6.76486400	8.65662300
C	0.40216600	-7.47959800	8.55660100
O	-0.79135900	-7.78687600	8.65662300
C	7.23779600	-1.92884100	8.55660100
O	7.16121500	-3.15890400	8.65662300

C	4.07103800	6.28750800	8.55660100
O	5.21723300	5.83456600	8.65662300
C	6.98924400	2.69380500	8.55660100
O	7.65030200	1.65365000	8.65662300
H	7.75361100	-0.11416600	9.36046300
H	6.20569800	-4.64982100	9.36046300
H	2.28741900	-7.40940200	9.36046300
H	2.50457600	7.33884300	9.36046300
H	6.33990800	4.46509600	9.36046300
C	6.76637900	-1.20071200	7.29783200
H	5.66499800	-1.20337300	7.33259600
H	7.09941200	-0.16374300	7.30114900
C	4.76835500	-4.94857400	7.29783200
H	3.87575500	-4.30335200	7.33259600
H	5.64729900	-4.30540000	7.30114900
C	0.94898100	-6.80625000	7.29783200
H	0.60610500	-5.75959600	7.33259600
H	2.03811000	-6.80254100	7.30114900
C	3.23287100	6.06416900	7.29783200
H	2.89505700	5.01587100	7.33259600
H	2.34956700	6.70134300	7.30114900
C	6.17987700	3.00578200	7.29783200
H	5.29040500	2.35625300	7.33259600
H	5.83979000	4.04045900	7.30114900
C	5.65980600	-5.45401800	5.07923200
O	6.49808200	-4.54356200	5.14517400
C	1.37308800	-7.73914400	5.07923200
O	2.58642000	-7.49529600	5.14517400
C	7.78467100	-1.08564300	5.07923200
O	7.92769800	0.14365800	5.14517400
C	3.43810300	7.06817900	5.07923200
O	2.31316700	7.58408100	5.14517400
C	6.93605600	3.69741100	5.07923200
O	6.32920200	4.77600500	5.14517400
C	8.23103500	-1.86791300	3.85158900

H	7.93297600	-2.91771900	3.93637500
H	9.32956400	-1.83647500	3.83959900
C	5.56111500	-6.34925500	3.85158900
H	4.70292000	-7.02337000	3.93637500
H	6.46832300	-6.96952000	3.83959900
C	0.76703900	-8.40539600	3.85158900
H	-0.32349200	-8.44633300	3.93637500
H	1.13640200	-9.44044500	3.83959900
C	4.32002100	7.25096300	3.85158900
H	5.22634000	6.64308300	3.93637500
H	4.62958600	8.30544100	3.83959900
C	7.75697900	3.32690800	3.85158900
H	8.13290400	2.30240200	3.93637500
H	8.62722900	3.99804100	3.83959900
N	4.78642800	-5.73140800	6.08061100
N	0.50346500	-7.45019800	6.08061100
N	7.24113800	-1.82341400	6.08061100
N	3.97180500	6.32326600	6.08061100
N	6.92998000	2.78106100	6.08061100
H	4.04985300	-6.42124000	5.92316100
H	-0.49791000	-7.57533600	5.92316100
H	7.05071000	-2.81444800	5.92316100
H	4.85548900	5.83591100	5.92316100
H	7.35843500	1.86736700	5.92316100
N	6.87360900	3.61322000	9.54170500
N	7.68466400	-1.11705000	9.54170500
N	5.56043800	-5.42064400	9.54170500
N	1.31231400	-7.65373700	9.54170500
N	3.43706900	6.96336200	9.54170500
C	5.55029600	-6.01694800	10.86998300
H	6.52832700	-6.45897600	11.09651300
H	4.78687800	-6.79108800	10.89500300
C	8.02695700	-1.60543100	10.86998300
H	9.07801800	-1.38816700	11.09651300
H	7.86436700	-2.68044900	10.89500300

C	7.43759400	3.41930600	10.86998300
H	7.93793500	2.45403000	10.89500300
H	8.16021500	4.21287400	11.09651300
C	4.00732200	7.13798500	10.86998300
H	4.12548700	8.20474100	11.09651300
H	4.97948200	6.65115300	10.89500300
C	0.95361000	-8.13019600	10.86998300
H	1.48503700	-9.06267500	11.09651300
H	-0.11903600	-8.30776200	10.89500300
C	7.14841100	-0.90466200	11.90595000
O	6.29752300	-1.43726600	12.60305600
C	5.25143900	-4.93361700	11.90595000
O	4.25000000	-4.86436400	12.60305600
C	6.31493200	3.46984400	11.90595000
O	5.93960700	2.53881900	12.60305600
C	3.06936500	6.51898700	11.90595000
O	3.31296300	5.54516100	12.60305600
C	1.34859600	-7.07809800	11.90595000
O	0.57912100	-6.43344000	12.60305600
O	5.76787800	4.68001700	11.92386000
H	4.90816800	4.73623100	12.42162400
O	7.41715600	0.39594000	11.92386000
H	6.75467900	0.94674300	12.42162400
O	6.23333300	-4.03937300	11.92386000
H	6.02113100	-3.20437000	12.42162400
O	1.91546600	7.17648700	11.92386000
H	1.18690500	6.71664100	12.42162400
O	2.66858900	-6.93178300	11.92386000
H	2.98771600	-6.13152200	12.42162400
C	-5.49078900	5.53105700	2.55293100
C	-5.52744900	6.39662100	1.28773800
H	-6.32666400	4.82152400	2.54078500
H	-4.57110200	4.92886600	2.55558000
C	-5.46682900	5.56496800	0.00000000
H	-6.44493200	7.00379800	1.28796000

H	-4.69003400	7.10976400	1.30349400
H	-4.54464300	4.96521400	0.00000000
H	-6.29718400	4.84298100	0.00000000
C	-7.69321500	1.24731400	2.55293100
C	-8.23164000	1.92602200	1.28773800
H	-7.95240000	0.18197500	2.54078500
H	-6.59521400	1.30070900	2.55558000
H	-9.33078900	1.87795600	1.28796000
H	-7.97333200	2.99518700	1.30349400
C	-6.95709400	-3.51286000	2.55293100
C	-7.79162400	-3.28025200	1.28773800
H	-6.54058900	-4.52708200	2.54078500
H	-6.10017800	-2.82427300	2.55558000
C	-6.98194200	-3.47959300	0.00000000
H	-8.65260100	-3.96520200	1.28796000
H	-8.21108700	-2.26345000	1.30349400
H	-6.12657100	-2.78787700	0.00000000
H	-6.55188500	-4.49241400	0.00000000
C	-3.56360000	-6.93124100	2.55293100
C	-4.37547300	-7.23358100	1.28773800
H	-2.63049500	-7.50694800	2.54078500
H	-3.27508100	-5.87048000	2.55558000
C	-3.60325600	-6.91893300	0.00000000
H	-4.66941400	-8.29378700	1.28796000
H	-5.31248700	-6.65752500	1.30349400
H	-3.31782700	-5.85654700	0.00000000
H	-2.66001200	-7.48554100	0.00000000
C	1.19106800	-7.70212300	2.55293100
C	0.71196100	-8.42392800	1.28773800
H	2.28435800	-7.61941500	2.54078500
H	0.80098500	-6.67436300	2.55558000
C	1.15175100	-7.71547500	0.00000000
H	1.09733000	-9.45442800	1.28796000
H	-0.38469700	-8.50865300	1.30349400
H	0.75821400	-6.68821600	0.00000000

H	2.24789600	-7.61944600	0.00000000
C	5.49078900	-5.53105700	2.55293100
C	5.52744900	-6.39662100	1.28773800
H	6.32666400	-4.82152400	2.54078500
H	4.57110200	-4.92886600	2.55558000
H	6.44493200	-7.00379800	1.28796000
H	4.69003400	-7.10976400	1.30349400
C	7.69321500	-1.24731400	2.55293100
C	8.23164000	-1.92602200	1.28773800
H	7.95240000	-0.18197500	2.54078500
H	6.59521400	-1.30070900	2.55558000
H	9.33078900	-1.87795600	1.28796000
H	7.97333200	-2.99518700	1.30349400
C	6.95709400	3.51286000	2.55293100
C	7.79162400	3.28025200	1.28773800
H	6.54058900	4.52708200	2.54078500
H	6.10017800	2.82427300	2.55558000
H	8.65260100	3.96520200	1.28796000
H	8.21108700	2.26345000	1.30349400
C	3.56360000	6.93124100	2.55293100
C	4.37547300	7.23358100	1.28773800
H	2.63049500	7.50694800	2.54078500
H	3.27508100	5.87048000	2.55558000
H	4.66941400	8.29378700	1.28796000
H	5.31248700	6.65752500	1.30349400
C	-1.19106800	7.70212300	2.55293100
C	-0.71196100	8.42392800	1.28773800
H	-2.28435800	7.61941500	2.54078500
H	-0.80098500	6.67436300	2.55558000
H	-1.09733000	9.45442800	1.28796000
H	0.38469700	8.50865300	1.30349400
C	-4.72175600	5.81473500	-8.55660100
O	-3.93678800	6.76486400	-8.65662300
C	-0.40216600	7.47959800	-8.55660100
O	0.79135900	7.78687600	-8.65662300

C	-7.23779600	1.92884100	-8.55660100
O	-7.16121500	3.15890400	-8.65662300
C	-4.07103800	-6.28750800	-8.55660100
O	-5.21723300	-5.83456600	-8.65662300
C	-6.98924400	-2.69380500	-8.55660100
O	-7.65030200	-1.65365000	-8.65662300
H	-7.75361100	0.11416600	-9.36046300
H	-6.20569800	4.64982100	-9.36046300
H	-2.28741900	7.40940200	-9.36046300
H	-2.50457600	-7.33884300	-9.36046300
H	-6.33990800	-4.46509600	-9.36046300
C	-6.76637900	1.20071200	-7.29783200
H	-5.66499800	1.20337300	-7.33259600
H	-7.09941200	0.16374300	-7.30114900
C	-4.76835500	4.94857400	-7.29783200
H	-3.87575500	4.30335200	-7.33259600
H	-5.64729900	4.30540000	-7.30114900
C	-0.94898100	6.80625000	-7.29783200
H	-0.60610500	5.75959600	-7.33259600
H	-2.03811000	6.80254100	-7.30114900
C	-3.23287100	-6.06416900	-7.29783200
H	-2.89505700	-5.01587100	-7.33259600
H	-2.34956700	-6.70134300	-7.30114900
C	-6.17987700	-3.00578200	-7.29783200
H	-5.29040500	-2.35625300	-7.33259600
H	-5.83979000	-4.04045900	-7.30114900
C	-5.65980600	5.45401800	-5.07923200
O	-6.49808200	4.54356200	-5.14517400
C	-1.37308800	7.73914400	-5.07923200
O	-2.58642000	7.49529600	-5.14517400
C	-7.78467100	1.08564300	-5.07923200
O	-7.92769800	-0.14365800	-5.14517400
C	-3.43810300	-7.06817900	-5.07923200
O	-2.31316700	-7.58408100	-5.14517400
C	-6.93605600	-3.69741100	-5.07923200

O	-6.32920200	-4.77600500	-5.14517400
C	-8.23103500	1.86791300	-3.85158900
H	-7.93297600	2.91771900	-3.93637500
H	-9.32956400	1.83647500	-3.83959900
C	-5.56111500	6.34925500	-3.85158900
H	-4.70292000	7.02337000	-3.93637500
H	-6.46832300	6.96952000	-3.83959900
C	-0.76703900	8.40539600	-3.85158900
H	0.32349200	8.44633300	-3.93637500
H	-1.13640200	9.44044500	-3.83959900
C	-4.32002100	-7.25096300	-3.85158900
H	-5.22634000	-6.64308300	-3.93637500
H	-4.62958600	-8.30544100	-3.83959900
C	-7.75697900	-3.32690800	-3.85158900
H	-8.13290400	-2.30240200	-3.93637500
H	-8.62722900	-3.99804100	-3.83959900
N	-4.78642800	5.73140800	-6.08061100
N	-0.50346500	7.45019800	-6.08061100
N	-7.24113800	1.82341400	-6.08061100
N	-3.97180500	-6.32326600	-6.08061100
N	-6.92998000	-2.78106100	-6.08061100
H	-4.04985300	6.42124000	-5.92316100
H	0.49791000	7.57533600	-5.92316100
H	-7.05071000	2.81444800	-5.92316100
H	-4.85548900	-5.83591100	-5.92316100
H	-7.35843500	-1.86736700	-5.92316100
N	-6.87360900	-3.61322000	-9.54170500
N	-7.68466400	1.11705000	-9.54170500
N	-5.56043800	5.42064400	-9.54170500
N	-1.31231400	7.65373700	-9.54170500
N	-3.43706900	-6.96336200	-9.54170500
C	-5.55029600	6.01694800	-10.86998300
H	-6.52832700	6.45897600	-11.09651300
H	-4.78687800	6.79108800	-10.89500300
C	-8.02695700	1.60543100	-10.86998300

H	-9.07801800	1.38816700	-11.09651300
H	-7.86436700	2.68044900	-10.89500300
C	-7.43759400	-3.41930600	-10.86998300
H	-7.93793500	-2.45403000	-10.89500300
H	-8.16021500	-4.21287400	-11.09651300
C	-4.00732200	-7.13798500	-10.86998300
H	-4.12548700	-8.20474100	-11.09651300
H	-4.97948200	-6.65115300	-10.89500300
C	-0.95361000	8.13019600	-10.86998300
H	-1.48503700	9.06267500	-11.09651300
H	0.11903600	8.30776200	-10.89500300
C	-7.14841100	0.90466200	-11.90595000
O	-6.29752300	1.43726600	-12.60305600
C	-5.25143900	4.93361700	-11.90595000
O	-4.25000000	4.86436400	-12.60305600
C	-6.31493200	-3.46984400	-11.90595000
O	-5.93960700	-2.53881900	-12.60305600
C	-3.06936500	-6.51898700	-11.90595000
O	-3.31296300	-5.54516100	-12.60305600
C	-1.34859600	7.07809800	-11.90595000
O	-0.57912100	6.43344000	-12.60305600
O	-5.76787800	-4.68001700	-11.92386000
H	-4.90816800	-4.73623100	-12.42162400
O	-7.41715600	-0.39594000	-11.92386000
H	-6.75467900	-0.94674300	-12.42162400
O	-6.23333300	4.03937300	-11.92386000
H	-6.02113100	3.20437000	-12.42162400
O	-1.91546600	-7.17648700	-11.92386000
H	-1.18690500	-6.71664100	-12.42162400
O	-2.66858900	6.93178300	-11.92386000
H	-2.98771600	6.13152200	-12.42162400
C	4.72175600	-5.81473500	-8.55660100
O	3.93678800	-6.76486400	-8.65662300
C	0.40216600	-7.47959800	-8.55660100
O	-0.79135900	-7.78687600	-8.65662300

C	7.23779600	-1.92884100	-8.55660100
O	7.16121500	-3.15890400	-8.65662300
C	4.07103800	6.28750800	-8.55660100
O	5.21723300	5.83456600	-8.65662300
C	6.98924400	2.69380500	-8.55660100
O	7.65030200	1.65365000	-8.65662300
H	7.75361100	-0.11416600	-9.36046300
H	6.20569800	-4.64982100	-9.36046300
H	2.28741900	-7.40940200	-9.36046300
H	2.50457600	7.33884300	-9.36046300
H	6.33990800	4.46509600	-9.36046300
C	6.76637900	-1.20071200	-7.29783200
H	5.66499800	-1.20337300	-7.33259600
H	7.09941200	-0.16374300	-7.30114900
C	4.76835500	-4.94857400	-7.29783200
H	3.87575500	-4.30335200	-7.33259600
H	5.64729900	-4.30540000	-7.30114900
C	0.94898100	-6.80625000	-7.29783200
H	0.60610500	-5.75959600	-7.33259600
H	2.03811000	-6.80254100	-7.30114900
C	3.23287100	6.06416900	-7.29783200
H	2.89505700	5.01587100	-7.33259600
H	2.34956700	6.70134300	-7.30114900
C	6.17987700	3.00578200	-7.29783200
H	5.29040500	2.35625300	-7.33259600
H	5.83979000	4.04045900	-7.30114900
C	5.65980600	-5.45401800	-5.07923200
O	6.49808200	-4.54356200	-5.14517400
C	1.37308800	-7.73914400	-5.07923200
O	2.58642000	-7.49529600	-5.14517400
C	7.78467100	-1.08564300	-5.07923200
O	7.92769800	0.14365800	-5.14517400
C	3.43810300	7.06817900	-5.07923200
O	2.31316700	7.58408100	-5.14517400
C	6.93605600	3.69741100	-5.07923200

O	6.32920200	4.77600500	-5.14517400
C	8.23103500	-1.86791300	-3.85158900
H	7.93297600	-2.91771900	-3.93637500
H	9.32956400	-1.83647500	-3.83959900
C	5.56111500	-6.34925500	-3.85158900
H	4.70292000	-7.02337000	-3.93637500
H	6.46832300	-6.96952000	-3.83959900
C	0.76703900	-8.40539600	-3.85158900
H	-0.32349200	-8.44633300	-3.93637500
H	1.13640200	-9.44044500	-3.83959900
C	4.32002100	7.25096300	-3.85158900
H	5.22634000	6.64308300	-3.93637500
H	4.62958600	8.30544100	-3.83959900
C	7.75697900	3.32690800	-3.85158900
H	8.13290400	2.30240200	-3.93637500
H	8.62722900	3.99804100	-3.83959900
N	4.78642800	-5.73140800	-6.08061100
N	0.50346500	-7.45019800	-6.08061100
N	7.24113800	-1.82341400	-6.08061100
N	3.97180500	6.32326600	-6.08061100
N	6.92998000	2.78106100	-6.08061100
H	4.04985300	-6.42124000	-5.92316100
H	-0.49791000	-7.57533600	-5.92316100
H	7.05071000	-2.81444800	-5.92316100
H	4.85548900	5.83591100	-5.92316100
H	7.35843500	1.86736700	-5.92316100
N	6.87360900	3.61322000	-9.54170500
N	7.68466400	-1.11705000	-9.54170500
N	5.56043800	-5.42064400	-9.54170500
N	1.31231400	-7.65373700	-9.54170500
N	3.43706900	6.96336200	-9.54170500
C	5.55029600	-6.01694800	-10.86998300
H	6.52832700	-6.45897600	-11.09651300
H	4.78687800	-6.79108800	-10.89500300
C	8.02695700	-1.60543100	-10.86998300

H	9.07801800	-1.38816700	-11.09651300
H	7.86436700	-2.68044900	-10.89500300
C	7.43759400	3.41930600	-10.86998300
H	7.93793500	2.45403000	-10.89500300
H	8.16021500	4.21287400	-11.09651300
C	4.00732200	7.13798500	-10.86998300
H	4.12548700	8.20474100	-11.09651300
H	4.97948200	6.65115300	-10.89500300
C	0.95361000	-8.13019600	-10.86998300
H	1.48503700	-9.06267500	-11.09651300
H	-0.11903600	-8.30776200	-10.89500300
C	7.14841100	-0.90466200	-11.90595000
O	6.29752300	-1.43726600	-12.60305600
C	5.25143900	-4.93361700	-11.90595000
O	4.25000000	-4.86436400	-12.60305600
C	6.31493200	3.46984400	-11.90595000
O	5.93960700	2.53881900	-12.60305600
C	3.06936500	6.51898700	-11.90595000
O	3.31296300	5.54516100	-12.60305600
C	1.34859600	-7.07809800	-11.90595000
O	0.57912100	-6.43344000	-12.60305600
O	5.76787800	4.68001700	-11.92386000
H	4.90816800	4.73623100	-12.42162400
O	7.41715600	0.39594000	-11.92386000
H	6.75467900	0.94674300	-12.42162400
O	6.23333300	-4.03937300	-11.92386000
H	6.02113100	-3.20437000	-12.42162400
O	1.91546600	7.17648700	-11.92386000
H	1.18690500	6.71664100	-12.42162400
O	2.66858900	-6.93178300	-11.92386000
H	2.98771600	-6.13152200	-12.42162400
C	-5.49078900	5.53105700	-2.55293100
C	-5.52744900	6.39662100	-1.28773800
H	-6.32666400	4.82152400	-2.54078500
H	-4.57110200	4.92886600	-2.55558000

H	-6.44493200	7.00379800	-1.28796000
H	-4.69003400	7.10976400	-1.30349400
C	-7.69321500	1.24731400	-2.55293100
C	-8.23164000	1.92602200	-1.28773800
H	-7.95240000	0.18197500	-2.54078500
H	-6.59521400	1.30070900	-2.55558000
H	-9.33078900	1.87795600	-1.28796000
H	-7.97333200	2.99518700	-1.30349400
C	-6.95709400	-3.51286000	-2.55293100
C	-7.79162400	-3.28025200	-1.28773800
H	-6.54058900	-4.52708200	-2.54078500
H	-6.10017800	-2.82427300	-2.55558000
H	-8.65260100	-3.96520200	-1.28796000
H	-8.21108700	-2.26345000	-1.30349400
C	-3.56360000	-6.93124100	-2.55293100
C	-4.37547300	-7.23358100	-1.28773800
H	-2.63049500	-7.50694800	-2.54078500
H	-3.27508100	-5.87048000	-2.55558000
H	-4.66941400	-8.29378700	-1.28796000
H	-5.31248700	-6.65752500	-1.30349400
C	1.19106800	-7.70212300	-2.55293100
C	0.71196100	-8.42392800	-1.28773800
H	2.28435800	-7.61941500	-2.54078500
H	0.80098500	-6.67436300	-2.55558000
H	1.09733000	-9.45442800	-1.28796000
H	-0.38469700	-8.50865300	-1.30349400
C	5.49078900	-5.53105700	-2.55293100
C	5.52744900	-6.39662100	-1.28773800
H	6.32666400	-4.82152400	-2.54078500
H	4.57110200	-4.92886600	-2.55558000
C	5.46682900	-5.56496800	0.00000000
H	6.44493200	-7.00379800	-1.28796000
H	4.69003400	-7.10976400	-1.30349400
H	4.54464300	-4.96521400	0.00000000
H	6.29718400	-4.84298100	0.00000000

C	7.69321500	-1.24731400	-2.55293100
C	8.23164000	-1.92602200	-1.28773800
H	7.95240000	-0.18197500	-2.54078500
H	6.59521400	-1.30070900	-2.55558000
H	9.33078900	-1.87795600	-1.28796000
H	7.97333200	-2.99518700	-1.30349400
C	6.95709400	3.51286000	-2.55293100
C	7.79162400	3.28025200	-1.28773800
H	6.54058900	4.52708200	-2.54078500
H	6.10017800	2.82427300	-2.55558000
C	6.98194200	3.47959300	0.00000000
H	8.65260100	3.96520200	-1.28796000
H	8.21108700	2.26345000	-1.30349400
H	6.12657100	2.78787700	0.00000000
H	6.55188500	4.49241400	0.00000000
C	3.56360000	6.93124100	-2.55293100
C	4.37547300	7.23358100	-1.28773800
H	2.63049500	7.50694800	-2.54078500
H	3.27508100	5.87048000	-2.55558000
C	3.60325600	6.91893300	0.00000000
H	4.66941400	8.29378700	-1.28796000
H	5.31248700	6.65752500	-1.30349400
H	3.31782700	5.85654700	0.00000000
H	2.66001200	7.48554100	0.00000000
C	-1.19106800	7.70212300	-2.55293100
C	-0.71196100	8.42392800	-1.28773800
H	-2.28435800	7.61941500	-2.54078500
H	-0.80098500	6.67436300	-2.55558000
C	-1.15175100	7.71547500	0.00000000
H	-1.09733000	9.45442800	-1.28796000
H	0.38469700	8.50865300	-1.30349400
H	-0.75821400	6.68821600	0.00000000
H	-2.24789600	7.61944600	0.00000000
C	7.69376300	-1.28883300	0.00000000
H	6.59517300	-1.34566800	0.00000000

H	7.94116100	-0.21666200	0.00000000
C	-7.69376300	1.28883300	0.00000000
H	-7.94116100	0.21666200	0.00000000
H	-6.59517300	1.34566800	0.00000000

$[P\text{-}(CH_2)_7\text{-}P]_{12}$

C	0.72841500	8.97975800	8.54209100
O	1.95421500	9.11103900	8.63789600
C	-3.85905300	8.14090600	8.54209100
O	-2.86312000	8.86749900	8.63789600
H	-5.57900700	7.37679100	9.35508300
H	-1.14316600	9.17799100	9.35508300
C	-4.13285300	7.33144200	7.27496000
H	-3.42508600	6.48712200	7.28874500
H	-5.14253000	6.92296900	7.28322900
C	0.08656500	8.41564100	7.27496000
H	0.27735000	7.33055500	7.28874500
H	-0.99207700	8.56673200	7.28322900
C	-0.20315400	9.43971400	5.07475300
O	-1.43881700	9.37030000	5.14066300
C	-4.89579300	8.07345500	5.07475300
O	-5.93120200	7.39550900	5.14066300
C	-4.59064900	8.92900000	3.85286900
H	-3.60413900	9.39401500	3.94524000
H	-5.33749600	9.73493400	3.83697600
C	0.48888200	10.02806600	3.85286900
H	1.57573100	9.93752500	3.94524000
H	0.24506000	11.09944800	3.83697600
N	0.61742200	9.01255900	6.06777100
N	-3.97157600	8.11381600	6.06777100
H	1.62695100	9.00584800	5.91360400
H	-3.09394300	8.61276900	5.91360400
N	-4.75376000	7.95050500	9.53767900
N	-0.14162500	9.26221900	9.53767900
C	0.28825500	9.65887100	10.87077900
H	-0.09623900	10.65709900	11.11393600

H	1.37506000	9.67218000	10.89478900
C	-4.57979900	8.50895600	10.87077900
H	-5.41189500	9.18119900	11.11393600
H	-3.64525300	9.06388400	10.89478900
C	-4.55461600	7.37353300	11.89399300
O	-3.59778900	7.04032300	12.57564600
C	-0.25764600	8.66297500	11.89399300
O	0.40438500	7.89599300	12.57564600
O	-5.73689900	6.76453000	11.91767300
H	-5.75418300	5.90551200	12.41408500
O	-1.58603500	8.72670400	11.91767300
H	-2.03051200	7.99141500	12.41408500
C	-0.00804000	9.37715000	2.55223100
C	0.55785400	10.03515200	1.28808400
H	-1.10346500	9.42333300	2.53471300
H	0.25861000	8.31069800	2.55826300
C	0.03825600	9.38358200	0.00000000
H	0.29902800	11.10448900	1.28685500
H	1.65669400	9.98746200	1.30649000
H	0.30742000	8.31696400	0.00000000
H	-1.06155000	9.41768700	0.00000000
C	-4.69553800	8.11683100	2.55223100
C	-4.53446000	8.96962300	1.28808400
H	-5.66729600	7.60911300	2.53471300
H	-3.93138600	7.32658100	2.55826300
H	-5.29327800	9.76628400	1.28685500
H	-3.55899200	9.47774300	1.30649000
C	0.72841500	8.97975800	-8.54209100
O	1.95421500	9.11103900	-8.63789600
C	-3.85905300	8.14090600	-8.54209100
O	-2.86312000	8.86749900	-8.63789600
H	-5.57900700	7.37679100	-9.35508300
H	-1.14316600	9.17799100	-9.35508300
C	-4.13285300	7.33144200	-7.27496000
H	-3.42508600	6.48712200	-7.28874500

H	-5.14253000	6.92296900	-7.28322900
C	0.08656500	8.41564100	-7.27496000
H	0.27735000	7.33055500	-7.28874500
H	-0.99207700	8.56673200	-7.28322900
C	-0.20315400	9.43971400	-5.07475300
O	-1.43881700	9.37030000	-5.14066300
C	-4.89579300	8.07345500	-5.07475300
O	-5.93120200	7.39550900	-5.14066300
C	-4.59064900	8.92900000	-3.85286900
H	-3.60413900	9.39401500	-3.94524000
H	-5.33749600	9.73493400	-3.83697600
C	0.48888200	10.02806600	-3.85286900
H	1.57573100	9.93752500	-3.94524000
H	0.24506000	11.09944800	-3.83697600
N	0.61742200	9.01255900	-6.06777100
N	-3.97157600	8.11381600	-6.06777100
H	1.62695100	9.00584800	-5.91360400
H	-3.09394300	8.61276900	-5.91360400
N	-4.75376000	7.95050500	-9.53767900
N	-0.14162500	9.26221900	-9.53767900
C	0.28825500	9.65887100	-10.87077900
H	-0.09623900	10.65709900	-11.11393600
H	1.37506000	9.67218000	-10.89478900
C	-4.57979900	8.50895600	-10.87077900
H	-5.41189500	9.18119900	-11.11393600
H	-3.64525300	9.06388400	-10.89478900
C	-4.55461600	7.37353300	-11.89399300
O	-3.59778900	7.04032300	-12.57564600
C	-0.25764600	8.66297500	-11.89399300
O	0.40438500	7.89599300	-12.57564600
O	-5.73689900	6.76453000	-11.91767300
H	-5.75418300	5.90551200	-12.41408500
O	-1.58603500	8.72670400	-11.91767300
H	-2.03051200	7.99141500	-12.41408500
C	-0.00804000	9.37715000	-2.55223100

C	0.55785400	10.03515200	-1.28808400
H	-1.10346500	9.42333300	-2.53471300
H	0.25861000	8.31069800	-2.55826300
H	0.29902800	11.10448900	-1.28685500
H	1.65669400	9.98746200	-1.30649000
C	-4.69553800	8.11683100	-2.55223100
C	-4.53446000	8.96962300	-1.28808400
H	-5.66729600	7.60911300	-2.53471300
H	-3.93138600	7.32658100	-2.55826300
H	-5.29327800	9.76628400	-1.28685500
H	-3.55899200	9.47774300	-1.30649000
C	-4.65866000	8.14554900	0.00000000
H	-5.62817300	7.62518100	0.00000000
H	-3.89224900	7.35641200	0.00000000
C	-7.41249100	5.12070500	8.54209100
O	-6.91328400	6.24791900	8.63789600
C	-8.97975800	0.72841500	8.54209100
O	-9.11103900	1.95421500	8.63789600
H	-9.17799100	-1.14316600	9.35508300
H	-8.51995700	3.59898500	9.35508300
C	-8.41564100	0.08656500	7.27496000
H	-7.33055500	0.27735000	7.28874500
H	-8.56673200	-0.99207700	7.28322900
C	-7.24487600	4.28278800	7.27496000
H	-6.20977200	3.90546900	7.28874500
H	-7.91504600	3.42420200	7.28322900
C	-8.27660900	4.54392100	5.07475300
O	-8.83432700	3.43909800	5.14066300
C	-9.43971400	-0.20315400	5.07475300
O	-9.37030000	-1.43881700	5.14066300
C	-10.02806600	0.48888200	3.85286900
H	-9.93752500	1.57573100	3.94524000
H	-11.09944800	0.24506000	3.83697600
C	-8.44011900	5.43741700	3.85286900
H	-7.81828400	6.33338600	3.94524000

H	-9.48987400	5.76195300	3.83697600
N	-7.49639400	5.04098300	6.06777100
N	-9.01255900	0.61742200	6.06777100
H	-6.98581800	5.91190500	5.91360400
H	-9.00584800	1.62695100	5.91360400
N	-9.26221900	-0.14162500	9.53767900
N	-8.09212900	4.50845900	9.53767900
C	-8.22070000	5.07907200	10.87077900
H	-9.27743800	5.24520400	11.11393600
H	-7.68882300	6.02692700	10.89478900
C	-9.65887100	0.28825500	10.87077900
H	-10.65709900	-0.09623900	11.11393600
H	-9.67218000	1.37506000	10.89478900
C	-8.66297500	-0.25764600	11.89399300
O	-7.89599300	0.40438500	12.57564600
C	-7.63117900	4.10835900	11.89399300
O	-6.63593800	4.29820400	12.57564600
O	-8.72670400	-1.58603500	11.91767300
H	-7.99141500	-2.03051200	12.41408500
O	-8.35056500	2.98980600	11.91767300
H	-7.93602400	2.23723200	12.41408500
C	-8.12487000	4.68161300	2.55223100
C	-8.41177000	5.50069100	1.28808400
H	-8.71257900	3.75603800	2.53471300
H	-7.06797100	4.37931200	2.55826300
C	-8.10729300	4.72492200	0.00000000
H	-9.46725500	5.81121100	1.28685500
H	-7.82104900	6.42847000	1.30649000
H	-7.04899200	4.42471600	0.00000000
H	-8.68673100	3.78951400	0.00000000
C	-9.37715000	-0.00804000	2.55223100
C	-10.03515200	0.55785400	1.28808400
H	-9.42333300	-1.10346500	2.53471300
H	-8.31069800	0.25861000	2.55826300
H	-11.10448900	0.29902800	1.28685500

H	-9.98746200	1.65669400	1.30649000
C	-7.41249100	5.12070500	-8.54209100
O	-6.91328400	6.24791900	-8.63789600
C	-8.97975800	0.72841500	-8.54209100
O	-9.11103900	1.95421500	-8.63789600
H	-9.17799100	-1.14316600	-9.35508300
H	-8.51995700	3.59898500	-9.35508300
C	-8.41564100	0.08656500	-7.27496000
H	-7.33055500	0.27735000	-7.28874500
H	-8.56673200	-0.99207700	-7.28322900
C	-7.24487600	4.28278800	-7.27496000
H	-6.20977200	3.90546900	-7.28874500
H	-7.91504600	3.42420200	-7.28322900
C	-8.27660900	4.54392100	-5.07475300
O	-8.83432700	3.43909800	-5.14066300
C	-9.43971400	-0.20315400	-5.07475300
O	-9.37030000	-1.43881700	-5.14066300
C	-10.02806600	0.48888200	-3.85286900
H	-9.93752500	1.57573100	-3.94524000
H	-11.09944800	0.24506000	-3.83697600
C	-8.44011900	5.43741700	-3.85286900
H	-7.81828400	6.33338600	-3.94524000
H	-9.48987400	5.76195300	-3.83697600
N	-7.49639400	5.04098300	-6.06777100
N	-9.01255900	0.61742200	-6.06777100
H	-6.98581800	5.91190500	-5.91360400
H	-9.00584800	1.62695100	-5.91360400
N	-9.26221900	-0.14162500	-9.53767900
N	-8.09212900	4.50845900	-9.53767900
C	-8.22070000	5.07907200	-10.87077900
H	-9.27743800	5.24520400	-11.11393600
H	-7.68882300	6.02692700	-10.89478900
C	-9.65887100	0.28825500	-10.87077900
H	-10.65709900	-0.09623900	-11.11393600
H	-9.67218000	1.37506000	-10.89478900

C	-8.66297500	-0.25764600	-11.89399300
O	-7.89599300	0.40438500	-12.57564600
C	-7.63117900	4.10835900	-11.89399300
O	-6.63593800	4.29820400	-12.57564600
O	-8.72670400	-1.58603500	-11.91767300
H	-7.99141500	-2.03051200	-12.41408500
O	-8.35056500	2.98980600	-11.91767300
H	-7.93602400	2.23723200	-12.41408500
C	-8.12487000	4.68161300	-2.55223100
C	-8.41177000	5.50069100	-1.28808400
H	-8.71257900	3.75603800	-2.53471300
H	-7.06797100	4.37931200	-2.55826300
H	-9.46725500	5.81121100	-1.28685500
H	-7.82104900	6.42847000	-1.30649000
C	-9.37715000	-0.00804000	-2.55223100
C	-10.03515200	0.55785400	-1.28808400
H	-9.42333300	-1.10346500	-2.53471300
H	-8.31069800	0.25861000	-2.55826300
H	-11.10448900	0.29902800	-1.28685500
H	-9.98746200	1.65669400	-1.30649000
C	-9.38358200	0.03825600	0.00000000
H	-9.41768700	-1.06155000	0.00000000
H	-8.31696400	0.30742000	0.00000000
C	-8.14090600	-3.85905300	8.54209100
O	-8.86749900	-2.86312000	8.63789600
C	-5.12070500	-7.41249100	8.54209100
O	-6.24791900	-6.91328400	8.63789600
H	-3.59898500	-8.51995700	9.35508300
H	-7.37679100	-5.57900700	9.35508300
C	-4.28278800	-7.24487600	7.27496000
H	-3.90546900	-6.20977200	7.28874500
H	-3.42420200	-7.91504600	7.28322900
C	-7.33144200	-4.13285300	7.27496000
H	-6.48712200	-3.42508600	7.28874500
H	-6.92296900	-5.14253000	7.28322900

C	-8.07345500	-4.89579300	5.07475300
O	-7.39550900	-5.93120200	5.14066300
C	-4.54392100	-8.27660900	5.07475300
O	-3.43909800	-8.83432700	5.14066300
C	-5.43741700	-8.44011900	3.85286900
H	-6.33338600	-7.81828400	3.94524000
H	-5.76195300	-9.48987400	3.83697600
C	-8.92900000	-4.59064900	3.85286900
H	-9.39401500	-3.60413900	3.94524000
H	-9.73493400	-5.33749600	3.83697600
N	-8.11381600	-3.97157600	6.06777100
N	-5.04098300	-7.49639400	6.06777100
H	-8.61276900	-3.09394300	5.91360400
H	-5.91190500	-6.98581800	5.91360400
N	-4.50845900	-8.09212900	9.53767900
N	-7.95050500	-4.75376000	9.53767900
C	-8.50895600	-4.57979900	10.87077900
H	-9.18119900	-5.41189500	11.11393600
H	-9.06388400	-3.64525300	10.89478900
C	-5.07907200	-8.22070000	10.87077900
H	-5.24520400	-9.27743800	11.11393600
H	-6.02692700	-7.68882300	10.89478900
C	-4.10835900	-7.63117900	11.89399300
O	-4.29820400	-6.63593800	12.57564600
C	-7.37353300	-4.55461600	11.89399300
O	-7.04032300	-3.59778900	12.57564600
O	-2.98980600	-8.35056500	11.91767300
H	-2.23723200	-7.93602400	12.41408500
O	-6.76453000	-5.73689900	11.91767300
H	-5.90551200	-5.75418300	12.41408500
C	-8.11683100	-4.69553800	2.55223100
C	-8.96962300	-4.53446000	1.28808400
H	-7.60911300	-5.66729600	2.53471300
H	-7.32658100	-3.93138600	2.55826300
C	-8.14554900	-4.65866000	0.00000000

H	-9.76628400	-5.29327800	1.28685500
H	-9.47774300	-3.55899200	1.30649000
H	-7.35641200	-3.89224900	0.00000000
H	-7.62518100	-5.62817300	0.00000000
C	-4.68161300	-8.12487000	2.55223100
C	-5.50069100	-8.41177000	1.28808400
H	-3.75603800	-8.71257900	2.53471300
H	-4.37931200	-7.06797100	2.55826300
H	-5.81121100	-9.46725500	1.28685500
H	-6.42847000	-7.82104900	1.30649000
C	-8.14090600	-3.85905300	-8.54209100
O	-8.86749900	-2.86312000	-8.63789600
C	-5.12070500	-7.41249100	-8.54209100
O	-6.24791900	-6.91328400	-8.63789600
H	-3.59898500	-8.51995700	-9.35508300
H	-7.37679100	-5.57900700	-9.35508300
C	-4.28278800	-7.24487600	-7.27496000
H	-3.90546900	-6.20977200	-7.28874500
H	-3.42420200	-7.91504600	-7.28322900
C	-7.33144200	-4.13285300	-7.27496000
H	-6.48712200	-3.42508600	-7.28874500
H	-6.92296900	-5.14253000	-7.28322900
C	-8.07345500	-4.89579300	-5.07475300
O	-7.39550900	-5.93120200	-5.14066300
C	-4.54392100	-8.27660900	-5.07475300
O	-3.43909800	-8.83432700	-5.14066300
C	-5.43741700	-8.44011900	-3.85286900
H	-6.33338600	-7.81828400	-3.94524000
H	-5.76195300	-9.48987400	-3.83697600
C	-8.92900000	-4.59064900	-3.85286900
H	-9.39401500	-3.60413900	-3.94524000
H	-9.73493400	-5.33749600	-3.83697600
N	-8.11381600	-3.97157600	-6.06777100
N	-5.04098300	-7.49639400	-6.06777100
H	-8.61276900	-3.09394300	-5.91360400

H	-5.91190500	-6.98581800	-5.91360400
N	-4.50845900	-8.09212900	-9.53767900
N	-7.95050500	-4.75376000	-9.53767900
C	-8.50895600	-4.57979900	-10.87077900
H	-9.18119900	-5.41189500	-11.11393600
H	-9.06388400	-3.64525300	-10.89478900
C	-5.07907200	-8.22070000	-10.87077900
H	-5.24520400	-9.27743800	-11.11393600
H	-6.02692700	-7.68882300	-10.89478900
C	-4.10835900	-7.63117900	-11.89399300
O	-4.29820400	-6.63593800	-12.57564600
C	-7.37353300	-4.55461600	-11.89399300
O	-7.04032300	-3.59778900	-12.57564600
O	-2.98980600	-8.35056500	-11.91767300
H	-2.23723200	-7.93602400	-12.41408500
O	-6.76453000	-5.73689900	-11.91767300
H	-5.90551200	-5.75418300	-12.41408500
C	-8.11683100	-4.69553800	-2.55223100
C	-8.96962300	-4.53446000	-1.28808400
H	-7.60911300	-5.66729600	-2.53471300
H	-7.32658100	-3.93138600	-2.55826300
H	-9.76628400	-5.29327800	-1.28685500
H	-9.47774300	-3.55899200	-1.30649000
C	-4.68161300	-8.12487000	-2.55223100
C	-5.50069100	-8.41177000	-1.28808400
H	-3.75603800	-8.71257900	-2.53471300
H	-4.37931200	-7.06797100	-2.55826300
H	-5.81121100	-9.46725500	-1.28685500
H	-6.42847000	-7.82104900	-1.30649000
C	-4.72492200	-8.10729300	0.00000000
H	-3.78951400	-8.68673100	0.00000000
H	-4.42471600	-7.04899200	0.00000000
C	-0.72841500	-8.97975800	8.54209100
O	-1.95421500	-9.11103900	8.63789600
C	3.85905300	-8.14090600	8.54209100

O	2.86312000	-8.86749900	8.63789600
H	5.57900700	-7.37679100	9.35508300
H	1.14316600	-9.17799100	9.35508300
C	4.13285300	-7.33144200	7.27496000
H	3.42508600	-6.48712200	7.28874500
H	5.14253000	-6.92296900	7.28322900
C	-0.08656500	-8.41564100	7.27496000
H	-0.27735000	-7.33055500	7.28874500
H	0.99207700	-8.56673200	7.28322900
C	0.20315400	-9.43971400	5.07475300
O	1.43881700	-9.37030000	5.14066300
C	4.89579300	-8.07345500	5.07475300
O	5.93120200	-7.39550900	5.14066300
C	4.59064900	-8.92900000	3.85286900
H	3.60413900	-9.39401500	3.94524000
H	5.33749600	-9.73493400	3.83697600
C	-0.48888200	-10.02806600	3.85286900
H	-1.57573100	-9.93752500	3.94524000
H	-0.24506000	-11.09944800	3.83697600
N	-0.61742200	-9.01255900	6.06777100
N	3.97157600	-8.11381600	6.06777100
H	-1.62695100	-9.00584800	5.91360400
H	3.09394300	-8.61276900	5.91360400
N	4.75376000	-7.95050500	9.53767900
N	0.14162500	-9.26221900	9.53767900
C	-0.28825500	-9.65887100	10.87077900
H	0.09623900	-10.65709900	11.11393600
H	-1.37506000	-9.67218000	10.89478900
C	4.57979900	-8.50895600	10.87077900
H	5.41189500	-9.18119900	11.11393600
H	3.64525300	-9.06388400	10.89478900
C	4.55461600	-7.37353300	11.89399300
O	3.59778900	-7.04032300	12.57564600
C	0.25764600	-8.66297500	11.89399300
O	-0.40438500	-7.89599300	12.57564600

O	5.73689900	-6.76453000	11.91767300
H	5.75418300	-5.90551200	12.41408500
O	1.58603500	-8.72670400	11.91767300
H	2.03051200	-7.99141500	12.41408500
C	0.00804000	-9.37715000	2.55223100
C	-0.55785400	-10.03515200	1.28808400
H	1.10346500	-9.42333300	2.53471300
H	-0.25861000	-8.31069800	2.55826300
C	-0.03825600	-9.38358200	0.00000000
H	-0.29902800	-11.10448900	1.28685500
H	-1.65669400	-9.98746200	1.30649000
H	-0.30742000	-8.31696400	0.00000000
H	1.06155000	-9.41768700	0.00000000
C	4.69553800	-8.11683100	2.55223100
C	4.53446000	-8.96962300	1.28808400
H	5.66729600	-7.60911300	2.53471300
H	3.93138600	-7.32658100	2.55826300
H	5.29327800	-9.76628400	1.28685500
H	3.55899200	-9.47774300	1.30649000
C	-0.72841500	-8.97975800	-8.54209100
O	-1.95421500	-9.11103900	-8.63789600
C	3.85905300	-8.14090600	-8.54209100
O	2.86312000	-8.86749900	-8.63789600
H	5.57900700	-7.37679100	-9.35508300
H	1.14316600	-9.17799100	-9.35508300
C	4.13285300	-7.33144200	-7.27496000
H	3.42508600	-6.48712200	-7.28874500
H	5.14253000	-6.92296900	-7.28322900
C	-0.08656500	-8.41564100	-7.27496000
H	-0.27735000	-7.33055500	-7.28874500
H	0.99207700	-8.56673200	-7.28322900
C	0.20315400	-9.43971400	-5.07475300
O	1.43881700	-9.37030000	-5.14066300
C	4.89579300	-8.07345500	-5.07475300
O	5.93120200	-7.39550900	-5.14066300

C	4.59064900	-8.92900000	-3.85286900
H	3.60413900	-9.39401500	-3.94524000
H	5.33749600	-9.73493400	-3.83697600
C	-0.48888200	-10.02806600	-3.85286900
H	-1.57573100	-9.93752500	-3.94524000
H	-0.24506000	-11.09944800	-3.83697600
N	-0.61742200	-9.01255900	-6.06777100
N	3.97157600	-8.11381600	-6.06777100
H	-1.62695100	-9.00584800	-5.91360400
H	3.09394300	-8.61276900	-5.91360400
N	4.75376000	-7.95050500	-9.53767900
N	0.14162500	-9.26221900	-9.53767900
C	-0.28825500	-9.65887100	-10.87077900
H	0.09623900	-10.65709900	-11.11393600
H	-1.37506000	-9.67218000	-10.89478900
C	4.57979900	-8.50895600	-10.87077900
H	5.41189500	-9.18119900	-11.11393600
H	3.64525300	-9.06388400	-10.89478900
C	4.55461600	-7.37353300	-11.89399300
O	3.59778900	-7.04032300	-12.57564600
C	0.25764600	-8.66297500	-11.89399300
O	-0.40438500	-7.89599300	-12.57564600
O	5.73689900	-6.76453000	-11.91767300
H	5.75418300	-5.90551200	-12.41408500
O	1.58603500	-8.72670400	-11.91767300
H	2.03051200	-7.99141500	-12.41408500
C	0.00804000	-9.37715000	-2.55223100
C	-0.55785400	-10.03515200	-1.28808400
H	1.10346500	-9.42333300	-2.53471300
H	-0.25861000	-8.31069800	-2.55826300
H	-0.29902800	-11.10448900	-1.28685500
H	-1.65669400	-9.98746200	-1.30649000
C	4.69553800	-8.11683100	-2.55223100
C	4.53446000	-8.96962300	-1.28808400
H	5.66729600	-7.60911300	-2.53471300

H	3.93138600	-7.32658100	-2.55826300
H	5.29327800	-9.76628400	-1.28685500
H	3.55899200	-9.47774300	-1.30649000
C	4.65866000	-8.14554900	0.00000000
H	5.62817300	-7.62518100	0.00000000
H	3.89224900	-7.35641200	0.00000000
C	7.41249100	-5.12070500	8.54209100
O	6.91328400	-6.24791900	8.63789600
C	8.97975800	-0.72841500	8.54209100
O	9.11103900	-1.95421500	8.63789600
H	9.17799100	1.14316600	9.35508300
H	8.51995700	-3.59898500	9.35508300
C	8.41564100	-0.08656500	7.27496000
H	7.33055500	-0.27735000	7.28874500
H	8.56673200	0.99207700	7.28322900
C	7.24487600	-4.28278800	7.27496000
H	6.20977200	-3.90546900	7.28874500
H	7.91504600	-3.42420200	7.28322900
C	8.27660900	-4.54392100	5.07475300
O	8.83432700	-3.43909800	5.14066300
C	9.43971400	0.20315400	5.07475300
O	9.37030000	1.43881700	5.14066300
C	10.02806600	-0.48888200	3.85286900
H	9.93752500	-1.57573100	3.94524000
H	11.09944800	-0.24506000	3.83697600
C	8.44011900	-5.43741700	3.85286900
H	7.81828400	-6.33338600	3.94524000
H	9.48987400	-5.76195300	3.83697600
N	7.49639400	-5.04098300	6.06777100
N	9.01255900	-0.61742200	6.06777100
H	6.98581800	-5.91190500	5.91360400
H	9.00584800	-1.62695100	5.91360400
N	9.26221900	0.14162500	9.53767900
N	8.09212900	-4.50845900	9.53767900
C	8.22070000	-5.07907200	10.87077900

H	9.27743800	-5.24520400	11.11393600
H	7.68882300	-6.02692700	10.89478900
C	9.65887100	-0.28825500	10.87077900
H	10.65709900	0.09623900	11.11393600
H	9.67218000	-1.37506000	10.89478900
C	8.66297500	0.25764600	11.89399300
O	7.89599300	-0.40438500	12.57564600
C	7.63117900	-4.10835900	11.89399300
O	6.63593800	-4.29820400	12.57564600
O	8.72670400	1.58603500	11.91767300
H	7.99141500	2.03051200	12.41408500
O	8.35056500	-2.98980600	11.91767300
H	7.93602400	-2.23723200	12.41408500
C	8.12487000	-4.68161300	2.55223100
C	8.41177000	-5.50069100	1.28808400
H	8.71257900	-3.75603800	2.53471300
H	7.06797100	-4.37931200	2.55826300
C	8.10729300	-4.72492200	0.00000000
H	9.46725500	-5.81121100	1.28685500
H	7.82104900	-6.42847000	1.30649000
H	7.04899200	-4.42471600	0.00000000
H	8.68673100	-3.78951400	0.00000000
C	9.37715000	0.00804000	2.55223100
C	10.03515200	-0.55785400	1.28808400
H	9.42333300	1.10346500	2.53471300
H	8.31069800	-0.25861000	2.55826300
H	11.10448900	-0.29902800	1.28685500
H	9.98746200	-1.65669400	1.30649000
C	7.41249100	-5.12070500	-8.54209100
O	6.91328400	-6.24791900	-8.63789600
C	8.97975800	-0.72841500	-8.54209100
O	9.11103900	-1.95421500	-8.63789600
H	9.17799100	1.14316600	-9.35508300
H	8.51995700	-3.59898500	-9.35508300
C	8.41564100	-0.08656500	-7.27496000

H	7.33055500	-0.27735000	-7.28874500
H	8.56673200	0.99207700	-7.28322900
C	7.24487600	-4.28278800	-7.27496000
H	6.20977200	-3.90546900	-7.28874500
H	7.91504600	-3.42420200	-7.28322900
C	8.27660900	-4.54392100	-5.07475300
O	8.83432700	-3.43909800	-5.14066300
C	9.43971400	0.20315400	-5.07475300
O	9.37030000	1.43881700	-5.14066300
C	10.02806600	-0.48888200	-3.85286900
H	9.93752500	-1.57573100	-3.94524000
H	11.09944800	-0.24506000	-3.83697600
C	8.44011900	-5.43741700	-3.85286900
H	7.81828400	-6.33338600	-3.94524000
H	9.48987400	-5.76195300	-3.83697600
N	7.49639400	-5.04098300	-6.06777100
N	9.01255900	-0.61742200	-6.06777100
H	6.98581800	-5.91190500	-5.91360400
H	9.00584800	-1.62695100	-5.91360400
N	9.26221900	0.14162500	-9.53767900
N	8.09212900	-4.50845900	-9.53767900
C	8.22070000	-5.07907200	-10.87077900
H	9.27743800	-5.24520400	-11.11393600
H	7.68882300	-6.02692700	-10.89478900
C	9.65887100	-0.28825500	-10.87077900
H	10.65709900	0.09623900	-11.11393600
H	9.67218000	-1.37506000	-10.89478900
C	8.66297500	0.25764600	-11.89399300
O	7.89599300	-0.40438500	-12.57564600
C	7.63117900	-4.10835900	-11.89399300
O	6.63593800	-4.29820400	-12.57564600
O	8.72670400	1.58603500	-11.91767300
H	7.99141500	2.03051200	-12.41408500
O	8.35056500	-2.98980600	-11.91767300
H	7.93602400	-2.23723200	-12.41408500

C	8.12487000	-4.68161300	-2.55223100
C	8.41177000	-5.50069100	-1.28808400
H	8.71257900	-3.75603800	-2.53471300
H	7.06797100	-4.37931200	-2.55826300
H	9.46725500	-5.81121100	-1.28685500
H	7.82104900	-6.42847000	-1.30649000
C	9.37715000	0.00804000	-2.55223100
C	10.03515200	-0.55785400	-1.28808400
H	9.42333300	1.10346500	-2.53471300
H	8.31069800	-0.25861000	-2.55826300
H	11.10448900	-0.29902800	-1.28685500
H	9.98746200	-1.65669400	-1.30649000
C	9.38358200	-0.03825600	0.00000000
H	9.41768700	1.06155000	0.00000000
H	8.31696400	-0.30742000	0.00000000
C	8.14090600	3.85905300	8.54209100
O	8.86749900	2.86312000	8.63789600
C	5.12070500	7.41249100	8.54209100
O	6.24791900	6.91328400	8.63789600
H	3.59898500	8.51995700	9.35508300
H	7.37679100	5.57900700	9.35508300
C	4.28278800	7.24487600	7.27496000
H	3.90546900	6.20977200	7.28874500
H	3.42420200	7.91504600	7.28322900
C	7.33144200	4.13285300	7.27496000
H	6.48712200	3.42508600	7.28874500
H	6.92296900	5.14253000	7.28322900
C	8.07345500	4.89579300	5.07475300
O	7.39550900	5.93120200	5.14066300
C	4.54392100	8.27660900	5.07475300
O	3.43909800	8.83432700	5.14066300
C	5.43741700	8.44011900	3.85286900
H	6.33338600	7.81828400	3.94524000
H	5.76195300	9.48987400	3.83697600
C	8.92900000	4.59064900	3.85286900

H	9.39401500	3.60413900	3.94524000
H	9.73493400	5.33749600	3.83697600
N	8.11381600	3.97157600	6.06777100
N	5.04098300	7.49639400	6.06777100
H	8.61276900	3.09394300	5.91360400
H	5.91190500	6.98581800	5.91360400
N	4.50845900	8.09212900	9.53767900
N	7.95050500	4.75376000	9.53767900
C	8.50895600	4.57979900	10.87077900
H	9.18119900	5.41189500	11.11393600
H	9.06388400	3.64525300	10.89478900
C	5.07907200	8.22070000	10.87077900
H	5.24520400	9.27743800	11.11393600
H	6.02692700	7.68882300	10.89478900
C	4.10835900	7.63117900	11.89399300
O	4.29820400	6.63593800	12.57564600
C	7.37353300	4.55461600	11.89399300
O	7.04032300	3.59778900	12.57564600
O	2.98980600	8.35056500	11.91767300
H	2.23723200	7.93602400	12.41408500
O	6.76453000	5.73689900	11.91767300
H	5.90551200	5.75418300	12.41408500
C	8.11683100	4.69553800	2.55223100
C	8.96962300	4.53446000	1.28808400
H	7.60911300	5.66729600	2.53471300
H	7.32658100	3.93138600	2.55826300
C	8.14554900	4.65866000	0.00000000
H	9.76628400	5.29327800	1.28685500
H	9.47774300	3.55899200	1.30649000
H	7.35641200	3.89224900	0.00000000
H	7.62518100	5.62817300	0.00000000
C	4.68161300	8.12487000	2.55223100
C	5.50069100	8.41177000	1.28808400
H	3.75603800	8.71257900	2.53471300
H	4.37931200	7.06797100	2.55826300

H	5.81121100	9.46725500	1.28685500
H	6.42847000	7.82104900	1.30649000
C	8.14090600	3.85905300	-8.54209100
O	8.86749900	2.86312000	-8.63789600
C	5.12070500	7.41249100	-8.54209100
O	6.24791900	6.91328400	-8.63789600
H	3.59898500	8.51995700	-9.35508300
H	7.37679100	5.57900700	-9.35508300
C	4.28278800	7.24487600	-7.27496000
H	3.90546900	6.20977200	-7.28874500
H	3.42420200	7.91504600	-7.28322900
C	7.33144200	4.13285300	-7.27496000
H	6.48712200	3.42508600	-7.28874500
H	6.92296900	5.14253000	-7.28322900
C	8.07345500	4.89579300	-5.07475300
O	7.39550900	5.93120200	-5.14066300
C	4.54392100	8.27660900	-5.07475300
O	3.43909800	8.83432700	-5.14066300
C	5.43741700	8.44011900	-3.85286900
H	6.33338600	7.81828400	-3.94524000
H	5.76195300	9.48987400	-3.83697600
C	8.92900000	4.59064900	-3.85286900
H	9.39401500	3.60413900	-3.94524000
H	9.73493400	5.33749600	-3.83697600
N	8.11381600	3.97157600	-6.06777100
N	5.04098300	7.49639400	-6.06777100
H	8.61276900	3.09394300	-5.91360400
H	5.91190500	6.98581800	-5.91360400
N	4.50845900	8.09212900	-9.53767900
N	7.95050500	4.75376000	-9.53767900
C	8.50895600	4.57979900	-10.87077900
H	9.18119900	5.41189500	-11.11393600
H	9.06388400	3.64525300	-10.89478900
C	5.07907200	8.22070000	-10.87077900
H	5.24520400	9.27743800	-11.11393600

H	6.02692700	7.68882300	-10.89478900
C	4.10835900	7.63117900	-11.89399300
O	4.29820400	6.63593800	-12.57564600
C	7.37353300	4.55461600	-11.89399300
O	7.04032300	3.59778900	-12.57564600
O	2.98980600	8.35056500	-11.91767300
H	2.23723200	7.93602400	-12.41408500
O	6.76453000	5.73689900	-11.91767300
H	5.90551200	5.75418300	-12.41408500
C	8.11683100	4.69553800	-2.55223100
C	8.96962300	4.53446000	-1.28808400
H	7.60911300	5.66729600	-2.53471300
H	7.32658100	3.93138600	-2.55826300
H	9.76628400	5.29327800	-1.28685500
H	9.47774300	3.55899200	-1.30649000
C	4.68161300	8.12487000	-2.55223100
C	5.50069100	8.41177000	-1.28808400
H	3.75603800	8.71257900	-2.53471300
H	4.37931200	7.06797100	-2.55826300
H	5.81121100	9.46725500	-1.28685500
H	6.42847000	7.82104900	-1.30649000
C	4.72492200	8.10729300	0.00000000
H	3.78951400	8.68673100	0.00000000
H	4.42471600	7.04899200	0.00000000
