# Supplementary material for: Hydrogen-Bond-Driven Peptide Nanotube Formation: A DFT Study
Source: Molecules. 2023 Aug 24;28(17):6217. doi: 10.3390/molecules28176217 (PMC10488343; doi:10.3390/molecules28176217)
Supplement: Supplementary file 1 [file molecules-28-06217-s001.zip › S3.pdf]

Optimized Nanotube Dimers

$[P-(CH_2)_7-P]_4$ Dimer

C	3.03681200	0.79610200	22.57796800
O	3.54761400	-0.32366700	22.62088400
C	-0.79610200	3.03681200	22.57796800
O	0.32366700	3.54761400	22.62088400
C	-3.03681200	-0.79610200	22.57796800
O	-3.54761400	0.32366700	22.62088400
C	0.79610200	-3.03681200	22.57796800
O	-0.32366700	-3.54761400	22.62088400
H	-2.53763100	2.52406300	23.53787100
H	2.52406300	2.53763100	23.53787100
H	-2.52406300	-2.53763100	23.53787100
H	2.53763100	-2.52406300	23.53787100
C	-1.30513200	2.27200500	21.36075900
H	-0.94671900	1.23635800	21.47289800
H	-2.39333900	2.21081300	21.34660400
C	2.27200500	1.30513200	21.36075900
H	1.23635800	0.94671900	21.47289800
H	2.21081300	2.39333900	21.34660400
C	-2.27200500	-1.30513200	21.36075900
H	-1.23635800	-0.94671900	21.47289800
H	-2.21081300	-2.39333900	21.34660400
C	1.30513200	-2.27200500	21.36075900
H	0.94671900	-1.23635800	21.47289800
H	2.39333900	-2.21081300	21.34660400
C	2.66742300	1.61721400	18.98788300
O	2.03996800	2.68328100	19.00625500
C	-1.61721400	2.66742300	18.98788300
O	-2.68328100	2.03996800	19.00625500
C	-2.66742300	-1.61721400	18.98788300
O	-2.03996800	-2.68328100	19.00625500
C	1.61721400	-2.66742300	18.98788300
O	2.68328100	-2.03996800	19.00625500
C	-1.08375000	3.34723000	17.73318000
H	0.00952100	3.40362600	17.78333600

H	-1.45194900	4.38401300	17.75324000
C	3.34723000	1.08375000	17.73318000
H	3.40362600	-0.00952100	17.78333600
H	4.38401300	1.45194900	17.75324000
C	-3.34723000	-1.08375000	17.73318000
H	-3.40362600	0.00952100	17.78333600
H	-4.38401300	-1.45194900	17.75324000
C	1.08375000	-3.34723000	17.73318000
H	-0.00952100	-3.40362600	17.78333600
H	1.45194900	-4.38401300	17.75324000
N	2.84984400	0.86691300	20.11178900
N	-0.86691300	2.84984400	20.11178900
N	-2.84984400	-0.86691300	20.11178900
N	0.86691300	-2.84984400	20.11178900
H	3.13927000	-0.10416500	20.01799200
H	0.10416500	3.13927000	20.01799200
H	-3.13927000	0.10416500	20.01799200
H	-0.10416500	-3.13927000	20.01799200
N	-1.67180800	3.04652300	23.62147300
N	3.04652300	1.67180800	23.62147300
N	-3.04652300	-1.67180800	23.62147300
N	1.67180800	-3.04652300	23.62147300
C	3.59102100	1.32087400	24.92144100
H	4.43518800	1.97613500	25.17823400
H	3.95424300	0.29494100	24.86638100
C	-1.32087400	3.59102100	24.92144100
H	-1.97613500	4.43518800	25.17823400
H	-0.29494100	3.95424300	24.86638100
C	-3.59102100	-1.32087400	24.92144100
H	-4.43518800	-1.97613500	25.17823400
H	-3.95424300	-0.29494100	24.86638100
C	1.32087400	-3.59102100	24.92144100
H	0.29494100	-3.95424300	24.86638100
H	1.97613500	-4.43518800	25.17823400
C	-1.44184600	2.54800800	26.02521500
O	-0.59877200	2.35347100	26.88945800

C	2.54800800	1.44184600	26.02521500
O	2.35347100	0.59877200	26.88945800
C	-2.54800800	-1.44184600	26.02521500
O	-2.35347100	-0.59877200	26.88945800
C	1.44184600	-2.54800800	26.02521500
O	0.59877200	-2.35347100	26.88945800
O	-2.58551300	1.88026800	25.94946500
H	-2.55876300	1.07167200	26.53686400
O	1.88026800	2.58551300	25.94946500
H	1.07167200	2.55876300	26.53686400
O	-1.88026800	-2.58551300	25.94946500
H	-1.07167200	-2.55876300	26.53686400
O	2.58551300	-1.88026800	25.94946500
H	2.55876300	-1.07167200	26.53686400
C	-2.66287800	-1.54341300	16.44229000
H	-2.56154200	-2.63471100	16.46426000
H	-1.63998600	-1.14138100	16.41512400
C	-1.54341300	2.66287800	16.44229000
H	-1.14138100	1.63998600	16.41512400
H	-2.63471100	2.56154200	16.46426000
C	2.66287800	1.54341300	16.44229000
H	1.63998600	1.14138100	16.41512400
H	2.56154200	2.63471100	16.46426000
C	1.54341300	-2.66287800	16.44229000
H	2.63471100	-2.56154200	16.46426000
H	1.14138100	-1.63998600	16.41512400
C	-3.41090700	-1.11067600	15.17534000
H	-4.42898500	-1.52763500	15.19051700
H	-3.52908800	-0.01744100	15.17264800
C	-1.11067600	3.41090700	15.17534000
H	-1.52763500	4.42898500	15.19051700
H	-0.01744100	3.52908800	15.17264800
C	3.41090700	1.11067600	15.17534000
H	4.42898500	1.52763500	15.19051700
H	3.52908800	0.01744100	15.17264800
C	1.11067600	-3.41090700	15.17534000

H	1.52763500	-4.42898500	15.19051700
H	0.01744100	-3.52908800	15.17264800
C	-1.54699100	2.69900600	13.88843700
H	-1.13839100	1.67714400	13.88900700
H	-2.64150100	2.58730600	13.88898900
C	3.04269300	0.74484200	5.21606900
O	3.57116700	-0.36837400	5.21013600
C	-0.74484200	3.04269300	5.21606900
O	0.36837400	3.57116700	5.21013600
C	-3.04269300	-0.74484200	5.21606900
O	-3.57116700	0.36837400	5.21013600
C	0.74484200	-3.04269300	5.21606900
O	-0.36837400	-3.57116700	5.21013600
H	-2.45969300	2.56951600	4.17435100
H	2.56951600	2.45969300	4.17435100
H	-2.56951600	-2.45969300	4.17435100
H	2.45969300	-2.56951600	4.17435100
C	-1.28112000	2.26734900	6.41339100
H	-0.93120300	1.22946000	6.29940300
H	-2.37020500	2.21624600	6.41080800
C	2.26734900	1.28112000	6.41339100
H	1.22946000	0.93120300	6.29940300
H	2.21624600	2.37020500	6.41080800
C	-2.26734900	-1.28112000	6.41339100
H	-1.22946000	-0.93120300	6.29940300
H	-2.21624600	-2.37020500	6.41080800
C	1.28112000	-2.26734900	6.41339100
H	0.93120300	-1.22946000	6.29940300
H	2.37020500	-2.21624600	6.41080800
C	2.66157800	1.61201200	8.78729000
O	2.04169500	2.68259600	8.76360400
C	-1.61201200	2.66157800	8.78729000
O	-2.68259600	2.04169500	8.76360400
C	-2.66157800	-1.61201200	8.78729000
O	-2.04169500	-2.68259600	8.76360400
C	1.61201200	-2.66157800	8.78729000

O	2.68259600	-2.04169500	8.76360400
C	-1.08331200	3.34317700	10.04327300
H	0.00982700	3.40326000	9.99552800
H	-1.45504100	4.37868300	10.02201500
C	3.34317700	1.08331200	10.04327300
H	3.40326000	-0.00982700	9.99552800
H	4.37868300	1.45504100	10.02201500
C	-3.34317700	-1.08331200	10.04327300
H	-3.40326000	0.00982700	9.99552800
H	-4.37868300	-1.45504100	10.02201500
C	1.08331200	-3.34317700	10.04327300
H	-0.00982700	-3.40326000	9.99552800
H	1.45504100	-4.37868300	10.02201500
N	2.83482200	0.85121100	7.67023200
N	-0.85121100	2.83482200	7.67023200
N	-2.83482200	-0.85121100	7.67023200
N	0.85121100	-2.83482200	7.67023200
H	3.12548200	-0.11933000	7.76936500
H	0.11933000	3.12548200	7.76936500
H	-3.12548200	0.11933000	7.76936500
H	-0.11933000	-3.12548200	7.76936500
N	-1.56999500	3.05366800	4.13521700
N	3.05366800	1.56999500	4.13521700
N	-3.05366800	-1.56999500	4.13521700
N	1.56999500	-3.05366800	4.13521700
C	3.71739300	1.16852500	2.91368600
H	4.78887300	1.41504700	2.93143400
H	3.66070200	0.07960000	2.82311100
C	-1.16852500	3.71739300	2.91368600
H	-1.41504700	4.78887300	2.93143400
H	-0.07960000	3.66070200	2.82311100
C	-3.71739300	-1.16852500	2.91368600
H	-4.78887300	-1.41504700	2.93143400
H	-3.66070200	-0.07960000	2.82311100
C	1.16852500	-3.71739300	2.91368600
H	0.07960000	-3.66070200	2.82311100

H	1.41504700	-4.78887300	2.93143400
C	-1.77945600	3.15618500	1.64910400
O	-1.46113300	3.61948000	0.55755800
C	3.15618500	1.77945600	1.64910400
O	3.61948000	1.46113300	0.55755800
C	-3.15618500	-1.77945600	1.64910400
O	-3.61948000	-1.46113300	0.55755800
C	1.77945600	-3.15618500	1.64910400
O	1.46113300	-3.61948000	0.55755800
O	-2.66169500	2.18692900	1.82328300
H	-3.04177200	1.91582100	0.92971700
O	2.18692900	2.66169500	1.82328300
H	1.91582100	3.04177200	0.92971700
O	-2.18692900	-2.66169500	1.82328300
H	-1.91582100	-3.04177200	0.92971700
O	2.66169500	-2.18692900	1.82328300
H	3.04177200	-1.91582100	0.92971700
C	-2.65886900	-1.54309500	11.33410800
H	-2.55602100	-2.63426100	11.31146400
H	-1.63664500	-1.13941300	11.36259100
C	-1.54309500	2.65886900	11.33410800
H	-1.13941300	1.63664500	11.36259100
H	-2.63426100	2.55602100	11.31146400
C	2.65886900	1.54309500	11.33410800
H	1.63664500	1.13941300	11.36259100
H	2.55602100	2.63426100	11.31146400
C	1.54309500	-2.65886900	11.33410800
H	2.63426100	-2.55602100	11.31146400
H	1.13941300	-1.63664500	11.36259100
C	-3.40915200	-1.11175900	12.60028100
H	-4.42669600	-1.52996400	12.58346300
H	-3.52876000	-0.01867300	12.60275500
C	-1.11175900	3.40915200	12.60028100
H	-1.52996400	4.42669600	12.58346300
H	-0.01867300	3.52876000	12.60275500
C	3.40915200	1.11175900	12.60028100

H	4.42669600	1.52996400	12.58346300
H	3.52876000	0.01867300	12.60275500
C	1.11175900	-3.40915200	12.60028100
H	1.52996400	-4.42669600	12.58346300
H	0.01867300	-3.52876000	12.60275500
C	1.54699100	-2.69900600	13.88843700
C	-2.69900600	-1.54699100	13.88843700
C	2.69900600	1.54699100	13.88843700
H	1.67714400	1.13839100	13.88900700
H	2.58730600	2.64150100	13.88898900
H	-1.67714400	-1.13839100	13.88900700
H	-2.58730600	-2.64150100	13.88898900
H	2.64150100	-2.58730600	13.88898900
H	1.13839100	-1.67714400	13.88900700
C	-0.79610200	-3.03681200	-22.57796800
O	0.32366700	-3.54761400	-22.62088400
C	-3.03681200	0.79610200	-22.57796800
O	-3.54761400	-0.32366700	-22.62088400
C	0.79610200	3.03681200	-22.57796800
O	-0.32366700	3.54761400	-22.62088400
C	3.03681200	-0.79610200	-22.57796800
O	3.54761400	0.32366700	-22.62088400
H	-2.52406300	2.53763100	-23.53787100
H	-2.53763100	-2.52406300	-23.53787100
H	2.53763100	2.52406300	-23.53787100
H	2.52406300	-2.53763100	-23.53787100
C	-2.27200500	1.30513200	-21.36075900
H	-1.23635800	0.94671900	-21.47289800
H	-2.21081300	2.39333900	-21.34660400
C	-1.30513200	-2.27200500	-21.36075900
H	-0.94671900	-1.23635800	-21.47289800
H	-2.39333900	-2.21081300	-21.34660400
C	1.30513200	2.27200500	-21.36075900
H	0.94671900	1.23635800	-21.47289800
H	2.39333900	2.21081300	-21.34660400
C	2.27200500	-1.30513200	-21.36075900

H	1.23635800	-0.94671900	-21.47289800
H	2.21081300	-2.39333900	-21.34660400
C	-1.61721400	-2.66742300	-18.98788300
O	-2.68328100	-2.03996800	-19.00625500
C	-2.66742300	1.61721400	-18.98788300
O	-2.03996800	2.68328100	-19.00625500
C	1.61721400	2.66742300	-18.98788300
O	2.68328100	2.03996800	-19.00625500
C	2.66742300	-1.61721400	-18.98788300
O	2.03996800	-2.68328100	-19.00625500
C	-3.34723000	1.08375000	-17.73318000
H	-3.40362600	-0.00952100	-17.78333600
H	-4.38401300	1.45194900	-17.75324000
C	-1.08375000	-3.34723000	-17.73318000
H	0.00952100	-3.40362600	-17.78333600
H	-1.45194900	-4.38401300	-17.75324000
C	1.08375000	3.34723000	-17.73318000
H	-0.00952100	3.40362600	-17.78333600
H	1.45194900	4.38401300	-17.75324000
C	3.34723000	-1.08375000	-17.73318000
H	3.40362600	0.00952100	-17.78333600
H	4.38401300	-1.45194900	-17.75324000
N	-0.86691300	-2.84984400	-20.11178900
N	-2.84984400	0.86691300	-20.11178900
N	0.86691300	2.84984400	-20.11178900
N	2.84984400	-0.86691300	-20.11178900
H	0.10416500	-3.13927000	-20.01799200
H	-3.13927000	-0.10416500	-20.01799200
H	-0.10416500	3.13927000	-20.01799200
H	3.13927000	0.10416500	-20.01799200
N	-3.04652300	1.67180800	-23.62147300
N	-1.67180800	-3.04652300	-23.62147300
N	1.67180800	3.04652300	-23.62147300
N	3.04652300	-1.67180800	-23.62147300
C	-1.32087400	-3.59102100	-24.92144100
H	-1.97613500	-4.43518800	-25.17823400

H	-0.29494100	-3.95424300	-24.86638100
C	-3.59102100	1.32087400	-24.92144100
H	-4.43518800	1.97613500	-25.17823400
H	-3.95424300	0.29494100	-24.86638100
C	1.32087400	3.59102100	-24.92144100
H	1.97613500	4.43518800	-25.17823400
H	0.29494100	3.95424300	-24.86638100
C	3.59102100	-1.32087400	-24.92144100
H	3.95424300	-0.29494100	-24.86638100
H	4.43518800	-1.97613500	-25.17823400
C	-2.54800800	1.44184600	-26.02521500
O	-2.35347100	0.59877200	-26.88945800
C	-1.44184600	-2.54800800	-26.02521500
O	-0.59877200	-2.35347100	-26.88945800
C	1.44184600	2.54800800	-26.02521500
O	0.59877200	2.35347100	-26.88945800
C	2.54800800	-1.44184600	-26.02521500
O	2.35347100	-0.59877200	-26.88945800
O	-1.88026800	2.58551300	-25.94946500
H	-1.07167200	2.55876300	-26.53686400
O	-2.58551300	-1.88026800	-25.94946500
H	-2.55876300	-1.07167200	-26.53686400
O	2.58551300	1.88026800	-25.94946500
H	2.55876300	1.07167200	-26.53686400
O	1.88026800	-2.58551300	-25.94946500
H	1.07167200	-2.55876300	-26.53686400
C	1.54341300	2.66287800	-16.44229000
H	2.63471100	2.56154200	-16.46426000
H	1.14138100	1.63998600	-16.41512400
C	-2.66287800	1.54341300	-16.44229000
H	-1.63998600	1.14138100	-16.41512400
H	-2.56154200	2.63471100	-16.46426000
C	-1.54341300	-2.66287800	-16.44229000
H	-1.14138100	-1.63998600	-16.41512400
H	-2.63471100	-2.56154200	-16.46426000
C	2.66287800	-1.54341300	-16.44229000

H	2.56154200	-2.63471100	-16.46426000
H	1.63998600	-1.14138100	-16.41512400
C	1.11067600	3.41090700	-15.17534000
H	1.52763500	4.42898500	-15.19051700
H	0.01744100	3.52908800	-15.17264800
C	-3.41090700	1.11067600	-15.17534000
H	-4.42898500	1.52763500	-15.19051700
H	-3.52908800	0.01744100	-15.17264800
C	-1.11067600	-3.41090700	-15.17534000
H	-1.52763500	-4.42898500	-15.19051700
H	-0.01744100	-3.52908800	-15.17264800
C	3.41090700	-1.11067600	-15.17534000
H	4.42898500	-1.52763500	-15.19051700
H	3.52908800	-0.01744100	-15.17264800
C	-2.69900600	1.54699100	-13.88843700
H	-1.67714400	1.13839100	-13.88900700
H	-2.58730600	2.64150100	-13.88898900
C	-0.74484200	-3.04269300	-5.21606900
O	0.36837400	-3.57116700	-5.21013600
C	-3.04269300	0.74484200	-5.21606900
O	-3.57116700	-0.36837400	-5.21013600
C	0.74484200	3.04269300	-5.21606900
O	-0.36837400	3.57116700	-5.21013600
C	3.04269300	-0.74484200	-5.21606900
O	3.57116700	0.36837400	-5.21013600
H	-2.56951600	2.45969300	-4.17435100
H	-2.45969300	-2.56951600	-4.17435100
H	2.45969300	2.56951600	-4.17435100
H	2.56951600	-2.45969300	-4.17435100
C	-2.26734900	1.28112000	-6.41339100
H	-1.22946000	0.93120300	-6.29940300
H	-2.21624600	2.37020500	-6.41080800
C	-1.28112000	-2.26734900	-6.41339100
H	-0.93120300	-1.22946000	-6.29940300
H	-2.37020500	-2.21624600	-6.41080800
C	1.28112000	2.26734900	-6.41339100

H	0.93120300	1.22946000	-6.29940300
H	2.37020500	2.21624600	-6.41080800
C	2.26734900	-1.28112000	-6.41339100
H	1.22946000	-0.93120300	-6.29940300
H	2.21624600	-2.37020500	-6.41080800
C	-1.61201200	-2.66157800	-8.78729000
O	-2.68259600	-2.04169500	-8.76360400
C	-2.66157800	1.61201200	-8.78729000
O	-2.04169500	2.68259600	-8.76360400
C	1.61201200	2.66157800	-8.78729000
O	2.68259600	2.04169500	-8.76360400
C	2.66157800	-1.61201200	-8.78729000
O	2.04169500	-2.68259600	-8.76360400
C	-3.34317700	1.08331200	-10.04327300
H	-3.40326000	-0.00982700	-9.99552800
H	-4.37868300	1.45504100	-10.02201500
C	-1.08331200	-3.34317700	-10.04327300
H	0.00982700	-3.40326000	-9.99552800
H	-1.45504100	-4.37868300	-10.02201500
C	1.08331200	3.34317700	-10.04327300
H	-0.00982700	3.40326000	-9.99552800
H	1.45504100	4.37868300	-10.02201500
C	3.34317700	-1.08331200	-10.04327300
H	3.40326000	0.00982700	-9.99552800
H	4.37868300	-1.45504100	-10.02201500
N	-0.85121100	-2.83482200	-7.67023200
N	-2.83482200	0.85121100	-7.67023200
N	0.85121100	2.83482200	-7.67023200
N	2.83482200	-0.85121100	-7.67023200
H	0.11933000	-3.12548200	-7.76936500
H	-3.12548200	-0.11933000	-7.76936500
H	-0.11933000	3.12548200	-7.76936500
H	3.12548200	0.11933000	-7.76936500
N	-3.05366800	1.56999500	-4.13521700
N	-1.56999500	-3.05366800	-4.13521700
N	1.56999500	3.05366800	-4.13521700

N	3.05366800	-1.56999500	-4.13521700
C	-1.16852500	-3.71739300	-2.91368600
H	-1.41504700	-4.78887300	-2.93143400
H	-0.07960000	-3.66070200	-2.82311100
C	-3.71739300	1.16852500	-2.91368600
H	-4.78887300	1.41504700	-2.93143400
H	-3.66070200	0.07960000	-2.82311100
C	1.16852500	3.71739300	-2.91368600
H	1.41504700	4.78887300	-2.93143400
H	0.07960000	3.66070200	-2.82311100
C	3.71739300	-1.16852500	-2.91368600
H	3.66070200	-0.07960000	-2.82311100
H	4.78887300	-1.41504700	-2.93143400
C	-3.15618500	1.77945600	-1.64910400
O	-3.61948000	1.46113300	-0.55755800
C	-1.77945600	-3.15618500	-1.64910400
O	-1.46113300	-3.61948000	-0.55755800
C	1.77945600	3.15618500	-1.64910400
O	1.46113300	3.61948000	-0.55755800
C	3.15618500	-1.77945600	-1.64910400
O	3.61948000	-1.46113300	-0.55755800
O	-2.18692900	2.66169500	-1.82328300
H	-1.91582100	3.04177200	-0.92971700
O	-2.66169500	-2.18692900	-1.82328300
H	-3.04177200	-1.91582100	-0.92971700
O	2.66169500	2.18692900	-1.82328300
H	3.04177200	1.91582100	-0.92971700
O	2.18692900	-2.66169500	-1.82328300
H	1.91582100	-3.04177200	-0.92971700
C	1.54309500	2.65886900	-11.33410800
H	2.63426100	2.55602100	-11.31146400
H	1.13941300	1.63664500	-11.36259100
C	-2.65886900	1.54309500	-11.33410800
H	-1.63664500	1.13941300	-11.36259100
H	-2.55602100	2.63426100	-11.31146400
C	-1.54309500	-2.65886900	-11.33410800

H	-1.13941300	-1.63664500	-11.36259100
H	-2.63426100	-2.55602100	-11.31146400
C	2.65886900	-1.54309500	-11.33410800
H	2.55602100	-2.63426100	-11.31146400
H	1.63664500	-1.13941300	-11.36259100
C	1.11175900	3.40915200	-12.60028100
H	1.52996400	4.42669600	-12.58346300
H	0.01867300	3.52876000	-12.60275500
C	-3.40915200	1.11175900	-12.60028100
H	-4.42669600	1.52996400	-12.58346300
H	-3.52876000	0.01867300	-12.60275500
C	-1.11175900	-3.40915200	-12.60028100
H	-1.52996400	-4.42669600	-12.58346300
H	-0.01867300	-3.52876000	-12.60275500
C	3.40915200	-1.11175900	-12.60028100
H	4.42669600	-1.52996400	-12.58346300
H	3.52876000	-0.01867300	-12.60275500
C	2.69900600	-1.54699100	-13.88843700
C	1.54699100	2.69900600	-13.88843700
C	-1.54699100	-2.69900600	-13.88843700
H	-1.13839100	-1.67714400	-13.88900700
H	-2.64150100	-2.58730600	-13.88898900
H	1.13839100	1.67714400	-13.88900700
H	2.64150100	2.58730600	-13.88898900
H	2.58730600	-2.64150100	-13.88898900
H	1.67714400	-1.13839100	-13.88900700

[P-$(CH_2)_7$-P]$_6$ Dimer

C	-0.69353500	-4.40633500	5.19690000
O	0.44987900	-4.87071400	5.11684100
C	3.46923100	-2.80378600	5.19690000
O	4.44310200	-2.04575000	5.11684100
C	4.16276500	1.60254900	5.19690000
O	3.99322300	2.82496400	5.11684100
C	-3.46923100	2.80378600	5.19690000
O	-4.44310200	2.04575000	5.11684100
C	-4.16276500	-1.60254900	5.19690000

O	-3.99322300	-2.82496400	5.11684100
C	0.69353500	4.40633500	5.19690000
O	-0.44987900	4.87071400	5.11684100
H	-2.55790600	-4.16383000	4.36424000
H	2.32702900	-4.29712600	4.36424000
H	4.88493500	-0.13329600	4.36424000
H	-2.32702900	4.29712600	4.36424000
H	-4.88493500	0.13329600	4.36424000
H	2.55790600	4.16383000	4.36424000
C	-1.13754900	-3.57740700	6.40191500
H	-2.21704000	-3.43686300	6.39387600
H	-0.66405000	-2.58709400	6.30785600
C	2.52935100	-2.77384900	6.40191500
H	1.86789100	-3.63844400	6.39387600
H	1.90846400	-1.86863100	6.30785600
C	3.66689900	0.80355700	6.40191500
H	4.08493000	-0.20158100	6.39387600
H	2.57251400	0.71846200	6.30785600
C	-2.52935100	2.77384900	6.40191500
H	-1.86789100	3.63844400	6.39387600
H	-1.90846400	1.86863100	6.30785600
C	-3.66689900	-0.80355700	6.40191500
H	-4.08493000	0.20158100	6.39387600
H	-2.57251400	-0.71846200	6.30785600
C	1.13754900	3.57740700	6.40191500
H	2.21704000	3.43686300	6.39387600
H	0.66405000	2.58709400	6.30785600
C	-1.66096600	-4.34241600	8.66814900
O	-2.83391800	-3.94783200	8.59708900
C	2.93015900	-3.60964600	8.66814900
O	2.00196400	-4.42816000	8.59708900
C	4.59112500	0.73276900	8.66814900
O	4.83588100	-0.48032900	8.59708900
C	-2.93015900	3.60964600	8.66814900
O	-2.00196400	4.42816000	8.59708900

C	-4.59112500	-0.73276900	8.66814900
O	-4.83588100	0.48032900	8.59708900
C	1.66096600	4.34241600	8.66814900
O	2.83391800	3.94783200	8.59708900
C	-1.13392900	-5.04970100	9.90991900
H	-0.04355700	-5.13675400	9.86268000
H	-1.54119700	-6.07082600	9.88865100
C	3.80620500	-3.50686200	9.90991900
H	4.42678100	-2.60609800	9.86268000
H	4.48689100	-4.37012900	9.88865100
C	4.94013400	1.54283900	9.90991900
H	4.47033800	2.53065600	9.86268000
H	6.02808800	1.70069700	9.88865100
C	-3.80620500	3.50686200	9.90991900
H	-4.42678100	2.60609800	9.86268000
H	-4.48689100	4.37012900	9.88865100
C	-4.94013400	-1.54283900	9.90991900
H	-4.47033800	-2.53065600	9.86268000
H	-6.02808800	-1.70069700	9.88865100
C	1.13392900	5.04970100	9.90991900
H	0.04355700	5.13675400	9.86268000
H	1.54119700	6.07082600	9.88865100
N	-0.76975700	-4.20589500	7.65345400
N	3.25753300	-2.76957600	7.65345400
N	4.02729000	1.43631900	7.65345400
N	-3.25753300	2.76957600	7.65345400
N	-4.02729000	-1.43631900	7.65345400
N	0.76975700	4.20589500	7.65345400
H	3.94511000	-2.03234400	7.82258100
H	3.73261600	2.40039300	7.82258100
H	0.21249400	-4.43273700	7.82258100
H	-3.94511000	2.03234400	7.82258100
H	-3.73261600	-2.40039300	7.82258100
H	-0.21249400	4.43273700	7.82258100
N	-1.63185600	-4.56770000	4.23218400

N	3.13981600	-3.69707800	4.23218400
N	4.77167200	0.87062100	4.23218400
N	-3.13981600	3.69707800	4.23218400
N	-4.77167200	-0.87062100	4.23218400
N	1.63185600	4.56770000	4.23218400
C	3.98031400	-3.85517900	3.06305700
H	4.63006400	-4.73896100	3.16284700
H	4.63346900	-2.98504700	2.99262200
C	-1.34852500	-5.37464300	3.06305700
H	-1.78902800	-6.37923400	3.16284700
H	-0.26839200	-5.50522500	2.99262200
C	-5.32884000	-1.51946400	3.06305700
H	-4.90186100	-2.52017800	2.99262200
H	-6.41909300	-1.64027300	3.16284700
C	-3.98031400	3.85517900	3.06305700
H	-4.63006400	4.73896100	3.16284700
H	-4.63346900	2.98504700	2.99262200
C	1.34852500	5.37464300	3.06305700
H	0.26839200	5.50522500	2.99262200
H	1.78902800	6.37923400	3.16284700
C	5.32884000	1.51946400	3.06305700
H	6.41909300	1.64027300	3.16284700
H	4.90186100	2.52017800	2.99262200
C	1.85144600	4.82495200	1.74306700
O	1.31303900	5.16119800	0.69330800
C	3.25280800	-4.01587500	1.74306700
O	3.81320900	-3.71772400	0.69330800
C	-1.85144600	-4.82495200	1.74306700
O	-1.31303900	-5.16119800	0.69330800
C	-5.10425400	-0.80907700	1.74306700
O	-5.12624800	-1.44347400	0.69330800
C	-3.25280800	4.01587500	1.74306700
O	-3.81320900	3.71772400	0.69330800
C	5.10425400	0.80907700	1.74306700
O	5.12624800	1.44347400	0.69330800

O	2.91830300	4.04814500	1.82541700
H	3.24824600	3.84585500	0.89814100
O	2.04664500	-4.55139600	1.82541700
H	1.70648600	-4.73599100	0.89814100
O	-2.91830300	-4.04814500	1.82541700
H	-3.24824600	-3.84585500	0.89814100
O	-4.96494700	0.50325200	1.82541700
H	-4.95473100	0.89013500	0.89814100
O	-2.04664500	4.55139600	1.82541700
H	-1.70648600	4.73599100	0.89814100
O	4.96494700	-0.50325200	1.82541700
H	4.95473100	-0.89013500	0.89814100
C	-1.57471800	-4.35447600	11.20549500
C	-1.14199700	-5.10402200	12.47135000
H	-1.16004300	-3.33646000	11.22715300
H	-2.66543500	-4.24483600	11.19147600
C	-1.57355500	-4.39057100	13.75904800
H	-0.04892800	-5.22620500	12.47119200
H	-1.56269900	-6.12044500	12.45651400
C	-2.98372800	3.54098300	11.20549500
C	-3.84921400	3.54100900	12.47135000
H	-2.30943800	2.67285600	11.22715300
H	-2.34341800	4.43075200	11.19147600
C	-3.01556800	3.55802400	13.75904800
H	-4.50156200	2.65547500	12.47119200
H	-4.51911100	4.41355900	12.45651400
C	2.98372800	-3.54098300	11.20549500
C	3.84921400	-3.54100900	12.47135000
H	2.30943800	-2.67285600	11.22715300
H	2.34341800	-4.43075200	11.19147600
C	3.01556800	-3.55802400	13.75904800
H	4.50156200	-2.65547500	12.47119200
H	4.51911100	-4.41355900	12.45651400
C	4.55844500	0.81349200	11.20549500
C	4.99121100	1.56301300	12.47135000

H	3.46948000	0.66360400	11.22715300
H	5.00885300	-0.18591600	11.19147600
C	4.58912300	0.83254700	13.75904800
H	4.55049000	2.57073000	12.47119200
H	6.08181000	1.70688500	12.45651400
C	-4.55844500	-0.81349200	11.20549500
C	-4.99121100	-1.56301300	12.47135000
H	-3.46948000	-0.66360400	11.22715300
H	-5.00885300	0.18591600	11.19147600
C	-4.58912300	-0.83254700	13.75904800
H	-4.55049000	-2.57073000	12.47119200
H	-6.08181000	-1.70688500	12.45651400
C	1.57471800	4.35447600	11.20549500
C	1.14199700	5.10402200	12.47135000
H	1.16004300	3.33646000	11.22715300
H	2.66543500	4.24483600	11.19147600
C	1.57355500	4.39057100	13.75904800
H	0.04892800	5.22620500	12.47119200
H	1.56269900	6.12044500	12.45651400
C	-0.75999800	-4.39849700	22.36340200
O	0.37736300	-4.86898700	22.47447500
C	3.42921100	-2.85742600	22.36340200
O	4.40534800	-2.10768700	22.47447500
C	4.18920900	1.54107100	22.36340200
O	4.02798500	2.76130000	22.47447500
C	-3.42921100	2.85742600	22.36340200
O	-4.40534800	2.10768700	22.47447500
C	-4.18920900	-1.54107100	22.36340200
O	-4.02798500	-2.76130000	22.47447500
C	0.75999800	4.39849700	22.36340200
O	-0.37736300	4.86898700	22.47447500
H	-2.61381800	-4.03530500	23.16425400
H	2.18776700	-4.28128500	23.16425400
H	4.80158500	-0.24598000	23.16425400
H	-2.18776700	4.28128500	23.16425400

H	-4.80158500	0.24598000	23.16425400
H	2.61381800	4.03530500	23.16425400
C	-1.19719900	-3.62288600	21.12015300
H	-2.27775300	-3.48989400	21.11235800
H	-0.73803500	-2.62326400	21.19725700
C	2.53891200	-2.84824700	21.12015300
H	1.88346000	-3.71753800	21.11235800
H	1.90279500	-1.95078900	21.19725700
C	3.73611000	0.77463900	21.12015300
H	4.16121300	-0.22764500	21.11235800
H	2.64083100	0.67247500	21.19725700
C	-2.53891200	2.84824700	21.12015300
H	-1.88346000	3.71753800	21.11235800
H	-1.90279500	1.95078900	21.19725700
C	-3.73611000	-0.77463900	21.12015300
H	-4.16121300	0.22764500	21.11235800
H	-2.64083100	-0.67247500	21.19725700
C	1.19719900	3.62288600	21.12015300
H	2.27775300	3.48989400	21.11235800
H	0.73803500	2.62326400	21.19725700
C	-1.69318100	-4.36654500	18.84972100
O	-2.85532200	-3.94065100	18.90992000
C	2.93494800	-3.64961000	18.84972100
O	1.98504300	-4.44310600	18.90992000
C	4.62812900	0.71693500	18.84972100
O	4.84036400	-0.50245600	18.90992000
C	-2.93494800	3.64961000	18.84972100
O	-1.98504300	4.44310600	18.90992000
C	-4.62812900	-0.71693500	18.84972100
O	-4.84036400	0.50245600	18.90992000
C	1.69318100	4.36654500	18.84972100
O	2.85532200	3.94065100	18.90992000
C	-1.16342200	-5.07023900	17.60750300
H	-0.07357300	-5.16190000	17.66089400
H	-1.57412600	-6.09018500	17.62181600

C	3.80924500	-3.54267200	17.60750300
H	4.43355000	-2.64466600	17.66089400
H	4.48719200	-4.40832500	17.62181600
C	4.97266700	1.52756700	17.60750300
H	4.50712300	2.51723400	17.66089400
H	6.06131800	1.68185900	17.62181600
C	-3.80924500	3.54267200	17.60750300
H	-4.43355000	2.64466600	17.66089400
H	-4.48719200	4.40832500	17.62181600
C	-4.97266700	-1.52756700	17.60750300
H	-4.50712300	-2.51723400	17.66089400
H	-6.06131800	-1.68185900	17.62181600
C	1.16342200	5.07023900	17.60750300
H	0.07357300	5.16190000	17.66089400
H	1.57412600	6.09018500	17.62181600
N	-0.81663200	-4.27005600	19.88406500
N	3.28966100	-2.84225200	19.88406500
N	4.10629300	1.42780400	19.88406500
N	-3.28966100	2.84225200	19.88406500
N	-4.10629300	-1.42780400	19.88406500
N	0.81663200	4.27005600	19.88406500
H	3.97334300	-2.10142800	19.72042000
H	3.80656100	2.39030200	19.72042000
H	0.16678200	-4.49173000	19.72042000
H	-3.97334300	2.10142800	19.72042000
H	-3.80656100	-2.39030200	19.72042000
H	-0.16678200	4.49173000	19.72042000
N	-1.70736700	-4.47336400	23.32948300
N	3.02036300	-3.71530500	23.32948300
N	4.72773000	0.75805900	23.32948300
N	-3.02036300	3.71530500	23.32948300
N	-4.72773000	-0.75805900	23.32948300
N	1.70736700	4.47336400	23.32948300
C	3.64931100	-3.77341000	24.64113300
H	4.04536900	-4.77941100	24.82896000

H	4.46763900	-3.05560300	24.65766400
C	-1.44321300	-5.04710100	24.64113300
H	-2.11640600	-5.89309800	24.82896000
H	-0.41241000	-5.39689000	24.65766400
C	-5.09252400	-1.27369200	24.64113300
H	-4.88004900	-2.34128700	24.65766400
H	-6.16177600	-1.11368700	24.82896000
C	-3.64931100	3.77341000	24.64113300
H	-4.04536900	4.77941100	24.82896000
H	-4.46763900	3.05560300	24.65766400
C	1.44321300	5.04710100	24.64113300
H	0.41241000	5.39689000	24.65766400
H	2.11640600	5.89309800	24.82896000
C	5.09252400	1.27369200	24.64113300
H	6.16177600	1.11368700	24.82896000
H	4.88004900	2.34128700	24.65766400
C	1.66813900	3.99704900	25.72639000
O	0.81721100	3.59449500	26.50838600
C	2.62747600	-3.44317500	25.72639000
O	2.70431900	-2.50497300	26.50838600
C	-1.66813900	-3.99704900	25.72639000
O	-0.81721100	-3.59449500	26.50838600
C	-4.29561500	-0.55387400	25.72639000
O	-3.52152900	-1.08952200	26.50838600
C	-2.62747600	3.44317500	25.72639000
O	-2.70431900	2.50497300	26.50838600
C	4.29561500	0.55387400	25.72639000
O	3.52152900	1.08952200	26.50838600
O	2.91586800	3.55274200	25.70336000
H	3.03396900	2.71653800	26.24038500
O	1.61883100	-4.30158600	25.70336000
H	0.83560600	-3.98576300	26.24038500
O	-2.91586800	-3.55274200	25.70336000
H	-3.03396900	-2.71653800	26.24038500
O	-4.53469800	0.74884500	25.70336000

H	-3.86957500	1.26922600	26.24038500
O	-1.61883100	4.30158600	25.70336000
H	-0.83560600	3.98576300	26.24038500
O	4.53469800	-0.74884500	25.70336000
H	3.86957500	-1.26922600	26.24038500
C	-1.59300600	-4.36716800	16.31262300
C	-1.15232900	-5.11110600	15.04621800
H	-1.17577800	-3.35010400	16.29951500
H	-2.68346600	-4.25447300	16.31910700
H	-0.05945800	-5.23486700	15.05370400
H	-1.57454100	-6.12699100	15.05282300
C	-2.98557500	3.56316700	16.31262300
C	-3.85018300	3.55349900	15.04621800
H	-2.31338600	2.69330500	16.29951500
H	-2.34274800	4.45118600	16.31910700
H	-4.50379900	2.66892500	15.05370400
H	-4.51885900	4.42708800	15.05282300
C	2.98557500	-3.56316700	16.31262300
C	3.85018300	-3.55349900	15.04621800
H	2.31338600	-2.69330500	16.29951500
H	2.34274800	-4.45118600	16.31910700
H	4.50379900	-2.66892500	15.05370400
H	4.51885900	-4.42708800	15.05282300
C	4.57858100	0.80400000	16.31262300
C	5.00251200	1.55760700	15.04621800
H	3.48916400	0.65679900	16.29951500
H	5.02621400	-0.19671300	16.31910700
H	4.56325700	2.56594100	15.05370400
H	6.09340000	1.69990300	15.05282300
C	-4.57858100	-0.80400000	16.31262300
C	-5.00251200	-1.55760700	15.04621800
H	-3.48916400	-0.65679900	16.29951500
H	-5.02621400	0.19671300	16.31910700
H	-4.56325700	-2.56594100	15.05370400
H	-6.09340000	-1.69990300	15.05282300

C	1.59300600	4.36716800	16.31262300
C	1.15232900	5.11110600	15.04621800
H	1.17577800	3.35010400	16.29951500
H	2.68346600	4.25447300	16.31910700
H	0.05945800	5.23486700	15.05370400
H	1.57454100	6.12699100	15.05282300
H	2.36347400	-4.44425900	13.75504000
H	2.34336300	-2.68704800	13.76350300
H	-1.15537100	-3.37293600	13.76350300
H	-2.66710400	-4.26895800	13.75504000
H	-3.49873300	-0.68588700	13.76350300
H	-5.03057900	0.17530100	13.75504000
H	-2.34336300	2.68704800	13.76350300
H	-2.36347400	4.44425900	13.75504000
H	2.66710400	4.26895800	13.75504000
H	1.15537100	3.37293600	13.76350300
H	5.03057900	-0.17530100	13.75504000
H	3.49873300	0.68588700	13.76350300
C	3.42921100	2.85742600	-22.36340200
O	4.40534800	2.10768700	-22.47447500
C	4.18920900	-1.54107100	-22.36340200
O	4.02798500	-2.76130000	-22.47447500
C	0.75999800	-4.39849700	-22.36340200
O	-0.37736300	-4.86898700	-22.47447500
C	-4.18920900	1.54107100	-22.36340200
O	-4.02798500	2.76130000	-22.47447500
C	-0.75999800	4.39849700	-22.36340200
O	0.37736300	4.86898700	-22.47447500
C	-3.42921100	-2.85742600	-22.36340200
O	-4.40534800	-2.10768700	-22.47447500
H	2.18776700	4.28128500	-23.16425400
H	4.80158500	0.24598000	-23.16425400
H	2.61381800	-4.03530500	-23.16425400
H	-4.80158500	-0.24598000	-23.16425400
H	-2.61381800	4.03530500	-23.16425400

H	-2.18776700	-4.28128500	-23.16425400
C	2.53891200	2.84824700	-21.12015300
H	1.88346000	3.71753800	-21.11235800
H	1.90279500	1.95078900	-21.19725700
C	3.73611000	-0.77463900	-21.12015300
H	4.16121300	0.22764500	-21.11235800
H	2.64083100	-0.67247500	-21.19725700
C	1.19719900	-3.62288600	-21.12015300
H	2.27775300	-3.48989400	-21.11235800
H	0.73803500	-2.62326400	-21.19725700
C	-3.73611000	0.77463900	-21.12015300
H	-4.16121300	-0.22764500	-21.11235800
H	-2.64083100	0.67247500	-21.19725700
C	-1.19719900	3.62288600	-21.12015300
H	-2.27775300	3.48989400	-21.11235800
H	-0.73803500	2.62326400	-21.19725700
C	-2.53891200	-2.84824700	-21.12015300
H	-1.88346000	-3.71753800	-21.11235800
H	-1.90279500	-1.95078900	-21.19725700
C	2.93494800	3.64961000	-18.84972100
O	1.98504300	4.44310600	-18.90992000
C	4.62812900	-0.71693500	-18.84972100
O	4.84036400	0.50245600	-18.90992000
C	1.69318100	-4.36654500	-18.84972100
O	2.85532200	-3.94065100	-18.90992000
C	-4.62812900	0.71693500	-18.84972100
O	-4.84036400	-0.50245600	-18.90992000
C	-1.69318100	4.36654500	-18.84972100
O	-2.85532200	3.94065100	-18.90992000
C	-2.93494800	-3.64961000	-18.84972100
O	-1.98504300	-4.44310600	-18.90992000
C	3.80924500	3.54267200	-17.60750300
H	4.43355000	2.64466600	-17.66089400
H	4.48719200	4.40832500	-17.62181600
C	4.97266700	-1.52756700	-17.60750300

H	4.50712300	-2.51723400	-17.66089400
H	6.06131800	-1.68185900	-17.62181600
C	1.16342200	-5.07023900	-17.60750300
H	0.07357300	-5.16190000	-17.66089400
H	1.57412600	-6.09018500	-17.62181600
C	-4.97266700	1.52756700	-17.60750300
H	-4.50712300	2.51723400	-17.66089400
H	-6.06131800	1.68185900	-17.62181600
C	-1.16342200	5.07023900	-17.60750300
H	-0.07357300	5.16190000	-17.66089400
H	-1.57412600	6.09018500	-17.62181600
C	-3.80924500	-3.54267200	-17.60750300
H	-4.43355000	-2.64466600	-17.66089400
H	-4.48719200	-4.40832500	-17.62181600
N	3.28966100	2.84225200	-19.88406500
N	4.10629300	-1.42780400	-19.88406500
N	0.81663200	-4.27005600	-19.88406500
N	-4.10629300	1.42780400	-19.88406500
N	-0.81663200	4.27005600	-19.88406500
N	-3.28966100	-2.84225200	-19.88406500
H	3.80656100	-2.39030200	-19.72042000
H	-0.16678200	-4.49173000	-19.72042000
H	3.97334300	2.10142800	-19.72042000
H	-3.80656100	2.39030200	-19.72042000
H	0.16678200	4.49173000	-19.72042000
H	-3.97334300	-2.10142800	-19.72042000
N	3.02036300	3.71530500	-23.32948300
N	4.72773000	-0.75805900	-23.32948300
N	1.70736700	-4.47336400	-23.32948300
N	-4.72773000	0.75805900	-23.32948300
N	-1.70736700	4.47336400	-23.32948300
N	-3.02036300	-3.71530500	-23.32948300
C	5.09252400	-1.27369200	-24.64113300
H	6.16177600	-1.11368700	-24.82896000
H	4.88004900	-2.34128700	-24.65766400

C	3.64931100	3.77341000	-24.64113300
H	4.04536900	4.77941100	-24.82896000
H	4.46763900	3.05560300	-24.65766400
C	-1.44321300	5.04710100	-24.64113300
H	-0.41241000	5.39689000	-24.65766400
H	-2.11640600	5.89309800	-24.82896000
C	-5.09252400	1.27369200	-24.64113300
H	-6.16177600	1.11368700	-24.82896000
H	-4.88004900	2.34128700	-24.65766400
C	-3.64931100	-3.77341000	-24.64113300
H	-4.46763900	-3.05560300	-24.65766400
H	-4.04536900	-4.77941100	-24.82896000
C	1.44321300	-5.04710100	-24.64113300
H	2.11640600	-5.89309800	-24.82896000
H	0.41241000	-5.39689000	-24.65766400
C	-2.62747600	-3.44317500	-25.72639000
O	-2.70431900	-2.50497300	-26.50838600
C	4.29561500	-0.55387400	-25.72639000
O	3.52152900	-1.08952200	-26.50838600
C	2.62747600	3.44317500	-25.72639000
O	2.70431900	2.50497300	-26.50838600
C	-1.66813900	3.99704900	-25.72639000
O	-0.81721100	3.59449500	-26.50838600
C	-4.29561500	0.55387400	-25.72639000
O	-3.52152900	1.08952200	-26.50838600
C	1.66813900	-3.99704900	-25.72639000
O	0.81721100	-3.59449500	-26.50838600
O	-1.61883100	-4.30158600	-25.70336000
H	-0.83560600	-3.98576300	-26.24038500
O	4.53469800	0.74884500	-25.70336000
H	3.86957500	1.26922600	-26.24038500
O	1.61883100	4.30158600	-25.70336000
H	0.83560600	3.98576300	-26.24038500
O	-2.91586800	3.55274200	-25.70336000
H	-3.03396900	2.71653800	-26.24038500

O	-4.53469800	-0.74884500	-25.70336000
H	-3.86957500	-1.26922600	-26.24038500
O	2.91586800	-3.55274200	-25.70336000
H	3.03396900	-2.71653800	-26.24038500
C	2.98557500	3.56316700	-16.31262300
C	3.85018300	3.55349900	-15.04621800
H	2.31338600	2.69330500	-16.29951500
H	2.34274800	4.45118600	-16.31910700
C	3.01556800	3.55802400	-13.75904800
H	4.50379900	2.66892500	-15.05370400
H	4.51885900	4.42708800	-15.05282300
C	-4.57858100	0.80400000	-16.31262300
C	-5.00251200	1.55760700	-15.04621800
H	-3.48916400	0.65679900	-16.29951500
H	-5.02621400	-0.19671300	-16.31910700
C	-4.58912300	0.83254700	-13.75904800
H	-4.56325700	2.56594100	-15.05370400
H	-6.09340000	1.69990300	-15.05282300
C	4.57858100	-0.80400000	-16.31262300
C	5.00251200	-1.55760700	-15.04621800
H	3.48916400	-0.65679900	-16.29951500
H	5.02621400	0.19671300	-16.31910700
C	4.58912300	-0.83254700	-13.75904800
H	4.56325700	-2.56594100	-15.05370400
H	6.09340000	-1.69990300	-15.05282300
C	1.59300600	-4.36716800	-16.31262300
C	1.15232900	-5.11110600	-15.04621800
H	1.17577800	-3.35010400	-16.29951500
H	2.68346600	-4.25447300	-16.31910700
C	1.57355500	-4.39057100	-13.75904800
H	0.05945800	-5.23486700	-15.05370400
H	1.57454100	-6.12699100	-15.05282300
C	-1.59300600	4.36716800	-16.31262300
C	-1.15232900	5.11110600	-15.04621800
H	-1.17577800	3.35010400	-16.29951500

H	-2.68346600	4.25447300	-16.31910700
C	-1.57355500	4.39057100	-13.75904800
H	-0.05945800	5.23486700	-15.05370400
H	-1.57454100	6.12699100	-15.05282300
C	-2.98557500	-3.56316700	-16.31262300
C	-3.85018300	-3.55349900	-15.04621800
H	-2.31338600	-2.69330500	-16.29951500
H	-2.34274800	-4.45118600	-16.31910700
C	-3.01556800	-3.55802400	-13.75904800
H	-4.50379900	-2.66892500	-15.05370400
H	-4.51885900	-4.42708800	-15.05282300
C	3.46923100	2.80378600	-5.19690000
O	4.44310200	2.04575000	-5.11684100
C	4.16276500	-1.60254900	-5.19690000
O	3.99322300	-2.82496400	-5.11684100
C	0.69353500	-4.40633500	-5.19690000
O	-0.44987900	-4.87071400	-5.11684100
C	-4.16276500	1.60254900	-5.19690000
O	-3.99322300	2.82496400	-5.11684100
C	-0.69353500	4.40633500	-5.19690000
O	0.44987900	4.87071400	-5.11684100
C	-3.46923100	-2.80378600	-5.19690000
O	-4.44310200	-2.04575000	-5.11684100
H	2.32702900	4.29712600	-4.36424000
H	4.88493500	0.13329600	-4.36424000
H	2.55790600	-4.16383000	-4.36424000
H	-4.88493500	-0.13329600	-4.36424000
H	-2.55790600	4.16383000	-4.36424000
H	-2.32702900	-4.29712600	-4.36424000
C	2.52935100	2.77384900	-6.40191500
H	1.86789100	3.63844400	-6.39387600
H	1.90846400	1.86863100	-6.30785600
C	3.66689900	-0.80355700	-6.40191500
H	4.08493000	0.20158100	-6.39387600
H	2.57251400	-0.71846200	-6.30785600

C	1.13754900	-3.57740700	-6.40191500
H	2.21704000	-3.43686300	-6.39387600
H	0.66405000	-2.58709400	-6.30785600
C	-3.66689900	0.80355700	-6.40191500
H	-4.08493000	-0.20158100	-6.39387600
H	-2.57251400	0.71846200	-6.30785600
C	-1.13754900	3.57740700	-6.40191500
H	-2.21704000	3.43686300	-6.39387600
H	-0.66405000	2.58709400	-6.30785600
C	-2.52935100	-2.77384900	-6.40191500
H	-1.86789100	-3.63844400	-6.39387600
H	-1.90846400	-1.86863100	-6.30785600
C	2.93015900	3.60964600	-8.66814900
O	2.00196400	4.42816000	-8.59708900
C	4.59112500	-0.73276900	-8.66814900
O	4.83588100	0.48032900	-8.59708900
C	1.66096600	-4.34241600	-8.66814900
O	2.83391800	-3.94783200	-8.59708900
C	-4.59112500	0.73276900	-8.66814900
O	-4.83588100	-0.48032900	-8.59708900
C	-1.66096600	4.34241600	-8.66814900
O	-2.83391800	3.94783200	-8.59708900
C	-2.93015900	-3.60964600	-8.66814900
O	-2.00196400	-4.42816000	-8.59708900
C	3.80620500	3.50686200	-9.90991900
H	4.42678100	2.60609800	-9.86268000
H	4.48689100	4.37012900	-9.88865100
C	4.94013400	-1.54283900	-9.90991900
H	4.47033800	-2.53065600	-9.86268000
H	6.02808800	-1.70069700	-9.88865100
C	1.13392900	-5.04970100	-9.90991900
H	0.04355700	-5.13675400	-9.86268000
H	1.54119700	-6.07082600	-9.88865100
C	-4.94013400	1.54283900	-9.90991900
H	-4.47033800	2.53065600	-9.86268000

H	-6.02808800	1.70069700	-9.88865100
C	-1.13392900	5.04970100	-9.90991900
H	-0.04355700	5.13675400	-9.86268000
H	-1.54119700	6.07082600	-9.88865100
C	-3.80620500	-3.50686200	-9.90991900
H	-4.42678100	-2.60609800	-9.86268000
H	-4.48689100	-4.37012900	-9.88865100
N	3.25753300	2.76957600	-7.65345400
N	4.02729000	-1.43631900	-7.65345400
N	0.76975700	-4.20589500	-7.65345400
N	-4.02729000	1.43631900	-7.65345400
N	-0.76975700	4.20589500	-7.65345400
N	-3.25753300	-2.76957600	-7.65345400
H	3.73261600	-2.40039300	-7.82258100
H	-0.21249400	-4.43273700	-7.82258100
H	3.94511000	2.03234400	-7.82258100
H	-3.73261600	2.40039300	-7.82258100
H	0.21249400	4.43273700	-7.82258100
H	-3.94511000	-2.03234400	-7.82258100
N	3.13981600	3.69707800	-4.23218400
N	4.77167200	-0.87062100	-4.23218400
N	1.63185600	-4.56770000	-4.23218400
N	-4.77167200	0.87062100	-4.23218400
N	-1.63185600	4.56770000	-4.23218400
N	-3.13981600	-3.69707800	-4.23218400
C	5.32884000	-1.51946400	-3.06305700
H	6.41909300	-1.64027300	-3.16284700
H	4.90186100	-2.52017800	-2.99262200
C	3.98031400	3.85517900	-3.06305700
H	4.63006400	4.73896100	-3.16284700
H	4.63346900	2.98504700	-2.99262200
C	-1.34852500	5.37464300	-3.06305700
H	-0.26839200	5.50522500	-2.99262200
H	-1.78902800	6.37923400	-3.16284700
C	-5.32884000	1.51946400	-3.06305700

H	-6.41909300	1.64027300	-3.16284700
H	-4.90186100	2.52017800	-2.99262200
C	-3.98031400	-3.85517900	-3.06305700
H	-4.63346900	-2.98504700	-2.99262200
H	-4.63006400	-4.73896100	-3.16284700
C	1.34852500	-5.37464300	-3.06305700
H	1.78902800	-6.37923400	-3.16284700
H	0.26839200	-5.50522500	-2.99262200
C	-3.25280800	-4.01587500	-1.74306700
O	-3.81320900	-3.71772400	-0.69330800
C	5.10425400	-0.80907700	-1.74306700
O	5.12624800	-1.44347400	-0.69330800
C	3.25280800	4.01587500	-1.74306700
O	3.81320900	3.71772400	-0.69330800
C	-1.85144600	4.82495200	-1.74306700
O	-1.31303900	5.16119800	-0.69330800
C	-5.10425400	0.80907700	-1.74306700
O	-5.12624800	1.44347400	-0.69330800
C	1.85144600	-4.82495200	-1.74306700
O	1.31303900	-5.16119800	-0.69330800
O	-2.04664500	-4.55139600	-1.82541700
H	-1.70648600	-4.73599100	-0.89814100
O	4.96494700	0.50325200	-1.82541700
H	4.95473100	0.89013500	-0.89814100
O	2.04664500	4.55139600	-1.82541700
H	1.70648600	4.73599100	-0.89814100
O	-2.91830300	4.04814500	-1.82541700
H	-3.24824600	3.84585500	-0.89814100
O	-4.96494700	-0.50325200	-1.82541700
H	-4.95473100	-0.89013500	-0.89814100
O	2.91830300	-4.04814500	-1.82541700
H	3.24824600	-3.84585500	-0.89814100
C	2.98372800	3.54098300	-11.20549500
C	3.84921400	3.54100900	-12.47135000
H	2.30943800	2.67285600	-11.22715300

H	2.34341800	4.43075200	-11.19147600
H	4.50156200	2.65547500	-12.47119200
H	4.51911100	4.41355900	-12.45651400
C	-4.55844500	0.81349200	-11.20549500
C	-4.99121100	1.56301300	-12.47135000
H	-3.46948000	0.66360400	-11.22715300
H	-5.00885300	-0.18591600	-11.19147600
H	-4.55049000	2.57073000	-12.47119200
H	-6.08181000	1.70688500	-12.45651400
C	4.55844500	-0.81349200	-11.20549500
C	4.99121100	-1.56301300	-12.47135000
H	3.46948000	-0.66360400	-11.22715300
H	5.00885300	0.18591600	-11.19147600
H	4.55049000	-2.57073000	-12.47119200
H	6.08181000	-1.70688500	-12.45651400
C	1.57471800	-4.35447600	-11.20549500
C	1.14199700	-5.10402200	-12.47135000
H	1.16004300	-3.33646000	-11.22715300
H	2.66543500	-4.24483600	-11.19147600
H	0.04892800	-5.22620500	-12.47119200
H	1.56269900	-6.12044500	-12.45651400
C	-1.57471800	4.35447600	-11.20549500
C	-1.14199700	5.10402200	-12.47135000
H	-1.16004300	3.33646000	-11.22715300
H	-2.66543500	4.24483600	-11.19147600
H	-0.04892800	5.22620500	-12.47119200
H	-1.56269900	6.12044500	-12.45651400
C	-2.98372800	-3.54098300	-11.20549500
C	-3.84921400	-3.54100900	-12.47135000
H	-2.30943800	-2.67285600	-11.22715300
H	-2.34341800	-4.43075200	-11.19147600
H	-4.50156200	-2.65547500	-12.47119200
H	-4.51911100	-4.41355900	-12.45651400
H	5.03057900	0.17530100	-13.75504000
H	3.49873300	-0.68588700	-13.76350300

H	2.34336300	2.68704800	-13.76350300
H	2.36347400	4.44425900	-13.75504000
H	-1.15537100	3.37293600	-13.76350300
H	-2.66710400	4.26895800	-13.75504000
H	-3.49873300	0.68588700	-13.76350300
H	-5.03057900	-0.17530100	-13.75504000
H	-2.36347400	-4.44425900	-13.75504000
H	-2.34336300	-2.68704800	-13.76350300
H	2.66710400	-4.26895800	-13.75504000
H	1.15537100	-3.37293600	-13.76350300

$[HCONHCH_2\text{-}COOH]_4$ Dimer

C	2.55201100	0.67102900	5.18149600
O	3.10134400	-0.43201000	5.20475800
C	-0.67102900	2.55201100	5.18149600
O	0.43201000	3.10134400	5.20475800
C	-2.55201100	-0.67102900	5.18149600
O	-3.10134400	0.43201000	5.20475800
C	0.67102900	-2.55201100	5.18149600
O	-0.43201000	-3.10134400	5.20475800
H	-2.43693600	2.11733800	4.29331200
H	2.11733800	2.43693600	4.29331200
H	-2.11733800	-2.43693600	4.29331200
H	2.43693600	-2.11733800	4.29331200
N	-1.60339100	2.69676500	4.21647300
N	2.69676500	1.60339100	4.21647300
N	-2.69676500	-1.60339100	4.21647300
N	1.60339100	-2.69676500	4.21647300
C	3.54710700	1.38236600	3.06927100
H	4.49436500	1.93609100	3.15880800
H	3.80280800	0.32191500	3.02934100
C	-1.38236600	3.54710700	3.06927100
H	-1.93609100	4.49436500	3.15880800
H	-0.32191500	3.80280800	3.02934100
C	-3.54710700	-1.38236600	3.06927100

H	-4.49436500	-1.93609100	3.15880800
H	-3.80280800	-0.32191500	3.02934100
C	1.38236600	-3.54710700	3.06927100
H	0.32191500	-3.80280800	3.02934100
H	1.93609100	-4.49436500	3.15880800
C	-1.78267600	2.94977300	1.73410700
O	-1.36990900	3.44351400	0.68881200
C	2.94977300	1.78267600	1.73410700
O	3.44351400	1.36990900	0.68881200
C	-2.94977300	-1.78267600	1.73410700
O	-3.44351400	-1.36990900	0.68881200
C	1.78267600	-2.94977300	1.73410700
O	1.36990900	-3.44351400	0.68881200
O	-2.62687500	1.93501500	1.80443800
H	-2.91833700	1.67675200	0.87669500
O	1.93501500	2.62687500	1.80443800
H	1.67675200	2.91833700	0.87669500
O	-1.93501500	-2.62687500	1.80443800
H	-1.67675200	-2.91833700	0.87669500
O	2.62687500	-1.93501500	1.80443800
H	2.91833700	-1.67675200	0.87669500
C	-0.67102900	-2.55201100	-5.18149600
O	0.43201000	-3.10134400	-5.20475800
C	-2.55201100	0.67102900	-5.18149600
O	-3.10134400	-0.43201000	-5.20475800
C	0.67102900	2.55201100	-5.18149600
O	-0.43201000	3.10134400	-5.20475800
C	2.55201100	-0.67102900	-5.18149600
O	3.10134400	0.43201000	-5.20475800
H	-2.11733800	2.43693600	-4.29331200
H	-2.43693600	-2.11733800	-4.29331200
H	2.43693600	2.11733800	-4.29331200
H	2.11733800	-2.43693600	-4.29331200
N	-2.69676500	1.60339100	-4.21647300
N	-1.60339100	-2.69676500	-4.21647300

N	1.60339100	2.69676500	-4.21647300
N	2.69676500	-1.60339100	-4.21647300
C	-1.38236600	-3.54710700	-3.06927100
H	-1.93609100	-4.49436500	-3.15880800
H	-0.32191500	-3.80280800	-3.02934100
C	-3.54710700	1.38236600	-3.06927100
H	-4.49436500	1.93609100	-3.15880800
H	-3.80280800	0.32191500	-3.02934100
C	1.38236600	3.54710700	-3.06927100
H	1.93609100	4.49436500	-3.15880800
H	0.32191500	3.80280800	-3.02934100
C	3.54710700	-1.38236600	-3.06927100
H	3.80280800	-0.32191500	-3.02934100
H	4.49436500	-1.93609100	-3.15880800
C	-2.94977300	1.78267600	-1.73410700
O	-3.44351400	1.36990900	-0.68881200
C	-1.78267600	-2.94977300	-1.73410700
O	-1.36990900	-3.44351400	-0.68881200
C	1.78267600	2.94977300	-1.73410700
O	1.36990900	3.44351400	-0.68881200
C	2.94977300	-1.78267600	-1.73410700
O	3.44351400	-1.36990900	-0.68881200
O	-1.93501500	2.62687500	-1.80443800
H	-1.67675200	2.91833700	-0.87669500
O	-2.62687500	-1.93501500	-1.80443800
H	-2.91833700	-1.67675200	-0.87669500
O	2.62687500	1.93501500	-1.80443800
H	2.91833700	1.67675200	-0.87669500
O	1.93501500	-2.62687500	-1.80443800
H	1.67675200	-2.91833700	-0.87669500
H	1.87280000	1.01116500	5.97892500
H	-1.01116500	1.87280000	5.97892500
H	1.01116500	-1.87280000	5.97892500
H	-1.87280000	-1.01116500	5.97892500
H	-1.01116500	-1.87280000	-5.97892500

H	1.87280000	-1.01116500	-5.97892500
H	-1.87280000	1.01116500	-5.97892500
H	1.01116500	1.87280000	-5.97892500

[HCONHCH$_2$-COOH]$_8$ Dimer

C	-0.12138500	5.54308600	-5.12209300
O	-1.34779000	5.64707800	-5.19812000
C	-3.97776700	3.81098900	-5.12194500
O	-4.91507400	3.01320900	-5.19594700
C	3.81766600	4.01700600	-5.11805100
O	3.03213800	4.96458700	-5.19395500
C	5.50413400	0.13683500	-5.11840200
O	5.61154300	1.36299700	-5.19248000
H	5.37274900	3.03509900	-4.28122600
H	1.66855900	5.95887700	-4.28230000
H	-3.01394400	5.37918200	-4.28917300
H	5.92160700	-1.65650300	-4.28660000
N	4.80340200	3.87785000	-4.20928000
N	0.66852400	6.14426900	-4.21020600
N	-3.84986600	4.79999700	-4.21498400
N	6.11037200	-0.65719800	-4.21337400
C	0.13774300	7.01094200	-3.18132000
H	0.35380300	8.06820700	-3.40261200
H	-0.94551900	6.89636600	-3.14858300
C	5.05265500	4.86735400	-3.18429800
H	5.95428400	5.45741700	-3.41317700
H	4.20888800	5.55601700	-3.14714300
C	6.98815800	-0.13161200	-3.19108100
H	8.04246300	-0.35211100	-3.42186400
H	6.87816900	0.95200700	-3.15563100
C	-4.84130100	5.04008100	-3.18986300
H	-5.43326400	5.94153900	-3.41419900
H	-5.52766000	4.19420100	-3.15900900
C	5.25550200	4.28667100	-1.79612900
O	4.88671100	4.89471700	-0.79661100
C	0.68787000	6.73823600	-1.79262300

O	-0.00526800	6.90594700	-0.79450400
C	6.72416900	-0.68337400	-1.80134400
O	6.89155100	0.01021800	-0.80346700
C	-4.26386700	5.23608100	-1.79920000
O	-4.87309200	4.85984800	-0.80313800
O	5.90456000	3.13433500	-1.78164200
H	6.08115300	2.85354100	-0.83372100
O	1.96064900	6.37930400	-1.77566400
H	2.28083500	6.30101700	-0.82700200
O	6.37278400	-1.95831100	-1.78387300
H	6.30045000	-2.28013400	-0.83530700
O	-3.11326700	5.88792300	-1.77843100
H	-2.83446800	6.05871800	-0.82885400
C	0.12138500	-5.54308600	-5.12209300
O	1.34779000	-5.64707800	-5.19812000
C	3.97776700	-3.81098900	-5.12194500
O	4.91507400	-3.01320900	-5.19594700
C	-3.81766600	-4.01700600	-5.11805100
O	-3.03213800	-4.96458700	-5.19395500
C	-5.50413400	-0.13683500	-5.11840200
O	-5.61154300	-1.36299700	-5.19248000
H	-5.37274900	-3.03509900	-4.28122600
H	-1.66855900	-5.95887700	-4.28230000
H	3.01394400	-5.37918200	-4.28917300
H	-5.92160700	1.65650300	-4.28660000
N	-4.80340200	-3.87785000	-4.20928000
N	-0.66852400	-6.14426900	-4.21020600
N	3.84986600	-4.79999700	-4.21498400
N	-6.11037200	0.65719800	-4.21337400
C	-0.13774300	-7.01094200	-3.18132000
H	-0.35380300	-8.06820700	-3.40261200
H	0.94551900	-6.89636600	-3.14858300
C	-5.05265500	-4.86735400	-3.18429800
H	-5.95428400	-5.45741700	-3.41317700
H	-4.20888800	-5.55601700	-3.14714300

C	-6.98815800	0.13161200	-3.19108100
H	-8.04246300	0.35211100	-3.42186400
H	-6.87816900	-0.95200700	-3.15563100
C	4.84130100	-5.04008100	-3.18986300
H	5.43326400	-5.94153900	-3.41419900
H	5.52766000	-4.19420100	-3.15900900
C	-5.25550200	-4.28667100	-1.79612900
O	-4.88671100	-4.89471700	-0.79661100
C	-0.68787000	-6.73823600	-1.79262300
O	0.00526800	-6.90594700	-0.79450400
C	-6.72416900	0.68337400	-1.80134400
O	-6.89155100	-0.01021800	-0.80346700
C	4.26386700	-5.23608100	-1.79920000
O	4.87309200	-4.85984800	-0.80313800
O	-5.90456000	-3.13433500	-1.78164200
H	-6.08115300	-2.85354100	-0.83372100
O	-1.96064900	-6.37930400	-1.77566400
H	-2.28083500	-6.30101700	-0.82700200
O	-6.37278400	1.95831100	-1.78387300
H	-6.30045000	2.28013400	-0.83530700
O	3.11326700	-5.88792300	-1.77843100
H	2.83446800	-6.05871800	-0.82885400
C	-1.91376800	5.16139200	5.12135400
O	-0.80856000	5.70320200	5.19473900
C	2.31639800	4.99504000	5.11802000
O	3.48461200	4.60741800	5.19239100
C	-5.03835000	2.31430100	5.12234800
O	-4.64618700	3.48094400	5.19849900
C	-5.20721700	-1.90682700	5.11801400
O	-5.76446300	-0.80936900	5.19385000
H	-6.13219800	0.83754700	4.28320400
H	-3.73647900	4.90225900	4.28948800
H	0.83622300	6.09011000	4.28618600
H	-4.92381700	-3.72368100	4.28085900
N	-5.90456000	1.82880000	4.21081800

N	-2.87239100	5.43844800	4.21506400
N	1.82846800	5.86719100	4.21335400
N	-5.47114300	-2.86647400	4.20894800
C	-2.69818300	6.44380400	3.19020800
H	-3.28926900	7.34572400	3.41502500
H	-1.64895000	6.73672500	3.15923100
C	-6.48892900	2.66062900	3.18224600
H	-7.54509000	2.88202600	3.40349500
H	-5.95341900	3.60925400	3.15030200
C	-6.47752600	-2.70148000	3.18350800
H	-7.37761500	-3.29419800	3.41158300
H	-6.77413600	-1.65345800	3.14673100
C	2.65947800	6.46431900	3.19136400
H	2.87612600	7.51933200	3.42253000
H	3.61010800	5.93271000	3.15566400
C	-6.45674300	2.04806500	1.79329800
O	-6.33536600	2.75124400	0.79548700
C	-3.10831200	5.99287700	1.79944200
O	-2.52261600	6.40480000	0.80336600
C	-6.02503800	-3.11801600	1.79527700
O	-6.43922800	-2.53986500	0.79577000
C	2.04812200	6.44180900	1.80165200
O	2.75116000	6.32163000	0.80361800
O	-6.63268900	0.73735700	1.77570200
H	-6.68796300	0.41302000	0.82676300
O	-4.16235000	5.19428000	1.77860500
H	-4.43063200	5.00735700	0.82904200
O	-5.22201500	-4.16883700	1.78072600
H	-5.03433600	-4.44231500	0.83277800
O	0.73830600	6.62422800	1.78441700
H	0.41423800	6.68618800	0.83589700
C	1.91376800	-5.16139200	5.12135400
O	0.80856000	-5.70320200	5.19473900
C	-2.31639800	-4.99504000	5.11802000
O	-3.48461200	-4.60741800	5.19239100

C	5.03835000	-2.31430100	5.12234800
O	4.64618700	-3.48094400	5.19849900
C	5.20721700	1.90682700	5.11801400
O	5.76446300	0.80936900	5.19385000
H	6.13219800	-0.83754700	4.28320400
H	3.73647900	-4.90225900	4.28948800
H	-0.83622300	-6.09011000	4.28618600
H	4.92381700	3.72368100	4.28085900
N	5.90456000	-1.82880000	4.21081800
N	2.87239100	-5.43844800	4.21506400
N	-1.82846800	-5.86719100	4.21335400
N	5.47114300	2.86647400	4.20894800
C	2.69818300	-6.44380400	3.19020800
H	3.28926900	-7.34572400	3.41502500
H	1.64895000	-6.73672500	3.15923100
C	6.48892900	-2.66062900	3.18224600
H	7.54509000	-2.88202600	3.40349500
H	5.95341900	-3.60925400	3.15030200
C	6.47752600	2.70148000	3.18350800
H	7.37761500	3.29419800	3.41158300
H	6.77413600	1.65345800	3.14673100
C	-2.65947800	-6.46431900	3.19136400
H	-2.87612600	-7.51933200	3.42253000
H	-3.61010800	-5.93271000	3.15566400
C	6.45674300	-2.04806500	1.79329800
O	6.33536600	-2.75124400	0.79548700
C	3.10831200	-5.99287700	1.79944200
O	2.52261600	-6.40480000	0.80336600
C	6.02503800	3.11801600	1.79527700
O	6.43922800	2.53986500	0.79577000
C	-2.04812200	-6.44180900	1.80165200
O	-2.75116000	-6.32163000	0.80361800
O	6.63268900	-0.73735700	1.77570200
H	6.68796300	-0.41302000	0.82676300
O	4.16235000	-5.19428000	1.77860500

H	4.43063200	-5.00735700	0.82904200
O	5.22201500	4.16883700	1.78072600
H	5.03433600	4.44231500	0.83277800
O	-0.73830600	-6.62422800	1.78441700
H	-0.41423800	-6.68618800	0.83589700
H	4.41824400	2.21503200	5.82034600
H	-4.41824400	-2.21503200	5.82034600
H	4.70784700	-1.53634100	5.82687300
H	-4.70784700	1.53634100	5.82687300
H	2.23073700	-4.37642300	5.82434200
H	-2.23073700	4.37642300	5.82434200
H	-1.53880500	-4.65472400	5.81822700
H	1.53880500	4.65472400	5.81822700
H	-3.78662700	-3.17037300	-5.82018700
H	3.78662700	3.17037300	-5.82018700
H	-0.46099700	-4.93093700	-5.82698000
H	0.46099700	4.93093700	-5.82698000
H	3.13162300	-3.78964500	-5.82508100
H	-3.13162300	3.78964500	-5.82508100
H	4.88396500	-0.44189300	-5.81927800
H	-4.88396500	0.44189300	-5.81927800

[HCONHCH$_2$-COOH]$_{10}$ Dimer

C	6.43749100	-2.62555700	5.05510200
O	7.00051500	-1.53137000	5.12953700
C	6.78889700	1.67632000	5.05493100
O	6.60864800	2.89355000	5.13130600
C	3.64530000	-5.91991600	5.05785700
O	4.73934700	-5.35665100	5.13247100
C	-4.53622600	-5.35242700	5.05623300
O	-3.67051900	-6.22688500	5.13270700
C	-0.54004400	-6.97118200	5.05845100
O	0.67582600	-7.16025100	5.13471600
H	2.36701400	-7.27012300	4.26840600
H	6.18545400	-4.46906800	4.26859000
H	7.65564000	0.03270000	4.26219400

H	-6.20386700	-4.53730900	4.25912300
H	-2.36604900	-7.30532800	4.26175100
N	-1.38208100	-7.56393700	4.18863800
N	3.31503900	-6.90102500	4.19448200
N	6.74244600	-3.61773400	4.19506100
N	7.61063200	1.04911300	4.18989600
N	-5.56548200	-5.32948500	4.18617800
C	7.80729800	-3.50079000	3.22257400
H	8.66935600	-4.12580200	3.50392500
H	8.14228000	-2.46468400	3.18912000
C	4.24737800	-7.42103700	3.21808900
H	4.58343800	-8.43319700	3.49297000
H	5.12394900	-6.77482400	3.18809700
C	-0.93143300	-8.52573400	3.20644000
H	0.15758900	-8.51606800	3.17682500
H	-1.25242800	-9.54451700	3.47489000
C	-5.77310600	-6.37113100	3.20386400
H	-6.63273200	-7.00417000	3.47472800
H	-4.88848900	-7.00616700	3.17060900
C	8.40169600	1.76650700	3.21374000
H	9.46706200	1.76898100	3.49311500
H	8.06368900	2.80155500	3.17744600
C	3.67719200	-7.51481500	1.81308100
O	4.36825300	-7.27536700	0.82842100
C	7.40367900	-3.91637000	1.81817200
O	7.82236100	-3.31920500	0.83201500
C	-1.44794800	-8.25923300	1.80286900
O	-0.74940400	-8.47086300	0.81712400
C	-6.03755100	-5.84949100	1.80186100
O	-5.59981000	-6.42972100	0.81387000
C	8.31718600	1.18847000	1.81146900
O	8.30804000	1.91455100	0.82298200
O	-2.70483600	-7.84915000	1.76365600
H	-3.00910700	-7.76358900	0.81082300
O	2.42093200	-7.92663200	1.77184300

H	2.12703800	-8.03976800	0.81866600
O	6.63039300	-4.98882500	1.77965800
H	6.46072700	-5.25692500	0.82726800
O	-6.81280200	-4.77835700	1.76673100
H	-7.01172000	-4.52885100	0.81488800
O	8.31667800	-0.13383500	1.77718600
H	8.33643700	-0.45343900	0.82577700
C	-6.43749100	2.62555700	5.05510200
O	-7.00051500	1.53137000	5.12953700
C	-6.78889700	-1.67632000	5.05493100
O	-6.60864800	-2.89355000	5.13130600
C	-3.64530000	5.91991600	5.05785700
O	-4.73934700	5.35665100	5.13247100
C	4.53622600	5.35242700	5.05623300
O	3.67051900	6.22688500	5.13270700
C	0.54004400	6.97118200	5.05845100
O	-0.67582600	7.16025100	5.13471600
H	-2.36701400	7.27012300	4.26840600
H	-6.18545400	4.46906800	4.26859000
H	-7.65564000	-0.03270000	4.26219400
H	6.20386700	4.53730900	4.25912300
H	2.36604900	7.30532800	4.26175100
N	1.38208100	7.56393700	4.18863800
N	-3.31503900	6.90102500	4.19448200
N	-6.74244600	3.61773400	4.19506100
N	-7.61063200	-1.04911300	4.18989600
N	5.56548200	5.32948500	4.18617800
C	-7.80729800	3.50079000	3.22257400
H	-8.66935600	4.12580200	3.50392500
H	-8.14228000	2.46468400	3.18912000
C	-4.24737800	7.42103700	3.21808900
H	-4.58343800	8.43319700	3.49297000
H	-5.12394900	6.77482400	3.18809700
C	0.93143300	8.52573400	3.20644000
H	-0.15758900	8.51606800	3.17682500

H	1.25242800	9.54451700	3.47489000
C	5.77310600	6.37113100	3.20386400
H	6.63273200	7.00417000	3.47472800
H	4.88848900	7.00616700	3.17060900
C	-8.40169600	-1.76650700	3.21374000
H	-9.46706200	-1.76898100	3.49311500
H	-8.06368900	-2.80155500	3.17744600
C	-3.67719200	7.51481500	1.81308100
O	-4.36825300	7.27536700	0.82842100
C	-7.40367900	3.91637000	1.81817200
O	-7.82236100	3.31920500	0.83201500
C	1.44794800	8.25923300	1.80286900
O	0.74940400	8.47086300	0.81712400
C	6.03755100	5.84949100	1.80186100
O	5.59981000	6.42972100	0.81387000
C	-8.31718600	-1.18847000	1.81146900
O	-8.30804000	-1.91455100	0.82298200
O	2.70483600	7.84915000	1.76365600
H	3.00910700	7.76358900	0.81082300
O	-2.42093200	7.92663200	1.77184300
H	-2.12703800	8.03976800	0.81866600
O	-6.63039300	4.98882500	1.77965800
H	-6.46072700	5.25692500	0.82726800
O	6.81280200	4.77835700	1.76673100
H	7.01172000	4.52885100	0.81488800
O	-8.31667800	0.13383500	1.77718600
H	-8.33643700	0.45343900	0.82577700
C	5.15687000	-4.73444700	-5.05718900
O	4.39875900	-5.70366800	-5.13334200
C	1.38208100	-6.82383600	-5.05854000
O	0.19818200	-7.15920700	-5.13425300
C	6.96528600	-0.82103900	-5.05528400
O	6.93194300	-2.05107800	-5.13205400
C	2.90540800	6.31224800	-5.05659600
O	4.06268600	5.89400100	-5.13086500

C	6.09503700	3.40274800	-5.05483500
O	6.79267400	2.38907100	-5.12998500
H	7.62558500	0.91405900	-4.25886600
H	6.71783800	-3.72986100	-4.26049000
H	3.23666300	-6.93636200	-4.26585700
H	1.46536900	7.49030900	-4.26992400
H	5.60954700	5.19758100	-4.26483100
N	6.26971800	4.42349900	-4.19197200
N	7.70199700	-0.10047600	-4.18642000
N	6.17653100	-4.59128000	-4.18728300
N	2.29174100	-7.31347000	-4.19263200
N	2.45291400	7.24591800	-4.19599100
C	6.50462300	-5.60095600	-3.20453400
H	7.43461800	-6.12631200	-3.47312400
H	5.70244700	-6.33752700	-3.17395700
C	8.56767800	-0.71858300	-3.20571800
H	9.62722300	-0.59385200	-3.47931600
H	8.35545600	-1.78659800	-3.17100100
C	7.33864800	4.43988700	-3.21705400
H	7.79866500	3.45299100	-3.18113700
H	8.11808600	5.16574400	-3.49746000
C	3.31209400	7.88467800	-3.22282800
H	3.51674000	8.93002300	-3.50267500
H	4.26347900	7.35488600	-3.19058700
C	1.96372700	-8.32774200	-3.21456700
H	2.40828600	-9.29778700	-3.48725400
H	0.88189100	-8.45285100	-3.18511800
C	8.40760100	-0.15559900	-1.80392200
O	8.48403500	-0.87765500	-0.81539600
C	6.70081800	-5.05254900	-1.80148800
O	6.33588900	-5.68354900	-0.81502900
C	6.88359700	4.80449200	-1.81430700
O	7.37233400	4.26652500	-0.82631500
C	2.73428700	7.91155500	-1.81805000
O	3.44857900	7.75998200	-0.83252400

C	2.44362500	-8.00495000	-1.80979600
O	1.77518000	-8.30166500	-0.82512200
O	5.98168200	5.77142000	-1.77915600
H	5.77846900	6.01817500	-0.82756800
O	8.24498800	1.15665800	-1.76969000
H	8.22168800	1.47561400	-0.81811200
O	7.33864800	-3.89442700	-1.76354300
H	7.50320000	-3.62351500	-0.81102800
O	1.43720900	8.16759000	-1.77835900
H	1.13093200	8.24661600	-0.82564700
O	3.64200200	-7.44667600	-1.76858300
H	3.93332700	-7.32742900	-0.81533100
C	-5.15687000	4.73444700	-5.05718900
O	-4.39875900	5.70366800	-5.13334200
C	-1.38208100	6.82383600	-5.05854000
O	-0.19818200	7.15920700	-5.13425300
C	-6.96528600	0.82103900	-5.05528400
O	-6.93194300	2.05107800	-5.13205400
C	-2.90540800	-6.31224800	-5.05659600
O	-4.06268600	-5.89400100	-5.13086500
C	-6.09503700	-3.40274800	-5.05483500
O	-6.79267400	-2.38907100	-5.12998500
H	-7.62558500	-0.91405900	-4.25886600
H	-6.71783800	3.72986100	-4.26049000
H	-3.23666300	6.93636200	-4.26585700
H	-1.46536900	-7.49030900	-4.26992400
H	-5.60954700	-5.19758100	-4.26483100
N	-6.26971800	-4.42349900	-4.19197200
N	-7.70199700	0.10047600	-4.18642000
N	-6.17653100	4.59128000	-4.18728300
N	-2.29174100	7.31347000	-4.19263200
N	-2.45291400	-7.24591800	-4.19599100
C	-6.50462300	5.60095600	-3.20453400
H	-7.43461800	6.12631200	-3.47312400
H	-5.70244700	6.33752700	-3.17395700

C	-8.56767800	0.71858300	-3.20571800
H	-9.62722300	0.59385200	-3.47931600
H	-8.35545600	1.78659800	-3.17100100
C	-7.33864800	-4.43988700	-3.21705400
H	-7.79866500	-3.45299100	-3.18113700
H	-8.11808600	-5.16574400	-3.49746000
C	-3.31209400	-7.88467800	-3.22282800
H	-3.51674000	-8.93002300	-3.50267500
H	-4.26347900	-7.35488600	-3.19058700
C	-1.96372700	8.32774200	-3.21456700
H	-2.40828600	9.29778700	-3.48725400
H	-0.88189100	8.45285100	-3.18511800
C	-8.40760100	0.15559900	-1.80392200
O	-8.48403500	0.87765500	-0.81539600
C	-6.70081800	5.05254900	-1.80148800
O	-6.33588900	5.68354900	-0.81502900
C	-6.88359700	-4.80449200	-1.81430700
O	-7.37233400	-4.26652500	-0.82631500
C	-2.73428700	-7.91155500	-1.81805000
O	-3.44857900	-7.75998200	-0.83252400
C	-2.44362500	8.00495000	-1.80979600
O	-1.77518000	8.30166500	-0.82512200
O	-5.98168200	-5.77142000	-1.77915600
H	-5.77846900	-6.01817500	-0.82756800
O	-8.24498800	-1.15665800	-1.76969000
H	-8.22168800	-1.47561400	-0.81811200
O	-7.33864800	3.89442700	-1.76354300
H	-7.50320000	3.62351500	-0.81102800
O	-1.43720900	-8.16759000	-1.77835900
H	-1.13093200	-8.24661600	-0.82564700
O	-3.64200200	7.44667600	-1.76858300
H	-3.93332700	7.32742900	-0.81533100
H	4.54877100	4.48015000	5.72690600
H	6.27783800	0.96537900	5.72140400
H	5.60514900	-2.90244400	5.71946300

H	2.80840500	-5.66436600	5.72505800
H	-1.06803100	-6.27688800	5.72929500
H	1.06803100	6.27688800	5.72929500
H	-2.80840500	5.66436600	5.72505800
H	-5.60514900	2.90244400	5.71946300
H	-6.27783800	-0.96537900	5.72140400
H	-4.54877100	-4.48015000	5.72690600
H	-2.10748500	-5.94891900	-5.72155200
H	-5.23569100	-3.57325200	-5.72061500
H	-6.37656000	0.17602000	-5.72472800
H	-5.06771000	3.86694200	-5.72825700
H	-1.82030200	6.06735900	-5.72679500
H	2.10748500	5.94891900	-5.72155200
H	5.23569100	3.57325200	-5.72061500
H	6.37656000	-0.17602000	-5.72472800
H	5.06771000	-3.86694200	-5.72825700
H	1.82030200	-6.06735900	-5.72679500

$[HCONHCH_2\text{-}COOH]_{16}$ Dimer

C	-6.32973500	9.47311800	4.88114100
O	-7.38439400	8.83894300	4.94502600
C	-2.22270700	11.17430400	4.88114100
O	-3.43977300	10.99200300	4.94502600
H	-0.57472500	12.12395700	4.19719300
H	-5.17061400	10.98113800	4.19719300
N	-1.59258300	12.09483300	4.12425800
N	-6.09984700	10.56471300	4.12425800
C	-7.10575000	11.13810700	3.25455000
H	-7.43467700	12.11903200	3.63029700
H	-7.96909200	10.47511900	3.22713500
C	-2.30248700	13.00952200	3.25455000
H	-2.23099400	14.04165400	3.63029700
H	-3.35382600	12.72738800	3.22713500
C	-1.77361300	13.01222000	1.82892300
O	-2.52666300	13.01052300	0.86128400
C	-6.61816600	11.34299100	1.82892300

O	-7.31324400	11.05324400	0.86128400
O	-0.45361300	13.06478100	1.75905700
H	-0.15733900	13.11126100	0.80202200
O	-5.41875900	11.89669300	1.75905700
H	-5.16282400	12.05301500	0.80202200
C	2.22270700	11.17430400	4.88114100
O	1.02852100	11.47163100	4.94502600
C	6.32973500	9.47311800	4.88114100
O	5.34023300	10.20480700	4.94502600
H	8.16654000	8.97932400	4.19719300
H	4.10866100	11.42101300	4.19719300
N	7.42621200	9.67846500	4.12425800
N	3.15713700	11.78362400	4.12425800
C	2.85130800	12.90035500	3.25455000
H	3.31233900	13.82656100	3.63029700
H	1.77202900	13.04202700	3.22713500
C	7.57101700	10.82722600	3.25455000
H	8.35139800	11.50649900	3.63029700
H	6.62810900	11.37113500	3.22713500
C	7.94689500	10.45516300	1.82892300
O	7.41320900	10.98645000	0.86128400
C	3.34095600	12.70045600	1.82892300
O	2.64457900	12.98706800	0.86128400
O	8.91744200	9.55894800	1.75905700
H	9.15980700	9.38231700	0.80202200
O	4.58059100	12.24387400	1.75905700
H	4.87210100	12.17343700	0.80202200
C	9.47311800	6.32973500	4.88114100
O	8.83894300	7.38439400	4.94502600
C	11.17430400	2.22270700	4.88114100
O	10.99200300	3.43977300	4.94502600
H	12.12395700	0.57472500	4.19719300
H	10.98113800	5.17061400	4.19719300
N	12.09483300	1.59258300	4.12425800
N	10.56471300	6.09984700	4.12425800

C	11.13810700	7.10575000	3.25455000
H	12.11903200	7.43467700	3.63029700
H	10.47511900	7.96909200	3.22713500
C	13.00952200	2.30248700	3.25455000
H	14.04165400	2.23099400	3.63029700
H	12.72738800	3.35382600	3.22713500
C	13.01222000	1.77361300	1.82892300
O	13.01052300	2.52666300	0.86128400
C	11.34299100	6.61816600	1.82892300
O	11.05324400	7.31324400	0.86128400
O	13.06478100	0.45361300	1.75905700
H	13.11126100	0.15733900	0.80202200
O	11.89669300	5.41875900	1.75905700
H	12.05301500	5.16282400	0.80202200
C	11.17430400	-2.22270700	4.88114100
O	11.47163100	-1.02852100	4.94502600
C	9.47311800	-6.32973500	4.88114100
O	10.20480700	-5.34023300	4.94502600
H	8.97932400	-8.16654000	4.19719300
H	11.42101300	-4.10866100	4.19719300
N	9.67846500	-7.42621200	4.12425800
N	11.78362400	-3.15713700	4.12425800
C	12.90035500	-2.85130800	3.25455000
H	13.82656100	-3.31233900	3.63029700
H	13.04202700	-1.77202900	3.22713500
C	10.82722600	-7.57101700	3.25455000
H	11.50649900	-8.35139800	3.63029700
H	11.37113500	-6.62810900	3.22713500
C	10.45516300	-7.94689500	1.82892300
O	10.98645000	-7.41320900	0.86128400
C	12.70045600	-3.34095600	1.82892300
O	12.98706800	-2.64457900	0.86128400
O	9.55894800	-8.91744200	1.75905700
H	9.38231700	-9.15980700	0.80202200
O	12.24387400	-4.58059100	1.75905700

H	12.17343700	-4.87210100	0.80202200
C	6.32973500	-9.47311800	4.88114100
O	7.38439400	-8.83894300	4.94502600
C	2.22270700	-11.17430400	4.88114100
O	3.43977300	-10.99200300	4.94502600
H	0.57472500	-12.12395700	4.19719300
H	5.17061400	-10.98113800	4.19719300
N	1.59258300	-12.09483300	4.12425800
N	6.09984700	-10.56471300	4.12425800
C	7.10575000	-11.13810700	3.25455000
H	7.43467700	-12.11903200	3.63029700
H	7.96909200	-10.47511900	3.22713500
C	2.30248700	-13.00952200	3.25455000
H	2.23099400	-14.04165400	3.63029700
H	3.35382600	-12.72738800	3.22713500
C	1.77361300	-13.01222000	1.82892300
O	2.52666300	-13.01052300	0.86128400
C	6.61816600	-11.34299100	1.82892300
O	7.31324400	-11.05324400	0.86128400
O	0.45361300	-13.06478100	1.75905700
H	0.15733900	-13.11126100	0.80202200
O	5.41875900	-11.89669300	1.75905700
H	5.16282400	-12.05301500	0.80202200
C	-2.22270700	-11.17430400	4.88114100
O	-1.02852100	-11.47163100	4.94502600
C	-6.32973500	-9.47311800	4.88114100
O	-5.34023300	-10.20480700	4.94502600
H	-8.16654000	-8.97932400	4.19719300
H	-4.10866100	-11.42101300	4.19719300
N	-7.42621200	-9.67846500	4.12425800
N	-3.15713700	-11.78362400	4.12425800
C	-2.85130800	-12.90035500	3.25455000
H	-3.31233900	-13.82656100	3.63029700
H	-1.77202900	-13.04202700	3.22713500
C	-7.57101700	-10.82722600	3.25455000

H	-8.35139800	-11.50649900	3.63029700
H	-6.62810900	-11.37113500	3.22713500
C	-7.94689500	-10.45516300	1.82892300
O	-7.41320900	-10.98645000	0.86128400
C	-3.34095600	-12.70045600	1.82892300
O	-2.64457900	-12.98706800	0.86128400
O	-8.91744200	-9.55894800	1.75905700
H	-9.15980700	-9.38231700	0.80202200
O	-4.58059100	-12.24387400	1.75905700
H	-4.87210100	-12.17343700	0.80202200
C	-9.47311800	-6.32973500	4.88114100
O	-8.83894300	-7.38439400	4.94502600
C	-11.17430400	-2.22270700	4.88114100
O	-10.99200300	-3.43977300	4.94502600
H	-12.12395700	-0.57472500	4.19719300
H	-10.98113800	-5.17061400	4.19719300
N	-12.09483300	-1.59258300	4.12425800
N	-10.56471300	-6.09984700	4.12425800
C	-11.13810700	-7.10575000	3.25455000
H	-12.11903200	-7.43467700	3.63029700
H	-10.47511900	-7.96909200	3.22713500
C	-13.00952200	-2.30248700	3.25455000
H	-14.04165400	-2.23099400	3.63029700
H	-12.72738800	-3.35382600	3.22713500
C	-13.01222000	-1.77361300	1.82892300
O	-13.01052300	-2.52666300	0.86128400
C	-11.34299100	-6.61816600	1.82892300
O	-11.05324400	-7.31324400	0.86128400
O	-13.06478100	-0.45361300	1.75905700
H	-13.11126100	-0.15733900	0.80202200
O	-11.89669300	-5.41875900	1.75905700
H	-12.05301500	-5.16282400	0.80202200
C	-11.17430400	2.22270700	4.88114100
O	-11.47163100	1.02852100	4.94502600
C	-9.47311800	6.32973500	4.88114100

O	-10.20480700	5.34023300	4.94502600
H	-8.97932400	8.16654000	4.19719300
H	-11.42101300	4.10866100	4.19719300
N	-9.67846500	7.42621200	4.12425800
N	-11.78362400	3.15713700	4.12425800
C	-12.90035500	2.85130800	3.25455000
H	-13.82656100	3.31233900	3.63029700
H	-13.04202700	1.77202900	3.22713500
C	-10.82722600	7.57101700	3.25455000
H	-11.50649900	8.35139800	3.63029700
H	-11.37113500	6.62810900	3.22713500
C	-10.45516300	7.94689500	1.82892300
O	-10.98645000	7.41320900	0.86128400
C	-12.70045600	3.34095600	1.82892300
O	-12.98706800	2.64457900	0.86128400
O	-9.55894800	8.91744200	1.75905700
H	-9.38231700	9.15980700	0.80202200
O	-12.24387400	4.58059100	1.75905700
H	-12.17343700	4.87210100	0.80202200
C	10.61366400	4.14267600	-4.88093800
O	11.11503400	3.01878500	-4.94462000
C	8.22041400	7.88900700	-4.88093800
O	9.11371300	7.04253300	-4.94462000
H	7.41315900	9.61133900	-4.19734200
H	10.52696600	6.04282600	-4.19734200
N	8.23097600	9.00465400	-4.12428000
N	11.05036200	5.16935800	-4.12428000
C	12.20345600	5.06360400	-3.25464800
H	13.03463100	5.67963900	-3.63043800
H	12.53170200	4.02572500	-3.22722400
C	9.33676600	9.34822100	-3.25464800
H	9.86892500	10.23544000	-3.63043800
H	10.03720500	8.51496000	-3.22722400
C	8.90443100	9.65310800	-1.82904900
O	9.51852300	9.21755300	-0.86128100

C	11.92070600	5.51073100	-1.82904900
O	12.32137300	4.87332700	-0.86128100
O	7.85469100	10.45513900	-1.75936500
H	7.63802000	10.66256900	-0.80234000
O	11.25779600	6.65342900	-1.75936500
H	11.13699900	6.92798500	-0.80234000
C	4.57568000	10.43430800	-4.88093800
O	5.72491200	9.99411900	-4.94462000
C	0.23434000	11.39108000	-4.88093800
O	1.46454600	11.42419100	-4.94462000
H	-1.55434800	12.03813800	-4.19734200
H	3.17076500	11.71661200	-4.19734200
N	-0.54707300	12.18743100	-4.12428000
N	4.15849800	11.46907400	-4.12428000
C	5.04863700	12.20965600	-3.25464800
H	5.20076400	13.23298700	-3.63043800
H	6.01463400	11.70786900	-3.22722400
C	-0.00810000	13.21228100	-3.25464800
H	-0.25916500	14.21593200	-3.63043800
H	1.07639000	13.11836100	-3.22722400
C	-0.52939500	13.12216200	-1.82904900
O	0.21281800	13.24840600	-0.86128100
C	4.53253700	12.32588700	-1.82904900
O	5.26656400	12.15848900	-0.86128100
O	-1.83879400	12.94700400	-1.75936500
H	-2.13867900	12.94047100	-0.80234000
O	3.25578000	12.66514900	-1.75936500
H	2.97622200	12.77387300	-0.80234000
C	-4.14267600	10.61366400	-4.88093800
O	-3.01878500	11.11503400	-4.94462000
C	-7.88900700	8.22041400	-4.88093800
O	-7.04253300	9.11371300	-4.94462000
H	-9.61133900	7.41315900	-4.19734200
H	-6.04282600	10.52696600	-4.19734200
N	-9.00465400	8.23097600	-4.12428000

N	-5.16935800	11.05036200	-4.12428000
C	-5.06360400	12.20345600	-3.25464800
H	-5.67963900	13.03463100	-3.63043800
H	-4.02572500	12.53170200	-3.22722400
C	-9.34822100	9.33676600	-3.25464800
H	-10.23544000	9.86892500	-3.63043800
H	-8.51496000	10.03720500	-3.22722400
C	-9.65310800	8.90443100	-1.82904900
O	-9.21755300	9.51852300	-0.86128100
C	-5.51073100	11.92070600	-1.82904900
O	-4.87332700	12.32137300	-0.86128100
O	-10.45513900	7.85469100	-1.75936500
H	-10.66256900	7.63802000	-0.80234000
O	-6.65342900	11.25779600	-1.75936500
H	-6.92798500	11.13699900	-0.80234000
C	-10.43430800	4.57568000	-4.88093800
O	-9.99411900	5.72491200	-4.94462000
C	-11.39108000	0.23434000	-4.88093800
O	-11.42419100	1.46454600	-4.94462000
H	-12.03813800	-1.55434800	-4.19734200
H	-11.71661200	3.17076500	-4.19734200
N	-12.18743100	-0.54707300	-4.12428000
N	-11.46907400	4.15849800	-4.12428000
C	-12.20965600	5.04863700	-3.25464800
H	-13.23298700	5.20076400	-3.63043800
H	-11.70786900	6.01463400	-3.22722400
C	-13.21228100	-0.00810000	-3.25464800
H	-14.21593200	-0.25916500	-3.63043800
H	-13.11836100	1.07639000	-3.22722400
C	-13.12216200	-0.52939500	-1.82904900
O	-13.24840600	0.21281800	-0.86128100
C	-12.32588700	4.53253700	-1.82904900
O	-12.15848900	5.26656400	-0.86128100
O	-12.94700400	-1.83879400	-1.75936500
H	-12.94047100	-2.13867900	-0.80234000

O	-12.66514900	3.25578000	-1.75936500
H	-12.77387300	2.97622200	-0.80234000
C	-10.61366400	-4.14267600	-4.88093800
O	-11.11503400	-3.01878500	-4.94462000
C	-8.22041400	-7.88900700	-4.88093800
O	-9.11371300	-7.04253300	-4.94462000
H	-7.41315900	-9.61133900	-4.19734200
H	-10.52696600	-6.04282600	-4.19734200
N	-8.23097600	-9.00465400	-4.12428000
N	-11.05036200	-5.16935800	-4.12428000
C	-12.20345600	-5.06360400	-3.25464800
H	-13.03463100	-5.67963900	-3.63043800
H	-12.53170200	-4.02572500	-3.22722400
C	-9.33676600	-9.34822100	-3.25464800
H	-9.86892500	-10.23544000	-3.63043800
H	-10.03720500	-8.51496000	-3.22722400
C	-8.90443100	-9.65310800	-1.82904900
O	-9.51852300	-9.21755300	-0.86128100
C	-11.92070600	-5.51073100	-1.82904900
O	-12.32137300	-4.87332700	-0.86128100
O	-7.85469100	-10.45513900	-1.75936500
H	-7.63802000	-10.66256900	-0.80234000
O	-11.25779600	-6.65342900	-1.75936500
H	-11.13699900	-6.92798500	-0.80234000
C	-4.57568000	-10.43430800	-4.88093800
O	-5.72491200	-9.99411900	-4.94462000
C	-0.23434000	-11.39108000	-4.88093800
O	-1.46454600	-11.42419100	-4.94462000
H	1.55434800	-12.03813800	-4.19734200
H	-3.17076500	-11.71661200	-4.19734200
N	0.54707300	-12.18743100	-4.12428000
N	-4.15849800	-11.46907400	-4.12428000
C	-5.04863700	-12.20965600	-3.25464800
H	-5.20076400	-13.23298700	-3.63043800
H	-6.01463400	-11.70786900	-3.22722400

C	0.00810000	-13.21228100	-3.25464800
H	0.25916500	-14.21593200	-3.63043800
H	-1.07639000	-13.11836100	-3.22722400
C	0.52939500	-13.12216200	-1.82904900
O	-0.21281800	-13.24840600	-0.86128100
C	-4.53253700	-12.32588700	-1.82904900
O	-5.26656400	-12.15848900	-0.86128100
O	1.83879400	-12.94700400	-1.75936500
H	2.13867900	-12.94047100	-0.80234000
O	-3.25578000	-12.66514900	-1.75936500
H	-2.97622200	-12.77387300	-0.80234000
C	4.14267600	-10.61366400	-4.88093800
O	3.01878500	-11.11503400	-4.94462000
C	7.88900700	-8.22041400	-4.88093800
O	7.04253300	-9.11371300	-4.94462000
H	9.61133900	-7.41315900	-4.19734200
H	6.04282600	-10.52696600	-4.19734200
N	9.00465400	-8.23097600	-4.12428000
N	5.16935800	-11.05036200	-4.12428000
C	5.06360400	-12.20345600	-3.25464800
H	5.67963900	-13.03463100	-3.63043800
H	4.02572500	-12.53170200	-3.22722400
C	9.34822100	-9.33676600	-3.25464800
H	10.23544000	-9.86892500	-3.63043800
H	8.51496000	-10.03720500	-3.22722400
C	9.65310800	-8.90443100	-1.82904900
O	9.21755300	-9.51852300	-0.86128100
C	5.51073100	-11.92070600	-1.82904900
O	4.87332700	-12.32137300	-0.86128100
O	10.45513900	-7.85469100	-1.75936500
H	10.66256900	-7.63802000	-0.80234000
O	6.65342900	-11.25779600	-1.75936500
H	6.92798500	-11.13699900	-0.80234000
C	10.43430800	-4.57568000	-4.88093800
O	9.99411900	-5.72491200	-4.94462000

C	11.39108000	-0.23434000	-4.88093800
O	11.42419100	-1.46454600	-4.94462000
H	12.03813800	1.55434800	-4.19734200
H	11.71661200	-3.17076500	-4.19734200
N	12.18743100	0.54707300	-4.12428000
N	11.46907400	-4.15849800	-4.12428000
C	12.20965600	-5.04863700	-3.25464800
H	13.23298700	-5.20076400	-3.63043800
H	11.70786900	-6.01463400	-3.22722400
C	13.21228100	0.00810000	-3.25464800
H	14.21593200	0.25916500	-3.63043800
H	13.11836100	-1.07639000	-3.22722400
C	13.12216200	0.52939500	-1.82904900
O	13.24840600	-0.21281800	-0.86128100
C	12.32588700	-4.53253700	-1.82904900
O	12.15848900	-5.26656400	-0.86128100
O	12.94700400	1.83879400	-1.75936500
H	12.94047100	2.13867900	-0.80234000
O	12.66514900	-3.25578000	-1.75936500
H	12.77387300	-2.97622200	-0.80234000
H	10.34991300	-2.65040500	5.47202200
H	4.41964500	-9.72720800	-5.47185000
H	10.57633900	1.51208600	5.47202200
H	0.36077800	-10.67809400	-5.47185000
H	9.19261300	5.44437500	5.47202200
H	-3.75301400	-10.00333600	-5.47185000
H	6.40939500	8.54780700	5.47202200
H	-7.29544400	-7.80566100	-5.47185000
H	2.65040500	10.34991300	5.47202200
H	-9.72720800	-4.41964500	-5.47185000
H	-1.51208600	10.57633900	5.47202200
H	-10.67809400	-0.36077800	-5.47185000
H	-5.44437500	9.19261300	5.47202200
H	-10.00333600	3.75301400	-5.47185000
H	-8.54780700	6.40939500	5.47202200

H	-7.80566100	7.29544400	-5.47185000
H	-10.34991300	2.65040500	5.47202200
H	-4.41964500	9.72720800	-5.47185000
H	-10.57633900	-1.51208600	5.47202200
H	-0.36077800	10.67809400	-5.47185000
H	-9.19261300	-5.44437500	5.47202200
H	3.75301400	10.00333600	-5.47185000
H	-6.40939500	-8.54780700	5.47202200
H	7.29544400	7.80566100	-5.47185000
H	-2.65040500	-10.34991300	5.47202200
H	9.72720800	4.41964500	-5.47185000
H	1.51208600	-10.57633900	5.47202200
H	10.67809400	0.36077800	-5.47185000
H	5.44437500	-9.19261300	5.47202200
H	10.00333600	-3.75301400	-5.47185000
H	8.54780700	-6.40939500	5.47202200
H	7.80566100	-7.29544400	-5.47185000
