# Supplementary material for: Hydrogen-Bond-Driven Peptide Nanotube Formation: A DFT Study
Source: Molecules. 2023 Aug 24;28(17):6217. doi: 10.3390/molecules28176217 (PMC10488343; doi:10.3390/molecules28176217)
Supplement: Supplementary file 1 [file molecules-28-06217-s001.zip › S_Captions.pdf]

Optimized geometries for [P-CH2-P]n Nanotubes, S1.

Optimized geometries for [P-(CH2)7-P]n Nanotubes, S2.

Optimized geometries for Nanotube Dimers, S3.
